# Hydrophobicity strategy drives contact-electro-catalysis on natural silicate minerals

**DOI:** 10.1038/s41467-026-77246-0

**Published:** 2026-08-29

**Authors:** Xinnan Li, Wangshu Tong, Shuchen Tu, Tianyang Hao, Xuxu Zhang, Tingting Cao, Yong Li, Qi An, Yizhi Sheng

**Affiliations:** 1 School of Materials Science and Technology, China University of Geosciences (Beijing), Beijing, China; 2 Beijing Key Laboratory of Materials Utilization of Nonmetallic Minerals and Solid Wastes, China University of Geosciences (Beijing), Beijing, China; 3 Engineering Research Center of Ministry of Education for Geological Carbon Storage and Low Carbon Utilization of Resources, China University of Geosciences (Beijing), Beijing, China; 4 School of Environment, South China Normal University, Guangzhou, China; 5 College of Geosciences and Surveying Engineering, China University of Mining and Technology-Beijing, Beijing, China; 6 State Key Laboratory of Geomicrobiology and Environmental Changes, China University of Geosciences (Beijing), Beijing, China

**Keywords:** Electrocatalysis, Heterogeneous catalysis, Pollution remediation, Carbon cycle

## Abstract

Contact-electro-catalysis (CEC) at catalyst-water interfaces shows promise for wastewater purification, metal recovery, and H_2_O_2_ production, but catalyst design has mainly emphasized electron-withdrawing ability while overlooking interfacial water transfer. Here, we develop a hydrophobicity engineering strategy for natural silicate minerals to optimize catalyst-water contact by balancing the water approach and removal. As a representative non-fluorinated system, alkyl-modified minerals with moderate hydrophobicity (water contact angle of 125–148°) achieve a H_2_O_2_ yield of 350.0 μmol·L^–1^·h^–1^ under ultrasonication, outperforming fluorinated minerals. Mechanistic analysis indicates that this enhancement arises from improved interfacial contact efficiency rather than stronger electron-withdrawing ability alone. This strategy also remains effective under weak-force conditions, enabling self-powered CEC in a natural river. The modified minerals selectively enriched microorganisms associated with oxidative stress resistance and oxidized organic carbon utilization, including *Novosphingobium* sp. Together, these findings identify hydrophobicity regulation as an effective contact-optimization strategy for CEC.

## Abstract (web summary)

Hydrophobicity regulation optimizes contact-electro-catalysis in silicate minerals, enhancing interfacial water transfer. Moderate hydrophobicity improves H_2_O_2_ production under various conditions, demonstrating effective performance.

## Introduction

Contact-electro-catalysis (CEC) is an emerging catalytic process in which mechanical energy drives interfacial electron transfer between solids and water, leading to the generation of reactive oxygen species (ROS)^1,2^. Because ROS can mediate pollutant degradation^3^, metal recovery^4^, and H_2_O_2_ production^5^, CEC has attracted increasing attention as a potential strategy for environmental and chemical transformations. To date, ultrasonic excitation has been the dominant approach for activating CEC because it provides sufficient energy to induce electron-cloud overlap between H_2_O and catalyst surfaces^6^. However, ultrasonic systems are energy-intensive, instrument-dependent, and often accompanied by noise pollution, which limits their broader practical deployment^7,8^. In contrast, hydrodynamic forces such as flowing water represent a more sustainable and widely available energy source, but the CEC under such weak-force conditions remains underexplored.

Current CEC systems operating under weak mechanical excitation still rely largely on perfluorinated materials, such as fluorinated ethylene propylene (FEP) and polytetrafluoroethylene (PTFE), owing to their strong electron-withdrawing ability^9–11^. However, the CEC is not determined solely by the electron-transfer ability; it also depends on the effectiveness of the catalyst-water interfacial contact. The strong hydrophobicity associated with fluorinated surfaces may reduce the probability of water approaching the catalyst, thereby limiting productive interfacial electron transfer. Although this limitation can be partly masked under intense ultrasonic excitation, it is expected to become much more important under weak-force conditions, where interfacial contact is inherently less intense. Therefore, maximizing fluorination may not be the optimal design strategy for CEC under realistic hydrodynamic environments. Instead, regulating catalyst wettability to balance the water approach and water removal may offer a more effective route to improve CEC performance.

Natural minerals provide an attractive platform for testing this idea because they are abundant, environmentally compatible, and widely involved in surface-mediated redox reactions^12,13^, ROS generation^14–17^, contaminant transformation, and biogeochemical cycling in natural systems^18–23^. Silicate minerals are particularly suitable because they constitute more than 90% of the Earth’s crust and possess abundant surface hydroxyl groups that can be chemically tuned to regulate hydrophilicity and hydrophobicity. Moreover, natural hydrodynamic settings such as rivers, waterfalls, rainfall, and tidal flows provide continuous low-grade mechanical energy^24–26^, which may drive mineral-water interfacial CEC over large spatial and temporal scales. However, whether mineral-based CEC can operate effectively under natural weak-force conditions and how interfacial wettability governs this process remain largely unknown.

Here, we develop a hydrophobicity-regulation strategy for CEC on natural silicate minerals to optimize interfacial water transfer. As proof of this strategy, alkyl-functionalized minerals are used to construct a non-fluorinated catalyst (M-C) with moderate hydrophobicity, which outperforms both the hydrophilic raw mineral (M) and the more hydrophobic fluorinated mineral (M-F). Combining experiments under ultrasonication, circulating water flow, and natural river conditions with a theoretical analysis based on extended Lennard-Jones (L-J) interactions and density functional theory (DFT), we show that moderate hydrophobicity enhances the CEC by balancing the water approach and water removal at the interface. We further demonstrate that this strategy is effective under weak-force and natural hydrodynamic conditions, where it reshapes interfacial chemistry and surface-associated microbial communities. These findings support hydrophobicity regulation as a contact-optimization strategy for CEC in the tested silicate mineral systems and suggest that balanced wettability may be relevant to environmentally occurring mineral-water interfaces under weak hydrodynamic excitation.

## Results

### CEC performances of minerals with hydrophilic and hydrophobic regulation

**Fig. 1:**
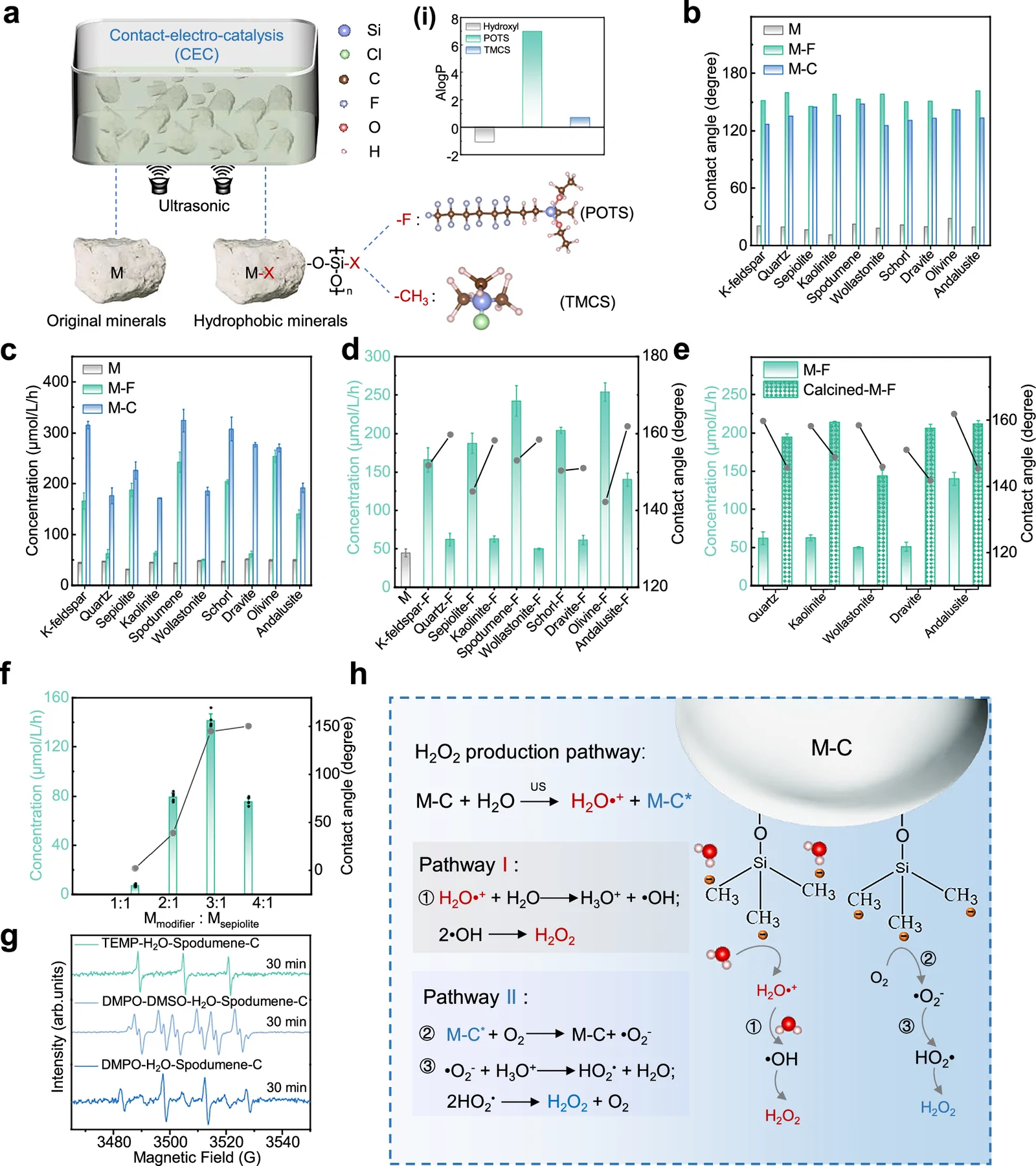
Hydrophobic modification of minerals and their CEC performances. **a** Schematic diagram of the modification strategy and ultrasonic-assisted CEC device; the fluorination modifier is 1H,1H,2H,2H-perfluorodecyltriethoxysilane (POTS) and the alkylation modifier is trimethylchlorosilane (TMCS). (illustration: AlogP values of different hydrophobic/hydrophilic molecules). **b** Contact angles of three types of minerals with different hydrophilicity/hydrophobicity. **c** H_2_O_2_ production of three types of minerals with different hydrophilicity/hydrophobicity through CEC. Error bars represent standard deviation based on ten independent replicates. **d** H_2_O_2_ production and contact angles of fluorinated modified minerals (M-F). Error bars represent standard deviation based on ten independent replicates. **e** H_2_O_2_ production and contact angles of calcined then fluorinated silicate minerals. Error bars represent standard deviation based on ten independent replicates. **f** Influence of the mass ratio of modifiers and sepiolite on the CEC performance and the contact angle of sepiolite-F. Error bars represent standard deviation based on five independent replicates. **g** Electron paramagnetic resonance spectra of spodumene-C under ultrasonication for 30 min. **h** Schematic illustration of reactive oxygen species (ROS) generation on alkylated modified minerals (M-C) via interfacial electron transfer between H_2_O and alkyl groups, proceeding through water oxidation (Pathway Ⅰ) and oxygen reduction (Pathway Ⅱ). Source data are provided as a Source Data file.

Based on the results of preliminary experiments, the CEC activities of natural silicate minerals (M), which can be classified into five categories—tectosilicates (quartz and K-feldspar), phyllosilicates (sepiolite and kaolinite), inosilicates (spodumene and wollastonite), cyclosilicates (schorl and dravite), and nesosilicates (olivine and andalusite)—were assessed (Supplementary Fig. 1). These five categories were selected according to the classical structural classification of silicate minerals based on the polymerization mode of silicon-oxygen tetrahedra, thereby covering the major structural motifs from isolated tetrahedra to three-dimensional frameworks and providing a representative mineral set for evaluating the general applicability of the hydrophobicity-regulation strategy. Their CEC performances were found to be limited. To examine whether the hydrophobic modification strategy is broadly applicable across the tested silicate minerals, two hydrophobic modifiers, 1H, 1H, 2H, 2H-perfluoroalkyltriethoxysilanes (POTS) and trimethylchlorosilane (TMCS), were applied to modify the surfaces of the ten mineral types individually to achieve different hydrophobicities (Supplementary Fig. 2). Unmodified minerals (M) and modified variants (M-F and M-C) were subsequently used as CEC catalysts under ultrasonic conditions to investigate their respective CEC performances (Fig. 1a).

The AlogP values of the functional groups and modifier molecules were calculated to evaluate the hydrophilicity/hydrophobicity of the minerals^27,28^ (Fig. 1a (i) and Supplementary Note 1). TMCS and POTS have positive AlogP values, indicating their hydrophobic nature, whereas the AlogP value of the hydroxyl group is negative. Moreover, the contact angles of both M-C and M-F exceed 125°, confirming their hydrophobicity, whereas those of M are less than 30°, indicating hydrophilicity (Fig. 1b). These results indicate that -CH_3_ and -F surface modifications can broadly impart hydrophobicity to the tested silicate minerals under the present modification conditions. In addition, all the minerals have negative zeta potentials (−3.0 to −35.2 mV; Supplementary Fig. 3), among which M-F has the highest negative potential (−6.9 to −35.2 mV), and M-C has the lowest negative potential (−3.0 to −23.7 mV).

Mineral CEC performance was assessed by monitoring H_2_O_2_ production under ultrasonic irradiation (40 kHz, 300 W; Fig. 1c). H_2_O_2_ generation was significantly greater for M-C and M-F than for M. The highest production rates were obtained for spodumene-C (350.0 μmol·L^–1^·h^–1^), olivine-F (270.6 μmol·L^–1^·h^–1^), and dravite (51.2 μmol·L^–1^·h^–1^). Both M-F and M-C exhibit high H_2_O_2_ generation, verifying the beneficial effect of hydrophobicity. Furthermore, M-C provides a new opportunity to reduce the dependence of CEC catalysts on -F groups. These findings suggest that hydrophobic modification plays a crucial role in governing CEC performance.

Although M-C is less hydrophobic than M-F is, it consistently has higher CEC performance than M-F does, indicating that excessively high hydrophobicity is unfavorable for CEC (Fig. 1b, c). To further isolate the effect of contact properties while minimizing the influence of differences in electron-withdrawing groups, we used M-F as a comparable platform to study the regulation of hydrophobicity, because the samples exhibit the most different contact angles before and after modification. Among mineral pairs across five structural classes—tectosilicates, phyllosilicates, inosilicates, cyclosilicates, and nesosilicates—those with moderate hydrophobicity consistently presented higher CEC performance (Fig. 1d). For the five minerals with the highest contact angles and lowest CEC activity, optimization by calcination or plasma treatment before fluorination reduced the contact angle and enhanced the CEC performance in all the cases (Fig. 1e, Supplementary Figs. 4 and 5), further demonstrating that moderate contact with water is a key factor controlling the CEC.

To better understand why the M-F samples still exhibited different wettability and CEC performances, we further analyzed their surface composition by XPS (Supplementary Fig. 6). The results revealed that the relative oxygen content of the surface was closely associated with the final wettability and CEC activity of the minerals: samples with a higher relative oxygen content on the surface generally exhibited lower contact angles and higher CEC performance, and the same trends were observed for the calcined then fluorinated minerals and the plasma-treated then fluorinated minerals (Supplementary Fig. 7). These results suggest that although fluorination reached a consistently high level under the present modification conditions, complete substitution of all surface oxygen-containing sites was still limited by the steric hindrance of the carbon-fluorine chains of POTS. As a result, differences in the remaining polar surface sites led to subtle but important variations in wettability, which further affected the effective mineral-water contact during CEC (Supplementary Fig. 8). This interpretation is also supported by the modifier dosage experiment, in which excessive fluorination caused a marked decrease in catalytic activity (Fig. 1f), indicating that overly strong hydrophobicity suppresses effective mineral-water contact (Supplementary Figs. 9, 10 and Supplementary Note 2). Therefore, for M-F, the final surface wettability is a more relevant descriptor of CEC performance than the fluorination treatment alone, and a moderately hydrophobic surface is more favorable for efficient CEC.

The free radicals and intermediates produced during the M-C CEC process were identified using electron paramagnetic resonance (EPR). Hydroxyl radicals (•OH), superoxide radicals (•O_2_^–^), and singlet oxygen (^1^O_2_), which is the product of •O_2_^–^, were detected under ultrasonic irradiation (Fig. 1g and Supplementary Fig. 11). To further confirm the CEC pathway of M-C, after replacing the H_2_O in the benzoic acid aqueous solution with H_2_^18^O, the ^18^O isotope labeling experiment was conducted in combination with liquid chromatography-mass spectrometry (LC-MS) to analyze the source of O in •OH. The ^18^O-incorporated species were identified via the total ion chromatogram and mass spectra, demonstrating that •OH originates from H_2_O, that is, the water oxidation reaction (WOR) of CEC (Supplementary Fig. 12a, b)^29^. WOR occurs directly during the CEC electron transfer process and generally plays a dominant role in the occurrence of CEC reactions and the amount of ROS produced. The detected •O_2_^–^ is the result of the reduction of O_2_ by electrons on the surface of M-C, namely, the oxygen reduction reaction (ORR), which is susceptible to mechanical energy and oxygen conditions. In addition, XPS analysis shows only negligible Fe signals in the tested minerals, suggesting that Fe-mediated Fenton-like reactions are unlikely to substantially contribute to ROS generation or affect the evaluation of CEC performance (Supplementary Fig. 13). Thus, the following pathway for H_2_O_2_ production is proposed (Fig. 1h): The collapse of ultrasonic cavitation bubbles facilitates electron transfer between H_2_O molecules and M-C, triggering H_2_O oxidation to generate •OH (➀), which subsequently forms H_2_O_2_. Moreover, the electrons acquired by M-C react with O_2_ to generate •O_2_^–^ (➁), which then undergoes superoxide dismutation to yield H_2_O_2_ (➂).

### Interface contact mechanism governed by catalyst hydrophobicity

**Fig. 2:**
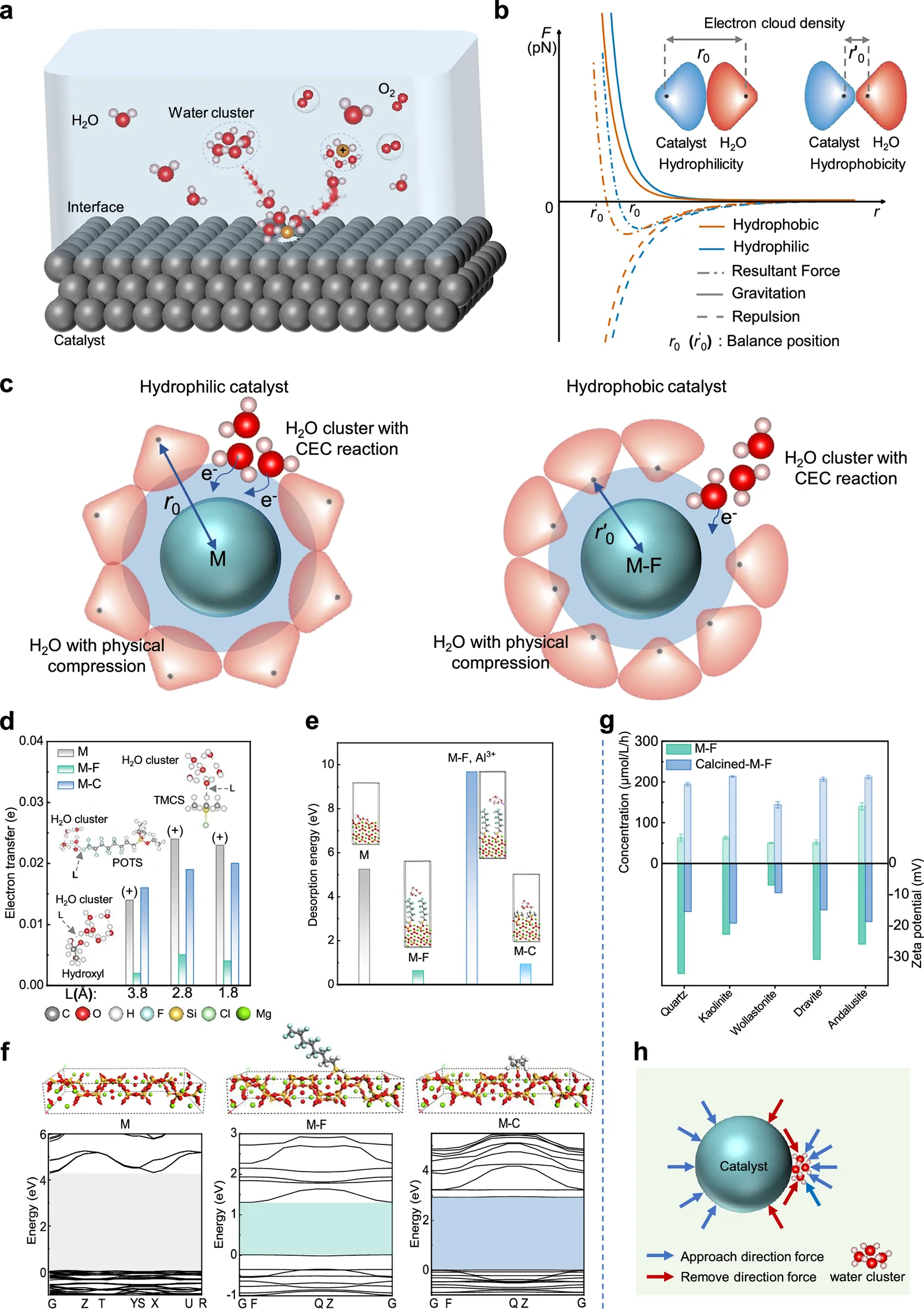
Theoretical analysis of the influence of catalyst hydrophilicity/hydrophobicity on water migration. **a** A schematic illustration of the contact–separation process between water clusters and the catalyst surface to achieve electron transfer. **b** Extended Lennard-Jones potential curve; *F* denotes the intermolecular force. (illustration: schematic illustration of electron cloud distribution of water and catalyst with hydrophilicity or hydrophobicity). **c** Interfacial contact analysis of hydrophilic and hydrophobic catalysts with water. The blue shaded ring represents the electron cloud distribution of the catalyst; the red shaded sector represents the electron cloud distribution of water; the straight blue arrow indicates the equilibrium distance between catalyst and water (*r*_0_ and *r*'_0_). **d** Electron transfer between hydrophilic/hydrophobic molecules and a hexameric water cluster as a function of distance based on DFT simulations. **e** The desorption energy of a hexameric water cluster on hydrophilic and hydrophobic mineral surfaces calculated by DFT simulations. **f** Energy band structures of modified minerals obtained by DFT simulations. **g** Influence of the zeta potential on CEC performance. Error bars represent standard deviation based on ten independent replicates. **h** Analysis of the force direction during the removal of water clusters from catalyst particles. Source data are provided as a Source Data file.

The hydrophobic properties of the catalyst strongly influence the mineral CEC, as proven by the preceding experiments. The underlying mechanism is analyzed in detail in this section. When H_2_O molecules and a catalyst contact, they do not result in a 100% CEC; instead, only a small fraction of H_2_O achieves a chemical reaction with concentrated energy or pathways, where the majority of H_2_O molecules merely overcome repulsive forces to convert mechanical energy into heat^30,31^, which represents a physical process (Fig. 2a–c). As a result of hydrogen bonding, H_2_O molecules tend to aggregate into clusters, and most H_2_O molecules act as carriers, transferring reacted H_2_O involved in CEC. Consequently, the migration of H_2_O molecules (physical process) substantially affects their contact with the catalyst surface, thereby influencing the electron-cloud overlap and de-overlap within the CEC (Fig. 2c). In this section, the extended L-J potential formula is employed to study the effect of catalyst hydrophilicity and hydrophobicity on the forces acting on H_2_O molecules to infer the physical factors governing their mobility at catalyst interfaces.

The intermolecular forces between H_2_O molecules and hydrophilic/hydrophobic catalyst molecules were calculated using the extended L-J potential formula^32–35^ (Supplementary Note 3) and are compared in Fig. 2b. These attractive and repulsive forces include dipole-dipole forces, dipole-induced dipole forces, hydrogen bonding, and Coulomb forces (Supplementary Fig. 14). The parameter *r*_*0*_ (*r'*_*0*_) represents the equilibrium distance between H_2_O and the catalyst in the absence of an external force (*r*_*0*_ for hydrophilic molecules and *r'*_*0*_ for hydrophobic molecules). When the molecular distance is less than *r*_*0*_(*r'*_*0*_), an external force is required to overcome the strong repulsive force arising from the electron-cloud overlap, as described by the Pauli exclusion principle. Increasing the catalyst hydrophilicity enhances the attractive interactions (e.g., dipole-dipole forces and hydrogen bonding), resulting in *r*_*0*_ > *r'*_*0*_. Thus, a hydrophilic catalyst with a larger *r*_*0*_ is more likely to approach H_2_O molecules and achieve electron-cloud overlap during CEC, whereas a hydrophobic catalyst shows the opposite trend (Fig. 2c).

DFT calculations were performed to further clarify how interfacial contact affects CEC performance by analyzing electron transfer (Fig. 2d), desorption energy (Fig. 2e), and energy band structures (Fig. 2f). The atomic coordinates of the optimized computational models are provided in Supplementary Data 1–10. For all three samples, the amount of electron transfer increased as the contact distance with water decreased. M acted as an electron donor to water, in contrast to M-C and M-F, which were electron acceptors. Despite M having the greatest electron transfer, its high desorption energy (Fig. 2e, 5.25 eV) limited the completion of the CEC process, resulting in poor performance. In contrast, both M-C and M-F exhibited similarly low desorption energies, allowing easy detachment of water molecules to complete the CEC (Fig. 2e). Moreover, compared with M-F, M-C showed better electron transfer (Fig. 2d), which can be attributed to its favorable wettability, facilitating better contact with water (Fig. 2c) despite its weaker electron-withdrawing ability (Fig. 2f). This favorable balance between water approach and removal gave M-C a performance advantage over M-F. In addition, the contact position between the modifiers and the water cluster was also regulated (Supplementary Fig. 15), and the interaction between a single water molecule and the samples was further calculated (Supplementary Figs. 16 and 17), both of which supported the positive role of moderately hydrophobic regulation in enhancing CEC performance.

Moreover, exchangeable cations in minerals and aqueous environments may influence the effective interfacial wettability of M-F under practical conditions. Because M-F has a more negative zeta potential (–6.9 to –35.2 mV) than M-C does (–3.0 to –23.7 mV), it is more susceptible to the adsorption of metal ions in water^36–38^, including mineral-derived ions and environmentally relevant cations such as Al^3+^, Ca^2+^ and Mg^2+^. Such ion adsorption may shift the catalyst toward a more hydrophilic state in aqueous media, rather than the hydrophobic behavior suggested by air-based contact angle measurements. Consistently, the results of DFT calculations indicate that cation-associated conditions can increase the water cluster desorption energy (9.69 eV in Fig. 2e), implying that the aqueous environment may alter the apparent interfacial behavior of M-F and thereby partly suppress its CEC performance (Fig. 2g). In contrast, M-C, with its less negative surface potential, is expected to be less affected by such ion-induced perturbation. We therefore consider cation adsorption here as an auxiliary factor that may modify the effective hydrophobicity/hydrophilicity of the catalyst in water, which may further contribute to the better CEC performance of M-C relative to M-F under practical conditions. A systematic analysis of ion-specific adsorption or electric double layer effects, however, is beyond the scope of the present study. Moreover, although dissolved O_2_ participate in the ORR, we focused only on catalyst-water interactions, as water is the main medium directly involved in mechanical contact and the most fundamental factor governing CEC performance^39,40^.

Overall, compared with M and M-F, the moderately hydrophobic catalyst M-C, which provides an optimal balance between the water cluster approach and the removal process, has the best CEC performance. Because mechanical forces applied directly in the separation direction are less effective for small water clusters (Fig. 2h), hydrophobicity was assumed to be a priority factor rather than hydrophilicity, especially under weak-force conditions. Thus, the following section further explores the applicability of this optimization strategy under weak-force contact conditions and in natural environmental settings.

### CEC under weak-force contact conditions and its impact on microbial metabolism in a natural river

**Fig. 3:**
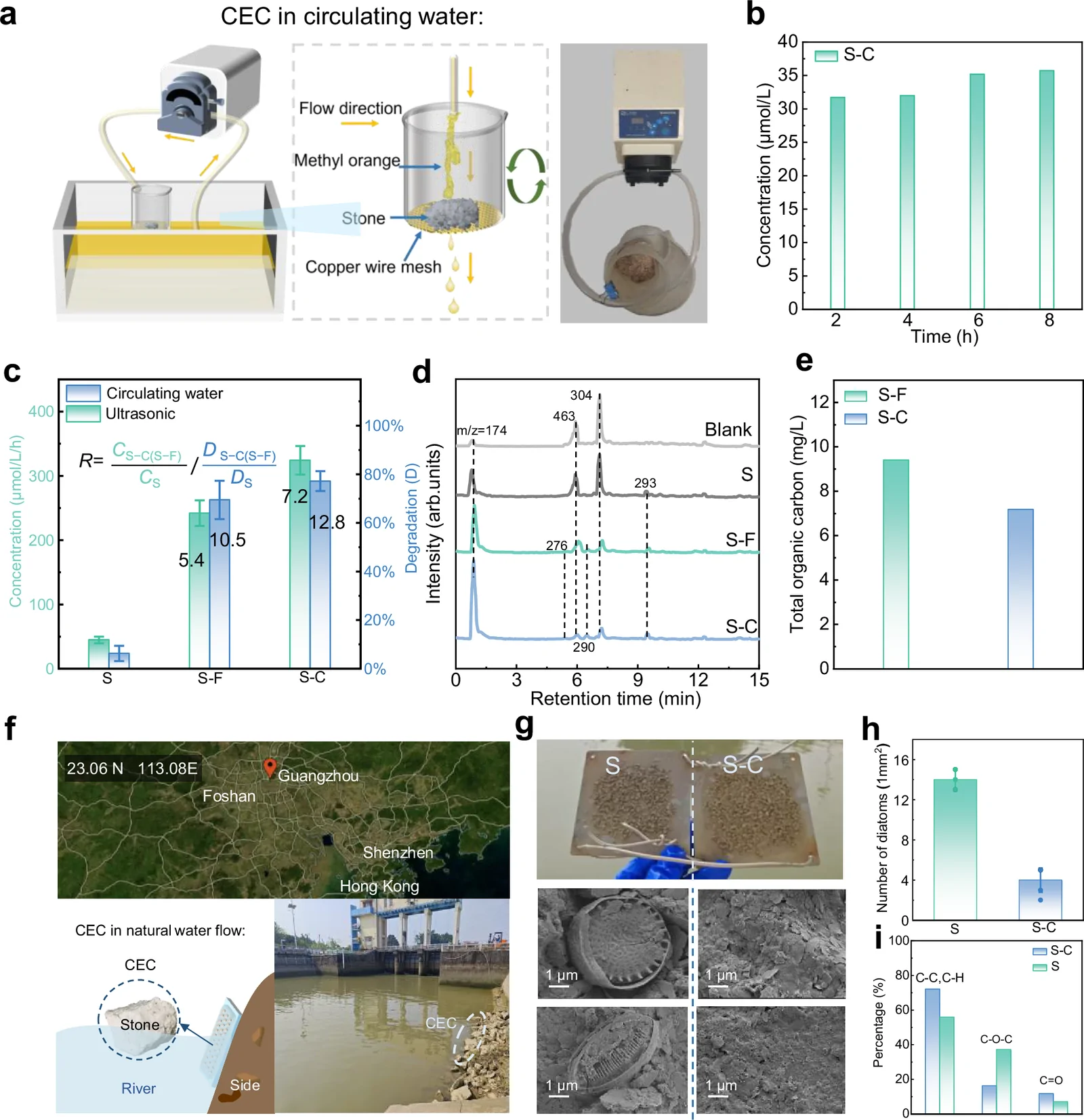
CEC of the mineral catalyst under weak-force excitation. **a** Device used for achieving CEC through circulating water flow. **b** H_2_O_2_ production through CEC under circulating water flow. **c** Comparison of CEC performance and *R* (the fold improvement in CEC performance) under different mechanical forces, including ultrasonication and circulating water flow; *C* represents concentration, and *D* represents degradation efficiency. Error bars represent standard deviation based on ten independent replicates. **d** Mass spectra of the methyl orange (MO) solution after separation by liquid chromatography. **e** Comparison of total organic carbon (TOC) between fluorinated modified stone (S-F) and alkylated modified stone (S-C). **f** The coordinate position of the CEC in the tributary of the Pearl River (top); diagrammatic sketch (left) and realistic scene (right) of CEC by the mineral catalysts in the river. Image captured from NASA Worldview (https://worldview.earthdata.nasa.gov/). **g** Optical photographs (top) of original stone (S) and S-C, and SEM images of diatom frustules on the surfaces of S and S‑C. The SEM experiment was repeated five times, yielding similar results. **h** Comparison of the number of diatoms on the surfaces of S and S-C per unit area. Error bars represent standard deviation based on five independent replicates. **i** Relative intensities of peaks in the XPS C spectra of S and S-C after one month in the river. Source data are provided as a Source Data file.

To evaluate the theoretical speculation that hydrophobicity is advantageous under weak-force contact conditions and to examine the feasibility of self-powered CEC in natural environments, natural stones composed primarily of SiO_2_ were used to prepare three types of catalysts (Supplementary Note 4): original stone (S), stone modified by the -F group (S-F), and stone modified by the -CH_3_ group (S-C). Their catalytic activities were assessed in a circulating water-flow system operating at 0.62 m/s as well as in a natural river environment with a maximum flow velocity of 0.26 m/s. In the circulation system, the catalysts were subjected to mechanical excitation via gravitational waterfall and flow acceleration from a recirculating pump (Fig. 3a). The power density of the circulating water flow is 0.25 times that of ultrasonication (Supplementary Note 5). Because H_2_O_2_ production under such weak-force conditions needs to accumulate to be detected but is an unstable end product in an open and continuously flowing system for reliable direct quantification (Fig. 3b), the degradation of methyl orange (MO) was employed as a stable indicator to accumulate and reflect the catalytic performance of the CEC over time.

A 400 mL MO solution (5 mg/L), circulated continuously by a pump, was continuously used to wash 50 g of S, S-F, and S-C. After 100 h, the MO degradation efficiency achieved through CEC was 77% for S-C, 69% for S-F, and only 4% for unmodified S (Fig. 3c). MO adsorption is negligible, confirming that catalytic degradation is the primary MO removal mechanism (Supplementary Figs. 18–21 and Supplementary Note 6) and underscoring the potential of these catalysts for attenuating organic contamination. Notably, the performance order under weak-force excitation (S-C > S-F > S) remains consistent with that observed under ultrasonic excitation. To compare the influence of hydrophobicity across different force regimes, a parameter *R* was introduced (Fig. 3c), defined as the fold improvement in CEC performance under ultrasonic (U) and circulating water-flow (C) conditions. For S-C, *R*_U_ = 7.2 and *R*_C_ = 12.8; for S-F, *R*_U_ = 5.4 and *R*_C_ = 10.5, indicating that moderate hydrophobicity plays a more pronounced role under weak mechanical excitation, with alkyl modification imparting greater responsiveness than fluorination does.

Furthermore, liquid chromatography-mass spectrometry (LC-MS) demonstrated that MO undergoes degradation rather than mere adsorption. The parent MO peak (*m*/*z* = 304; retention time 7.13 min) decreased most significantly for S-C, accompanied by a corresponding increase in oxidation products (Fig. 3d and Supplementary Table 1). In addition, to prove the stability of the catalyst modification, the stone catalysts were washed under a circulating water flow (0.62 m/s) for 100 h, and the eluted carbon concentrations of S-F and S-C were 9.4 mg/L and 7.1 mg/L, respectively, as determined by total organic carbon (TOC) analysis (Fig. 3e). The percentage of carbon eluted by S-F and S-C to the total carbon of the catalyst was slightly 0.07% and 0.05%, respectively, which indicates that the catalyst leads to little organic pollution to the environment.

To assess CEC performance under natural conditions, S-C and S were deployed in a tributary of the Pearl River (23.06°N, 113.08°E) for one month, where continuous tidal fluctuations and reservoir discharge generated sustained forces to stimulate CEC (Fig. 3f). Scanning electron microscopy (SEM) imaging revealed extensive diatom colonization on S, whereas S-C exhibited markedly reduced colonization (Fig. 3g, h and Supplementary Fig. 22). X-ray photoelectron spectroscopy (XPS) analysis further revealed that S contains higher proportions of C-O-C (37.2%) and N1*s* (organic nitrogen), indicating greater organic matter accumulation on the unmodified mineral surface (Fig. 3i and Supplementary Fig. 23). This behavior likely resulted from the predominantly hydrophilic nature of dissolved organic matter and diatom frustules in river water, which display high affinity for water and polar surfaces, thereby reducing their adhesion to hydrophobic substrates^41^. These observations support the sustainable application of CEC catalyst without passivation and highlight the antifouling advantage of hydrophobic CEC surfaces for long-term deployment in natural waters.

**Fig. 4:**
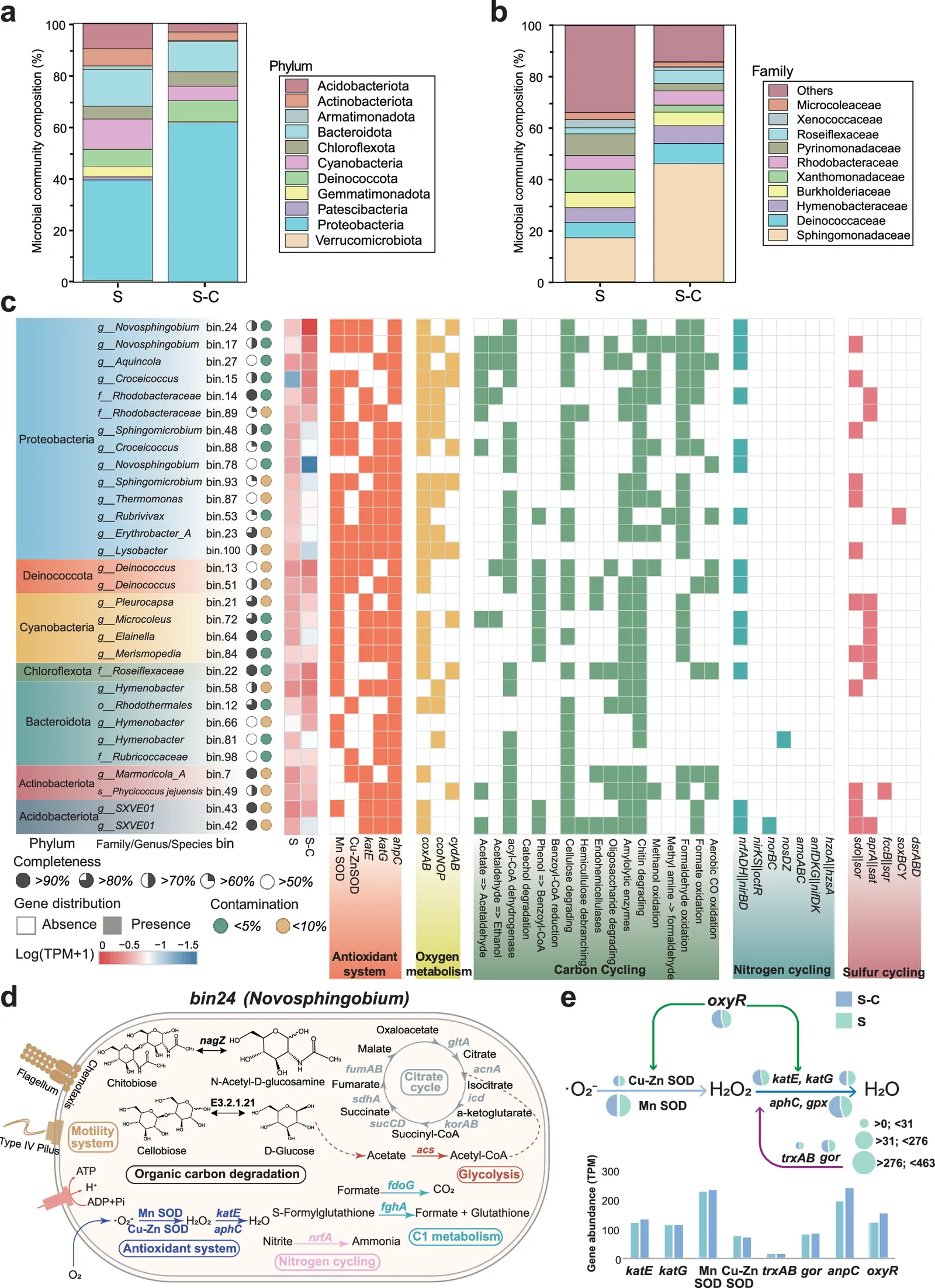
Genome-resolved metagenomic analysis of surface-attached microbial communities. **a** Microbial community composition at the phylum level. **b** Microbial community compositions at the family level. **c** Metabolic profiles of the 30 most abundant metagenome-assembled genomes (MAGs), including genes involved in antioxidant defense, oxygen metabolism, and carbon, nitrogen, and sulfur cycling. The presence of genes within each MAG is indicated by colored shading, and the degree of genome completeness, degree of contamination, and relative abundance (genome copies per million reads, similar to transcripts per million, TPM) are also provided. **d** Draft genome and key metabolic features of the most responsive MAG (*Novosphingobium* sp., bin 24), whose abundance increased ~30-fold from the original stone (S) to alkylated modified stones (S-C). **e** Relative abundance (in TPM) of pathways associated with oxidative stress regulation in the S and S-C samples. Pie charts represent the proportional abundance of genes within each pathway, with chart size scaled to the total TPM per sample. Source data are provided as a Source Data file.

An intriguing and relatively unexplored aspect of CEC is its impact on microbial community composition. To assess the influence of mineral-based CEC on surface-associated microbial communities, a genome-resolved metagenomic analysis was performed (Fig. 4). Following quality control and assembly, scaffolds ≥2000 bp were subjected to binning, resulting in 94 high- and medium-quality metagenome-assembled genomes (MAGs).

Notably, surface modification leads to a pronounced enrichment of Proteobacteria on mineral surfaces, accompanied by reductions in Actinobacteriota, Acidobacteriota, Cyanobacteria, and Gemmatimonadota, while other phyla remain relatively stable (Fig. 4a), suggesting that mineral surface chemistry acts as an ecological filter that shapes microbial attachment and persistence. At the MAG level, members of the aerobic, gram-negative genus *Novosphingobium* (particularly bin. 24 and bin. 17) within Sphingomonadaceae (Fig. 4b), along with *Aquincola* and *Croceicoccus*, are enriched on the modified surfaces (Fig. 4c), indicating selective enrichment on the modified mineral surface. These taxa exhibit broad metabolic versatility, including complete ROS detoxification systems and pathways for degrading diverse organic substrates (Fig. 4c). Compared with MAGs whose abundance decreases (e.g., bins 48, 88, 78, 93, 87, 53, and 64), the enriched MAGs encode genes involved in the utilization of low-molecular-weight compounds such as formate, acetate, acetaldehyde, and oligosaccharides, in addition to those targeting complex polymers such as cellulose and chitin (Fig. 4c, d). This pattern suggests a selective advantage for organisms capable of exploiting oxidized organic substrates. Although ROS-scavenging genes were broadly distributed across the microbial communities (Fig. 4c), the modified mineral sample (S-C) presented a relatively higher relative abundance of ROS-related genes (Fig. 4e), likely reflecting microbial resilience to oxidative stress^42^.

Moreover, genes related to motility, surface sensing, and colonization, such as flagellar assembly genes (e.g., *fli*, *flg*), pilus-associated genes (e.g., *pil*), adhesion-related proteins (e.g., *lap*), and chemotaxis genes (e.g., *che*), were broadly distributed across many MAGs recovered from both treatments, suggesting that the genetic potential for attachment and surface colonization is a common feature of the surface-associated community. Previous studies have shown that hydrophobic surfaces can potentially promote the attachment of microorganisms with higher cell-surface hydrophobicity and stronger biofilm-forming capacity, although microbial adhesion is also strongly modulated by surface charge, roughness, hydrodynamic conditions, and medium chemistry^43–45^. Taken together, these results support a multifactorial restructuring mechanism in which hydrophobic surface selection, altered organic matter adhesion, and potential oxidative pressure from ROS collectively select for microorganisms that are both capable of metabolizing the newly generated organic substrates and tolerant to oxidative stress. It should also be noted that the present metagenomic data cannot disentangle the relative contributions of hydrophobic surface selection, organic matter adhesion, and ROS-mediated oxidative pressure.

To date, demonstrations of CEC under natural weak-force contact conditions have remained limited. The present study is among the first to evaluate the CEC under authentic weak-force contact conditions by deploying -CH_3_-modified silicate catalysts in a natural river environment. These findings demonstrate that ambient water flow and mild turbulence can initiate CEC reactions on mineral substrates, providing proof-of-concept that it can function under open-air environmental conditions. This extends prior laboratory-based studies, which have relied on controlled agitation to drive electron transfer. The capacity of these durable mineral catalysts to sustain contact electrification and ROS production under gentle, continuous flow highlights the potential for CEC-based remediation systems to be powered entirely by natural hydrodynamic energy. Furthermore, such processes may be relevant to a broader range of low-energy aquatic systems, including groundwater, wetland, estuaries, and coastal zones, although this possibility requires validation under additional mineral compositions, water chemistries, biological settings, and hydrodynamic regimes. This process may indirectly influence localized biogeochemical transformations by modulating interfacial chemistry and surface-associated microbial communities. In addition, variations in mineral surface properties and oxidative conditions may provide an additional pathway for influencing organic matter turnover by altering the adsorption and activity of extracellular enzymes involved in decomposition^46,47^.

Despite the promising demonstration that moderately hydrophobic mineral catalysts can sustain contact electrification and ROS production under natural weak-force conditions, several limitations warrant consideration. First, environmental factors such as pH, salinity, and dissolved gases may influence CEC efficiency, potentially limiting its applicability in diverse aquatic systems. Sustained ROS generation may also pose ecological risks by inducing oxidative stress in sensitive organisms and disrupting native microbial communities^42,46,48^. Under the tested conditions, our results did not indicate broad community-wide suppression, although the negative effects on specific sensitive taxa (e.g., those with weaker antioxidant defense systems or lower tolerance to hydrophobic surface conditions) cannot be excluded. In addition, because the grafted functional groups are covalently bound to the mineral surface, leaching-related effects are expected to be limited under the tested conditions, but this aspect should be explicitly evaluated in future work. Moreover, hydrophobic mineral particles may aggregate in water, reducing the effective contact area and limiting long-term catalytic performance. Finally, the introduction of modified catalysts into natural environments may unintentionally alter sediment properties or react with organic matter in unanticipated ways, and the long-term ecological impacts of such deployments remain unclear. Future work should focus on optimizing catalyst dispersion, regulating ROS output, and conducting comprehensive environmental risk assessments prior to large-scale field application.

## Discussion

This work demonstrates that hydrophobicity regulation can serve as a contact-optimization strategy for contact-electro-catalysis (CEC) at the tested silicate mineral-water interfaces. Rather than simply pursuing stronger electron-withdrawing ability, our results show that efficient silicate mineral-based CEC depends on balancing interfacial wettability to coordinate water approach and removal during contact. As a material demonstration of this strategy, the moderately hydrophobic alkyl-modified mineral catalyst (M-C) outperformed both the hydrophilic raw mineral (M) and the more hydrophobic fluorinated counterpart (M-F), highlighting that moderate hydrophobicity is more favorable for CEC than either insufficient or excessive water affinity. Importantly, this advantage became even more pronounced under weak-force conditions, where excessive hydrophobicity more severely limits effective water-catalyst contact and makes balanced interfacial transfer especially critical.

Beyond catalyst design, this strategy remained effective under environmentally relevant conditions. The operation of alkyl-modified stone under circulating water flow and in a natural river shows that non-fluorinated mineral catalysts can sustain self-powered CEC driven by ambient hydrodynamic energy, extending the CEC beyond that of conventional fluorinated laboratory systems. More broadly, these findings suggest that hydrophobicity-regulated mineral-water interfacial CEC may represent a previously underappreciated pathway under certain mineral-water and weak-flow conditions linking reactive oxygen species generation with surface reactions, organic matter transformation and microbial niche differentiation in natural aquatic environments.

## Methods

### Chemicals

Materials were obtained from commercial suppliers and were utilized without further treatment unless previously stated. Quartz (SiO_2_, from Hebei, China, 99%), K-feldspar (K[AlSi_3_O_8_], from Shanxi, China, ≥95%), sepiolite (Mg_8_(H_2_O)_4_[Si_6_O_15_]_2_(OH)_4_•8H_2_O, Macklin, 99%), kaolinite (Al_4_[Si_4_O_10_](OH)_8_, Macklin, 99%), spodumene (LiAl[Si_2_O_6_], from Shanxi, China, 98%), wollastonite (Ca_3_[Si_3_O_9_], from Hebei, China, 99%), schorl (NaFe_3_Al_6_[Si_6_O_18_][BO_3_]_3_(OH, F)_4_, from Hebei, China, 99%), dravite (NaMg_3_Al_6_[Si_6_O_18_][BO_3_]_3_(OH, F)_4_, from Hebei, China, 98%), olivine ((Mg, Fe)_2_[SiO_4_], from Shanxi, China, 99.5%), andalusite (Al_2_SiO_5_, from Shandong, China, 99%), dimethylacetamide (C₄H₉NO, Innochem, 99.8%), 1H,1H,2H,2H-perfluorooctyltriethoxysilane (C_14_H_19_F_13_O_3_Si, Aladdin, ≥97%), acetic acid (C_2_H_4_O_2_, Sinopharm Chemical Reagent Co. Ltd, 99.7%), sodium acetate (C_2_H_3_NaO_2_, Macklin, 99%), 3,3,5,5-tetramethylbenzidine (C_16_H_20_N_2_, Shanghai Shifeng Biological Technology Co., Ltd, 99%), dimethyl sulfoxide (C_2_H_6_OS, Macklin, 99.9%), horseradish peroxidase (Macklin, RZ > 2), ethyl alcohol (C_2_H_6_O, Tianjin Damao Chemical Reagent Factory, 99.5%), 5,5-dimethyl-1-pyrroline N-oxide (C_6_H_11_NO, Macklin, 97%), 2,2,6,6-tetramethyl-4-piperidone hydrochloride (C_9_H_18_ClNO, Aladdin, ≥98%), n-hexane (C_6_H_14_, Aladdin, 98%), trimethylchlorosilane (C_3_H_9_SiCl, Macklin, 99%), benzoic acid (C_7_H_6_O_2_, Macklin, 99.9%), crystal sand (SiO_2_, from Hebei, China, 95%). Deionized water was used throughout the experiment.

### Sample preparation

Silicate minerals (0.3 g) were added to 10 mL of anhydrous dimethylacetamide (DMAC) to form a mixture. First, 0.9 g of 1H,1H,2H,2H-perfluorooctyltriethoxysilane (POTS) was added to the DMAC suspension, and the mixture was shaken on an oscillator for 5 min to obtain a homogeneous dispersion. The modified silicate mineral was obtained by centrifugation at a centrifugal force of 10732 × *g* for 8 min, after which the supernatant was poured off. The fluorinated silicate minerals were dried in an oven at 75 °C for 2 h. The alkylation modification procedure was identical to the fluorination procedure, except that the DMAC was replaced with n-hexane and the POTS was replaced with trimethylchlorosilane.

For calcination, 2 g of silicate minerals was placed in a 50 mL corundum crucible and heated in a muffle furnace at a specified temperature for 2 h, at a heating rate of 5 °C/min. The specific temperature is 800 °C for quartz, 550 °C for kaolinite, 600 °C for wollastonite, 700 °C for dravite, and 800 °C for andalusite. Calcination-modified silicate minerals were obtained after cooling to 25 °C. Plasma-modified silicate minerals were prepared by placing 0.3 g of the silicate mineral in a plasma processor and subjecting it to three consecutive treatments, each at 500 W for 5 min.

### H_2_O_2_ production quantification

20 mg of the fluorinated modified silicate mineral was added to a 100 mL beaker containing 50 mL of deionized water. The mixture was magnetically stirred at 500 rpm for 48 h and then sonicated in an ultrasonic cleaner at 40 kHz and 300 W for 1 h. An aliquot of 500 μL of the reacted liquid was transferred into 1.8 mL of acetic acid/sodium acetate buffer (pH 3.6), followed by the addition of 100 μL of 10 mg/mL 3,3′,5,5′-tetramethylbenzidine (TMB) in dimethyl sulfoxide (DMSO) and 20 μL of 0.1 mg/mL horseradish peroxidase aqueous solution. The resulting solution gradually turned blue, and its absorbance was measured using a UV–vis spectrophotometer, which revealed an absorption peak at 655 nm.

### Degradation of MO by circulating water flow

Water flow was simulated using a peristaltic pump to enable the crystal sand to degrade the dye under flow conditions. 400 mL of 5 mg/L methyl orange (MO) solution was added to a 2 L beaker. Under xenon lamp irradiation, 50 g of the modified crystal sand was placed in a funnel positioned above the beaker, allowing the dye to be degraded through the combined action of gravity and flowing water erosion. The xenon lamp was introduced to reduce catalyst poisoning, but did not affect the comparative evaluation of the flow-driven CEC performance (Supplementary Fig. 24 and Supplementary Note 7).

### ROS detection

The parameters for the electron paramagnetic resonance experiments were as follows. Hydroxyl radicals (•OH): First, 10 mg of the silicate mineral catalyst was added to 5 mL of DMPO solution (100 mM). The mixture was mixed and sonicated for 30 min, and aliquots were withdrawn for EPR analysis at 15 and 30 min.

Superoxide radical (•O_2_^−^): First, 10 mg of the silicate mineral catalyst was added to 5 mL of DMPO/DMSO solution (containing 100 mM DMPO and 1 mM DMSO). The mixture was mixed and sonicated for 30 min, and aliquots were taken for EPR measurement at 15 and 30 min.

Singlet oxygen radical (^1^O_2_): First, 10 mg of the silicate mineral catalyst was added to 5 mL of TEMP solution (100 mM). The mixture was mixed and sonicated for 30 min, and aliquots were sampled for EPR analysis at 15 and 30 min.

### Sample characterization

The UV-Vis absorbance was measured using a Shimadzu UV-7600 UV-visible spectrometer over the range of 400–800 nm. Fourier transform infrared spectroscopy (FTIR) was used to characterize the functional groups of the silicate mineral samples over the range of 400–3000 cm^–1^. X-ray diffraction (XRD) patterns were recorded on a Rigaku Ultima (Japan) over a 2θ range of 5° to 90°. The particle size and zeta potential of the silicate minerals were measured using a Malvern Zetasizer Nano ZS90. The contact angle of the silicate minerals was measured using a Krüss instrument (Germany) by the sessile drop method, and the contact angle values were calculated using Young’s equation. The morphology of the silicate minerals was observed by scanning electron microscopy (SEM; TESCAN MIRA LMS). The electronic states of the silicate mineral surfaces were analyzed by X-ray photoelectron spectroscopy (XPS; Thermo Scientific K-Alpha), and all binding energies were calibrated using the C 1 *s* peak at 284.8 eV. The presence of reactive oxygen species (ROS) was detected using an electron paramagnetic resonance (EPR) spectrometer (Bruker EMXnano, Germany). The amounts of polymeric carbon species and fluoride ions that remained in the system after contact-electro-catalysis were measured using a total organic carbon analyzer (XPERT-TOC/TNb, the Netherlands) and an ion-selective electrode (calomel electrode, PHS-3C), respectively.

Modified olivine (0.1 g/L), H_2_^18^O (98 atom %) (1 mL) and 50 mg/L benzoic acid (20 μL) were placed in a 20 mL glass vial. After ultrasonication for 30 min, the resulting mixtures of p-HBA, o-HBA and m-HBA were analyzed using an Ultimate 3000 UHPLC-Q Exactive liquid chromatography-mass spectrometer (Thermo Scientific, US). Chromatographic separation was performed on a Waters Atlantis T3 column (100 mm × 3.0 mm, 3.0 μm). Mobile phase A consisted of 5 mM ammonium formate and 0.1% formic acid in water, and mobile phase B was methanol. The elution program was isocratic: 0–12 min, 60% A and 40% B, at a flow rate of 0.3 mL/min. The injection volume was 10 μL.

Hydroxybenzoic acids with molecular formulae C_7_H_5_^16^O_2_^18^O and C_7_H_5_^16^O_3_ were qualitatively characterized under negative-heating electrospray ionization (HESI-) conditions. The HESI spray voltage was –3.0 kV. The capillary temperature was 320 °C, and the probe heater temperature was 250 °C. The sheath gas, auxiliary gas, and sweep gas were 40 (arbitrary units), 10 (arbitrary units), and 1 (arbitrary units), respectively.

### Experimental operation of placing minerals in the river

The minerals were bonded onto a 10 cm × 10 cm stainless steel plate using acrylic polyurethane paint. Samples S and S-C were each bonded onto separate steel plates and then connected with an iron wire. The stainless steel plate was fixed to a large stone on the shore with an iron wire.

### Metagenomic analysis

DNA was extracted from mineral samples collected after prolonged operations in the river using the E.Z.N.A.® Soil DNA Kit (Omega Bio-tek, Norcross, GA, USA) following the standardized protocol. DNA integrity was evaluated by 1% agarose gel electrophoresis, and DNA concentration and purity were determined using a Qubit 4.0 fluorometer and a NanoDrop One spectrophotometer (Thermo Fisher Scientific, Waltham, MA, USA). Sequencing libraries were prepared from qualified DNA using the ALFA-SEQ DNA Library Prep Kit (Findrop, China) according to the manufacturer’s instructions. Library fragment-size distributions were evaluated using a Qsep400 high-throughput nucleic acid-protein analysis system, and library concentrations were measured using a Qubit 4.0 fluorometer. Paired-end sequencing was performed using a DNBSEQ-T7 (MGI) platform (PE150).

A total of 125.6 GB of raw sequencing reads were assessed, deduplicated and quality-trimmed using FastQC v0.12.1 and fastp v0.23.4^49^, followed by the removal of host-derived sequences using KneadData v0.12.0^50^. All the high-quality datasets were coassembled using MEGAHIT v.1.2.9^51^ with the parameters “-k 21,33,55,77,99 –meta”^51^, and the assembly quality was evaluated with QUAST v5.2.0^52^. The assembly produced 6,704,300 contigs with a GC content of 61.96% and an N50 of 1101 bp. Genes were predicted from contigs ≥500 bp with Prokka v1.14.6^53^, and redundant genes were removed using CD-HIT-EST v4.8.1^54^. The predicted nucleotide sequences were translated into protein sequences using EMBOSS transeq v6.6.0^55^, and gene abundance was quantified with Salmon v1.10.2^56^. The functional annotation of protein sequences was performed using eggNOG-mapper v2.1.13^57^ to obtain Gene Ontology (GO), Kyoto Encyclopedia of Genes and Genomes (KEGG), and Cluster of Orthologous Groups (COG) classifications as previously described^58^.

To comprehensively characterize the functional metabolism of microorganisms, metagenomic binning was used to obtain the metagenome-assembled genome (MAG) as previously described^59^. Contigs ≥2000 bp were binned using MetaWRAP v1.3(--metabat2 --maxbin2 --concoct)^60^, and the resulting MAGs were dereplicated with dRep v3.4.2^61^. The quality of the resulting MAGs was evaluated using CheckM v1.2.2^62^, and only MAGs with completeness >50% and contamination <10% were retained for downstream analyses. The phylogenetic placement of the MAGs assembled in the present study was identified by GTDB-Tk (v2.3.2) based on 120 bacterial and 53 archaeal single-copy marker genes^63^. The relative abundance of each MAG was calculated using the ‘quant_bins’ module of MetaWRAP v1.3. Finally, genes within the genomes were annotated using METABOLIC v4.0^64^.

### Reporting summary

Further information on research design is available in the Nature Portfolio Reporting Summary linked to this article.

## Supplementary information

Supplementary Information

Description of Additional Supplementary Files

Supplementary Data 1-10

Reporting Summary

Transparent Peer Review file

## Source data

Source Data 1

Source Data 2

## Data Availability

The data supporting the findings of this study are reported in the main text or the Supplementary Information. The metagenomic sequencing reads have been deposited in the Genome Sequence Archive (GSA) under accession code CRA035050. The MAGs reconstructed have been deposited in Zenodo (https://doi.org/10.5281/zenodo.21494184). Source data are provided with this paper.
