# Supplementary material for: Hydrophobicity strategy drives contact-electro-catalysis on natural silicate minerals
Source: Nat Commun. 2026 Aug 29;17:10303. doi: 10.1038/s41467-026-77246-0 (PMC13620137; doi:10.1038/s41467-026-77246-0)
Supplement: Supplementary file 1 [file 41467_2026_77246_MOESM1_ESM.pdf]

Supplementary Information

Hydrophobicity Strategy Drives Contact-Electro-Catalysis on Natural Silicate Minerals

Xinnan Li[1], Wangshu Tong[1,2,3], Shuchen Tu[4], Tianyang Hao[4], Xuxu Zhang[1], Tingting Cao[1], Yong Li[5], Qi An[1,2,3]*, Yizhi Sheng[6]**

[1]School of Materials Science and Technology, China University of Geosciences (Beijing), 100083, China.

[2]Beijing Key Laboratory of Materials Utilization of Nonmetallic Minerals and Solid Wastes, China University of Geosciences (Beijing), 100083, China.

[3]Engineering Research Center of Ministry of Education for Geological Carbon Storage and Low Carbon Utilization of Resources, China University of Geosciences (Beijing), 100083, China.

[4]School of Environment, South China Normal University Guangzhou, 510006, China.

[5]College of Geoscience and Surveying Engineering, China University of Mining and Technology (Beijing), 100083, China.

[6]State Key Laboratory of Geomicrobiology and Environmental Changes, China University of Geosciences (Beijing), 100083, China.

*Correspondence author. E-mail: tong@cugb.edu.cn; an@cugb.edu.cn; shengyz@cugb.edu.cn.

The PDF file includes:

Supplementary Note 1 Density functional theory (DFT) calculation.

Supplementary Note 2 Exploration of factors influencing M–F CEC.

Supplementary Note 3 Extended Lennard-Jones potential formula.

Supplementary Note 4 Modification process of stone.

Supplementary Note 5 Estimation of ultrasonication and circulating water flow energy density.

Supplementary Note 6 Discussion on the adsorption of three dyes by S and S-C.

Supplementary Note 7 Effect of xenon lamp irradiation on flow-driven MO degradation.

Supplementary Fig.1 CEC performances of five categories (including ten types) of natural silicate minerals under ultrasonication.

Supplementary Fig.2 Modification process of silicate minerals.

Supplementary Fig.3 Zeta potentials of three types of minerals with different hydrophilicity/hydrophobicity.

Supplementary Fig.4 XRD patterns of five silicate minerals before and after calcination.

Supplementary Fig.5 H_2O_2 production and contact angles of plasma-treated then fluorinated silicate minerals.

Supplementary Fig.6 Relative fluorine content and relative oxygen content of M-F surfaces measured by XPS.

Supplementary Fig.7 Relative fluorine content and relative oxygen content.

Supplementary Fig.8 Schematic diagram illustrating the contact state between water and the surfaces of oxygen-rich M-F and oxygen-poor M-F after M is modified with sufficient modifier. Oxygen groups are represented by hydroxyl groups.

Supplementary Fig. 9 Schematic diagram illustrating the contact state between the surfaces of

oxygen-rich M-F and water after modification of M with sufficient modifier (left) and excessive modifier (right). Oxygen groups are represented by hydroxyl groups.

Supplementary Fig.10 Influencing factors of CEC and characterization of sepiolite-F.

Supplementary Fig.11 Electron paramagnetic resonance (EPR) spectra of spodumene-C under ultrasonication for 0 min.

Supplementary Fig.12 Liquid chromatography–mass spectrometry (LC–MS) analysis.

Supplementary Fig. 13 The Fe 2*p* XPS spectra of original silicate minerals and crystal sand.

Supplementary Fig.14 Types of interactions between H_2O molecules and catalyst molecules.

Supplementary Fig.15 Electron transfer between hydrophobic molecules and a hexameric water cluster as a function of configurations of hydrophobic molecules based on DFT simulations.

Supplementary Fig.16 Electron transfer between hydrophilic/hydrophobic molecules and a single H_2O molecule as a function of distances based on DFT simulations.

Supplementary Fig.17 Desorption energy of a single H_2O molecule on hydrophilic and hydrophobic mineral surfaces calculated by DFT simulations.

Supplementary Fig.18 Adsorption of MO by S, S-F, and S-C, and the corresponding images after adsorption.

Supplementary Fig.19 Performance of S–F in degrading three cationic dyes.

Supplementary Fig.20 Comparison of adsorption and degradation of MB, RhB, and CV by S and S-C.

Supplementary Fig.21 Comparison of images after adsorption of the three dyes by S and S-C.

Supplementary Fig.22 SEM images of S and S–C.

Supplementary Fig.23 XPS C, N, and P spectra and total elemental spectra of S and S–C.

Supplementary Fig.24 The control experiment with xenon lamp irradiation.

Supplementary Tab.1 Chemical structures of products formed during CEC degradation of MO and identified by mass spectrometry.

Supplementary Note 1 Density functional theory (DFT) calculation.

In this study, DFT calculations were performed using the QSAR, DMol^3, and CASTEP codes[1-3]. The QSAR module's thermodynamic category, using its fast descriptor model and engine, was employed to calculate AlogP. In DMol^3, the exchange–correlation interaction[4] was treated using the generalized gradient approximation (GGA) with the Perdew–Burke–Ernzerhof functional. An all-electron treatment was utilized for the core treatment. The double numerical quality (DNP)[5] basis (4.4) set was utilized for all geometric optimizations and total-energy calculations. Core electrons were modeled using effective core pseudopotentials developed by Dolg[6] and Bergner[7]. In CASTEP, the "ultrasoft" pseudopotential type[8] was selected. The positions of all atoms were fully relaxed until the following convergence criteria were met: 0.001 Ha/Å for force, 10^{-5} Ha for total energy, and 0.002 Å for displacement. Meanwhile, the DFT-D method was used to describe the van der Waals interaction. The desorption energy (E_{des}) of the $6H_2O$ cluster on the material surface is defined as:

$$E_{des} = E(sub) + E(6H_2O) - E(sub + 6H_2O) \quad (1)$$

where $E(sub + 6H_2O)$, $E(sub)$, and $E(6H_2O)$ are the total energies of the substrate with the adsorbed $6H_2O$, the pristine substrate, and the $6H_2O$ cluster, respectively.

Supplementary Note 2 Exploration of factors influencing M-F CEC

For fluorinated sepiolite (POTS:sepiolite mass ratio = 3:1), the colorimetric reaction of 20 mg sepiolite-F in 50 mL of H_2O under ultrasonication (40 kHz, 240 W) for 3 h is shown in Supplementary Fig. 10a. As irradiation time increased, a more intense blue coloration was observed, indicating a higher H_2O_2 concentration. Optimization experiments (Supplementary Fig. 10b-d) revealed that the H_2O_2 yield peaked at a catalyst loading of 20 mg/50 mL (Supplementary Fig. 10b). This is attributed to catalyst agglomeration at higher concentrations, which reduces solid-liquid interfacial area and impedes ultrasound propagation, and is consistent with prior CEC studies[9, 10].

To investigate the effect of varying POTS proportions on the modification process and CEC performance, the physicochemical properties of sepiolite modified with different POTS ratios were characterized. Based on the XRD patterns (Supplementary Fig. 10c), no new phases were formed after modification compared to the standard card[11]. As the POTS content increased, the XRD patterns showed progressively sharper characteristic peaks, particularly (110), suggesting that POTS carbon-fluorine chain groups were grafted onto sepiolite while modified sepiolite particles were oriented via Coulombic repulsion. However, at a POTS:sepiolite ratio of 4:1, the sepiolite crystalline phase was severely damaged, resulting in reduced crystallinity. This observation was corroborated by Fourier transform infrared (FTIR) spectroscopy (Supplementary Fig. 10d). The underlying mechanism is that excess POTS content destroys sepiolite's layer-chain structure, forming a thick, amorphous POTS overlayer[12].

Supplementary Note 3 Extended Lennard-Jones potential formula

The Lennard-Jones potential is a widely used two-body potential energy model for describing interactions between neutral atoms or molecules[13-15]. Its formula is as follows:

$$V(r) = 4\epsilon[(\frac{\sigma}{r})^{12} - (\frac{\sigma}{r})^{6}], \quad (1)$$

where ϵ represents the depth of the potential well and σ denotes the distance at which the potential energy is zero. The Lennard-Jones potential is primarily applicable to neutral, spherically symmetric particles, such as inert gases (argon and neon, among others) or nonpolar molecules, where London dispersion forces dominate interactions. Consequently, it does not account for orientational forces between polar groups or induced forces between polar and nonpolar groups.

The Stockmayer potential describes interactions between polar molecules by combining the Lennard-Jones potential with dipole–dipole interactions[16, 17]. Its formula is as follows:

$$U(r,\theta_1,\theta_2,\phi) = 4\epsilon[(\frac{\sigma}{r})^{12}-(\frac{\sigma}{r})^{6}] - \frac{\mu_1\mu_2}{4\pi\varepsilon_0 r^3}(2\cos\theta_1\cos\theta_2 - \sin\theta_1\sin\theta_2\cos\phi), \quad (2)$$

where μ_1 and μ_2 represent the magnitudes of the dipole moments of two molecules, ε_0 denotes the vacuum dielectric constant, θ_1 and θ_2 are the angles between the dipole moments and the molecular axes, and ϕ represents the difference in the azimuthal angles of the two dipoles in the plane perpendicular to the axis. The angle-dependent term cos ϕ describes the influence of dipole orientation on the interaction. From the formula, it can be seen that the attractive term of the Stockmayer potential varies as $\frac{1}{r^3}$.

In addition, hydrogen-bond interactions are not included in the formula. The relationship between hydrogen-bond potential energy and distance is described by empirical potential functions or nonbonded interactions in molecular force fields. In addition to the Lennard-Jones potential, the Buckingham potential is commonly used to describe this part of the potential energy[18-20]:

$$V(r) = \mathrm{A}e^{-\mathrm{B}r} - \frac{1}{r^6}. \qquad (3)$$

The attractive term of the hydrogen-bond potential also varies as $\frac{1}{r^6}$. Finally, catalyst surfaces usually adsorb some ions. The ionic–dipole forces between these ions and water molecules can be expressed using the Coulomb potential[21]:

$$U = \frac{qp\cos\theta}{4\pi\varepsilon_0 r^2}, \qquad (4)$$

where q represents the ionic charge, p denotes the dipole moment, θ is the angle between the dipole moment and the line connecting the ion to the dipole center, r represents the distance between the ion and the dipole center, and ε_0 is the vacuum dielectric constant. From the formula, it is evident that the attractive term of the Coulomb potential varies as $\frac{1}{r^2}$.

Therefore, when various forces are present, the potential energy between catalyst molecules in solution and water molecules can be extended from the Lennard-Jones potential. By simplifying constants and focusing on the power-law terms representing repulsive and attractive interactions, the extended Lennard-Jones potential can be expressed as:

$$V(r) = \mathrm{A}\left(\frac{\sigma}{r}\right)^{12} - \mathrm{B}\left(\frac{\sigma}{r}\right)^{6} - \mathrm{C}\left(\frac{\sigma}{r}\right)^{3} - \mathrm{D}\left(\frac{\sigma}{r}\right)^{6} - \mathrm{E}\left(\frac{\sigma}{r}\right)^{2}. \qquad (5)$$

Supplementary Note 4 Modification process of stone

The stone used was crystal sand, primarily composed of silica (SiO_2), with particle sizes ranging from 3 to 5 mm. This material is abundant in fluvial sediments. Prior to modification, the crystal sand was repeatedly washed with deionized water and oven-dried. The modification procedure followed the protocol detailed in Supplementary Fig. 2: 10 g of crystal sand was added to 10 mL of dimethylacetamide (DMAC) or 10 mL of n-hexane. After mixing, 1 g of either the POTS or the TMCS was introduced. The mixture was continuously agitated for uniform dispersion and allowed to react for 12 h under static conditions to ensure complete functionalization. Finally, the modified crystal sand was dried at 70°C for 2 h to remove residual organics.

Supplementary Note 5 Estimation of ultrasonication and circulating water flow energy density

For the ultrasonic cleaning machine, the energy density can be estimated using formula (1).

$$E\ (\mathrm{W/cm^2}) = \frac{P(\mathrm{W})}{A(\mathrm{cm^2})} \qquad (1)$$

E represents the energy density, *P* represents the effective transmission power, and *A* represents the emission area. By measuring the transmitted power (300W) and the emission area (480 cm^2) of the ultrasonic device, the energy density of the ultrasonic cleaning machine was calculated to be 6250 W/m^2.

For the peristaltic pump, the energy density can be estimated using formulas (2-3).

$$P_h\ (\mathrm{W}) = \rho g Q H \qquad (2)$$

$$I(\mathrm{w/cm^2}) = \frac{P_h}{A} \qquad (3)$$

P_h represents hydraulic power, ρ represents the liquid density, *g* is the gravitational acceleration, *Q* represents flow rate, *H* is head, *I* represents energy density, and *A* represents the scouring area. By measuring *Q*= 1000 ml/min, the inner diameter of the outlet *d* = 8 mm, and the scouring area $A \approx 10.6$ cm^2, P_h was calculated to be 1.67 W, and *I* was calculated to be 1571 W/ m^2. The energy density of the peristaltic pump is much lower than that of the ultrasonic cleaning machine, indicating that the circulation flow impact force of the peristaltic pump is relatively weak.”

Supplementary Note 6 Discussion on the adsorption of three dyes by S and S-C

As shown in Supplementary Fig. 20-21, removal of the three cationic dyes by S was almost entirely due to adsorption, which can be attributed to the abundant surface silicon hydroxyl groups (Si–OH) of the unmodified crystal sand (main component: silica). In near-neutral or mildly acidic aqueous solution, partial deprotonation of these hydroxyl groups generates a negatively charged surface[22], facilitating electrostatic adsorption of cationic dyes. Removal of the three dyes by S-C involved both CEC degradation and adsorption. Compared with S, the adsorption capacity of S-C toward the three cationic dyes decreased mainly due to the reduction in surface negative charge after grafting of alkyl groups, which weakened electrostatic attraction. Under these conditions, adsorption was dominated by van der Waals interactions.

Supplementary Note 7 Effect of xenon lamp irradiation on flow-driven MO degradation

The flow-driven methyl orange (MO) degradation experiments were conducted under xenon lamp irradiation. This illumination condition was intentionally introduced based on previous weak-force CEC studies, in which light was suggested to facilitate electron consumption and help restore the catalyst state, thereby reducing catalyst poisoning[23-25]. Nevertheless, because xenon lamp irradiation may introduce direct photolysis or alter radical-generation pathways, additional control experiments were conducted to clarify its role in the flow-driven MO degradation system.

To distinguish flow-driven CEC from light-induced background degradation, control experiments were performed under xenon lamp and dark conditions. These experiments included no-catalyst controls under xenon lamp and dark conditions, catalyst-containing flow experiments under xenon lamp and dark conditions, and quantitative experiments of free radicals under xenon lamp and dark conditions. As shown in Supplementary Fig. 24a, xenon lamp irradiation alone caused approximately 8% MO degradation in the absence of catalyst, whereas MO removal under the dark no-catalyst condition was negligible. This result confirms that xenon lamp irradiation introduced a measurable but limited photolysis background.

Catalyst-containing flow experiments further showed that illumination did not produce an observable enhancement of the flow-driven CEC performance after accounting for the xenon lamp background. The degradation efficiencies obtained under xenon lamp and dark conditions were comparable, and the relative catalytic trend

remained unchanged. Therefore, the xenon lamp background did not affect the comparative evaluation of flow-driven CEC performance among the tested catalyst systems.

Quantitative experiments of free radicals were further conducted to clarify the effect of xenon lamp irradiation on radical-generation pathways. Under xenon lamp irradiation, the $•O_2^-$ signal increased markedly, whereas it was nearly absent under dark conditions. In contrast, the •OH signal remained relatively stable with or without xenon lamp irradiation (Supplementary Fig. 24b). These results indicate that light mainly promotes the oxygen reduction-related pathway, likely by facilitating electron consumption and thereby reducing catalyst poisoning, whereas the dominant CEC pathway remains associated with water oxidation and •OH generation[23-25]. This interpretation is consistent with the stable MO degradation result with or without xenon lamp irradiation and the ^{18}O isotope labeling results showing that •OH originates from water during CEC.

Overall, xenon lamp irradiation introduced a limited photolysis background and enhanced the auxiliary $•O_2^-$ pathway, but it did not govern MO degradation or alter the comparative evaluation of flow-driven CEC performance. The flow-driven degradation behavior is therefore primarily attributed to CEC associated with water oxidation-derived •OH generation.

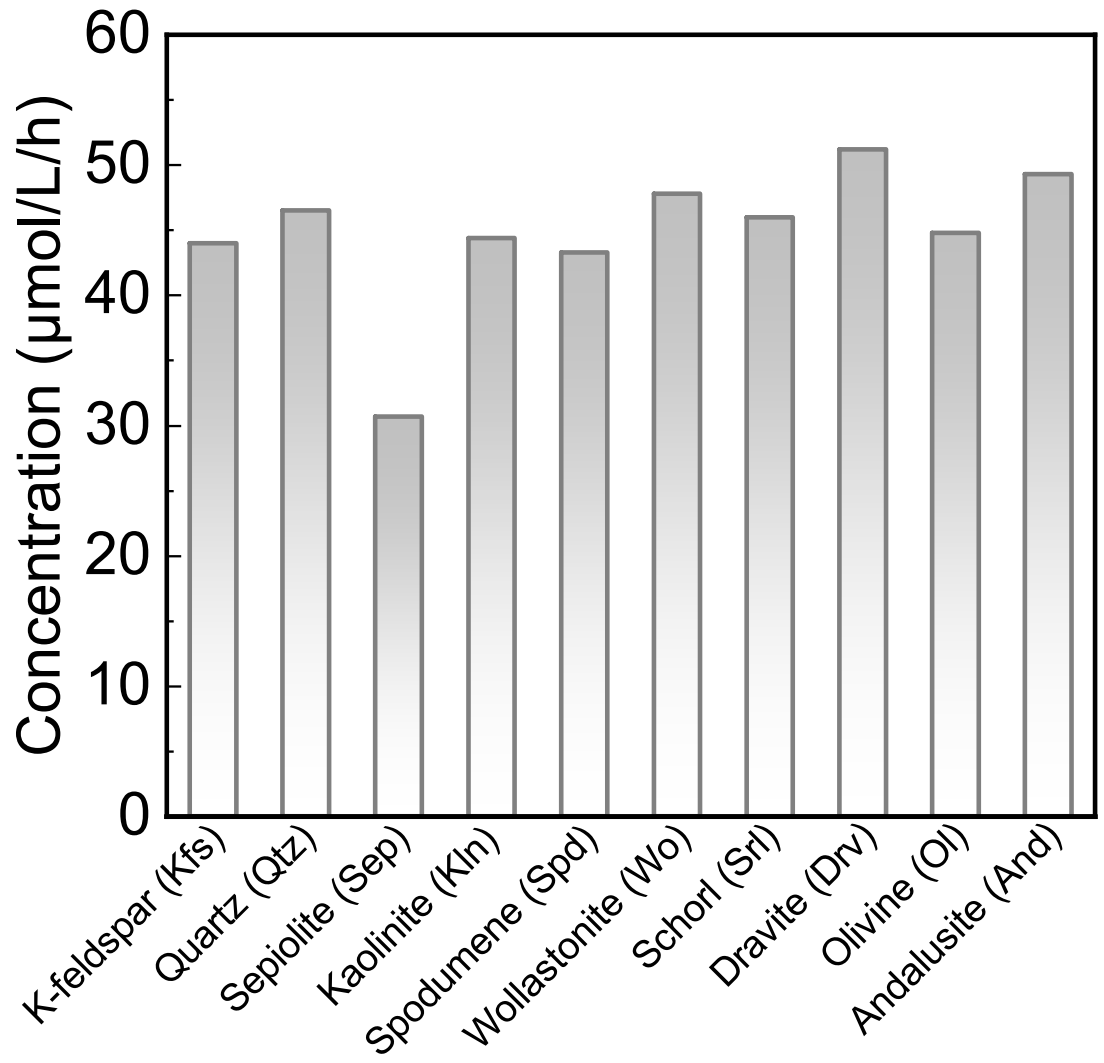

Supplementary Fig. 1 CEC performances of five categories (including ten types) of natural silicate minerals under ultrasonication. Source data are provided as a Source Data file.

Note: CEC performance was evaluated by measuring the concentration of H_2O_2 produced. The H_2O_2 concentration was quantified using an enzyme-based colorimetric assay[26].

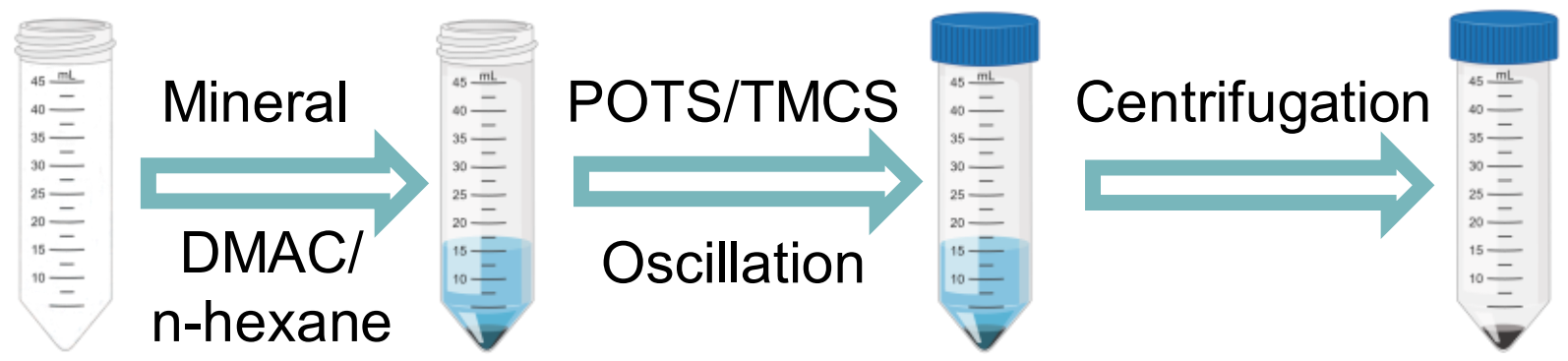

Supplementary Fig. 2 Modification process of silicate minerals.

Note: Silicate minerals were modified using distilled DMAC as the solvent and POTS as the modifier[12]. The reasons for choosing fluorination and alkylation are that fluorination is the most common method of hydrophobic modification in CEC, and alkylation can not only avoid environmental pollution from fluorine but also provide moderate hydrophobicity. The modified minerals were synthesized by first homogeneously mixing the mineral powder with the modifier, followed by centrifuging and drying. The modification mechanism involves cleavage of the ethyl group of POTS to form a silane alcohol, which then undergoes a dehydration and condensation reaction with the hydroxyl group and dangling oxygen atom on the surface of silicate minerals. This results in the grafting of carbon-fluorine chains onto the silicate minerals and the generation of low-molecular-weight alcohols[27]. The alkylation mechanism using TMCS is analogous; the solvent is changed to n-hexane.

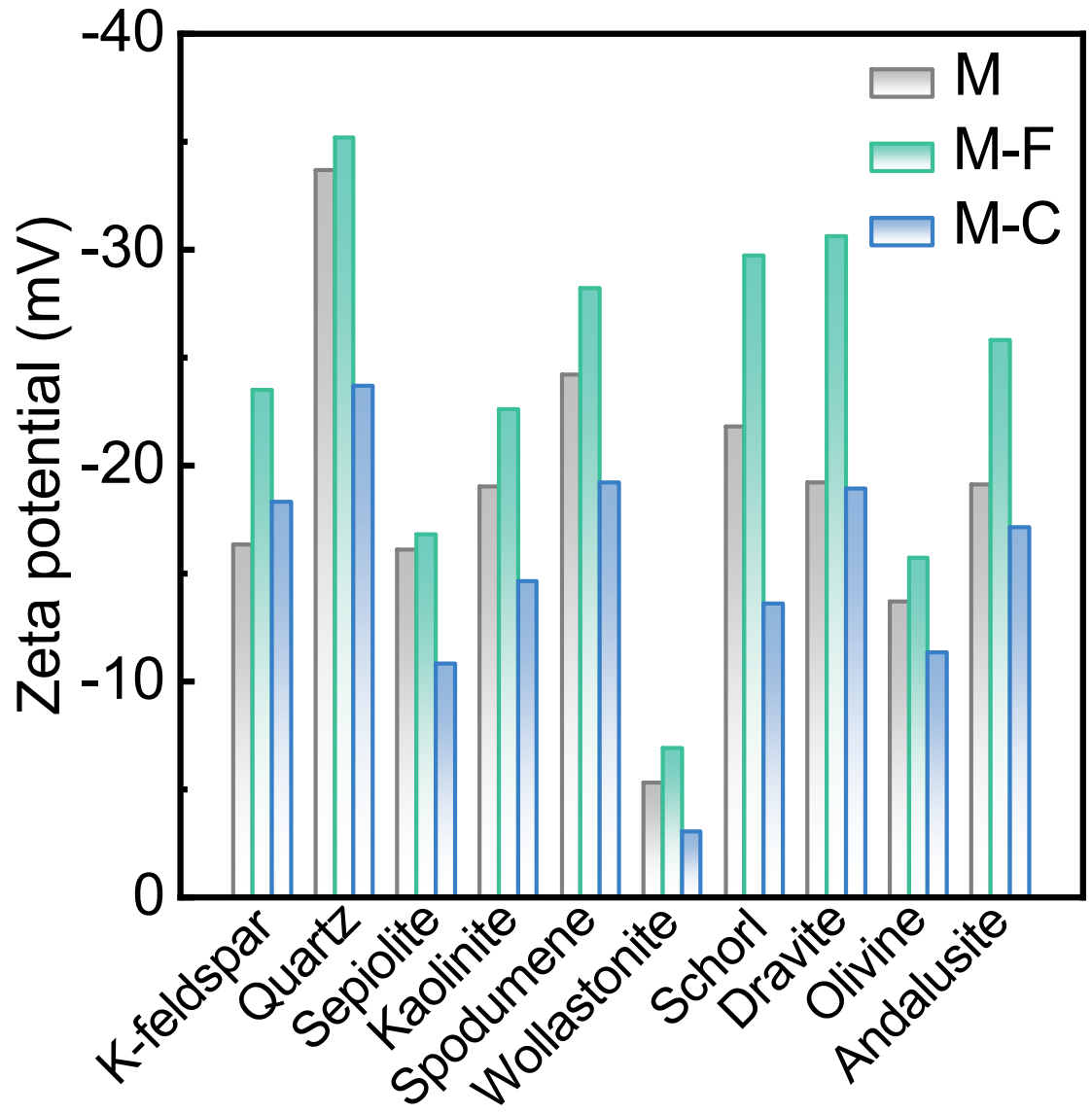

Supplementary Fig. 3 Zeta potentials of three types of minerals with different hydrophilicity/hydrophobicity. Source data are provided as a Source Data file.

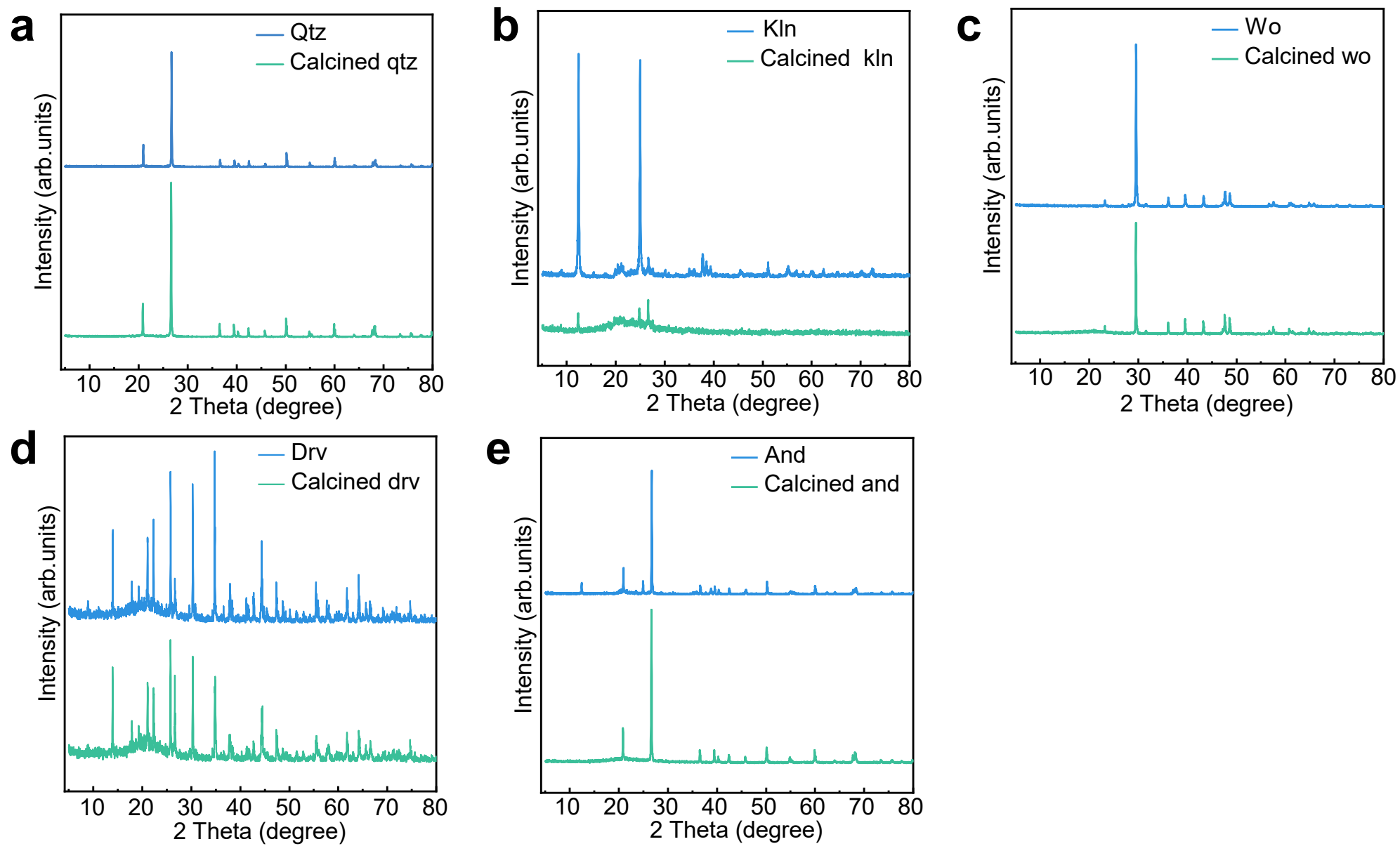

Supplementary Fig. 4 XRD patterns of five silicate minerals before and after calcination. a Quartz (qtz). **b** Kaolinite (kln). **c** Wollastonite (wo). **d** Dravite (drv). **e** Andalusite (and). Source data are provided as a Source Data file.

Note: Supplementary Fig. 4 presents the XRD patterns of the minerals before and after calcination. No new chemical phases were observed in the minerals after calcination.

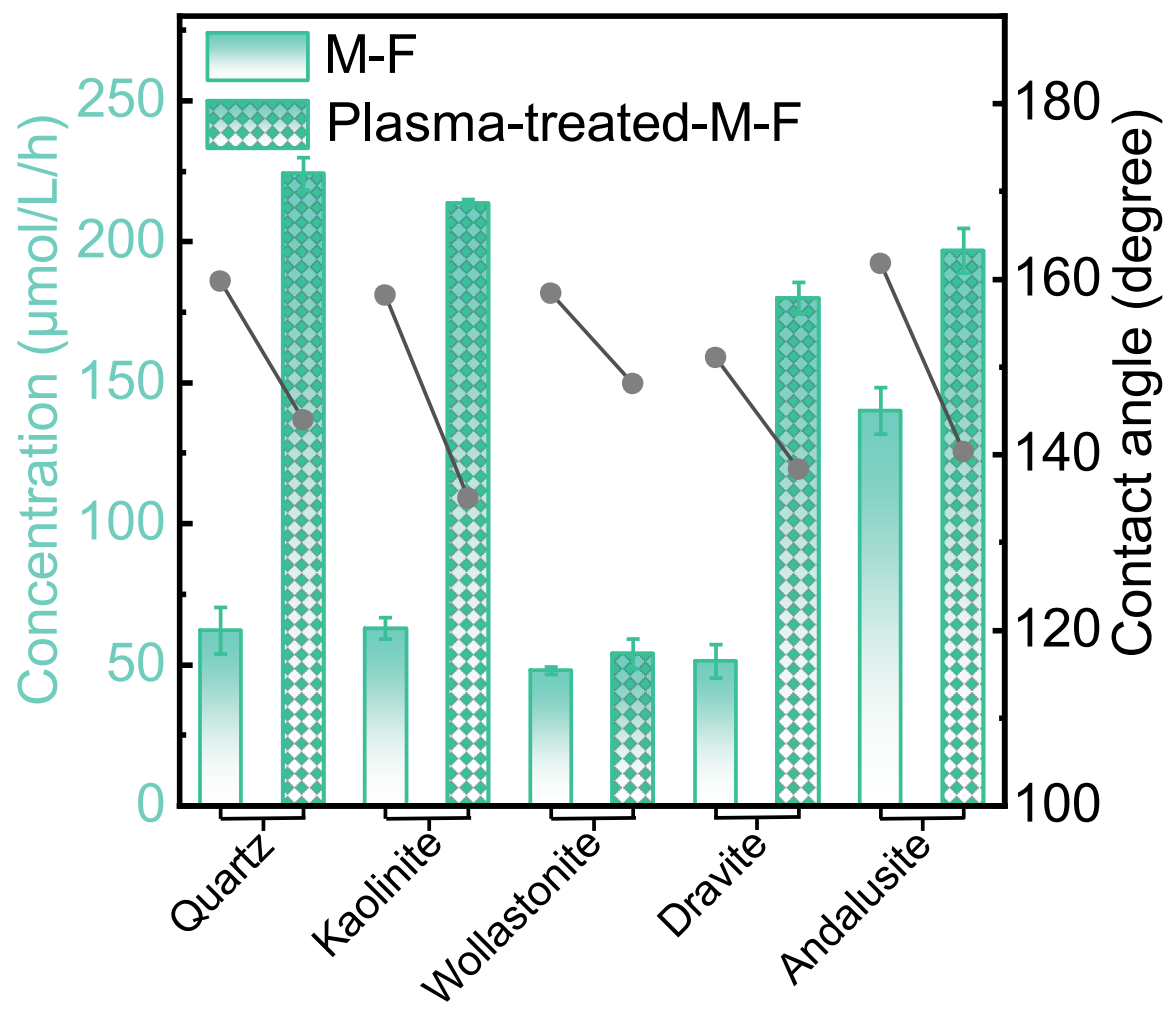

Supplementary Fig. 5 H_2O_2 production and contact angles of plasma-treated then fluorinated silicate minerals. Error bars represent standard deviation based on ten independent replicates. Source data are provided as a Source Data file.

Note: The plasma treatment can graft hydroxyl groups and dangling oxygen atoms onto the mineral surface. As shown in Supplementary Fig. 5, the silicate minerals were subjected to plasma treatment followed by POTS modification. After this treatment, the H_2O_2 yields for the five initially low-performing silicate minerals (Fig. 1d) were improved and the contact angles were decreased.

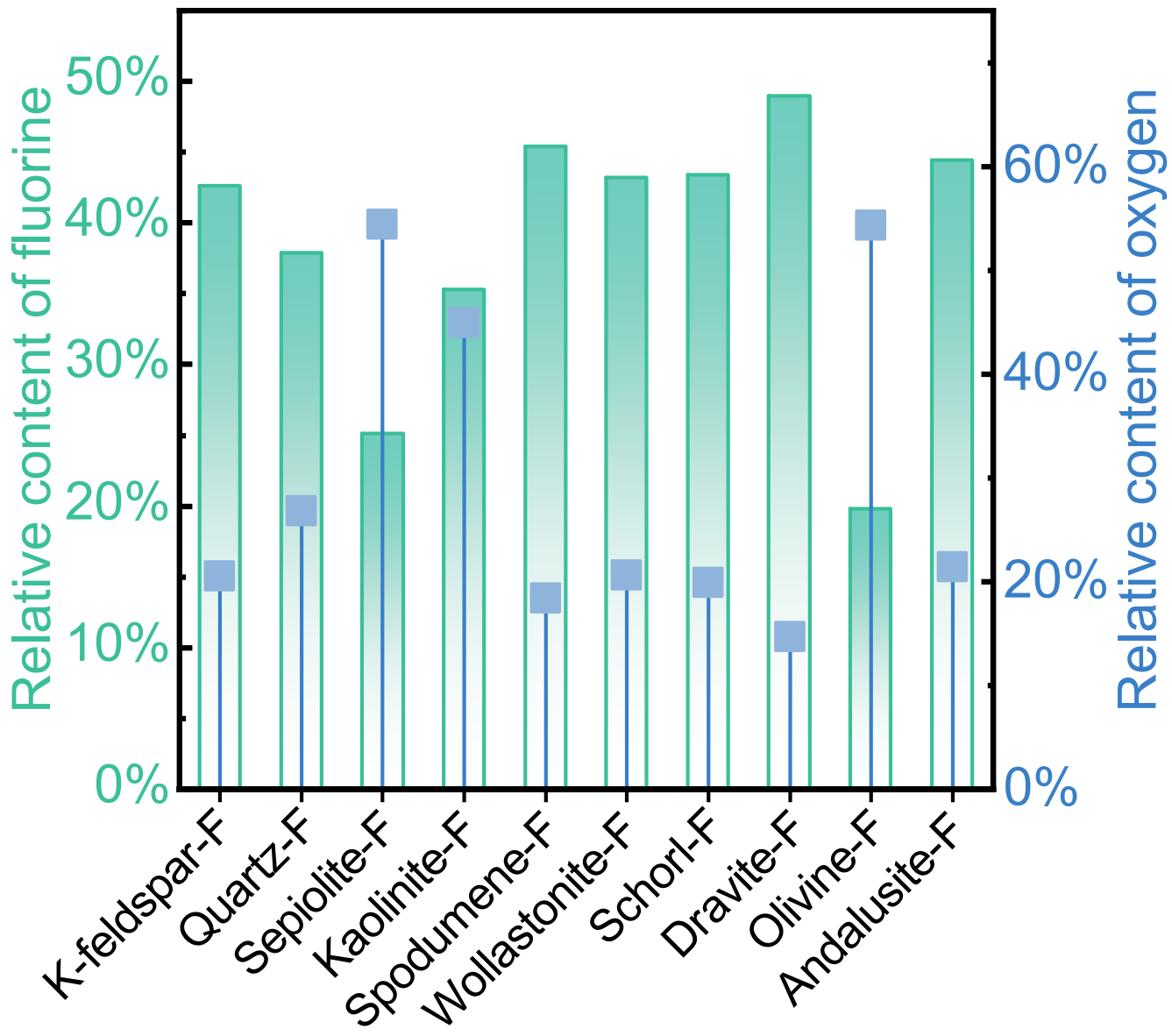

Supplementary Fig. 6 Relative fluorine content and relative oxygen content of M-F surfaces measured by XPS. Source data are provided as a Source Data file.

Note: Under the experimental conditions, fluorine coverage on the ten silicate minerals is close to saturation, and the steric hindrance of the modifier prevents complete reaction with all oxygen groups (hydroxyl groups, dangling oxygen atoms) on the mineral surfaces. This is supported by XPS data showing that lower relative fluorine content correlates with higher relative oxygen content (Supplementary Fig. 6). The improvement in hydrophilicity thus originates from the remaining oxygen groups that are not grafted by the modifier; the more such groups remain, the stronger the hydrophilicity. Olivine and sepiolite were both more hydrophilic than other minerals because of their high oxygen content (Fig. 1d).

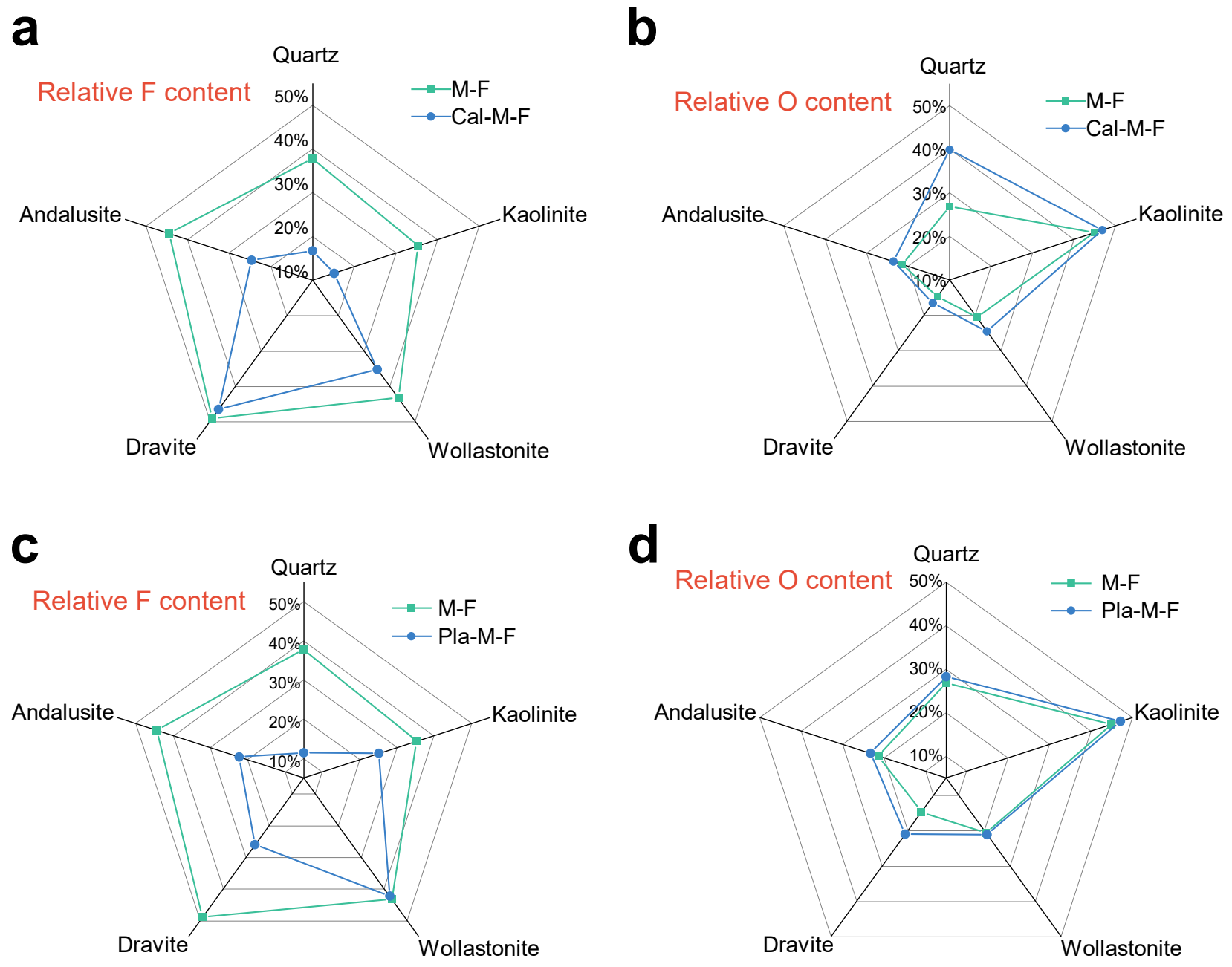

Supplementary Fig. 7 Relative fluorine content and relative oxygen content. a Relative fluorine content and **b** relative oxygen content of M-F and calcined then fluorinated minerals measured by XPS. **c** Relative fluorine content and **d** relative oxygen content of M-F and plasma-treated then fluorinated minerals measured by XPS. Source data are provided as a Source Data file.

Note: As shown in Supplementary Fig. 7, the relative fluorine content and relative oxygen content of M-F, calcined then fluorinated minerals (Cal-M-F), and plasma-treated then fluorinated minerals (Pla-M-F) were measured by XPS. The five extension directions represent five minerals, and the longer the extension length, the higher the relative content. It can be concluded that the Cal-M-F and Pla-M-F have higher relative oxygen content but lower relative fluorine content than the M-F.

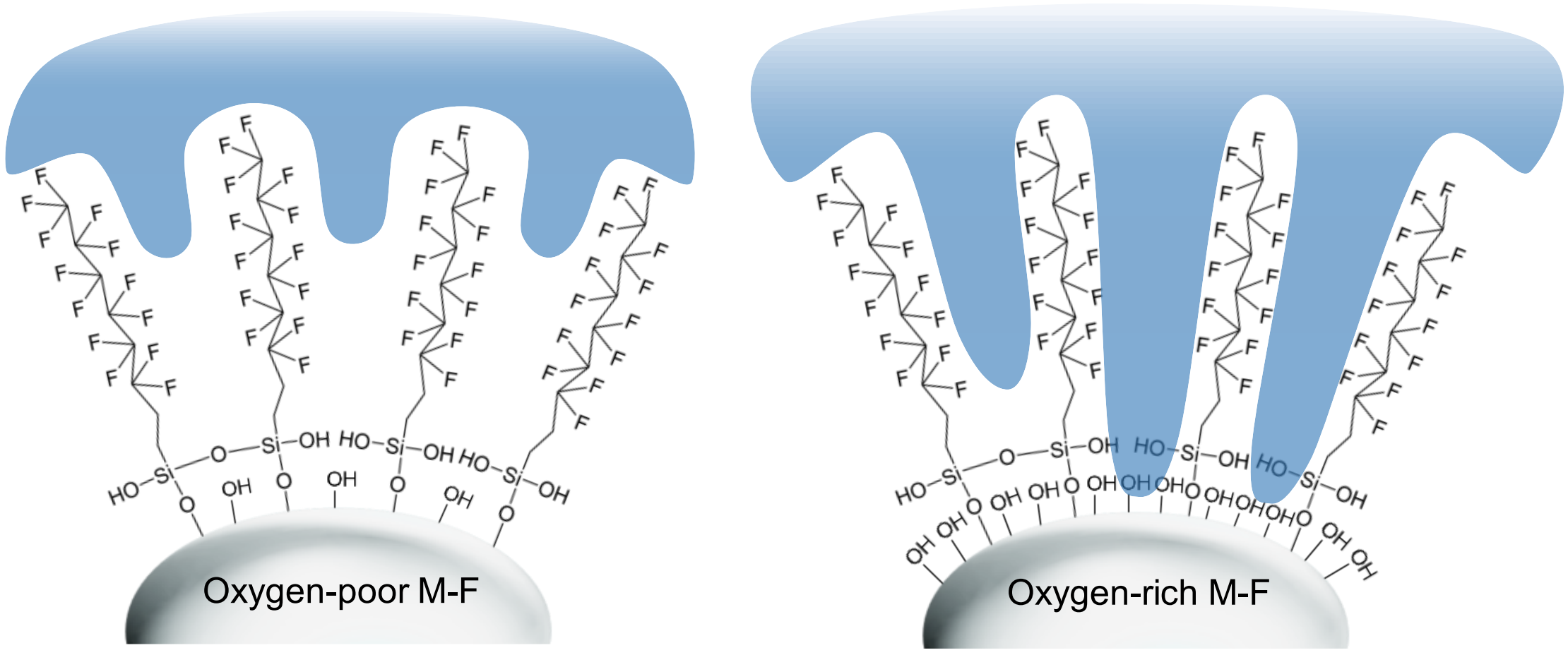

Supplementary Fig. 8 Schematic diagram illustrating the contact state between water and the surfaces of oxygen-rich M-F and oxygen-poor M-F after M is modified with sufficient modifier. Oxygen groups are represented by hydroxyl groups.

Note: When sufficient modifier is used to modify silicate minerals, both the oxygen-poor silicate minerals and oxygen-rich silicate minerals are fully modified, and carbon-fluorine chains are grafted onto the surfaces to saturation. However, oxygen-rich silicate minerals still have many ungrafted hydroxyl groups, which can attract water clusters and thus improve the interfacial contact between grafted carbon-fluorine chains and water.

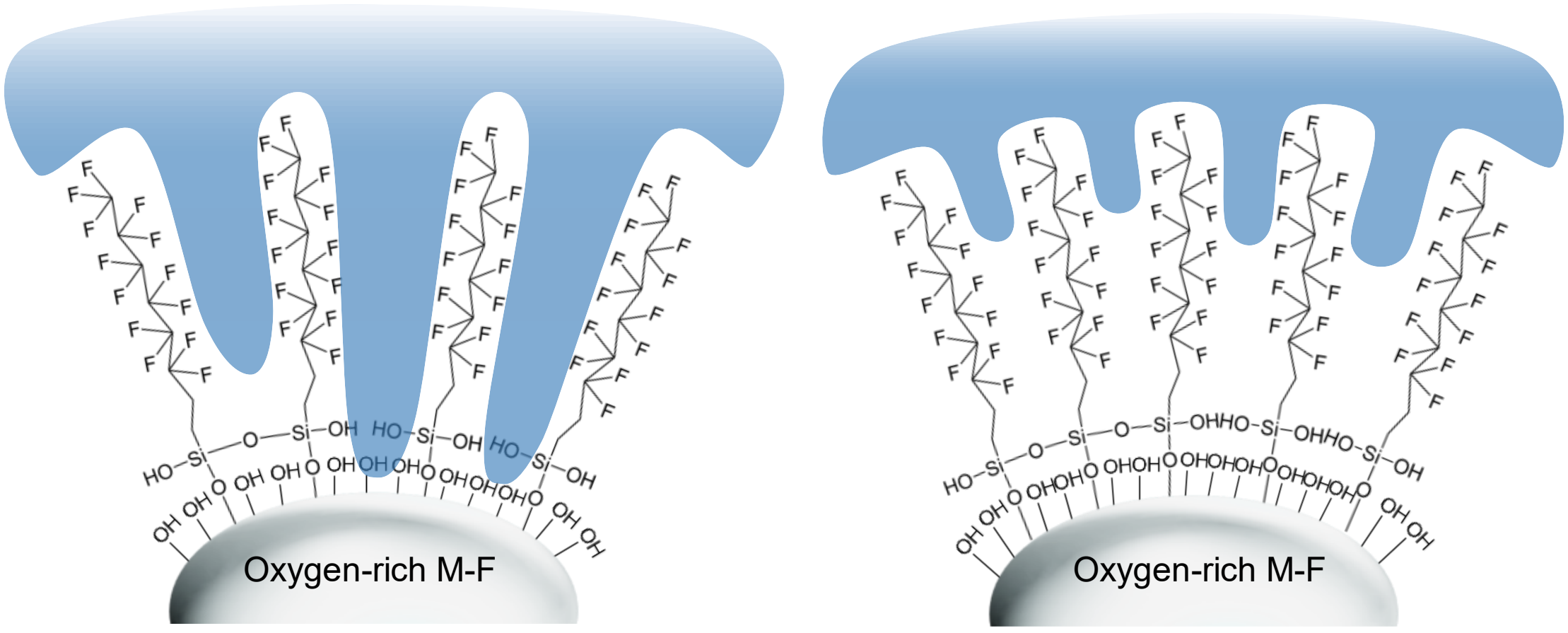

Supplementary Fig. 9 Schematic diagram illustrating the contact state between the surfaces of oxygen-rich M-F and water after modification of M with sufficient modifier (left) and excessive modifier (right). Oxygen groups are represented by hydroxyl groups.

Note: When sufficient and excessive amounts of modifier are used to modify the same oxygen-rich silicate mineral, the mineral modified with excessive modifier is grafted with more compact carbon-fluorine chains than that modified with sufficient modifier, resulting in fewer contact positions between water and fluorine groups and consequently poor CEC performance (Fig. 1f).

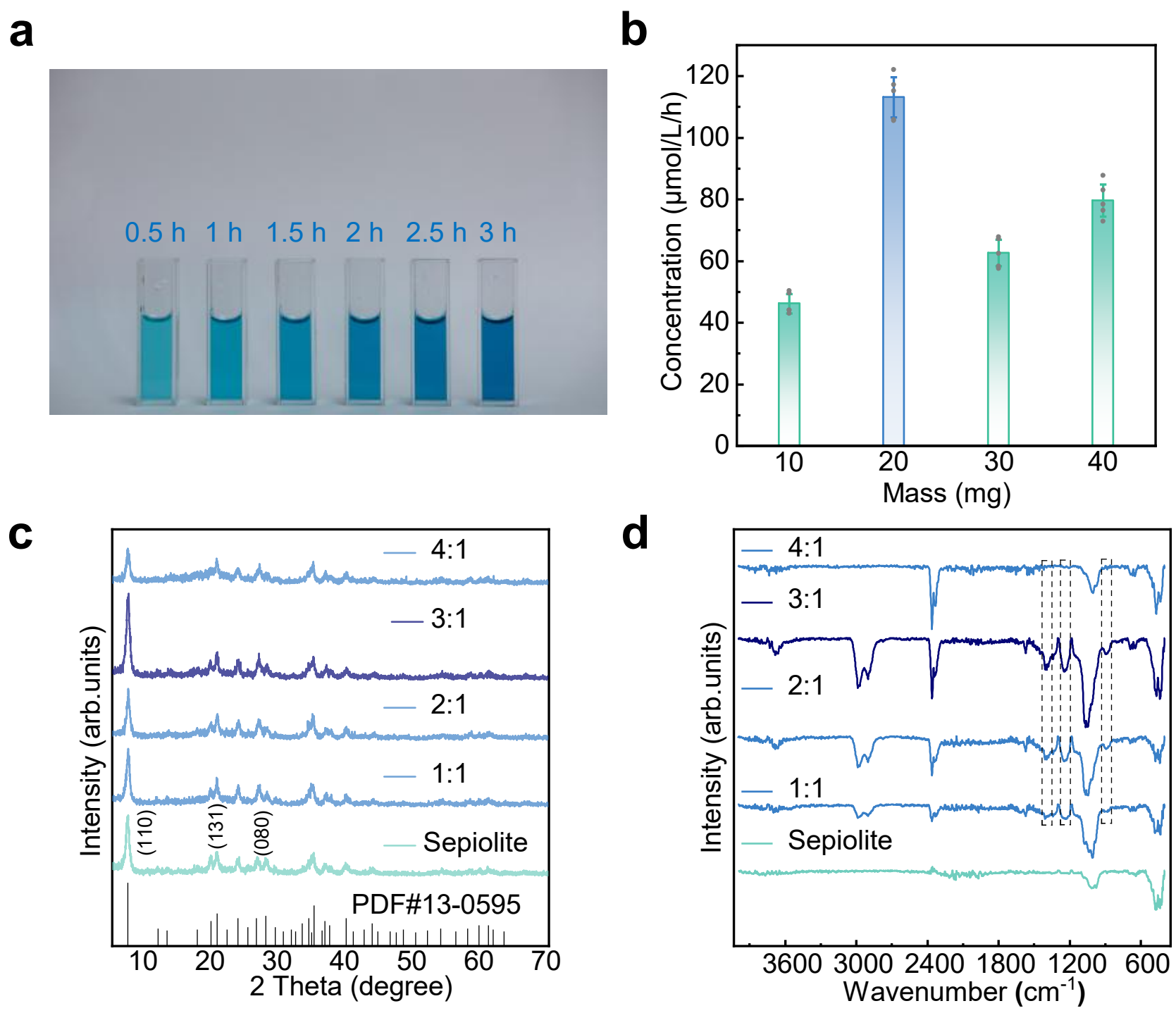

Supplementary Fig. 10 Influencing factors of CEC and characterization of sepiolite-F. a Enzyme-based colorimetric assay image. **b** Influence of sepiolite-F concentration on CEC performance. Error bars represent standard deviation based on five independent replicates. **c** XRD patterns of sepiolite-F. **d** FTIR spectra of sepiolite-F. Source data are provided as a Source Data file.

Note: Using sepiolite as an example, the factors influencing the CEC performance of sepiolite-F were investigated (Supplementary Note 2).

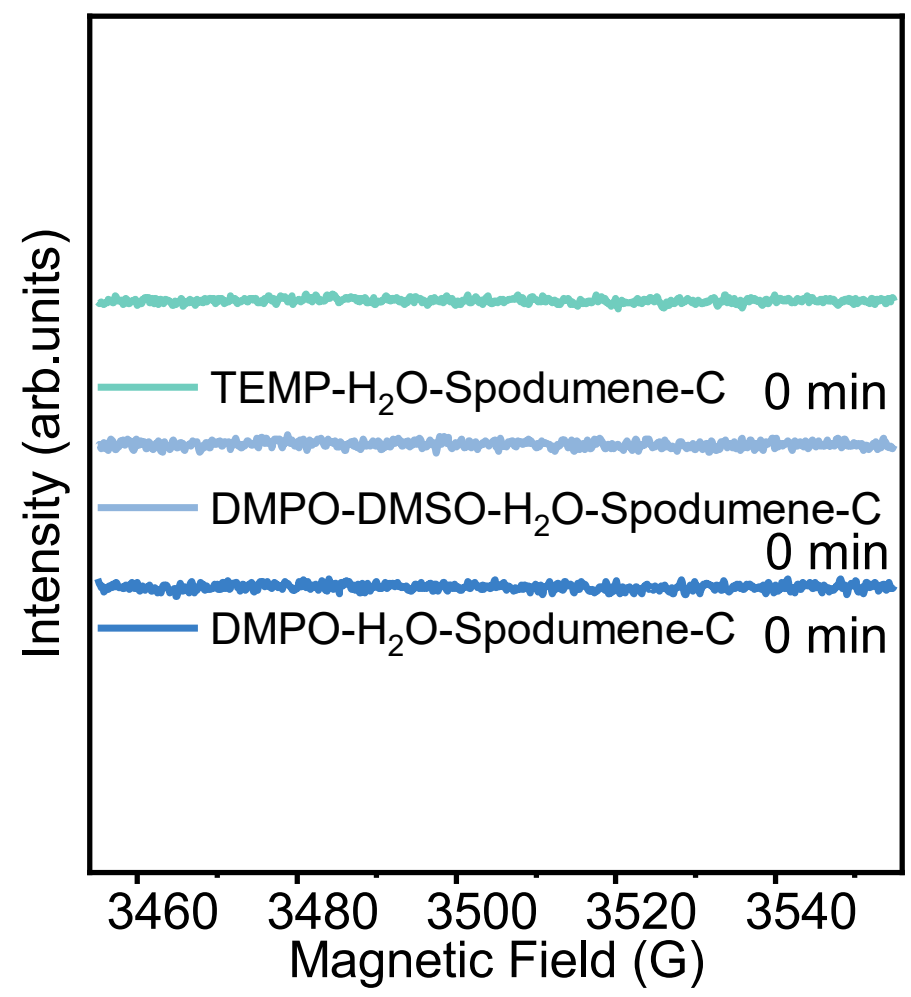

Supplementary Fig. 11 Electron paramagnetic resonance (EPR) spectra of spodumene-C under ultrasonication for 0 min. Source data are provided as a Source Data file.

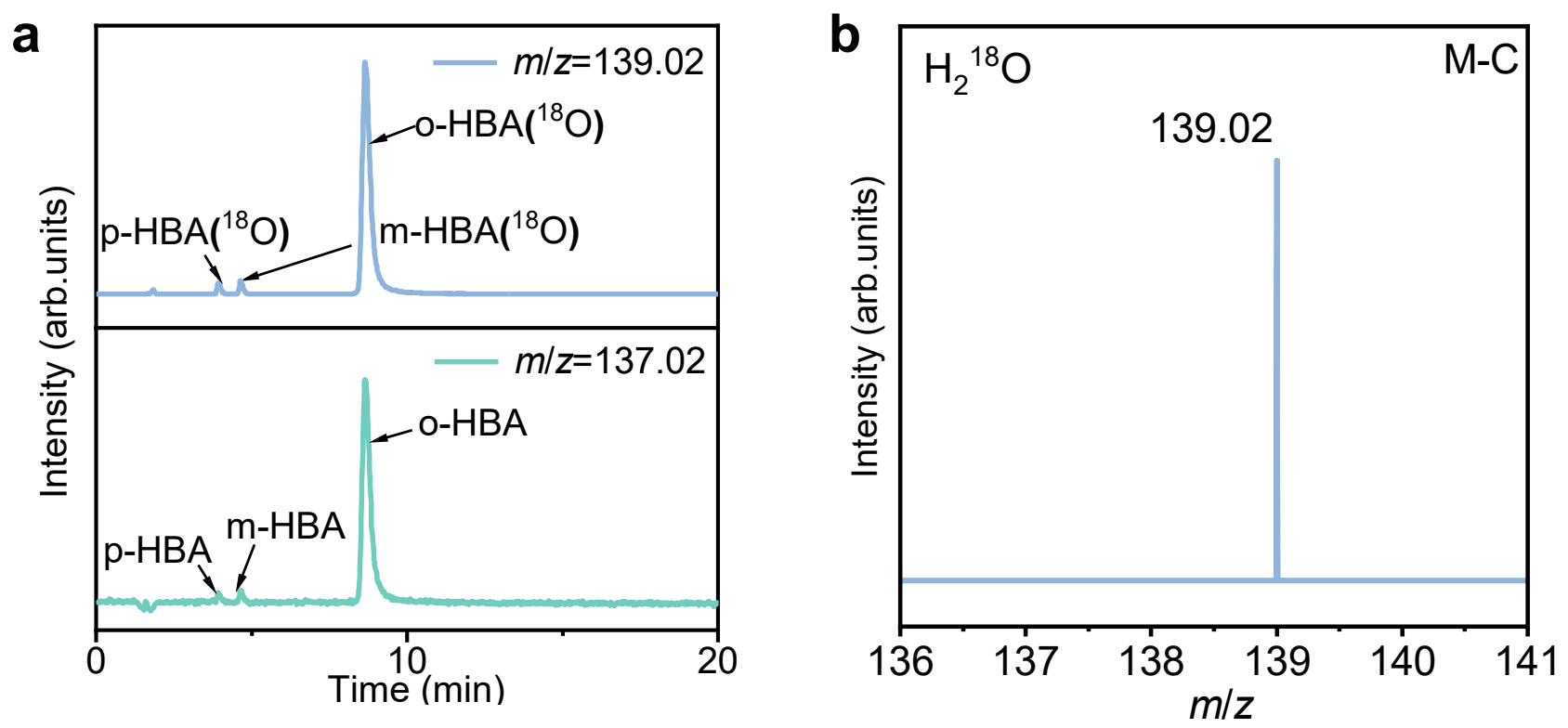

Supplementary Fig. 12 Liquid chromatography-mass spectrometry (LC-MS) analysis. a Total ion chromatograms of the reactions of $\bullet^{16}OH$ and $\bullet^{18}OH$ with benzoic acid. **b** Mass spectra of the ^{18}O-labeled water after CEC of M-C obtained by LC–MS. Source data are provided as a Source Data file.

Note: The ^{18}O-incorporated species include para-hydroxybenzoic acid, ortho-hydroxybenzoic acid, and meta-hydroxybenzoic acid.

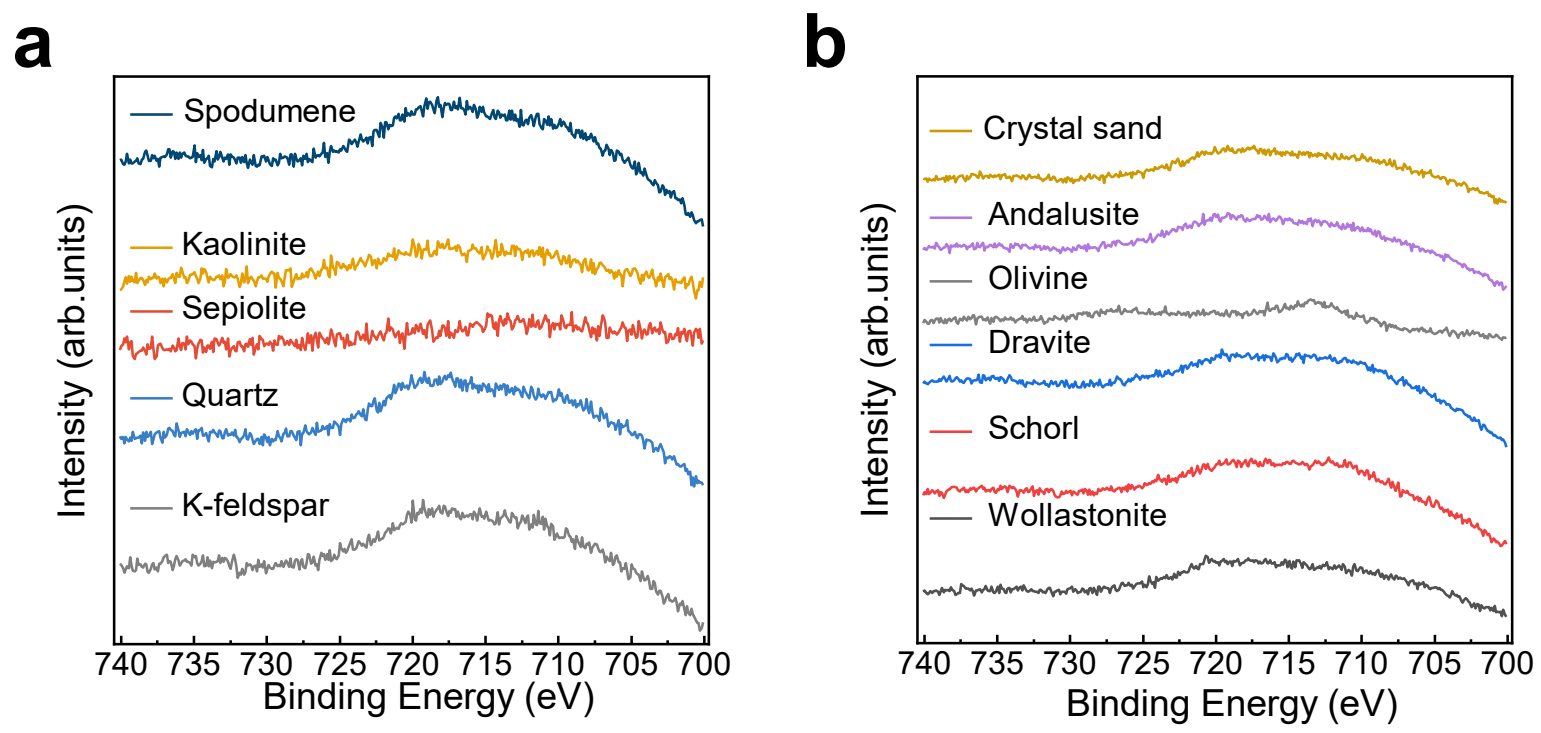

Supplementary Fig. 13 The Fe 2*p* XPS spectra of original silicate minerals and crystal sand. a K-feldspar, quartz, sepiolite, kaolinite, and spodumene. **b** Wollastonite, schorl, dravite, olivine, andalusite, and crystal sand. Source data are provided as a Source Data file.

Note: Metal-catalyzed background reactions are expected to contribute only a limited ROS generation compared with the dominant CEC process. Fe 2*p* spectra of the ten silicate minerals do not exhibit any obvious characteristic peaks (Supplementary Fig. 13). At such low surface concentrations, and with these metals predominantly incorporated within the silicate lattice rather than existing as freely dissolved ions, their capacity to drive substantial Fenton-like reactions is considered limited. Similarly, the crystal sand later used in the circulating water-flow experiments contains little surface Fe. Moreover, the primary performance metric evaluated in this study is H_2O_2 production during CEC. A Fenton-like reaction would consume H_2O_2 to generate •OH. Consequently, even if a minor Fenton-like contribution occurred, it would not artificially inflate the measured H_2O_2 production. Instead, such a side reaction would tend to decrease the detectable H_2O_2 level.

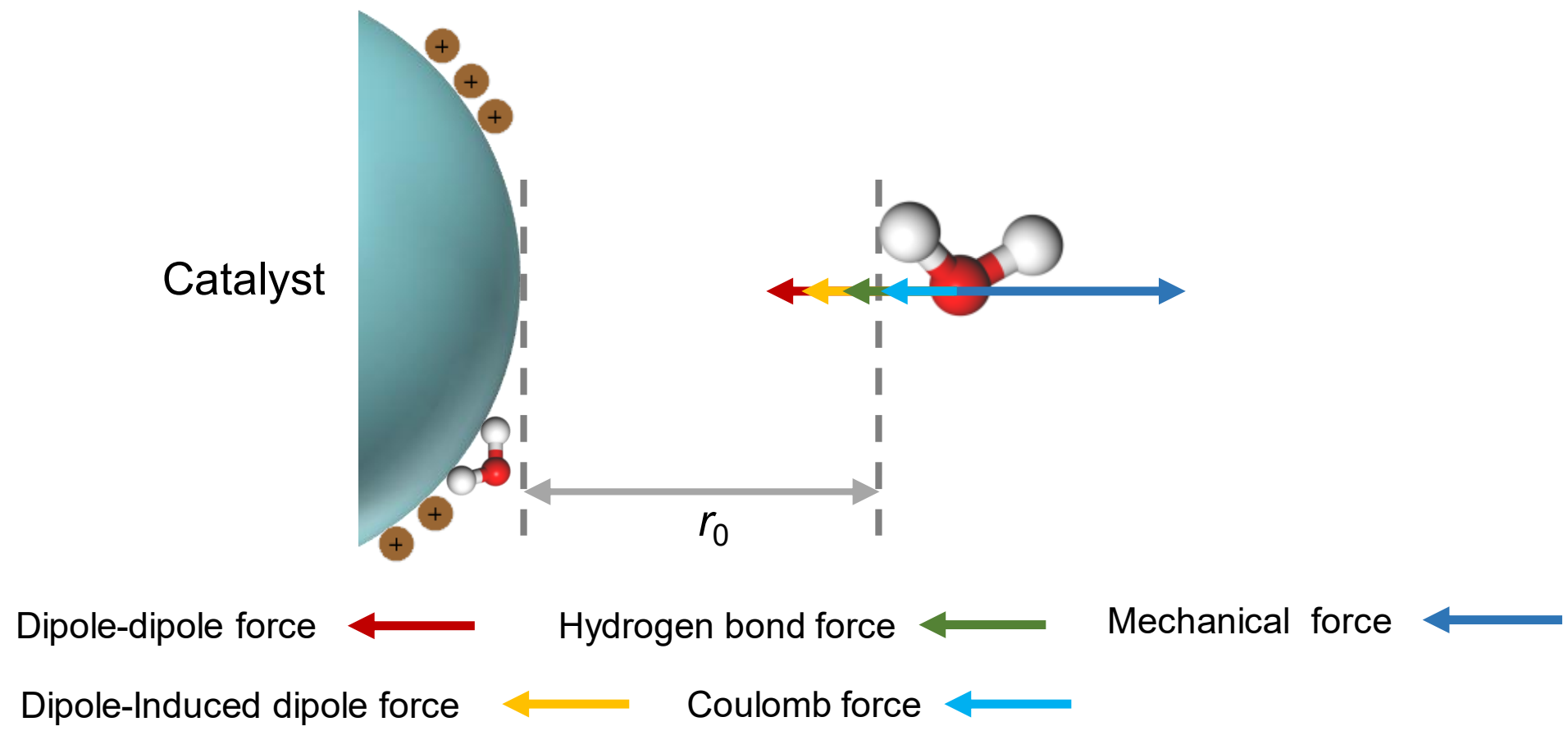

Supplementary Fig. 14 Types of interactions between H_2O molecules and catalyst molecules.

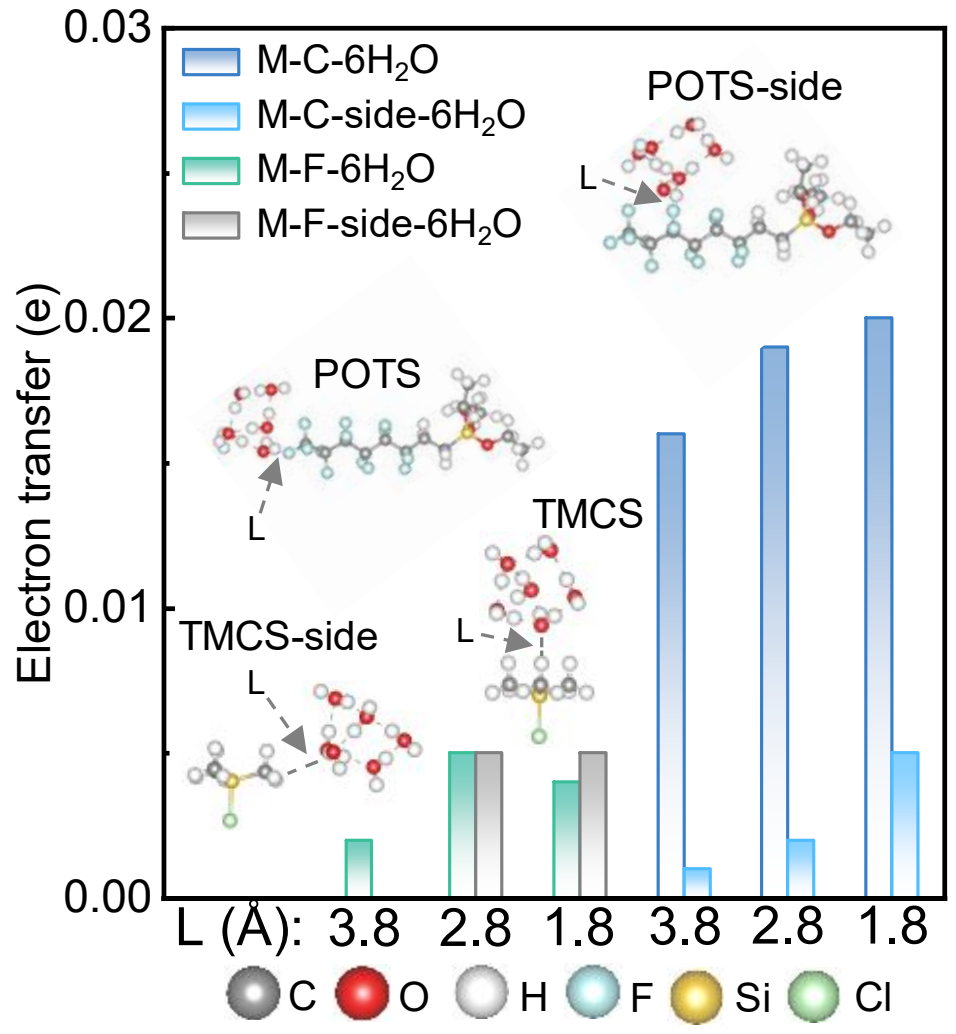

Supplementary Fig. 15 Electron transfer between hydrophobic molecules and a hexameric water cluster as a function of configurations of hydrophobic molecules based on DFT simulations. Source data are provided as a Source Data file.

Note: As shown in Supplementary Fig. 15, when a water cluster approaches M-F from the front or the side, the amounts of electron transfer from the front (0.004 *e* at 1.8 Å) and from the side (0.005 *e* at 1.8 Å) are almost the same. However, when a water cluster approaches M-C from the front or the side, the amount of electron transfer from the front (0.02 *e* at 1.8 Å) is much greater than that from the side (0.005 *e* at 1.8 Å), but the amount of electron transfer of M-C from the side is still at the same level as those of M-F from the front and the side.

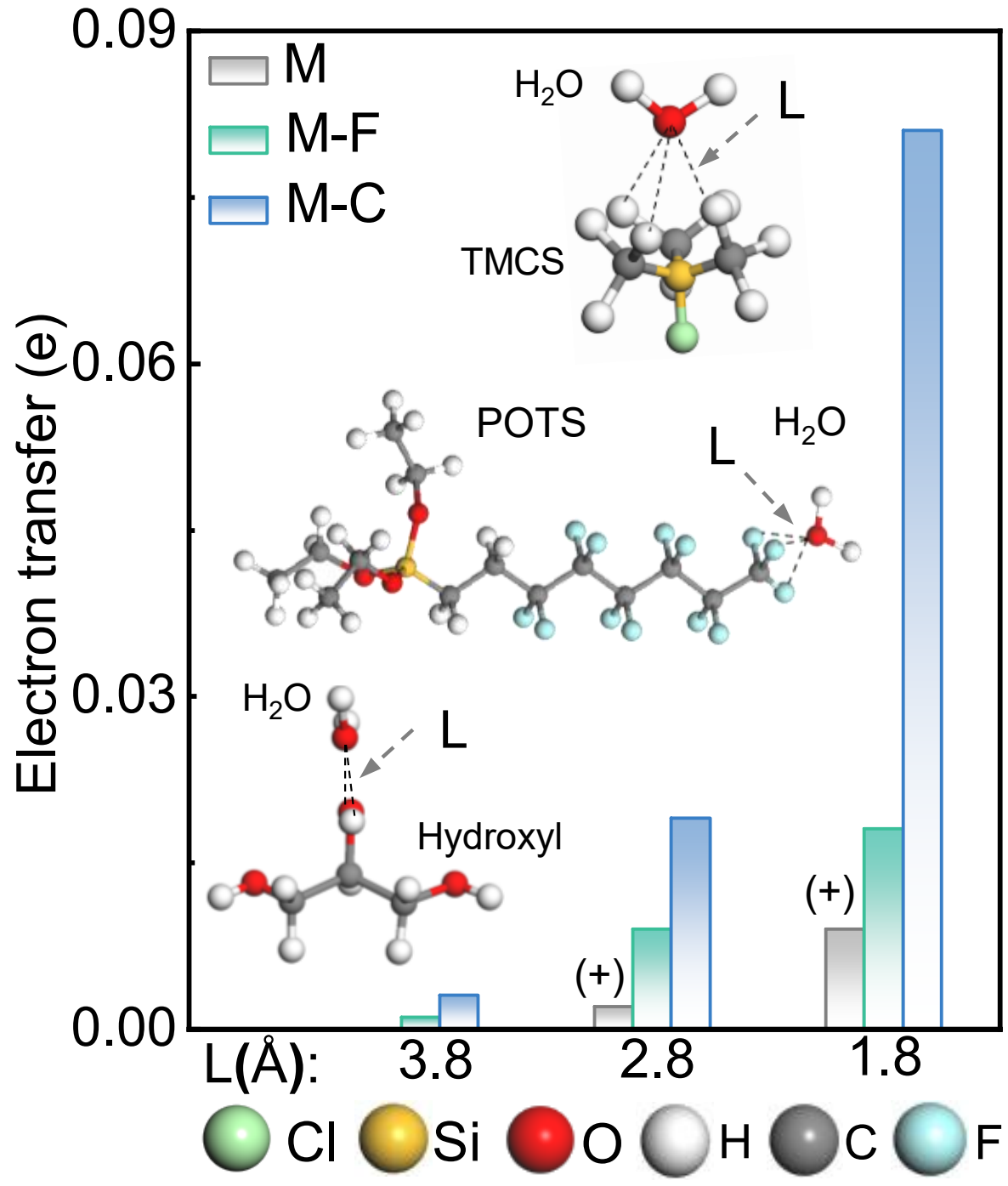

Supplementary Fig. 16 Electron transfer between hydrophilic/hydrophobic molecules and a single H_2O molecule as a function of distances based on DFT simulations. Source data are provided as a Source Data file.

Note: The amount of electron transfer between the water cluster and the catalysts is less affected by distance (Fig. 2d), while the amount of electron transfer between a single water molecule and the catalysts varies greatly with distance (Supplementary Fig. 16), because the water cluster has a larger range of electron clouds, reducing the sharp fluctuations in electron cloud overlap caused by distance changes. Among the catalysts, M, which has the highest hydrophilicity, exhibits the highest amount of electron transfer with the water cluster (0.023 e at 1.8 Å), but the lowest amount of electron transfer with a single water molecule (Supplementary Fig. 16). This is due to the more rapid increase in hydrogen bonding between M and the water cluster and the stronger overlap of intermolecular electron clouds compared to those in M-F and M-C. Moreover, in the electron transfer process between M and H_2O, M always plays the role of an electron

donor. In contrast, the amount of electron transfer between M-C and a single water molecule is the highest among the three samples.

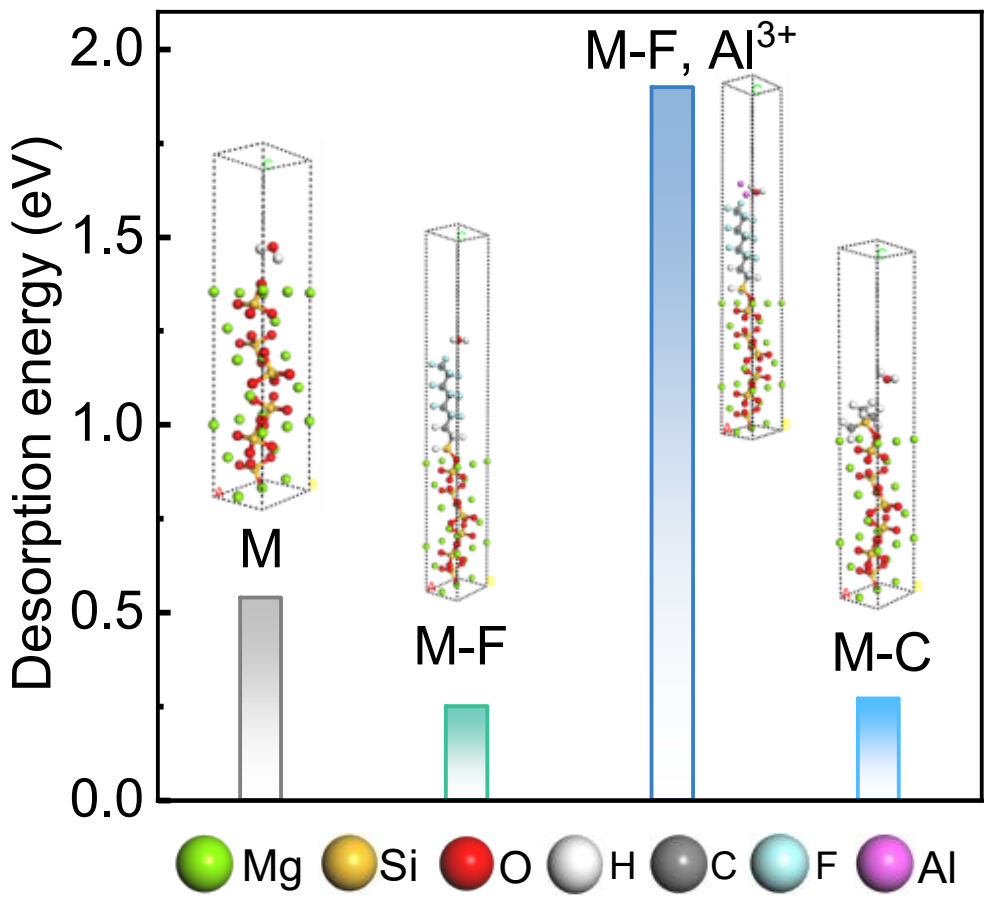

Supplementary Fig. 17 Desorption energy of a single H_2O molecule on hydrophilic and hydrophobic mineral surfaces calculated by DFT simulations. Source data are provided as a Source Data file.

Note: Among M (0.54 eV), M-F (0.25 eV), and M-C (0.27 eV), the most hydrophilic catalyst (M) exhibits the highest desorption energy, while the most hydrophobic catalyst (M-F) displays the lowest (Fig. 1b and Supplementary Fig. 17). Both the weak electron-withdrawing ability and the limited charge separation capacity of M contribute to its negligible CEC performance. In contrast, M-F exhibits the strongest electron-withdrawing ability and the most efficient charge separation. However, its experimentally observed CEC performance is inferior to that of M-C, primarily due to the reduced electron cloud overlap probability with H_2O resulting from the superhydrophobic nature of M-F (Fig. 2c). Thus, M-C achieves the highest CEC performance by maintaining a balanced contact process, allowing water to approach more easily while retaining a removal capacity comparable to that of M-F. In addition, M-F is strongly negatively charged and will adsorb metal ions and water molecules to form hydrated ions, which may greatly enhance its hydrophilicity and suppress the CEC performance under natural water conditions.

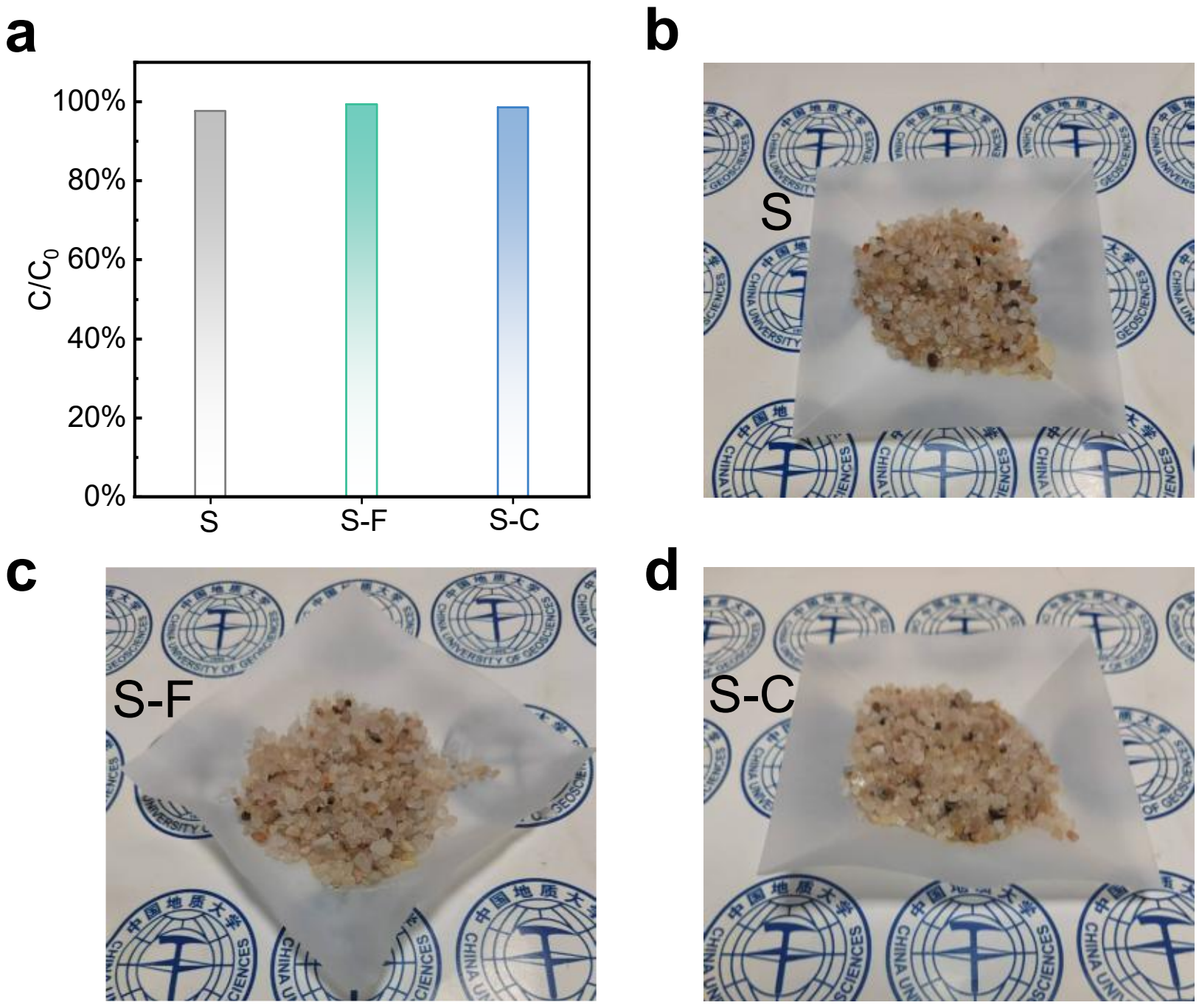

Supplementary Fig. 18 Adsorption of MO by S, S-F, and S-C, and the corresponding images after adsorption. a Comparison of MO absorbance before and after adsorption. **b** Image of S after adsorption of MO. **c** Image of S-F after adsorption of MO. **d** Image of S-C after adsorption of MO. Source data are provided as a Source Data file.

Note: The adsorption capacity was quantified by immersing S, S-F, and S-C in MO solution for 48 h. As shown in Supplementary Fig 18, the adsorption of MO by the three types of stones was extremely weak. Source data are provided as a Source Data file.

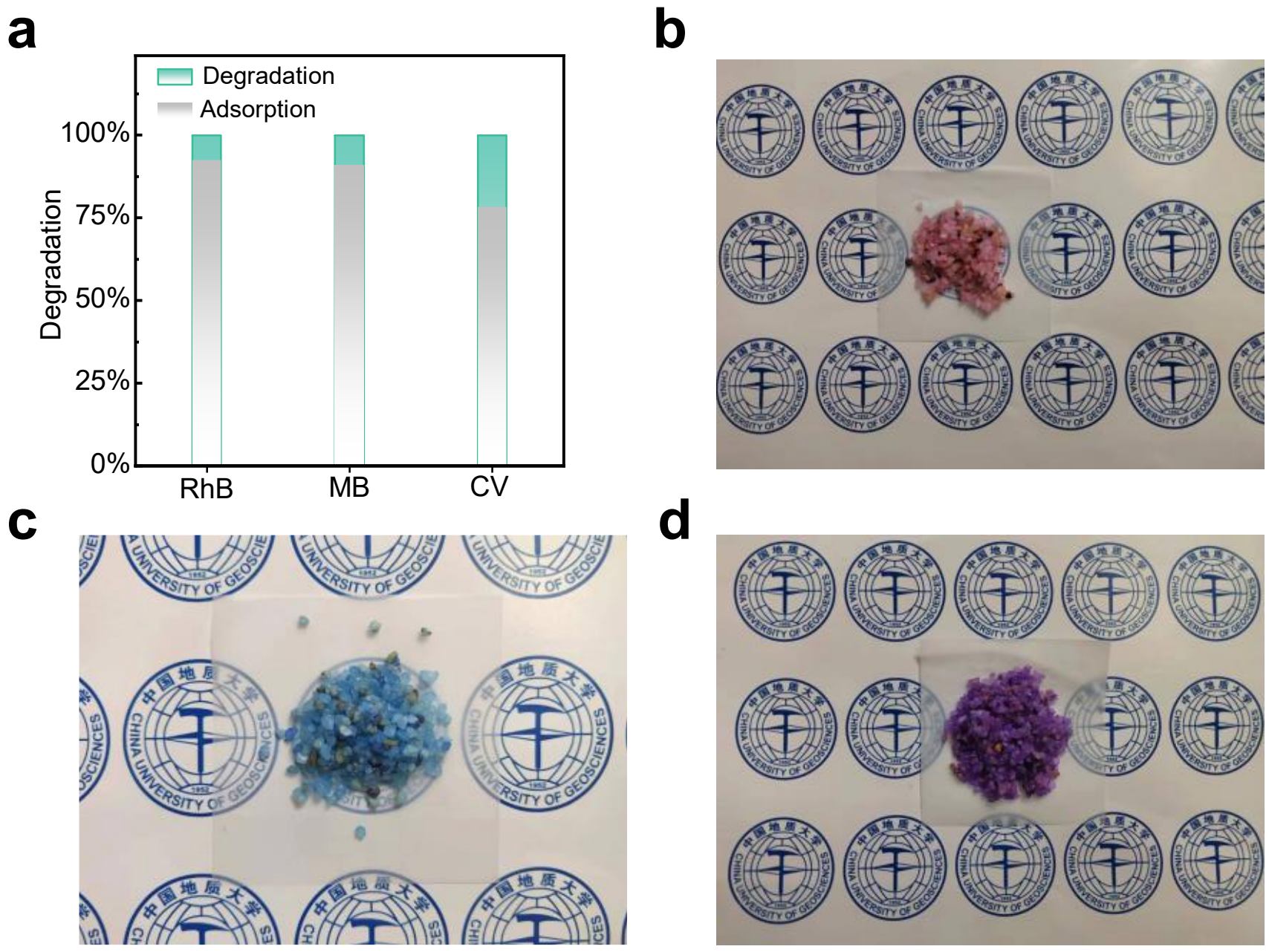

Supplementary Fig. 19 Performance of S-F in degrading three cationic dyes. a Degradation and adsorption behavior of S-F toward the three dyes. **b** Image of S-F after treatment of rhodamine B (RhB). **c** Image of S-F after treatment of methylene blue (MB). **d** Image of S-F after treatment of crystal violet (CV). Source data are provided as a Source Data file.

Note: The addition of 20 g S-F to 150 mL solutions of each dye (5 mg/L) resulted in >99.9% removal within 2 h. To distinguish adsorption from degradation, adsorption capacity was quantified by immersing S-F in MB, RhB, and CV solutions for 48 h to reach adsorption saturation. As shown in Supplementary Fig. 19a, the majority of dye removal was attributed to adsorption. This conclusion is further supported by Supplementary Fig. 19b-d, in which S-F exhibited chromatic changes corresponding to the respective dyes, confirming adsorption of cationic species onto the fluorinated surface during circulation. Consequently, these dyes were effectively removed from the aqueous system primarily through adsorption.

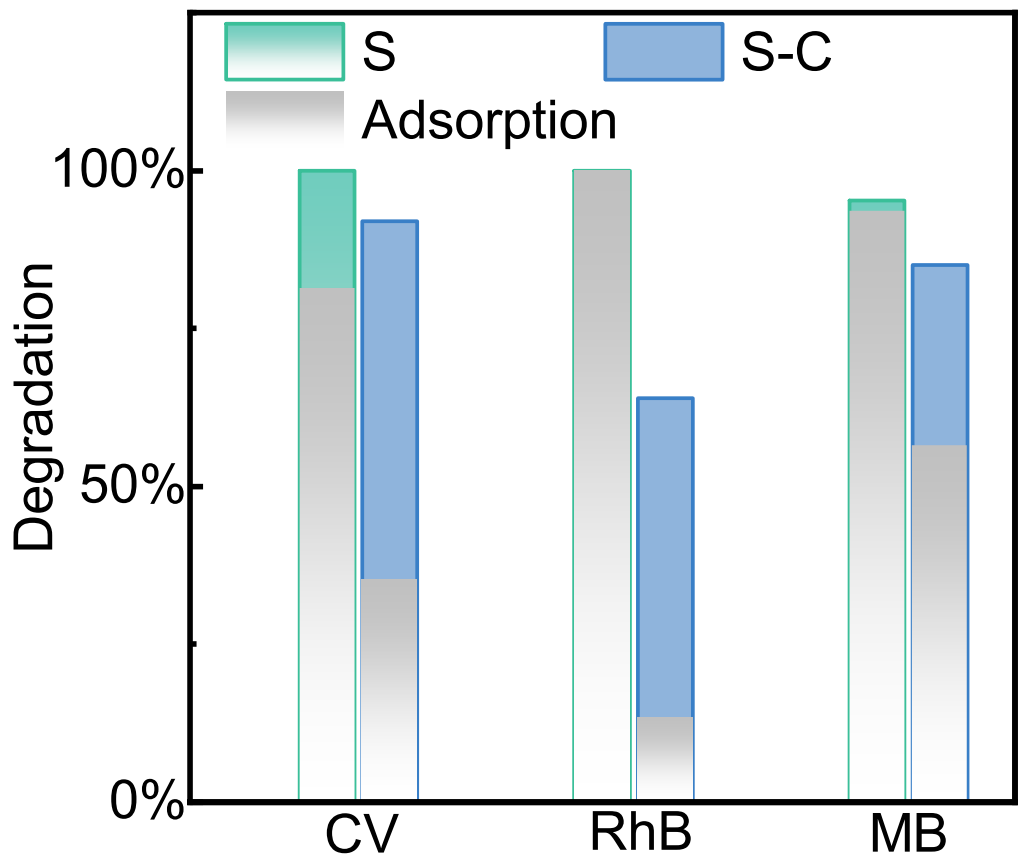

Supplementary Fig. 20 Comparison of adsorption and degradation of MB, RhB, and CV by S and S-C. Source data are provided as a Source Data file.

Note: The addition of 20 g S or S-C to 150 mL solutions of each dye (5 mg/L), respectively, and the corresponding removal behaviors are presented in Supplementary Fig. 20. Adsorption capacity was quantified by immersing S and S-C in MB, RhB, and CV solutions for 48 h to achieve adsorption saturation. As shown in Supplementary Fig. 20-21, adsorption of all three dyes by S was greater than that by S-C (Supplementary Note 6).

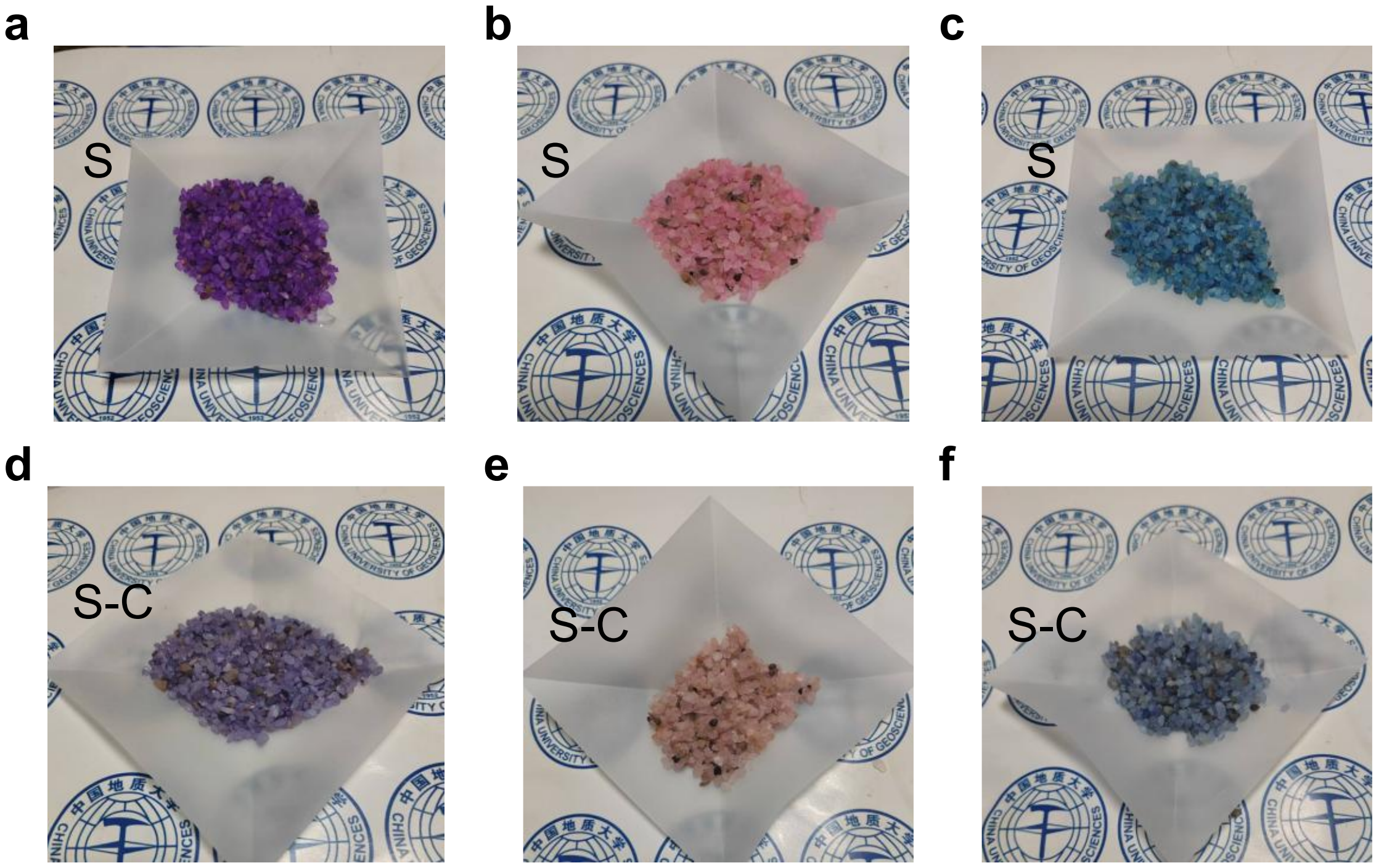

Supplementary Fig. 21 Comparison of images after adsorption of the three dyes by S and S-C. **a** Image of S after treatment of crystal violet (CV). **b** Image of S after treatment of rhodamine B (RhB). **c** Image of S after treatment of methylene blue (MB). **d** Image of S-C after treatment of CV. **e** Image of S-C after treatment of RhB. **f** Image of S-C after treatment of MB.

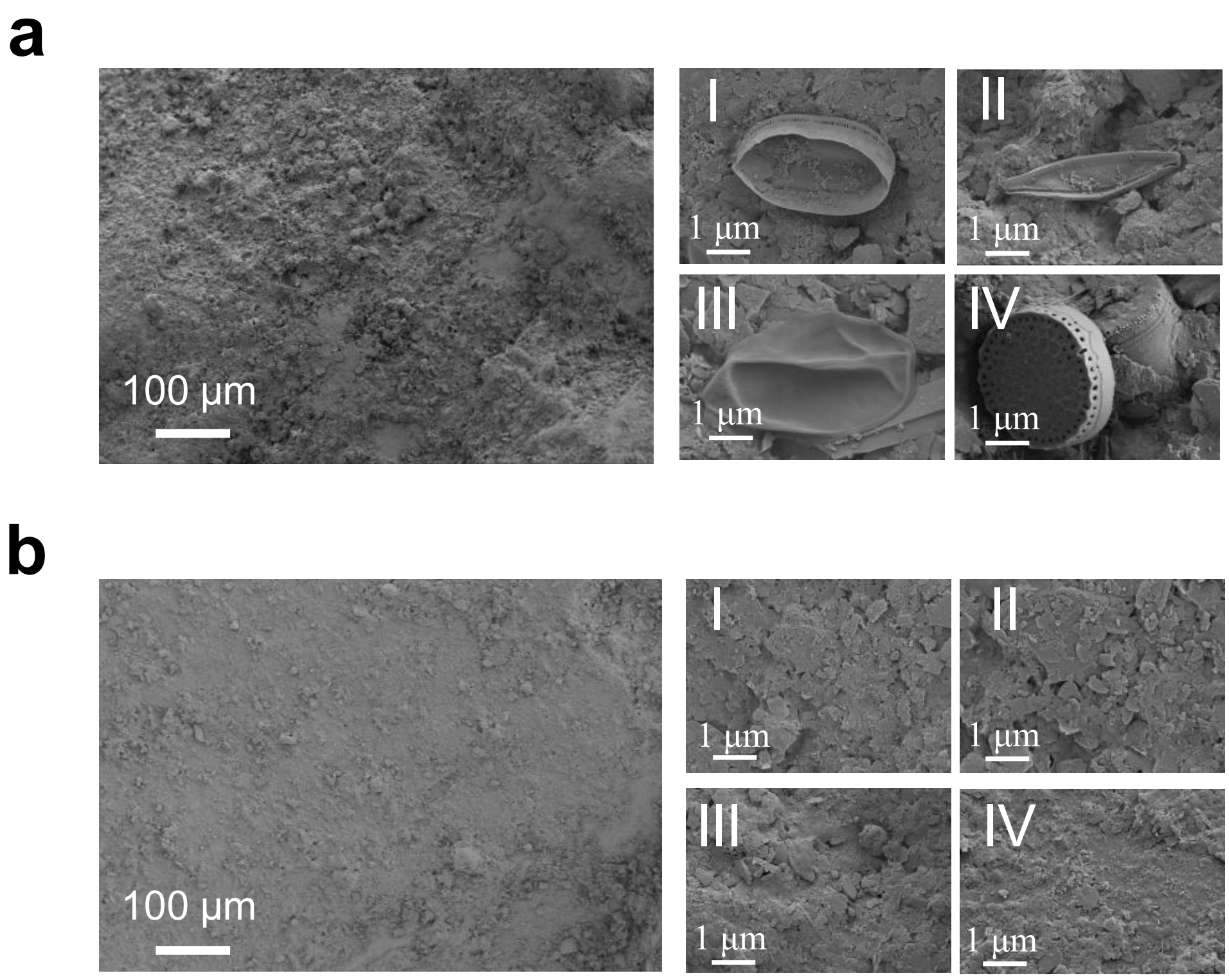

Supplementary Fig.22 SEM images of a S and b S-C. The SEM experiment was repeated five times, yielding similar results.

Note: The surfaces of S and S-C deployed in the river for one month (from January 13 to February 13, 2025) were measured by SEM. The diatoms surrounding the stone catalysts were analyzed to evaluate CEC performance and the effect on the real river environment. Diatom densities were quantified by averaging counts from 1 mm^2 sampling areas (Fig. 3h and Supplementary Fig. 22), revealing 3.75-fold higher colonization on S than on S-C under identical conditions.

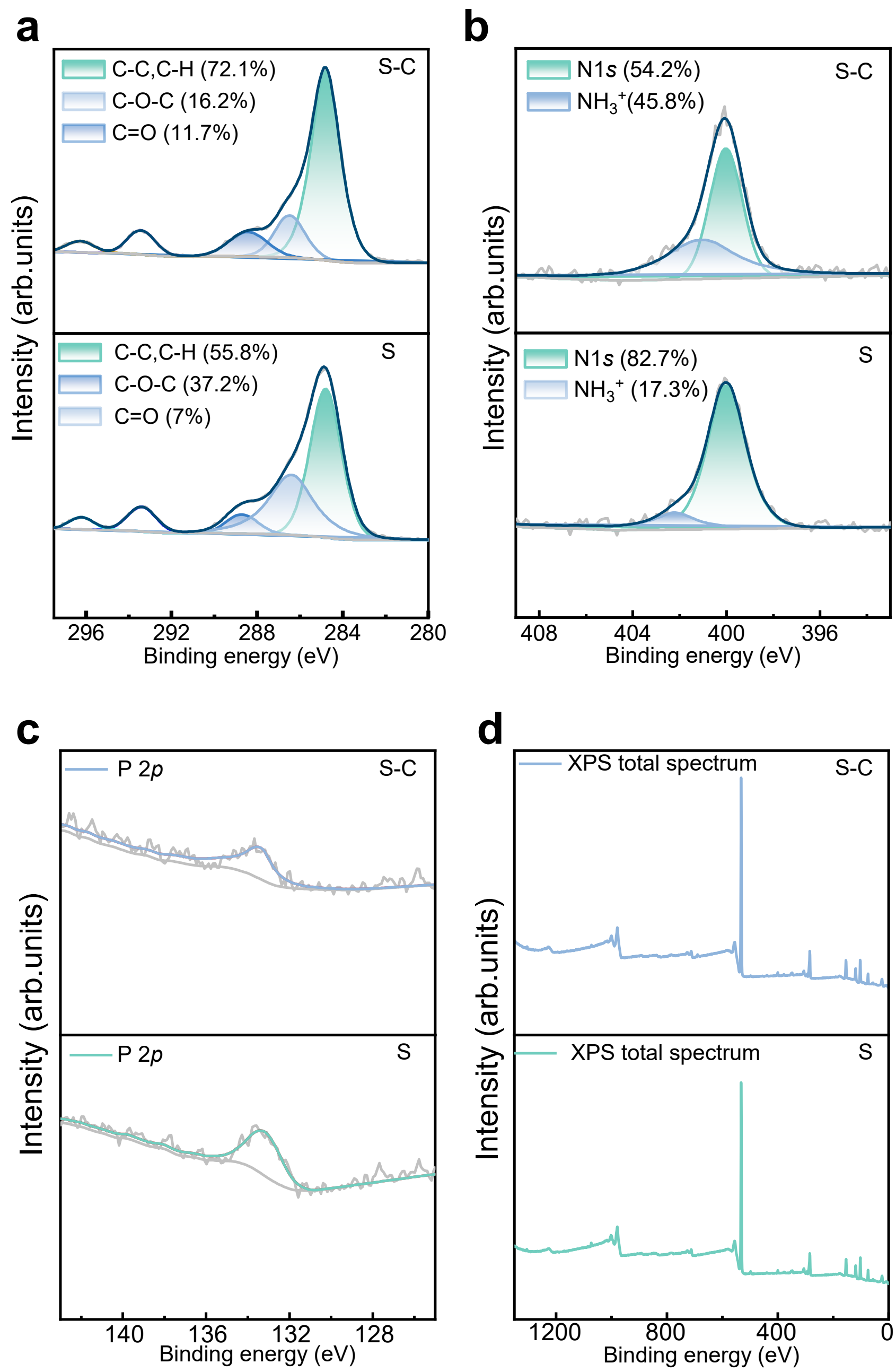

Supplementary Fig.23 XPS C, N, and P spectra and total elemental spectra of S and S-C. a Comparison of C 1*s* XPS spectra. **b** Comparison of N 1*s* XPS spectra. **c** Comparison of P 2*p* XPS spectra. **d** Comparison of total XPS spectra. Source data are provided as a Source Data file.

Note: Compared with S, S-C exhibits reduced C–O–C intensity (organic carbon) but increased C–C signal intensity (inorganic carbon) (Supplementary Fig. 23a). S-C also exhibits a reduced N 1*s* intensity (organic nitrogen) but an increased NH_3^+ signal

(inorganic ammonium) compared with S (Supplementary Fig. 23b), indicating a decrease in organic matter content. Furthermore, the P 2*p* spectrum of S-C (Supplementary Fig. 23c) shows an attenuated peak intensity compared with that of S, indicating substantial organic matter reduction on modified stone surfaces. Finally, no notable changes were observed in the elemental composition (Supplementary Fig. 23d).

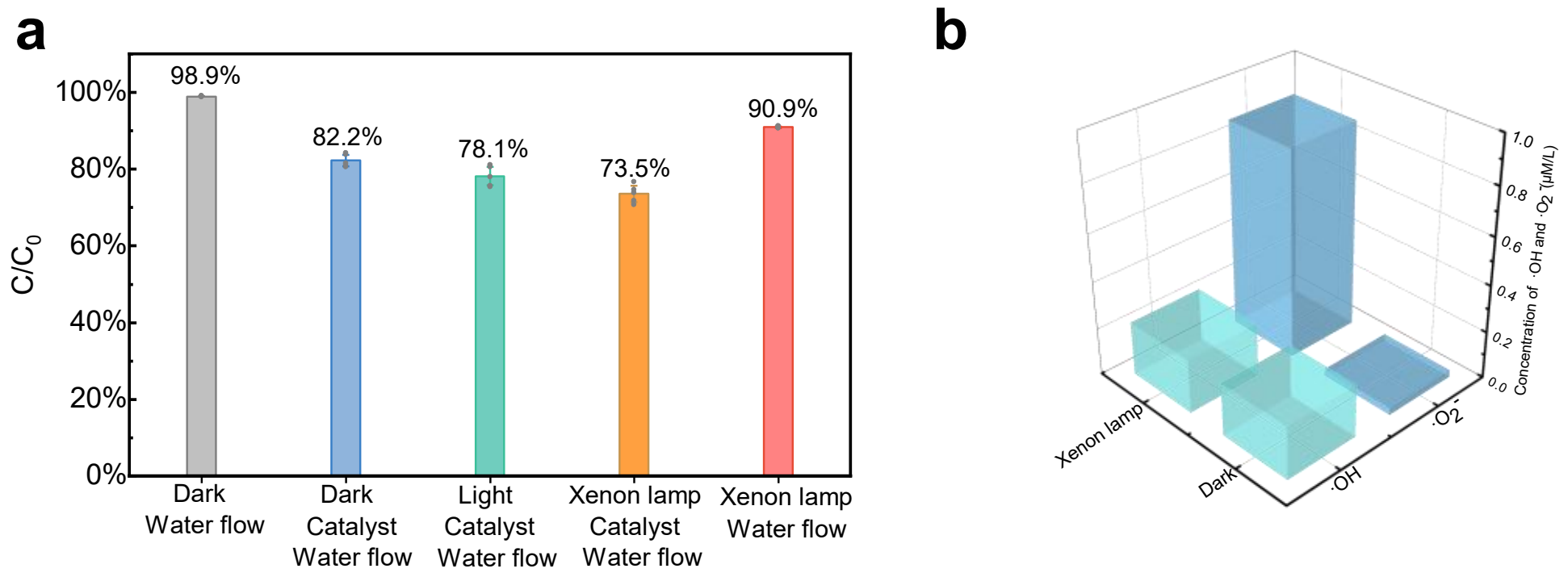

Supplementary Fig.24 The control experiment with xenon lamp irradiation. a MO degradation in no-catalyst controls under xenon lamp and dark conditions; MO degradation by catalyst-containing systems under flow conditions with and without xenon lamp irradiation (dark, natural light, and xenon lamp light). Error bars represent standard deviation based on five independent replicates. **b** Quantitative experiments of free radicals with or without xenon lamp irradiation. Source data are provided as a Source Data file.

Supplementary Table.1 Chemical structures of products formed during CEC degradation of MO and identified by mass spectrometry.

m/z	174	276	290	293
Degradation Products	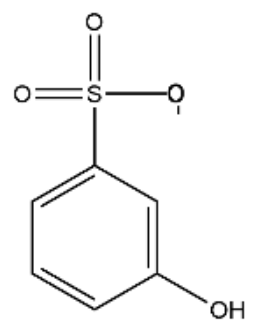 H^+	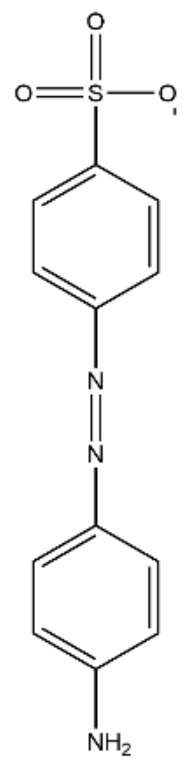	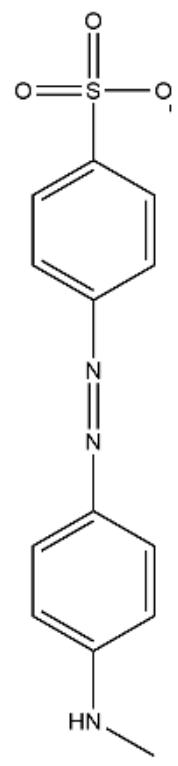	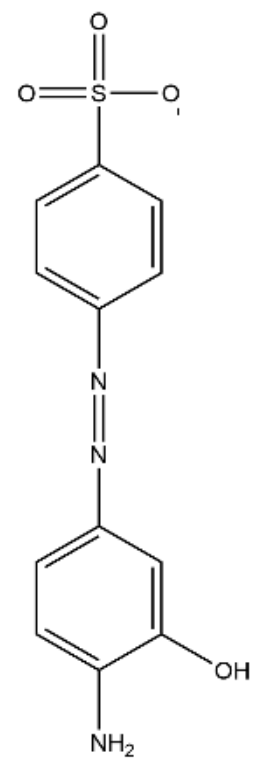 H^+

Supplementary References

1. Perdew JP, Burke K, Ernzerhof M. Generalized gradient approximation made simple. *Phys. Rev. Lett* **77**, 3865 (1996).

2. Payne MC, Teter MP, Allan DC, Arias TA, Joannopoulos JD. Iterative minimization techniques for ab initio total-energy calculations: molecular dynamics and conjugate gradients. *Rev. Mod. Phys* **64**, 1045 (1992).

3. Gieleciak R, Magdziarz T, Bak A, Polanski J. Modeling Robust QSAR. 1. Coding Molecules in 3D-QSAR from a Point to Surface Sectors and Molecular Volumes. *J. Chem. Inf. Model* **45**, 1447-1455 (2005).

4. Delley B. From molecules to solids with the $DMol^3$ approach. *J. Chem. Phys* **113**, 7756-7764 (2000).

5. Delley B. An all-electron numerical method for solving the local density functional for polyatomic molecules. *J. Chem. Phys* **92**, 508-517 (1990).

6. Dolg M, Wedig U, Stoll H, Preuss H. Energy-adjusted ab initio pseudopotentials for the first row transition elements. *J. Chem. Phys* **86**, 866-872 (1987).

7. Bergner A, Dolg M, Küchle W, Stoll H, Preuß H. Ab initio energy-adjusted pseudopotentials for elements of groups 13–17. *Mol. Phys* **80**, 1431-1441 (2006).

8. Hasnip PJ, Pickard CJ. Electronic energy minimisation with ultrasoft pseudopotentials. *Comput. Phys. Commun* **174**, 24-29 (2006).

9. Cao D-Q*, et al.* Contact-electro-catalysis for degradation of trace antibiotics in wastewater. *Chem. Eng. J* **487**, 150531 (2024).

10. Chen Z, Lu Y, Liu X, Li J, Liu Q. Novel magnetic catalysts for organic pollutant degradation via contact electro-catalysis. *Nano Energy* **108**, 108198 (2023).

11. Kıpçak İ, Kurtaran Ersal E, Özdemir M. Adsorptive Removal Of Ni^{2+} Ions From Aqueous Solutions by Nodular Sepiolite (Meerschaum) and Industrial Sepiolite Samples From Eskişehir, Turkey. *Clays Clay Miner* **68**, 220-236 (2024).

12. Sung G, Park JJ, Ha C-S. Fluorosilica/sepiolite hybrids for hydrophobic coating. *Compos. Interfaces* **19**, 547-556 (2013).

13. Jones J E. On the determination of molecular fields. II. From the equation of state of gas. *Proc. R. Soc. Lond. A* **106**, 463-477 (1924).

14. Jones JE. On the determination of molecular fields.—I. From the variation of the viscosity of a gas with temperature. *Proc. R. Soc. Lond. A* **106**, 441-462 (1924).

15. Schwerdtfeger P, Wales DJ. 100 Years of the Lennard-Jones Potential. *J. Chem. Theory Comput* **20**, 3379-3405 (2024).

16. Mourits FM, Rummens FHA. A critical evaluation of Lennard–Jones and Stockmayer potential parameters and of some correlation methods. *Can. J. Chem* **55**, 3007-3020 (1977).

17. Halkiadakis EA, Bowrey RG. The estimation of molecular parameters for the stockmayer (12-6-3) potential using critical properties. *Chem. Eng. Sci* **30**, 53-60 (1975).

18. Buckingham RA. The classical equation of state of gaseous helium, neon and argon. *Proc. R. Soc. Lond. A* **168**, 264-283 (1938).

19. Paşca D, Stoica C, Valls C. Anisotropic two-body problem under the Buckingham potential. *J. Math. Chem* **59**, 1368-1377 (2021).

20. Migliorati V, Serva A, Terenzio FM, D'Angelo P. Development of Lennard-Jones and Buckingham Potentials for Lanthanoid Ions in Water. *Inorganic Chemistry* **56**, 6214-6224 (2017).

21. Bethe HA, Bacher RF. Nuclear Physics A. Stationary States of Nuclei. *Rev. Mod. Phys* **8**, 82-229 (1936).

22. Parida SK, Dash S, Patel S, Mishra BK. Adsorption of organic molecules on silica surface. *Adv. Colloid Interface Sci* **121**, 77-110 (2006).

23. Song WZ*, et al.* Insulator polymers achieve efficient catalysis under visible light due to contact electrification. *Water Res* **226**, 119242 (2022).

24. Zhang M*, et al.* Rotation-mode liquid-solid triboelectric nanogenerator for efficient contact-electro-catalysis and adsorption. *Nano Energy* **110**, 108329 (2023).

25. Dong F*, et al.* Advanced Triboelectric Materials for Contact Electrocatalytic Degradation of Pollutants. *Small* **21**, 2500369 (2025).

26. Cao T*, et al.* H_2O_2 generation enhancement by ultrasonic nebulisation with a zinc layer for spray disinfection. *Chem. Eng. J* **431**, 134005 (2022).

27. Han B*, et al.* Durable and anti-corrosion superhydrophobic coating with bistratal structure prepared by ambient curing. *Prog. Org. Coat* **149**, 105922 (2020).
