# Supplementary material for: Hydrophobicity strategy drives contact-electro-catalysis on natural silicate minerals
Source: Nat Commun. 2026 Aug 29;17:10303. doi: 10.1038/s41467-026-77246-0 (PMC13620137; doi:10.1038/s41467-026-77246-0)
Supplement: Supplementary file 2 [file 41467_2026_77246_MOESM2_ESM.pdf]

Description of Additional Supplementary Files

Supplementary Data 1: Optimized atomic coordinates of Olivine-6H_2O model in XYZ format.

Supplementary Data 2: Optimized atomic coordinates of Olivine-POTS-6H_2O in XYZ format.

Supplementary Data 3: Optimized atomic coordinates of Olivine-TMCS-6H_2O in XYZ format.

Supplementary Data 4: Optimized atomic coordinates of Olivine-POTS-Al^{3+}-6H_2O model in XYZ format.

Supplementary Data 5: Optimized atomic coordinates of Glycerol-6H_2O model in XYZ format.

Supplementary Data 6: Optimized atomic coordinates of POTS-6H_2O model in XYZ format.

Supplementary Data 7: Optimized atomic coordinates of TMCS-6H_2O model in XYZ format.

Supplementary Data 8: Optimized atomic coordinates of sepiolite model in XYZ format.

Supplementary Data 9: Optimized atomic coordinates of sepiolite-POTS model in XYZ format.

Supplementary Data 10: Optimized atomic coordinates of sepiolite-TMCS model in XYZ format.
