# Supplementary material for: Hydrophobicity strategy drives contact-electro-catalysis on natural silicate minerals
Source: Nat Commun. 2026 Aug 29;17:10303. doi: 10.1038/s41467-026-77246-0 (PMC13620137; doi:10.1038/s41467-026-77246-0)
Supplement: Supplementary file 4 [file 41467_2026_77246_MOESM5_ESM.pdf]

nature portfolio

Peer Review File

Hydrophobicity Strategy Drives Contact-Electro-Catalysis on Natural Silicate Minerals

Corresponding Author: Dr Wangshu Tong

This file contains all reviewer reports in order by version, followed by all author rebuttals in order by version.

Version 0:

Reviewer comments:

Reviewer #1

(Remarks to the Author)
Sheng and colleagues report an approach using alkylation-based hydrophobic modification of natural minerals to develop fluorine-free contact-electro-catalysts. The study introduces a compelling mechanism suggesting that "moderate hydrophobicity" outperforms "super-hydrophobicity" and explores the practical potential of water-flow-driven CEC for ecological remediation. However, several critical issues must be addressed to strengthen the manuscript. The manuscript can be reconsidered only if the authors can resolve the following concerns:
1. The authors state in the abstract that this research provides insights into the regulation of biogeochemical cycles. Please clarify the specific mechanisms or pathways through which this CEC process influences biogeochemical regulation.
2. Alkylation is the focal point of this study; however, the mechanism illustrated in Figure 1 is based on fluorine-based modification. Is there a fundamental difference in the CEC mechanism between -F and -CH3 functional groups? The authors should align the schematic with their primary experimental focus.
3. Since CEC generates H2O2 and the natural minerals contain transition metals like Fe and Mn, could a Fenton-like reaction be induced? The authors need to decouple the ROS generation mechanism of CEC from potential metal-catalyzed background reactions.
4. In circulating water flow experiments, H2O2 was below detection limits, yet MO was successfully degraded. Is this contradictory? Please provide evidence or a theoretical explanation for ROS generation under such weak-force driving conditions.
5. In the study of H2O2 production, the friction between the magnetic stir bar and the mineral may contribute to the CEC signal. To exclude this potential artifact, the authors should conduct control experiments using mechanical stirring or placing the magnetic stir bar.
6. Under practical conditions, would the ROS or the modified mineral surfaces themselves exert toxic effects on beneficial microorganisms?
7. Microbial attachment varies significantly between hydrophilic and hydrophobic surfaces. How did the authors distinguish the biological response driven by CEC-generated ROS from the physical selection/attachment effects caused by the alkylated -CH3 surfaces?
8. For better readability, the "Materials and Methods" section should include subheadings such as "Sample Preparation," "H2O2Synthesis Quantification," and "ROS Detection"
9. The reference list contains numerous errors, including incomplete author lists, incorrect sub/superscripts in chemical formulas, erroneous journal titles, and missing page numbers. A thorough proofreading is required.
10. In Figure 1d, the unit "μMol" should be corrected to the standard lowercase "μmol"

Reviewer #2

(Remarks to the Author)
This work explores the use of non-fluorinated and hydrophobically modified natural minerals as catalysts for contact-electro-catalysis (CEC), and replaces the commonly used ultrasonic input with simulated river flow, thereby broadening potential application scenarios. The topic is interesting and potentially meaningful. The manuscript also attempts to rationalize CEC behavior in terms of catalyst hydrophobicity and electronegativity, but some interpretations are not yet sufficiently supported. In addition, the writing and organization lack clarity in places, and the connection between experimental data and analysis is sometimes weak, which may lead to reader confusion. Several mechanistic arguments also require stronger evidence and

more rigorous discussion. Overall, I believe the manuscript could be suitable for publication after major revision.
1. The authors chose to show data for only 5 of the 10 natural silicate mineral samples in multiple places where they should have shown all 10 (e.g., Fig. 1b and 1c, Supplementary Fig. 3, Supplementary Fig. 20, 21), which is very detrimental to the derivation of conclusions.
2. The manuscript mentions that "moderate hydrophobicity" is the key, is this a universal rule or specific to sepiolite, as the manuscript only provides data on the "hydrophobicity-CEC activity" relationship for sepiolite-F (Fig. 1f and Supplementary Fig. 15). Given the repeated emphasis on the critical role of hydrophobicity in both the paper's title and main text, it is recommended that the "hydrophobicity-CEC activity" relationship curves for the other nine silicate minerals be supplemented.
3. The author's explanation for the different effects of H2O2 production via CEC after fluorination of different silicate minerals (Fig. 1d) is confusing. First, different degrees of -F modification result from different silica-to-oxygen ratios. How the degree of -F modification was determined; the authors did not determine the F content of the different fluorinated silicate minerals. Then, different amounts of reactive oxygen atoms were exposed on different crystalline surfaces at the same silicon to oxygen ratio. How were the active oxygen exposure areas between spodumene and wollastonite calculated in Fig. 1e? Were the exposed crystal faces of spodumene and wollastonite in the inset determined by any assay? Furthermore, with a nearly 1-fold difference in silica-oxygen ratios, does the crystal surface exposure really result in such a large degree of -F modification, which brings about a difference in activity of several times? All of these are very important to whether the hydrophobic strategies highlighted in the title are universal. They should not be studied and recounted in such a generalized manner.
4. DFT calculations reveal that, at equivalent distances between the catalyst and H_2O molecules, the electron transfer amount at M-C (0.081 e at 1.8 Å) is markedly higher than that at M-F (0.019 e at 1.8 Å) (Fig. 1d). Does this contradict the stronger electronegativity of M-F (Fig. 2e)? Have computational models adequately accounted for the structure of water clusters at the actual interface, the dynamic conformations of modifying groups, and the influence of the double electric layer? Can this substantial discrepancy be fully explained by "contact propensity" alone?
5. In experiments involving the degradation of MO in circulating water flow, S-C and S-F achieved significant degradation rates (77%, 69%) (Fig. 3c). The text mentions "negligible adsorption", yet Supplementary Fig. 24 explicitly states that removal of cationic dyes "is primarily attributed to adsorption". Why does the reaction mechanism differ for MO, which is also a cationic dye? And how can the contributions of CEC degradation and adsorption be quantitatively distinguished? Furthermore, Supplementary Fig. 23 and 24 only provide data for S-F, with data for S and S-C missing. This cannot demonstrate that S and S-C share the same degradation mechanism as S-F. Regarding the results in Fig. 3c, the authors claim that hydrophobicity exerts a more pronounced effect under weak mechanical stimulation, thereby conferring superior CEC performance to S-C. However, this finding is at odds with the observation that M-F exhibits greater hydrophobicity than M-C (Fig. 1b).
6. The author attributes the improvement in Olivine-F performance to the addition of more -F groups, and this needs to be supported by experimental evidence.
7. Figure 2d-h: The author conducted a detailed analysis of the relationship between the catalyst's acquisition of electrons and its hydrophilicity and electronegativity during the WOR process, but failed to analyze the relationship between the catalyst's loss of electrons and these factors during the ORR process. There is a lack of evidence.
8. The article provides new ideas for designing CEC catalysts that are non-fluorinated, green, and suitable for natural environments. However, the manuscript makes extensive use of M-F research data and lacks M-C research data, including but not limited to: Fig. 1d, 1f, and 1g, Supplementary Fig. 6-14, and Supplementary Fig. 20.
9. Please add XPS data for Fe2p before and after fluorination in Supplementary Fig. 7. In addition, for Supplementary Fig. 11a, what is the reason for the significant change in the ratio of -CF2, -CF3 signal peaks located near 292, 294 eV before and after the reaction?
10. Fig. 1g and SI Fig. 17: How are ·OH and ·O2- quantified? And how can the conclusion "•OH plays a more significant role than •O2- in the formation of H2O2" be drawn based on the results of free radical capture?
11. SI Fig. 10: Why is the effect of the first cycle significantly worse than that of the subsequent cycles? Please explain.
12. Compared with the previously published works that produce H2O2 through CEC, the performance of H2O2 production in this study is not outstanding. Moreover, fluorine-containing materials such as PTFE and FEP have high stability and can be used for catalysis for a long time without causing excessive environmental pressure. The authors should more clearly elaborate on the uniqueness and innovation of the non-fluorine catalyst system in this work.

Reviewer #3

(Remarks to the Author)
This manuscript reports a systematic investigation of how interfacial hydrophobicity governs contact-electro-catalysis (CEC) on natural silicate minerals. By introducing alkyl ($-CH_3$) surface groups, the authors demonstrate that moderate hydrophobicity maximizes hydrogen peroxide generation and ROS formation, outperforming both hydrophilic and fluorinated mineral surfaces. The work combines mineral screening, surface chemistry, theoretical analysis, density functional theory, weak-force flow experiments, and a rare field-scale deployment in a natural river, including genome-resolved metagenomic analysis of surface-associated microbial communities. The study addresses an important sustainability challenge in CEC by reducing reliance on fluorinated polymers and provides a conceptual framework for catalyst design under weak mechanical excitation.

Below are some comments in detail:
1. While the field deployment and metagenomic results are compelling, claims related to "ecological restoration" and ecosystem-scale regulation are not fully supported. The data demonstrate microbial selection and organic matter attenuation, but not ecosystem recovery. The authors should temper these claims throughout the Abstract, Discussion, and

Conclusion.

2. Comparisons between ultrasonication and flow-driven CEC rely on relative performance metrics without rigorous normalization of mechanical energy input. A clearer discussion or estimation of energy densities and force regimes is required to substantiate claims regarding weak-force efficiency.

3. The DFT calculations treat single water molecules interacting with mineral surfaces, whereas real CEC interfaces involve hydrated clusters and dynamic water networks. These limitations should be explicitly acknowledged in the Discussion, or need some further work to strengthen this part.

4. Statistical treatment (error bars, replicate numbers) should be clarified for key figures, particularly Figs. 1d and 3c.

5. The influence of ionic strength and cation adsorption on zeta potential and hydrophobicity could be discussed more clearly.

6. The Introduction is somewhat lengthy and repetitive; streamlining would improve readability.

7. Several grammatical and stylistic issues should be corrected during revision.

Version 1:

Reviewer comments:

Reviewer #1

(Remarks to the Author)
Authors have almost replied to the questions.

Reviewer #2

(Remarks to the Author)
The authors have carried out a thorough and professional revision that satisfactorily addresses nearly all of the major concerns raised in the original review. The manuscript has been significantly improved in clarity, logical flow, data completeness, and mechanistic rigor. The addition of new XPS data, expanded mineral datasets, DFT calculations on water clusters, energy density comparisons, and careful tempering of ecological claims have strengthened the paper considerably. I am now happy to recommend acceptance.

Reviewer #3

(Remarks to the Author)
The manuscript has been substantially improved and many textual/presentation comments were addressed. However, several mechanistic and control-related reviewer concerns remain only partially resolved, especially Fenton/metal-background chemistry, flow-driven ROS evidence, and light/adsorption controls for MO degradation. Below are some comments in detail:

1, The flow-driven MO degradation experiment needs special attention. The Methods state that MO degradation was conducted “under xenon lamp irradiation.” If that is correct, then the authors must include no-catalyst light controls, dark flow controls, catalyst-without-flow controls, and possibly radical scavenger controls under the same illumination. If the xenon lamp statement is a leftover error, it should be removed immediately.

2, The microbial interpretation is now more cautious, but still not fully resolved. The authors should not claim that ROS alone drives microbial restructuring. The current data support a combined effect of hydrophobic surface selection, altered organic matter adhesion, and possible ROS-mediated oxidative pressure.

3, The hydrophobicity universality claim should be moderated. The data support “broadly observed among the tested silicate minerals under these conditions,” not a universal rule across natural minerals or all hydrodynamic regimes.

4, The abstract also appears to be about 190 words, while the Nature Communications formatting instructions and checklist specify an abstract limit of 150 words or fewer.

Version 2:

Reviewer comments:

Reviewer #3

(Remarks to the Author)
I went through the revised version and the response. I am quite happy with the answers and the revised manuscript. I think it can be accepted for publication now.

Open Access This Peer Review File is licensed under a Creative Commons Attribution 4.0 International License, which permits use, sharing, adaptation, distribution and reproduction in any medium or format, as long as you give appropriate credit to the original author(s) and the source, provide a link to the Creative Commons license, and indicate if changes were made.
In cases where reviewers are anonymous, credit should be given to 'Anonymous Referee' and the source.
The images or other third party material in this Peer Review File are included in the article's Creative Commons license, unless indicated otherwise in a credit line to the material. If material is not included in the article's Creative Commons license and your intended use is not permitted by statutory regulation or exceeds the permitted use, you will need to obtain permission directly from the copyright holder.
To view a copy of this license, visit https://creativecommons.org/licenses/by/4.0/

Reviewer #1: Sheng and colleagues report an approach using alkylation-based hydrophobic modification of natural minerals to develop fluorine-free contact-electro-catalysts. The study introduces a compelling mechanism suggesting that "moderate hydrophobicity" outperforms "super-hydrophobicity" and explores the practical potential of water-flow-driven CEC for ecological remediation. However, several critical issues must be addressed to strengthen the manuscript. The manuscript can be reconsidered only if the authors can resolve the following concerns:

Thank you for your insightful and constructive comments on our manuscript. We have carefully revised the manuscript to clarify the logical relationships among several datasets and to restructure parts of the text for greater accuracy and readability. Detailed point-by-point responses are provided below. It is hoped that these revisions adequately address the concerns raised.

Comment 1: The authors state in the abstract that this research provides insights into the regulation of biogeochemical cycles. Please clarify the specific mechanisms or pathways through which this CEC process influences biogeochemical regulation.

Reply: We appreciate this important comment. In the revised manuscript, the potential mechanisms by which **sustained water-flow-driven CEC may contribute to localized biogeochemical transformations at mineral-water interfaces** have been clarified and more explicitly described.

First, CEC-active surfaces may **directly** alter interfacial biogeochemical transformations through ROS-mediated oxidation processes and surface-dependent adsorption effects [1–3]. In our laboratory experiments, LC-MS detected low-molecular-weight oxidation products in solution (Fig. 3d and Supplementary Tab. 1), suggesting partial oxidation of organic matter at or near the mineral-water interface. These oxidized intermediates are likely to be more bioavailable and may influence carbon turnover by facilitating subsequent microbial utilization. Furthermore, XPS analysis showed shifts in surface-associated C- and N-containing functional groups after surface modification, which is consistent with altered organic matter chemistry. Sustained ROS generation at mineral-water interfaces may also modify localized redox

microenvironments, thereby affecting the transformation pathways of redox-sensitive compounds.

Second, CEC-active surfaces may **indirectly** influence localized biogeochemical transformations processes by reshaping surface-associated microbial communities [1, 3–4]. Metagenomic analysis indicated that modified mineral surfaces selectively enriched ROS-tolerant and metabolically versatile microorganisms. In particular, enriched taxa encoded complete ROS detoxification systems and metabolic pathways for degrading oxidized low-molecular-weight substrates including formate, acetate, acetaldehyde, and oligosaccharides, as well as more complex polymers such as cellulose and chitin (Fig. 4). These compositional shifts imply potential alterations in microbial carbon processing pathways. In addition, variations were observed in MAGs carrying genes involved in nitrogen and sulfur transformations. For example, MAGs harboring DNRA-related genes (e.g., nrfADH) were enriched, whereas MAGs associated with dissimilatory sulfate reduction genes (e.g., aprA and sat) exhibited decreased relative abundance. Although these genomic results do not directly quantify process rates, they indicate that CEC-driven surface reactions may influence the functional potential for localized carbon, nitrogen, and sulfur transformations. In addition to microbial community restructuring, our previous studies suggest that modifications in mineral surface properties (e.g., surface area, charge, reactivity, and ROS generation) and oxidative conditions may alter the adsorption behavior and activity of extracellular enzymes catalyzing the organic matter decomposition, thereby representing another indirect pathway through which CEC may affect localized elemental transformations [5–6].

The Abstract and Discussion sections have been revised accordingly to explicitly describe these mechanistic pathways and to avoid overstating the scope of the findings.

Page 2, lines 35-39: “Together, these findings establish hydrophobicity regulation as an effective contact-optimization strategy for CEC, providing a basis for non-fluorinated catalyst design and for harnessing naturally available mechanical energy in environmentally relevant interfacial processes.”

Pages 4-5, lines 86-91: "We further demonstrate that this strategy is effective under weak-force and natural hydrodynamic conditions, where it reshapes interfacial chemistry and surface-associated microbial communities. These findings establish hydrophobicity regulation as a contact-optimization principle for CEC and broaden its relevance from fluorinated laboratory systems to environmentally relevant mineral-water interfaces."

References:

[1] Dong H, Zeng Q, Sheng Y, Chen C, Yu G, Kappler A. Coupled iron cycling and organic matter transformation across redox interfaces. *Nat. Rev. Earth Environ* ***4***, 659-673 (2023).

[2] Kleber M, Bourg I.C, Coward E.K, Hansel C.M, Myneni S.C, Nunan N. Dynamic interactions at the mineral–organic matter interface. *Nat. Rev. Earth Environ* ***2***, 402-421(2021).

[3] Kappler A, Bryce C, Mansor M, Lueder U, Byrne J.M, Swanner E.D. An evolving view on biogeochemical cycling of iron. *Nat. Rev. Microbiol* ***19***, 360-374 (2021).

[4] Zhang P, et al. Production and significance of Reactive Oxygen Species in the subsurface. *Earth Sci. Rev* **23**, 105230 (2025).

[5] Sheng Y, Hu J, Kukkadapu R, Guo D, Zeng Q, Dong H. Inhibition of extracellular enzyme activity by reactive oxygen species upon oxygenation of reduced iron-bearing minerals. *Environ. Sci. Technol* ***57***, 3425-3433 (2023).

[6] Sheng Y, Dong H, Coffin E, Myrold D, Kleber M. The important role of enzyme adsorbing capacity of soil minerals in regulating β-glucosidase activity. *Geophys. Res. Lett* ***49***, e2021GL097556 (2022).

Comment 2: Alkylation is the focal point of this study; however, the mechanism illustrated in Figure 1 is based on fluorine-based modification. Is there a fundamental difference in the CEC mechanism between -F and $-CH_3$ functional groups? The authors should align the schematic with their primary experimental focus.

Reply: We thank the reviewer for this insightful comment. We agree that Fig. 1 should

more clearly reflect the main logic and experimental focus of this study. The central innovation of this work is the establishment of **a hydrophobicity-regulation strategy** for contact-electro-catalysis (CEC). Therefore, the core scientific question addressed in Fig. 1 concerns how modulation of surface wettability influences CEC performance. In this framework, both $–CH_3$ (–C) and –F functionalization are employed as approaches to regulate hydrophobicity, whereas the development of the non-fluorinated mineral catalyst M-C represents an additional advancement of the study.

To avoid potential confusion, Fig. 1 and the associated discussion have been revised to better reflect this logic. Fig. 1a-c illustrate the **consistent relationship** between moderate hydrophobicity and enhanced CEC performance across **different surface modification strategies**. Fig. 1d-e further demonstrate that this trend is maintained **across distinct mineral types**. The remaining panels (Fig. 1g-h) specifically focus on M-C, including its ROS generation behavior and proposed reaction pathway, as M-C constitutes the principal catalyst system developed in this work.

The hydrophobicity-regulation data of M-F were retained in Fig. 1d-f because they are critical for establishing the structure-performance relationship between hydrophobicity and CEC activity. Importantly, this comparison was conducted within a single modification platform to evaluate the effect of wettability on CEC while minimizing interference from differences in modifier chemistry. M-F was selected for this analysis because it exhibits the largest wettability variation before and after modification, thereby providing a broader and more controllable hydrophobicity window. This enables clearer observation of the influence of hydrophobicity on CEC performance. These results further indicate that excessive hydrophobicity is unfavorable for CEC, directly supporting the conclusion that moderate hydrophobicity is optimal. This also explains why M-C, with a more moderate hydrophobic character, outperforms the more strongly hydrophobic M-F.

In addition, the related text has been streamlined and further characterization of M-C has been incorporated into the Supporting Information to reinforce its role as the primary catalyst system. Specifically, the EPR spectra of M-C (Fig. R1-2) have been added in Fig. 1g, and the isotope-labeling experiment of M-C (Fig. R3) has been

included in Supplementary Fig. 12 to further substantiate the CEC mechanism of M-C. The corresponding schematic of the CEC mechanism for M-C (Fig. R4) has also been updated in Fig. 1h of the revised manuscript. Furthermore, Supplementary Fig. 4-14, 16-17 of the original Supporting Information, which mainly presented basic physicochemical characterization of M-F, have been removed to maintain focus on the central subject of this study, M-C.

Page 9, lines 175-178: "The free radicals and intermediates produced during the M-C CEC process were identified using electron paramagnetic resonance (EPR). Hydroxyl radicals (•OH), superoxide radicals ($•O_2^-$), and singlet oxygen ($^1O_2$), which is the product of $•O_2^-$, were detected under ultrasonic irradiation (Fig. 1g and Supplementary Fig. 11)."

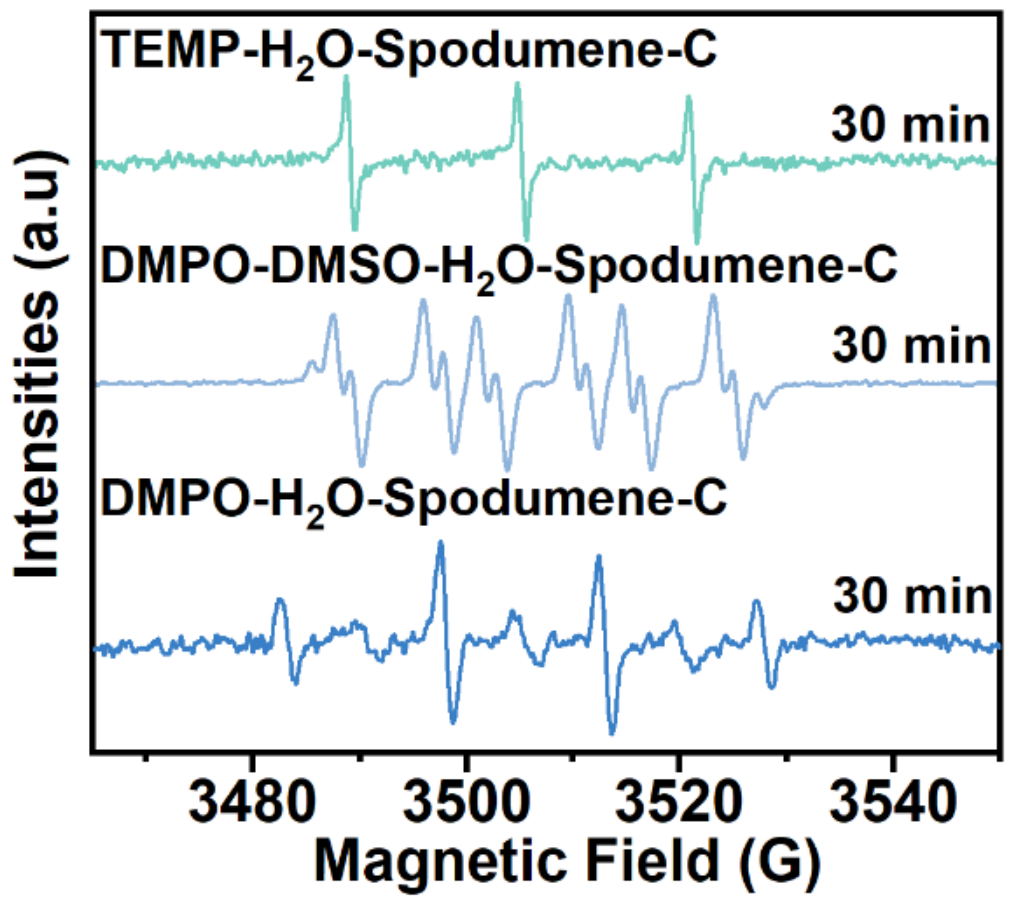

Fig. R1. Electron paramagnetic resonance (EPR) spectra of spodumene-C under ultrasonication for 30 min. (Fig. 1g)

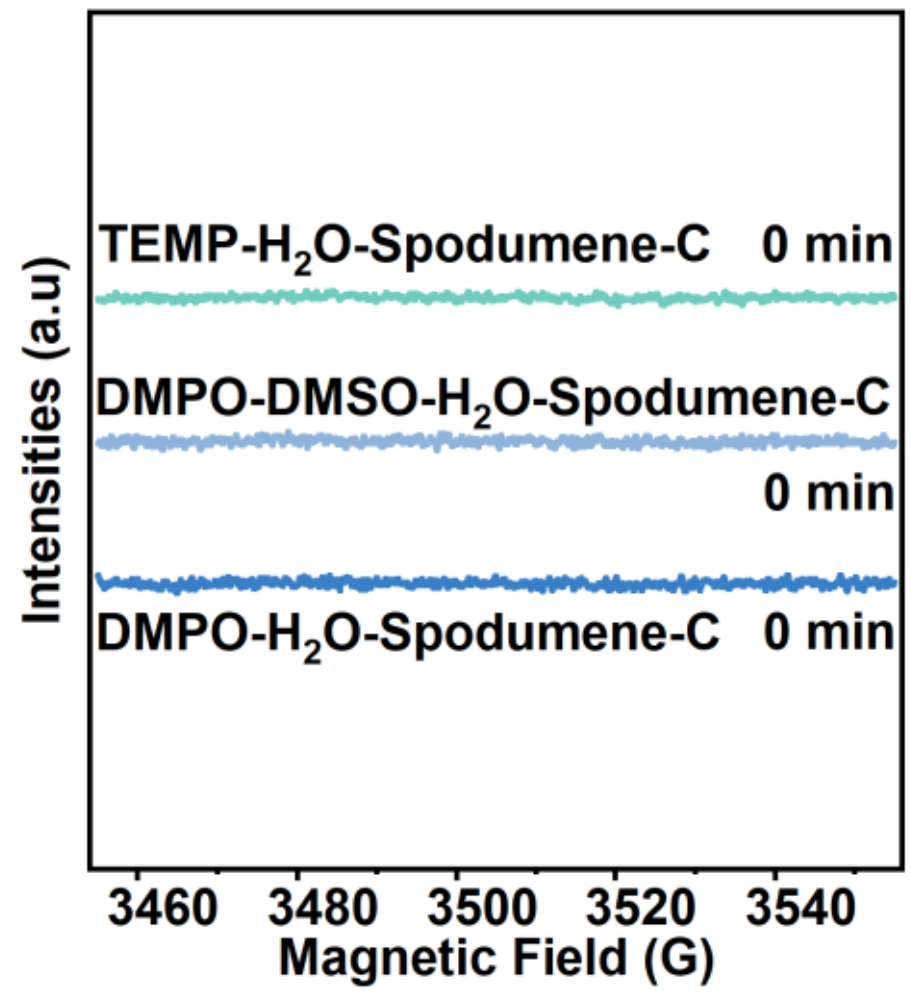

Fig. R2. EPR spectra of spodumene-C under ultrasonication for 0 min. (Supplementary Fig. 11)

Page 9, lines 178-184: "To further confirm the CEC pathway of M-C, after replacing the H_2O in the benzene acid aqueous solution with $H_2{}^{18}O$, the ^{18}O isotope labeling experiment was conducted in combination with liquid chromatography-mass spectrometry (LC-MS) to analyze the source of O in •OH. The ^{18}O-incorporated species were identified via the total ion chromatogram and mass spectra, demonstrating that •OH originates from H_2O, that is water oxidation reaction (WOR) of CEC (Supplementary Fig. 12a-b)[29]."

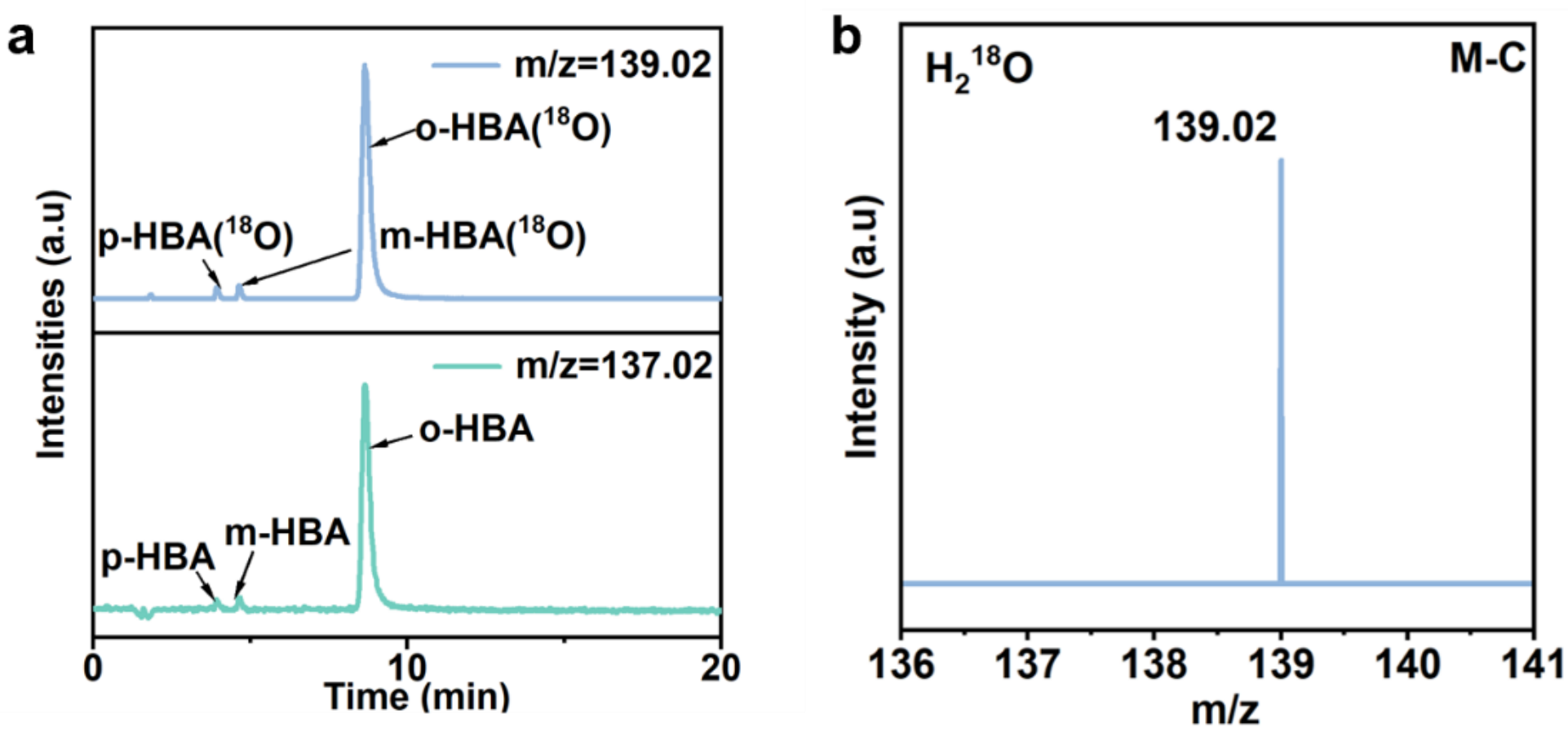

Fig. R3. Liquid chromatography-mass spectrometry (LC-MS) analysis. (a) Total ion chromatograms of the reactions of •^{16}OH and •^{18}OH with benzoic acid. (b) Mass spectra of the ^{18}O-labeled water after CEC of M-C obtained by LC–MS. (Supplementary Fig. 12)

Page 10, lines 189-193: "Thus, the following pathway for H_2O_2 production is proposed (Fig. 1h): The collapse of ultrasonic cavitation bubbles facilitates electron transfer between H_2O molecules and M-C, triggering H_2O oxidation to generate •OH (①), which subsequently forms H_2O_2. Moreover, the electrons acquired by M-C react with O_2 to generate $•O_2^-$ (②), which then undergoes superoxide dismutation to yield H_2O_2 (③)."

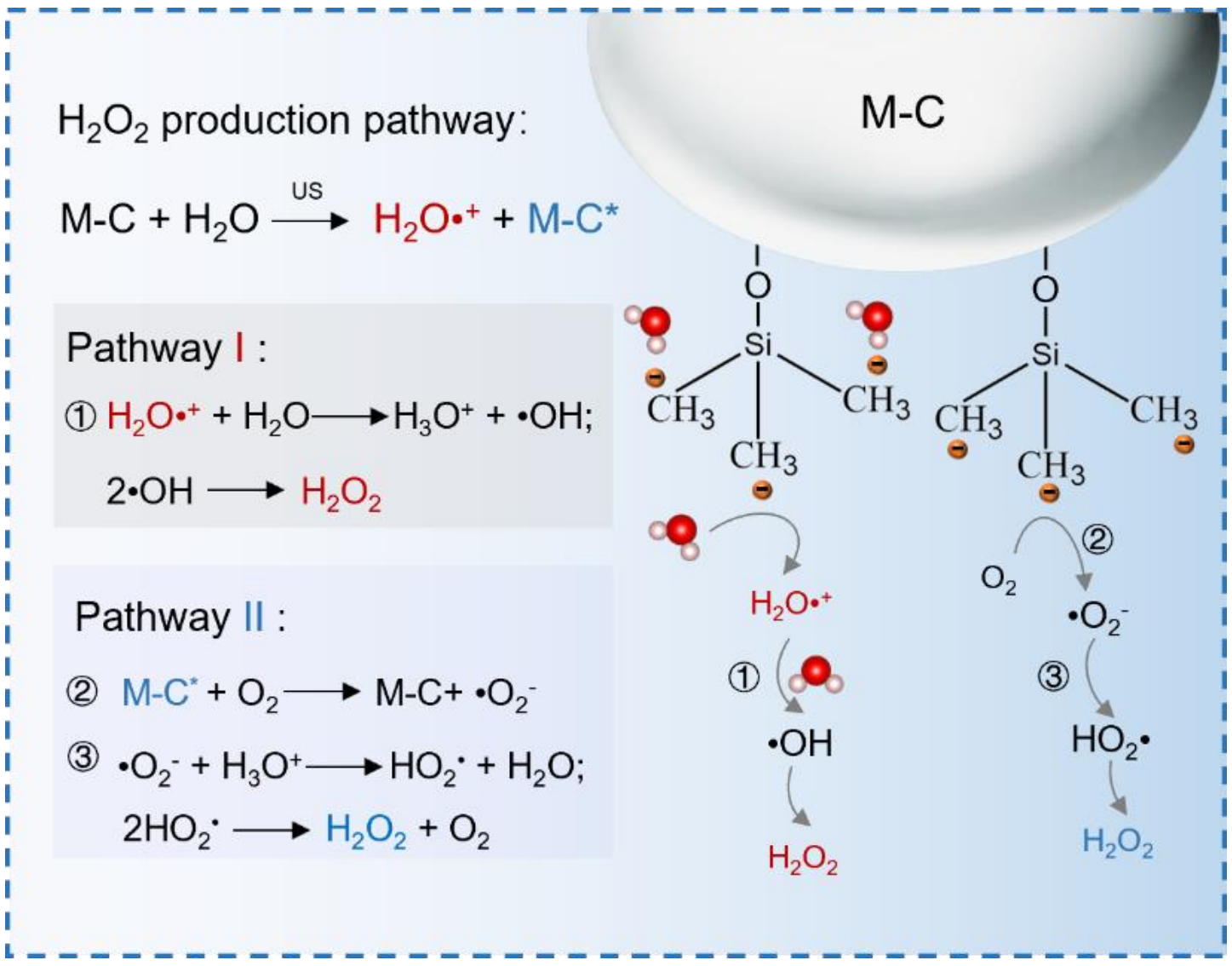

Fig. R4. Schematic illustration of ROS generation by M-C. (Fig. 1h)

Comment 3: Since CEC generates H_2O_2 and the natural minerals contain transition metals like Fe and Mn, could a Fenton-like reaction be induced? The authors need to decouple the ROS generation mechanism of CEC from potential metal-catalyzed background reactions.

Reply: We thank the reviewer for this important and insightful comment. We agree that, because CEC produces H_2O_2 and some natural minerals contain transition metals, a Fenton-like process is theoretically possible and should be considered carefully.

In our system, however, metal-catalyzed background reactions are expected to contribute only a limited ROS generation compared with the dominant CEC process. As noted by the reviewer, Fenton or Fenton-like reactions generally require accessible redox-active metal species. In our case, XPS analysis shows that the surface Fe content

of the representative fluorinated olivine sample is about 1% (Fig. R5). Similarly, the crystal sand used in the circulating water-flow experiments contains only about 1% surface Fe (Fig. R6). Furthermore, the Fe content of the other nine silicate minerals is much lower than that of olivine, and their Fe2p spectra do not exhibit any obvious characteristic peaks (Fig. R7). At such low surface concentrations, and with these metals predominantly incorporated within the silicate lattice rather than existing as freely dissolved ions, their capacity to drive substantial Fenton-like reactions is considered limited.

Although CEC-coupled Fenton systems have been reported in previous studies, those systems typically involved the deliberate addition of relatively high concentrations of Fe ions (1 mM) to enhance the conversion of H_2O_2 to •OH [1,2]. In contrast, no external Fe ions were introduced in the present experiments. Therefore, the experimental conditions differ fundamentally from those intentionally designed to promote strong Fenton chemistry.

Moreover, the primary performance metric evaluated in this study is **H_2O_2 production** during **CEC**. A Fenton-like reaction would **consume H_2O_2** to generate •OH. Consequently, even if a minor Fenton-like contribution occurred, it would not artificially inflate the measured H_2O_2 production. Instead, such a side reaction would tend to decrease the detectable H_2O_2 level.

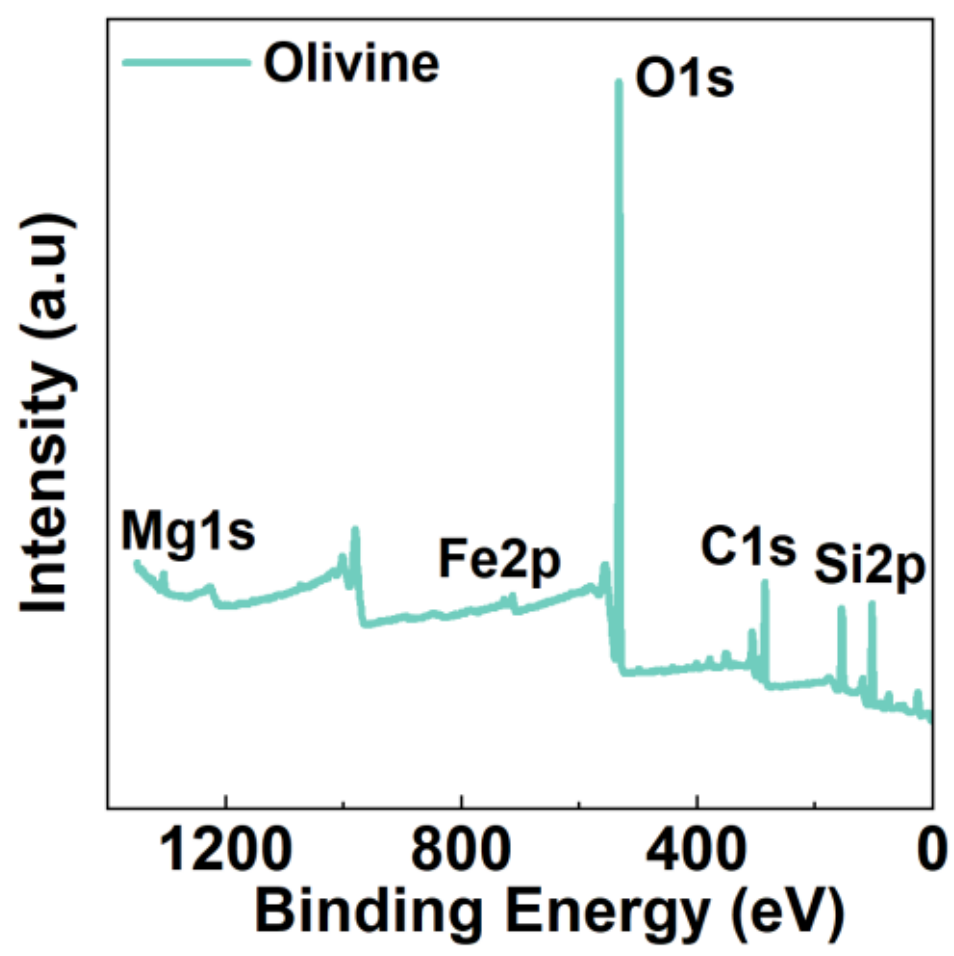

Fig. R5. The total element XPS spectrum of olivine mineral.

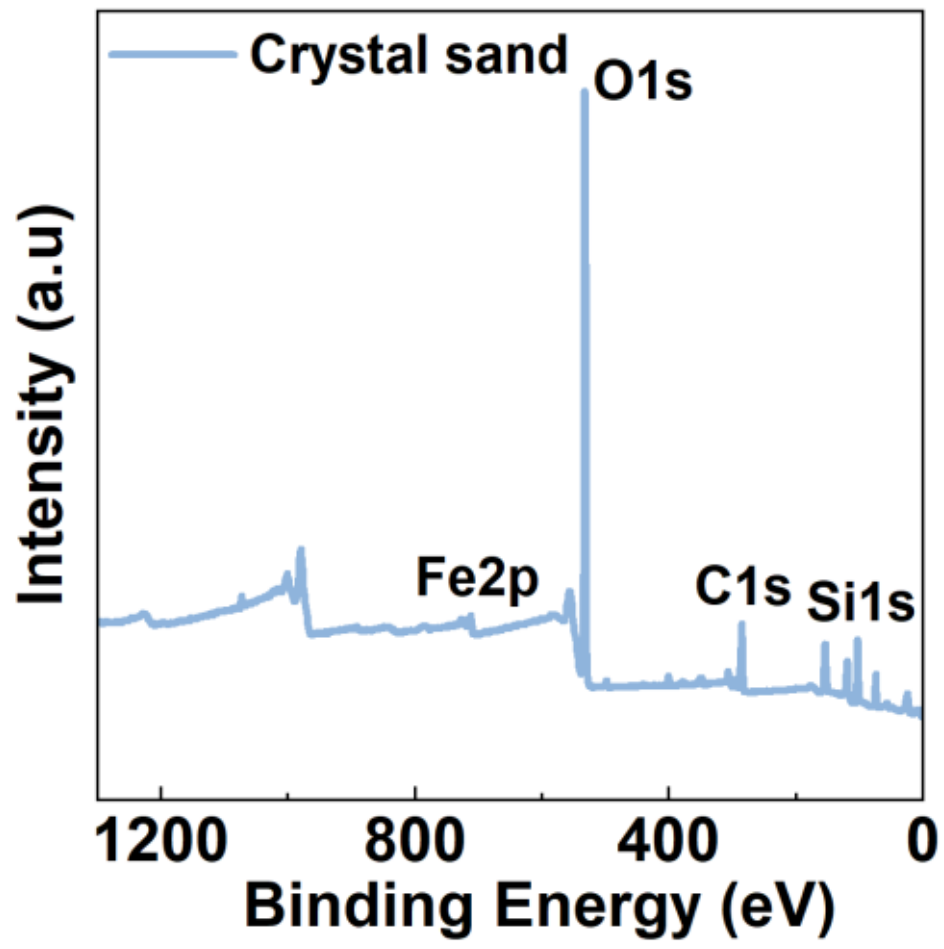

Fig. R6. The total element XPS spectrum of crystal sand.

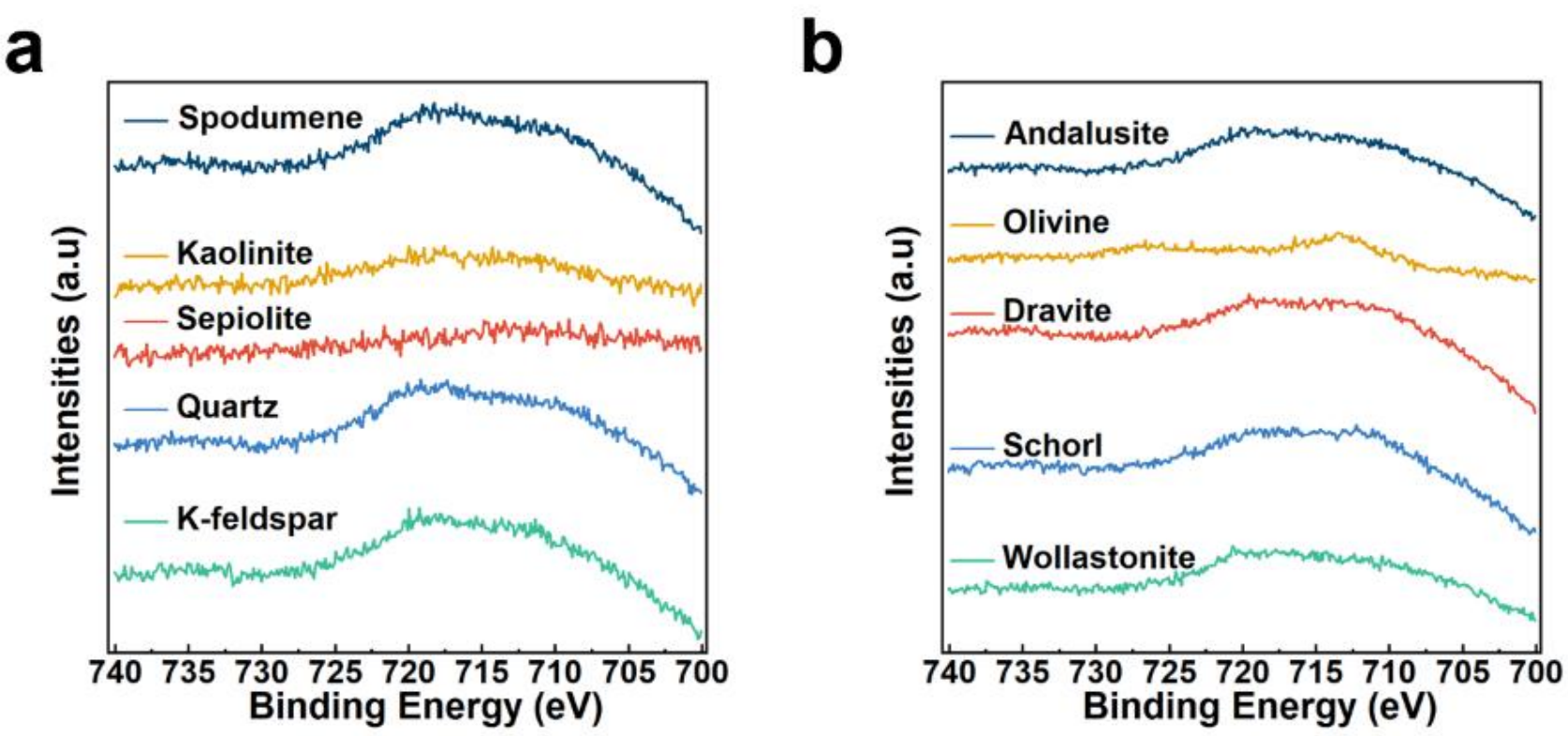

Fig. R7. The Fe 2p XPS spectra of original silicate minerals.

References:

[1] Ma D, et al. Fe III-driven self-cycled Fenton via contact-electro-catalysis for water purification. *npj Clean Water* **8**, 42 (2025).

[2] Li W, et al. Boosting Reactive Oxygen Species Generation via Contact-Electro-Catalysis with Fe III-Initiated Self-cycled Fenton System. *Angew. Chem. Int. Ed* **64**, e202413246 (2024).

Comment 4: In circulating water flow experiments, H_2O_2 was below detection limits, yet MO was successfully degraded. Is this contradictory? Please provide evidence or a theoretical explanation for ROS generation under such weak-force driving conditions.

Reply: We thank the reviewer for this insightful comment. We agree that the absence of detectable H_2O_2 under circulating water flow, despite observable MO degradation, requires further clarification. These results are not contradictory, but rather reflect the slow, low-flux, and dynamic characteristics of CEC under weak-force driving conditions.

First, **H_2O_2 is not a stable end product that necessarily accumulates to detectable levels in an open and continuously flowing system (100 h)**. Under weak hydrodynamic driving, ROS generation by CEC proceeds at a much lower rate than under ultrasonication, and thus prolonged accumulation would be required for H_2O_2 to approach the detection limit. However, during long-term circulation, the generated H_2O_2 can be continuously consumed through interfacial redox reactions, photochemical decay, or further conversion into other reactive oxygen species [1,2]. Consequently, direct quantification of H_2O_2 in the flowing system involves substantial uncertainty and may not accurately represent cumulative CEC activity. As shown in Fig. 3b, the negligible increase in H_2O_2 over several hours indicates that accumulation is difficult under these conditions.

Second, **MO degradation represents time-integrated response to ROS exposure**, whereas H_2O_2 measurement reflects only its instantaneous concentration at the sampling point. Even a very low steady-state ROS flux can progressively degrade MO during prolonged circulation, resulting in measurable pollutant transformation without detectable H_2O_2 accumulation. Therefore, MO degradation provides a more sensitive and practical indicator for evaluating CEC performance under weak-force conditions than direct H_2O_2 measurement.

Third, the natural river deployment experiment offers an additionally indirect evidence for ROS generation under environmentally relevant weak-force conditions (Fig. 4). The $-CH_3$-modified mineral surface (S-C) exhibited distinct interfacial chemical alterations and selectively enriched microorganisms with enhanced oxidative-stress-response capacity, including a higher relative abundance of **ROS-related genes**. Such microbial adaptation is consistent with sustained exposure to a mildly oxidative interfacial environment. Although ROS generation under natural water flow is slow and

spatially diffuse, making direct in situ H_2O_2 detection challenging, its cumulative effects can be recorded through **stable pollutant transformation products and microbial community adaptation**.

From a mechanistic standpoint, weak hydrodynamic forces such as water flow and droplet impact have been reported to induce liquid-solid contact electrification and associated redox reactions on dielectric surfaces, often evaluated through dye degradation as a proxy for CEC activity [3–6]. Thus, weak-force CEC should be regarded as a **slow but continuous interfacial process**, whose long-term cumulative outcomes are more readily captured than instantaneous ROS concentrations.

We have clarified this point in the revised manuscript as follows.

Page 17, lines 302-306: "Because H_2O_2 production under such weak-force conditions needs accumulate to be detected but is an unstable end product in an open and continuously flowing system for reliable direct quantification (Fig. 3b), the degradation of methyl orange (MO) was employed as a stable indicator to accumulate and reflect the catalytic performance of the CEC over time."

References:

[1] Rao Z, et al. Accelerated Photolysis of H_2O_2 at the Air–Water Interface of a Microdroplet. *J. Am. Chem. Soc* **145,** 24717 - 24723 (2023).

[2] Zhao X, Feng Y, Zhang B, Jin H, Wei X. Atomic insights into hydrogen peroxide decomposition on the surface of pure and pre-treated silver: A reactive molecular dynamics simulation study. *Appl. Surf. Sci* **644**, 158701 (2024).

[3] Wu Z, et al. Low-frequency contact-electro-catalysis driven by friction between the PTFE-coated fabric and dye solution. *J. Alloys Compd* **1010**, 177440 (2025).

[4] Zhang M, et al. Rotation-mode liquid-solid triboelectric nanogenerator for efficient contact-electro-catalysis and adsorption. *Nano Energy* **110**, 108329 (2023).

[5] Song WZ, et al. Insulator polymers achieve efficient catalysis under visible light due to contact electrification. *Water Res* **226**, 119242 (2022).

[6] Liu S, et al. Contact-Electro-Catalysis for Dye Degradation Using Polymer

Composites Fabricated under Cyclic Pulsating Pressure. *ACS Appl. Eng. Mater* **4**, 1238-1249 (2026).

Comment 5: In the study of H_2O_2 production, the friction between the magnetic stir bar and the mineral may contribute to the CEC signal. To exclude this potential artifact, the authors should conduct control experiments using mechanical stirring or placing the magnetic stir bar.

Reply: Thank you for your helpful suggestion. To evaluate the potential contribution of friction between the magnetic stir bar and the mineral, additional control experiments were conducted under magnetic stirring conditions.

As shown in Fig. R8, the average H_2O_2 production rate under magnetic stirring was low, about 13.5 μmol L^{-1} h^{-1}. By comparison, wollastonite, which exhibited the lowest CEC performance under ultrasonication, generated 3.7 times more H_2O_2 than this average value, whereas olivine, which showed the highest CEC performance under ultrasonication, produced nearly 20 times more H_2O_2. These results indicate that the contribution of friction between the magnetic stir bar and the modified silicate minerals to the observed CEC signal is minimal under magnetic stirring conditions.

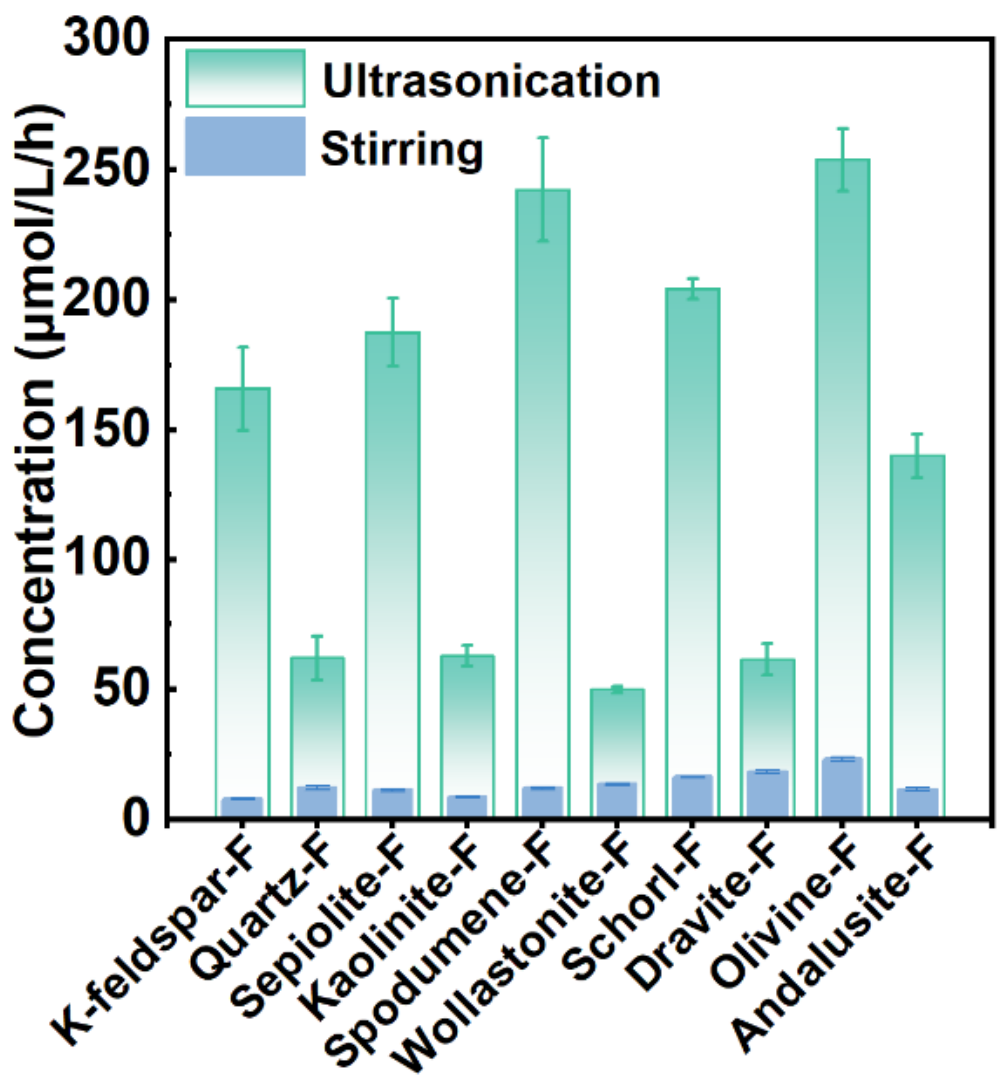

Fig. R8. Comparison of H_2O_2 production of M-F under ultrasonication and magnetic stirring.

Comment 6: Under practical conditions, would the ROS or the modified mineral

surfaces themselves exert toxic effects on beneficial microorganisms?

Reply: We appreciate this important question regarding ecological safety. This concern had been briefly acknowledged in the original Discussion, where we noted that ROS accumulation may pose ecological risks by inducing oxidative stress in sensitive organisms and altering native microbial communities. In response to the reviewer's comment, we have now clarified and expanded this discussion in the revised manuscript.

Under weak-force natural-flow conditions, ROS generation is expected to occur primarily at the mineral-water interface and to remain transient and spatially localized. Genome-resolved metagenomic analysis did not indicate community-wide microbial suppression. Instead, selective enrichment of ROS-tolerant and metabolically versatile taxa was observed, suggesting ecological filtering rather than broad toxicity-driven collapse of the surface-associated microbial community. At the same time, it is acknowledged that microorganisms with weaker antioxidant defense systems or preferences for more reduced microenvironments may be more sensitive to such oxidative conditions [1–3], as reflected by the decreased relative abundance of certain microorganisms in our results. This interpretation is further supported by XPS and SEM observations, showing reduced surface-associated C and N signals on the hydrophobically modified surfaces, implying that some surface-associated taxa or biomass components may be more sensitive to the combined effects of ROS generation and altered surface wettability. Therefore, the conclusions are restricted to the absence of evidence for broad community-level inhibition under the tested conditions, rather than the absence of any taxon-specific effects.

Regarding the modified mineral surfaces, the grafted functional groups are covalently bound to the silica framework, which is expected to limit leaching under the experimental conditions. Nevertheless, long-term exposure and large-scale deployment may still pose ecological risks, and comprehensive environmental risk assessment will be required prior to practical field implementation.

Page 24, lines 420-428: "Sustained ROS generation may also pose ecological risks by inducing oxidative stress in sensitive organisms and disrupting native microbial

communities[42,46,48]. Under the tested conditions, our results did not indicate broad community-wide suppression, although the negative effects on specific sensitive taxa (e.g., those with weaker antioxidant defense systems or lower tolerance to hydrophobic surface conditions) cannot be excluded. In addition, because the grafted functional groups are covalently bound to the mineral surface, leaching-related effects are expected to be limited under the tested conditions, but this aspect should be explicitly evaluated in future work."

References:

[1] Dong H, Zeng Q, Sheng Y, Chen C, Yu G, Kappler A. Coupled iron cycling and organic matter transformation across redox interfaces. *Nat. Rev. Earth Environ* ***4***, 659-673 (2023).

[2] Sheng Y, Hu J, Kukkadapu R, Guo D, Zeng Q, Dong H. Inhibition of extracellular enzyme activity by reactive oxygen species upon oxygenation of reduced iron-bearing minerals. *Environ. Sci. Technol* ***57***, 3425-3433 (2023).

[3] Zhang P, et al. Production and significance of Reactive Oxygen Species in the subsurface. *Earth Sci. Rev* **23**, 105230 (2025).

Comment 7: Microbial attachment varies significantly between hydrophilic and hydrophobic surfaces. How did the authors distinguish the biological response driven by CEC-generated ROS from the physical selection/attachment effects caused by the alkylated -CH_3 surfaces?

Reply: We appreciate the reviewer's insightful comment. It is agreed that alkylated –CH_3 surface modification increases hydrophobicity and may influence initial microbial attachment and biofilm development. In this study, genome-resolved metagenomics was conducted on surface-associated communities formed under identical natural-flow conditions, comparing unmodified and –CH_3–modified stone. It is therefore acknowledged that part of the observed taxonomic variation may reflect differences in surface wettability and early colonization preferences.

To further assess this possibility, genes associated with motility and surface

colonization were examined, including those involved in flagellar assembly (*fli, flg*), type IV pilus (*pil*), adhesion-related proteins (lap), and chemotaxis (*che*). These genes were broadly distributed across many MAGs recovered from both treatments, indicating that attachment and surface-colonization capacity are common characteristics within the surface-associated community. For example, the most strongly enriched MAGs (e.g., *Novosphingobium* bin 24 and bin 17 if applicable) also retained motility-related systems (updated Fig. 4d). However, because such modules are widespread, they cannot independently differentiate hydrophobic-surface selection from ROS-mediated ecological filtering. In other words, attachment-related genes reflect general surface persistence potential, but are not uniquely diagnostic of hydrophobic attachment effects. Previous studies have shown that hydrophobic surfaces can preferentially enhance the attachment of microorganisms with higher cell-surface hydrophobicity and stronger biofilm-forming capacity, although microbial adhesion is also strongly modulated by surface charge, roughness, hydrodynamic conditions, and medium chemistry [1–3].

Beyond potential adhesion effects, several lines of evidence indicate that microbial restructuring cannot be explained solely by hydrophobic attachment. Taxa enriched on S-C (e.g., Sphingomonadaceae-related MAGs) exhibited coordinated functional traits, including complete ROS detoxification systems and enhanced metabolic pathways for oxidized low-molecular-weight substrates (formate, acetate, acetaldehyde, oligosaccharides) as well as complex polymers (cellulose and chitin). In addition, the cumulative relative abundance of oxygen-related metabolism was consistently more enriched on S-C than on S. Differences in physical adhesion would be expected to influence colonization efficiency or biomass accumulation, but would not necessarily result in enrichment of oxidative stress response systems and oxidized-substrate metabolism pathways at the genomic level.

Taken together, these findings support a coupled mechanism in which surface hydrophobicity influences initial microbial attachment, while CEC-driven ROS generation establishes an oxidative microenvironment that exerts additional ecological filtering pressure. Weak ambient water flow and mild turbulence can initiate ROS-

generating surface reactions on mineral substrates, providing proof-of-concept that CEC can function under open-air environmental conditions. To avoid overinterpretation, the manuscript has been revised to clarify that the observed microbial shifts likely arise from the combined effects of altered surface wettability and ROS-mediated redox processes, rather than from ROS alone.

Pages 22-23, lines 384-397: "Moreover, genes related to motility, surface sensing, and colonization, such as flagellar assembly genes (e.g., fli, flg), pilus-associated genes (e.g., pil), adhesion-related proteins (e.g., lap), and chemotaxis genes (e.g., che), were broadly distributed across many MAGs recovered from both treatments, suggesting that the genetic potential for attachment and surface colonization is a common feature of the surface-associated community. Previous studies have shown that hydrophobic surfaces can potentially promote the attachment of microorganisms with higher cell-surface hydrophobicity and stronger biofilm-forming capacity, although microbial adhesion is also strongly modulated by surface charge, roughness, hydrodynamic conditions, and medium chemistry[43-45]. Taken together, these results support a coupled mechanism in which ROS-generating mineral surfaces and altered surface wettability jointly shape a dual ecological niche: producing oxidized organic substrates through ROS-mediated surface reactions while simultaneously selecting for oxidative stress-tolerant microorganisms capable of exploiting these newly generated metabolites."

References:

[1] Zheng S, Bawazir M, Dhall A, Kim H.E, He L, Heo J, Hwang G. Implication of surface properties, bacterial motility, and hydrodynamic conditions on bacterial surface sensing and their initial adhesion. *Front. Bioeng. Biotechnol* **9**, 643722 (2021).

[2] Kimkes TE, Heinemann M. How bacteria recognise and respond to surface contact. *FEMS Microbiol. Rev* **44**, 106-22(2020).

[3] Berne C, Ducret A, Hardy GG, Brun YV. Adhesins involved in attachment to abiotic surfaces by Gram-negative bacteria. *Microbiol. Spectr* **3**, MB-0018-2015 (2015).

Comment 8: For better readability, the "Materials and Methods" section should include subheadings such as "Sample Preparation," "H_2O_2 Synthesis Quantification," and "ROS Detection"

Reply: Thank you for this helpful suggestion. The Materials and Methods section has been reorganized with clear subheadings to improve readability and structure. The revised section now includes the following subheadings: Materials and Methods; Chemical reagents and materials; Sample preparation; H_2O_2 synthesis quantification; Degradation of MO by circulating water flow; and ROS detection.

Comment 9: The reference list contains numerous errors, including incomplete author lists, incorrect sub/superscripts in chemical formulas, erroneous journal titles, and missing page numbers. A thorough proofreading is required.

Reply: Thank you for your insightful observation. We have added and corrected the references in manuscript and Supporting Information.

Comment 10: In Figure 1d, the unit "µMol" should be corrected to the standard lowercase "µmol"

Reply: Thank you for this helpful suggestion. All figures in the manuscript and Supporting Information have been carefully reviewed. The unit in Fig. 1d (Fig. R9) has been corrected from "µMol" to the standard "µmol." The same correction has also been applied to Fig. 1f to ensure consistency throughout the manuscript.

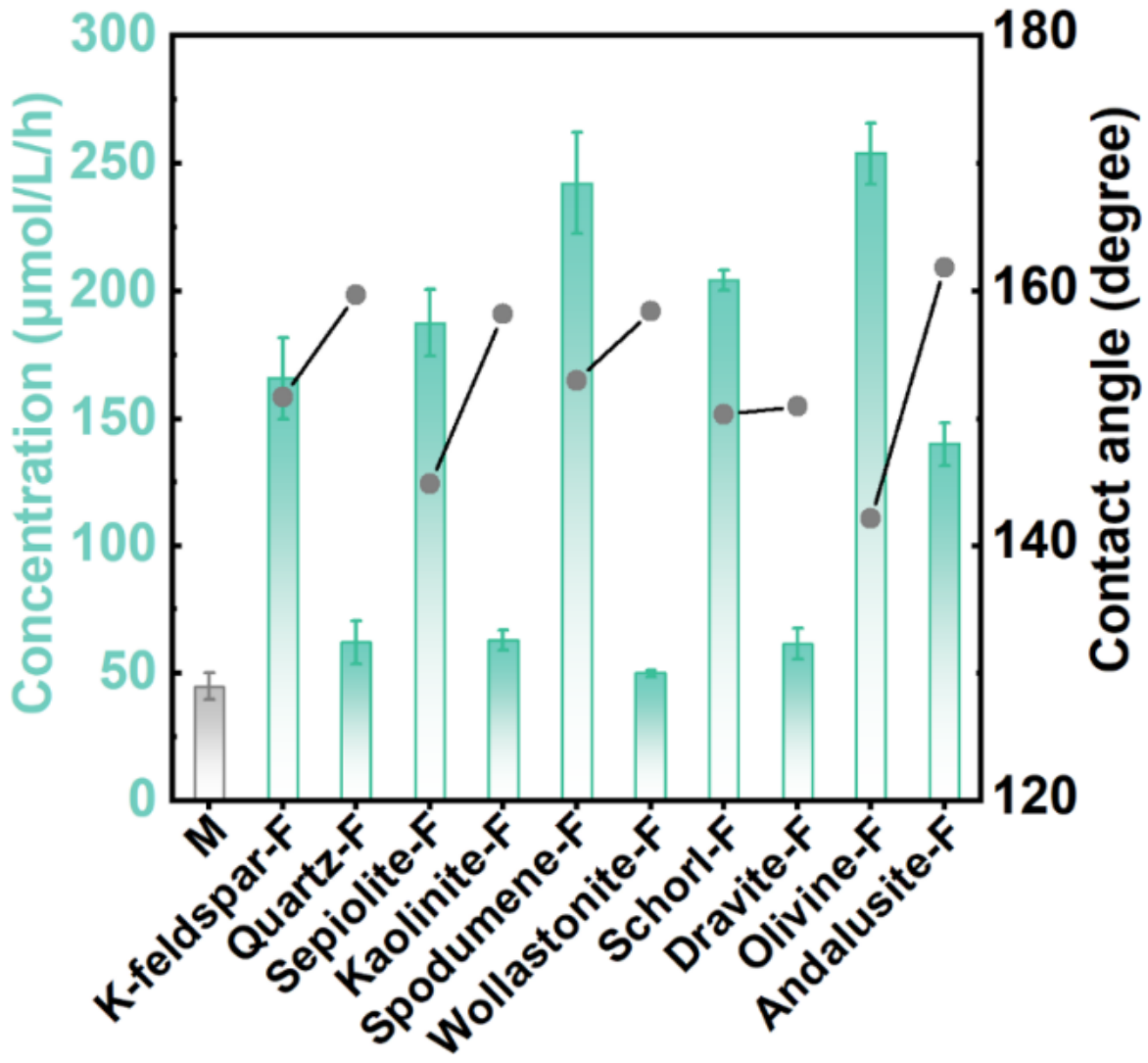

Fig. R9. H_2O_2 production (using 10 samples for error bar analysis) and contact angles of M-F. (Fig.1d)

Reviewer #2: This work explores the use of non-fluorinated and hydrophobically modified natural minerals as catalysts for contact-electro-catalysis (CEC), and replaces the commonly used ultrasonic input with simulated river flow, thereby broadening potential application scenarios. The topic is interesting and potentially meaningful. The manuscript also attempts to rationalize CEC behavior in terms of catalyst hydrophobicity and electronegativity, but some interpretations are not yet sufficiently supported. In addition, the writing and organization lack clarity in places, and the connection between experimental data and analysis is sometimes weak, which may lead to reader confusion. Several mechanistic arguments also require stronger evidence and more rigorous discussion. Overall, I believe the manuscript could be suitable for publication after major revision.

Thank you for your insightful and constructive comments on our manuscript. We have carefully revised the manuscript to clarify the logical relationships among several datasets and to restructure parts of the text for greater accuracy and readability. Detailed responses to each comment are provided below, and it is hoped that the revisions satisfactorily address the concerns raised.

Comment 1: The authors chose to show data for only 5 of the 10 natural silicate mineral samples in multiple places where they should have shown all 10 (e.g., Fig. 1b and 1c, Supplementary Fig. 3, Supplementary Fig. 20, 21), which is very detrimental to the derivation of conclusions.

Reply: Thank you for this constructive suggestion. We have supplemented the corresponding other silicate minerals data in Fig. 1b, Fig. 1c, and Supplementary Fig. 3 (Fig. R1-3) as follows.

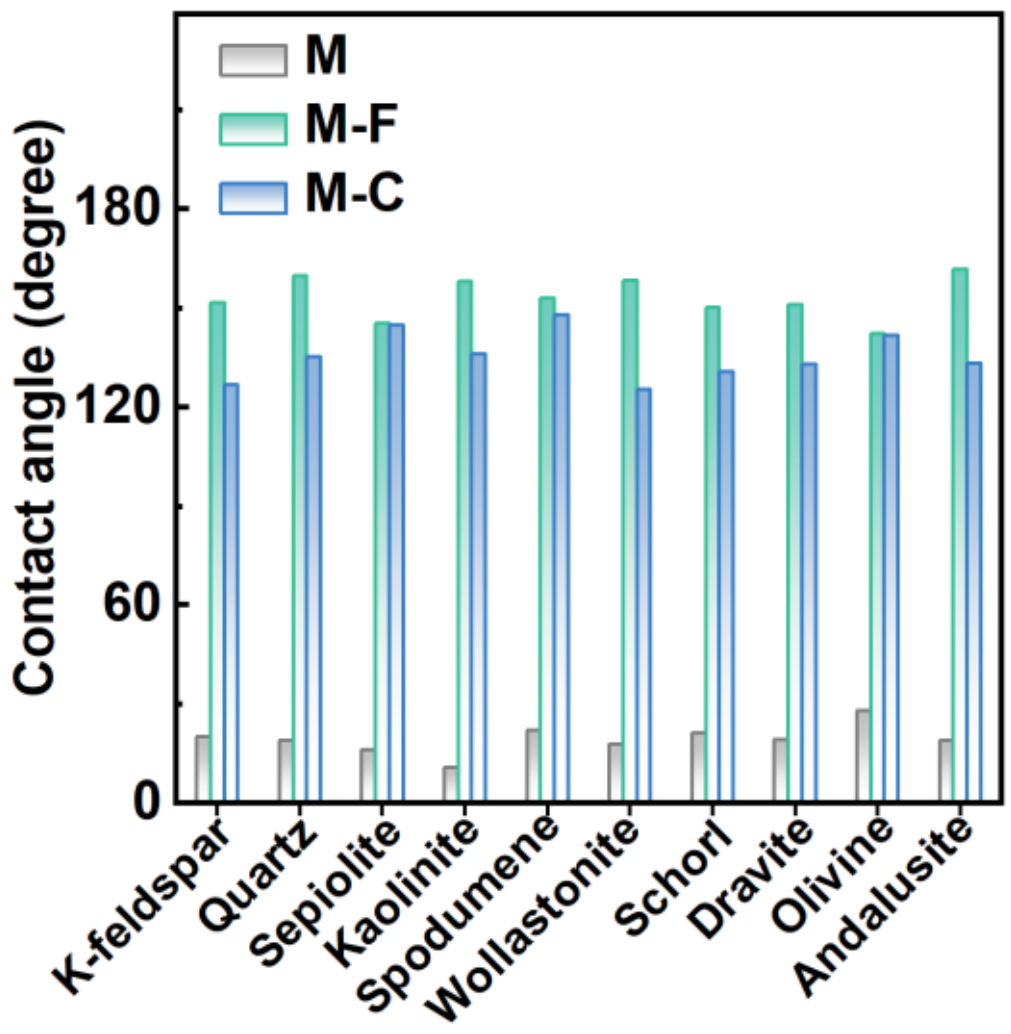

Fig. R1. Contact angle of three types of minerals with different hydrophilicity/hydrophobicity. (Fig. 1b)

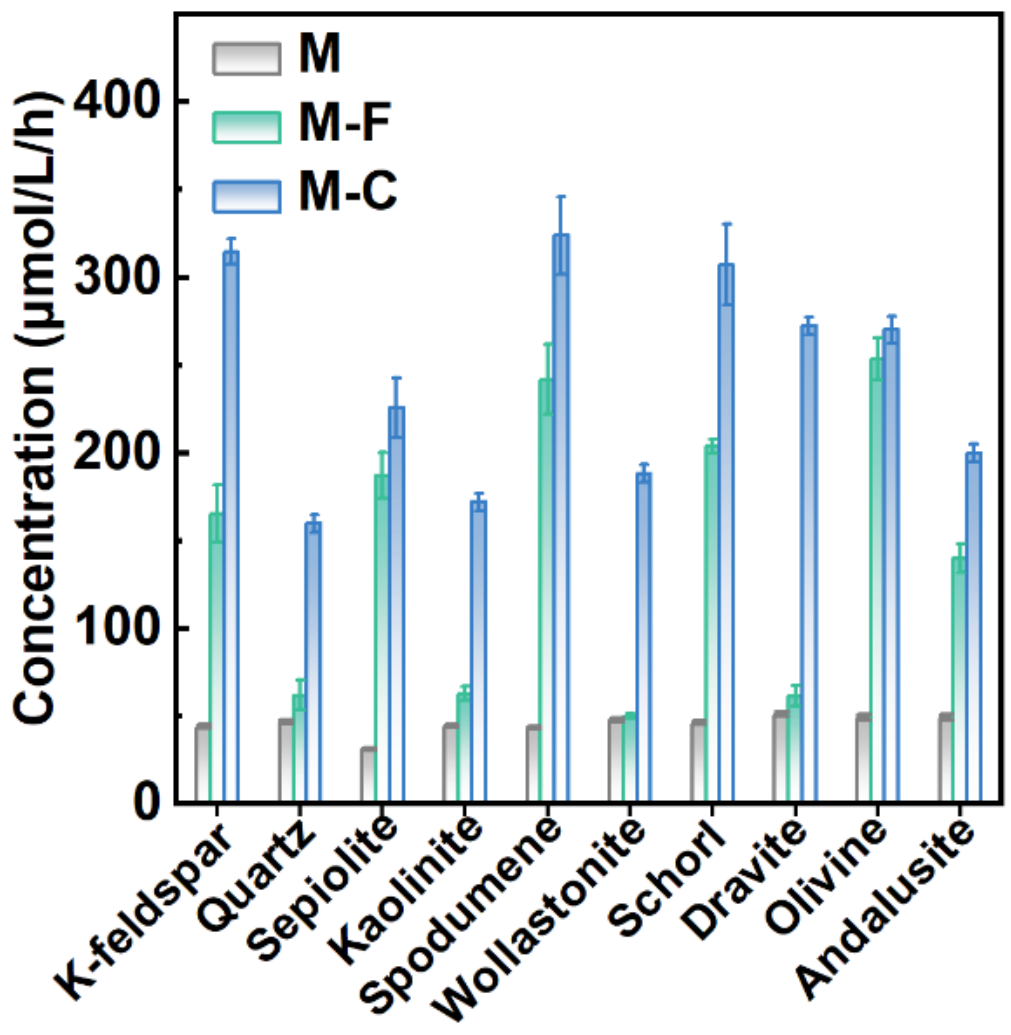

Fig. R2. H_2O_2 production of three types of minerals with different hydrophilicity/hydrophobicity through CEC. (Fig. 1c)

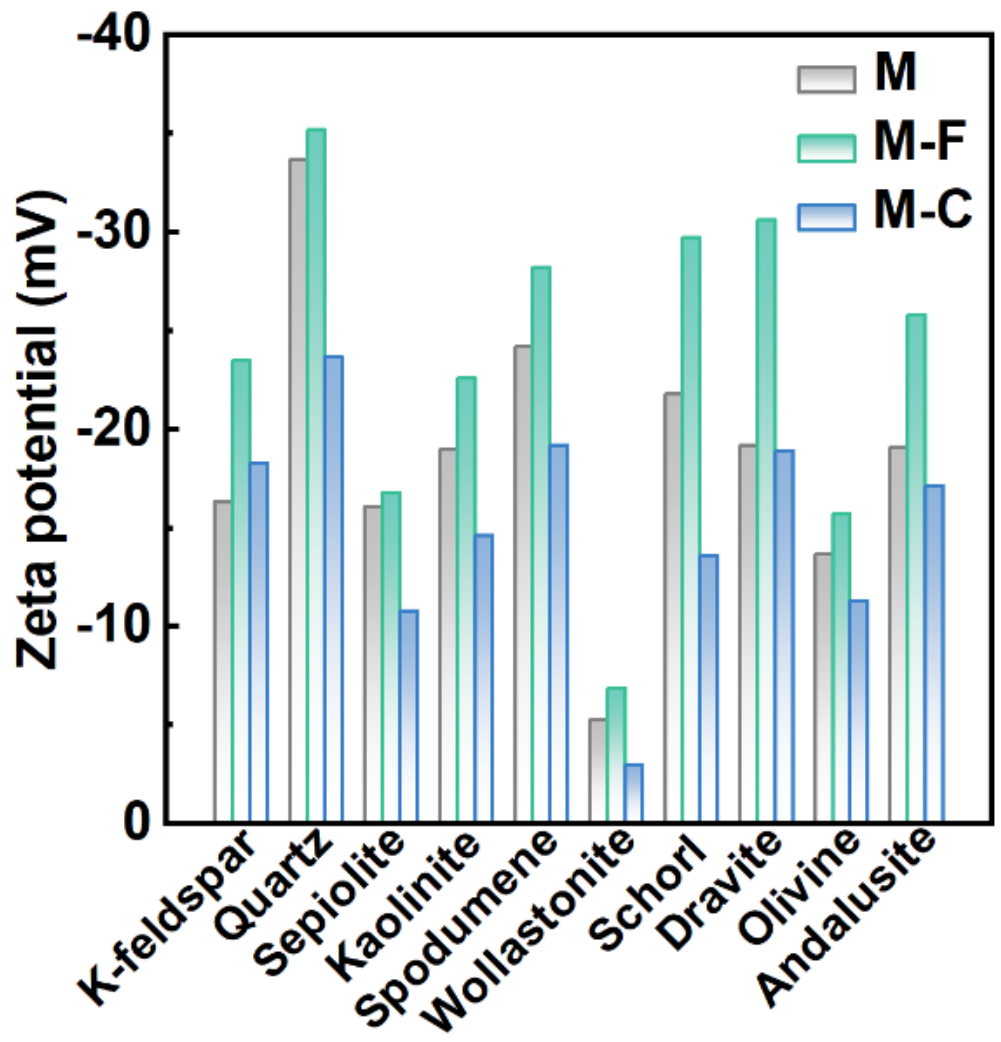

Fig. R3. Zeta potentials of three types of minerals with different hydrophilicity/hydrophobicity. (Supplementary Fig. 3)

The five categories of silicate minerals were classified according to the classical structural criterion, namely the polymerization mode of silicon-oxygen tetrahedra. Accordingly, we selected tectosilicates, phyllosilicates, inosilicates, cyclosilicates, and nesosilicates, which correspond to framework, sheet, chain, ring, and isolated tetrahedral structures, respectively. Two representative minerals were chosen from each category to provide both intra-category and inter-category comparisons. Although these samples cannot include every possible silicate mineral, they cover the major structural motifs of silicates and span a broad range of surface compositions, hydroxyl abundances, and interfacial properties. Therefore, this selection provides a sufficiently representative mineral set to evaluate whether hydrophobicity regulation is a broadly applicable strategy for improving mineral-based CEC performance.

Supplementary Fig. 20, 21 which relate to calcination and plasma treatments respectively, were retained as optimization results and key evidence demonstrating the **effect of wettability regulation on CEC performance**. These data have now been integrated into Fig. 1e in the revised manuscript. In addition, XPS analyses for all ten M-F samples (Supplementary Fig. 6), as well as for the five calcined then fluorinated minerals and five plasma-treated then fluorinated minerals (Supplementary Fig. 7),

have been added to further clarify the relationship among surface composition, wettability, and CEC performance.

With respect to the calcination and plasma experiments, the objective was not to indiscriminately optimize all ten minerals, but rather to further validate the role of hydrophobicity/hydrophilicity regulation in CEC. The ten silicate minerals were selected as two representatives from each of five structural classes. Within each class, only the sample exhibiting **a relatively larger contact angle and lower CEC performance** (Fig. R4a) was chosen for **further calcination and plasma treatment**. These five minerals were regarded as the most suitable candidates for optimization because they represented the lower-performing samples within each class and therefore provided the greatest potential for performance enhancement. In contrast, the other five minerals in the five classes already demonstrated relatively favorable CEC activity and were therefore not subjected to additional optimization treatments.

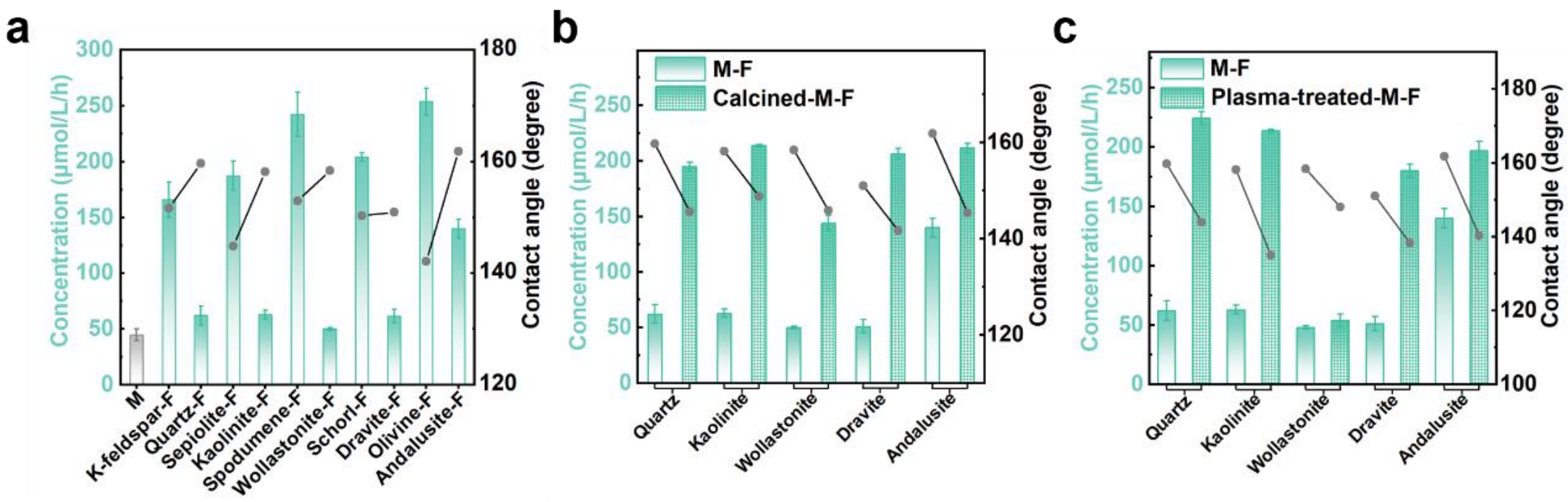

Fig. R4. Relationship between moderate hydrophobicity and enhanced CEC performance. (a) H_2O_2 production (using 10 samples for error bar analysis) and contact angles of M-F. (b) H_2O_2 production and contact angles of calcined then fluorinated silicate minerals. (c) H_2O_2 production and contact angles of plasma-treated then fluorinated silicate minerals. (Fig. 1d–e; Supplementary Fig. 5)

Following calcination or plasma treatment, all originally low-performing M-F samples exhibited significant enhancement in CEC performance. A shared characteristic of these optimized samples was a reduction in contact angle, shifting from a superhydrophobic state to a moderately hydrophobic condition (Fig. R4b-c). Based on the newly incorporated XPS results (Fig. R5), fluorination modification on all minerals was found to be about saturated. Thus, variations in hydrophobicity among the M-F samples do not primarily arise from major differences in fluorination extent,

but rather from differences in the residual surface oxygen-containing groups remaining after modification. Higher residual oxygen content mitigates excessive superhydrophobicity, increases the contact probability between water and the fluorinated surface, and consequently enhances CEC performance. The detailed analysis is provided in the response to **Comment 3**. These findings further substantiate the central conclusion that moderately regulated wettability, rather than maximized hydrophobicity alone, is critical for achieving superior CEC activity.

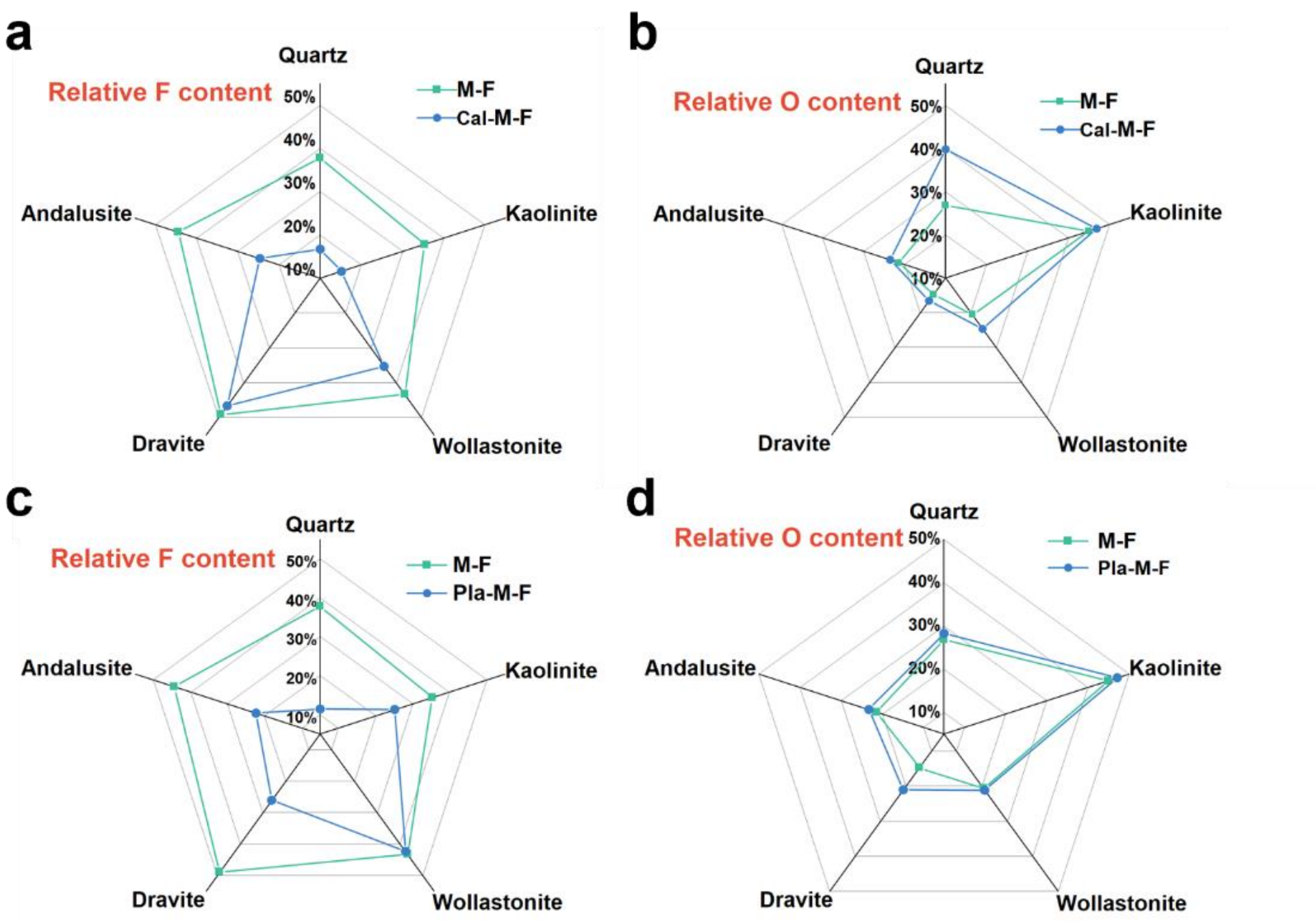

Fig. R5. (a) Relative fluorine content and (b) relative oxygen content of M-F and calcined then fluorinated minerals measured by XPS. (c) Relative fluorine content and (d) relative oxygen content of M-F and plasma-treated then fluorinated minerals measured by XPS. (Supplementary Fig. 7)

To clarify this logic clearer, the related discussion in the manuscript has been revised as follows.

Page 8, lines 142-154: "Although M-C is less hydrophobic than M-F is, it consistently has higher CEC performance than M-F does, indicating that excessively high hydrophobicity is unfavorable for CEC (Fig. 1b-c). To further isolate the effect of contact properties while minimizing the influence of differences in electron-withdrawing groups, we used M-F as a comparable platform to study the regulation of

hydrophobicity, because the samples exhibit the most different contact angles before and after modification. Among mineral pairs across five structural classes—tectosilicates, phyllosilicates, inosilicates, cyclosilicates, and nesosilicates—those with moderate hydrophobicity consistently presented higher CEC performance (Fig. 1d). For the five minerals with the highest contact angles and lowest CEC activity, optimization by calcination or plasma treatment before fluorination reduced the contact angle and enhanced the CEC performance in all the cases (Fig. 1e, Supplementary Fig. 4-5), further demonstrating that moderate contact with water is a key factor controlling the CEC."

Pages 8-9, lines 155-174: "To better understand why the M-F samples still exhibited different wettability and CEC performances, we further analyzed their surface composition by XPS (Supplementary Fig. 6). The results revealed that the relative oxygen content of the surface was closely associated with the final wettability and CEC activity of the minerals: samples with a higher relative oxygen content on the surface generally exhibited lower contact angles and higher CEC performance, and the same trends were observed for the calcined then fluorinated minerals and the plasma-treated then fluorinated minerals (Supplementary Fig. 7). These results suggest that although fluorination reached a consistently high level under the present modification conditions, complete substitution of all surface oxygen-containing sites was still limited by the steric hindrance of the carbon-fluorine chains of POTS. As a result, differences in the remaining polar surface sites led to subtle but important variations in wettability, which further affected the effective mineral-water contact during CEC (Supplementary Fig. 8). This interpretation is also supported by the modifier dosage experiment, in which excessive fluorination caused a marked decrease in catalytic activity (Fig. 1f), indicating that overly strong hydrophobicity suppresses effective mineral-water contact (Supplementary Fig. 9-10 and Supplementary Note 2). Therefore, for M-F, the final surface wettability is a more relevant descriptor of CEC performance than the fluorination treatment alone, and a moderately hydrophobic surface is more favorable for efficient CEC."

Comment 2: The manuscript mentions that "moderate hydrophobicity" is the key, is this a universal rule or specific to sepiolite, as the manuscript only provides data on the "hydrophobicity-CEC activity" relationship for sepiolite-F (Fig. 1f and Supplementary Fig. 15). Given the repeated emphasis on the critical role of hydrophobicity in both the paper's title and main text, it is recommended that the "hydrophobicity-CEC activity" relationship curves for the other nine silicate minerals be supplemented.

Reply: We thank the reviewer for this thoughtful comment. We fully understand the reviewer's concern regarding whether the conclusion that "moderate hydrophobicity" is favorable for CEC represents a general principle across silicate minerals rather than a phenomenon limited to a single mineral system. The suggestion to provide additional hydrophobicity–CEC activity curves could, in principle, provide further comparative information on fluorinated minerals.

After careful consideration, we would like to respectfully clarify that the central contribution of this work is the establishment of a **hydrophobicity-regulation strategy** for CEC. In the revised manuscript, Fig. 1 (Fig. R6) has been further clarified to emphasize the relationship between surface wettability and CEC performance. Specifically, Fig. R6a-c demonstrates the **consistent correspondence** between moderate hydrophobicity and enhanced CEC activity under **different surface modification strategies**, while Fig. R6d-e further demonstrates the same trend across **different mineral types**. Collectively, these results support a general design principle: neither fully hydrophilic nor excessively hydrophobic surfaces are optimal, whereas moderated wettability favors CEC. Although the exact optimal wettability window may vary depending on mineral surface structure and chemistry, but the overall trend is consistently supported by the datasets presented in this study.

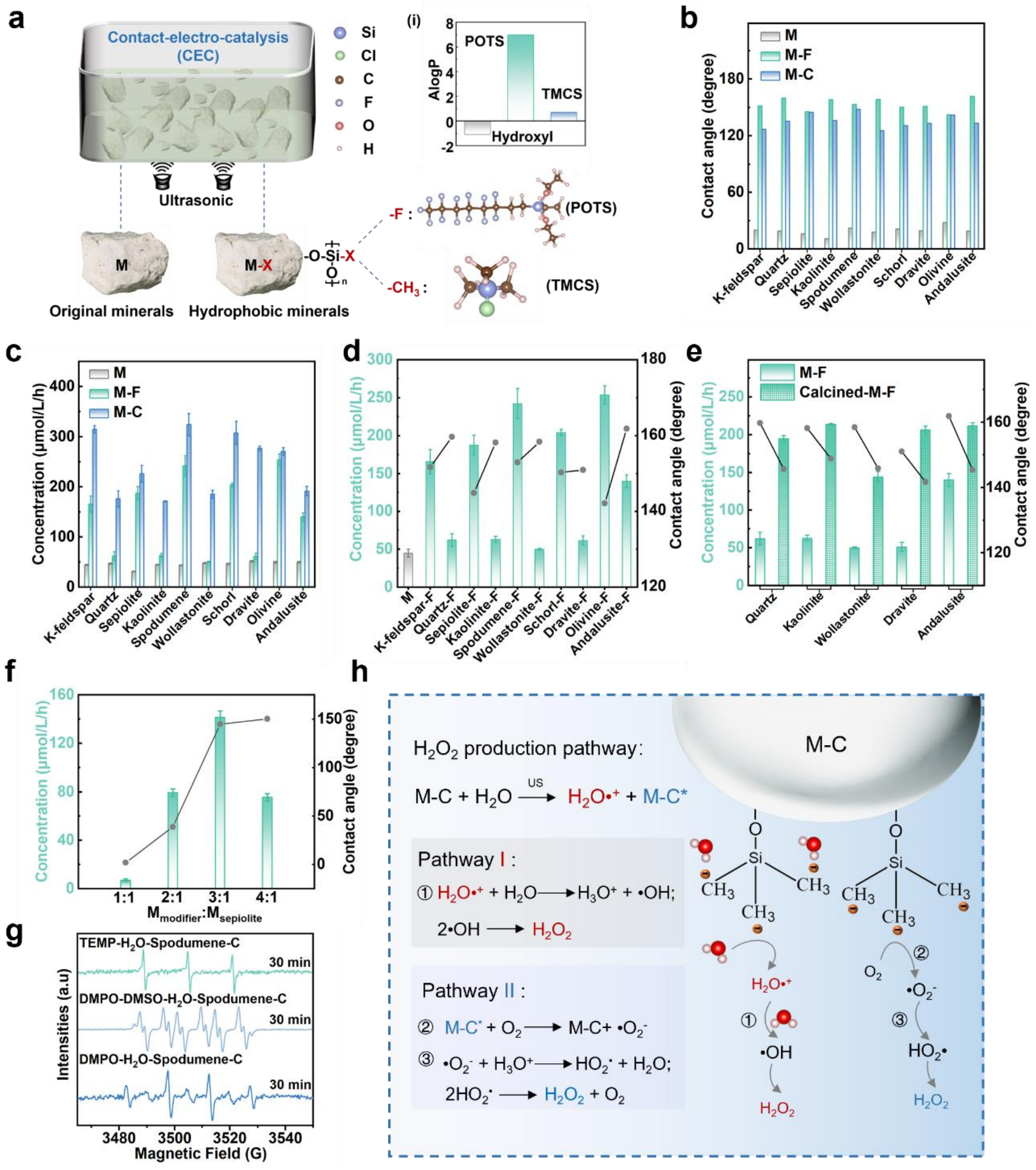

Fig. R6. Hydrophobic modification of minerals and their CEC performances. (a) Schematic diagram of the modification strategy and ultrasonic-assisted CEC device (illustration: AlogP values of different hydrophobic/hydrophilic molecules). (b) Contact angles of three types of minerals with different hydrophilicity/hydrophobicity. (c) H_2O_2 production of three types of minerals with different hydrophilicity/hydrophobicity through CEC. (d) H_2O_2 production (using 10 samples for error bar analysis) and contact angles of M-F. (e) H_2O_2 production and contact angles of calcined then fluorinated silicate minerals. (f) Influence of the mass ratio of modifiers and sepiolite on the CEC performance and the contact angle of sepiolite-F. (g) Electron paramagnetic resonance spectra of spodumene-C under ultrasonication for 30 min. (h) Schematic illustration of ROS generation by

M-C. (Fig. 1)

To further understand why different M-F samples exhibit different hydrophobicity, additional surface-composition analyses have been incorporated in Supplementary Fig. 6-7 (Fig. R7). These data **reveal a clear correlation** between residual surface oxygen content and contact angle. Under the present modification conditions, fluorination approached saturation on all mineral surfaces, and the observed variations in hydrophobicity among different M-F samples primarily arise from differences in residual surface oxygen groups. Higher residual oxygen content mitigates excessive hydrophobicity, increases effective mineral-water contact probability, and consequently enhances CEC performance. The detailed analysis is provided in the **response to Comment 3**. These newly added results offer a direct mechanistic explanation for mineral-dependent differences in M-F behavior.

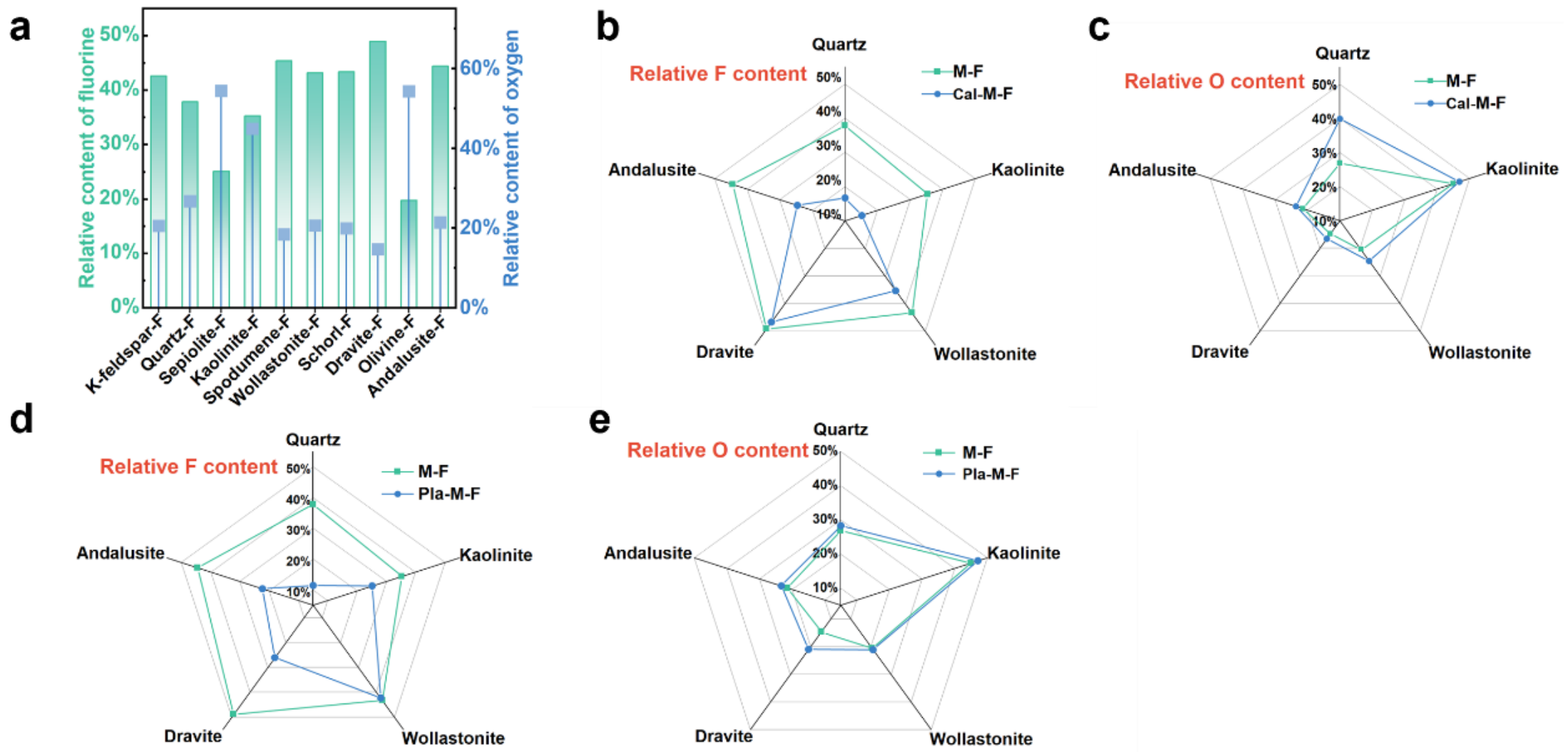

Fig. R7. (a) Relative fluorine content and relative oxygen content of M-F surfaces measured by XPS. (b) Relative fluorine content and (c) relative oxygen content of M-F and calcined then fluorinated minerals measured by XPS. (d) Relative fluorine content and (e) relative oxygen content of M-F and plasma-treated then fluorinated minerals measured by XPS. (Supplementary Fig. 6-7)

Based on these linked relationships—**residual surface oxygen content** versus **wettability** (Fig. R6d-e; Fig. R7) and **wettability** versus **CEC performance** (Fig. R6b-e)—full gradient fluorination studies on all ten minerals are not considered necessary.

Such experiments would mainly reproduce the same trends without providing fundamentally new mechanistic insight. Instead, one representative system was selected for gradient regulation (Fig. R6f) to integrate surface composition, wettability modulation, and CEC output within a single controlled platform. This approach provides a concise yet comprehensive validation of the composition-wettability-performance relationship while maintaining focus and clarity in the manuscript.

All supplementary XPS spectra for the total elemental composition are shown below:

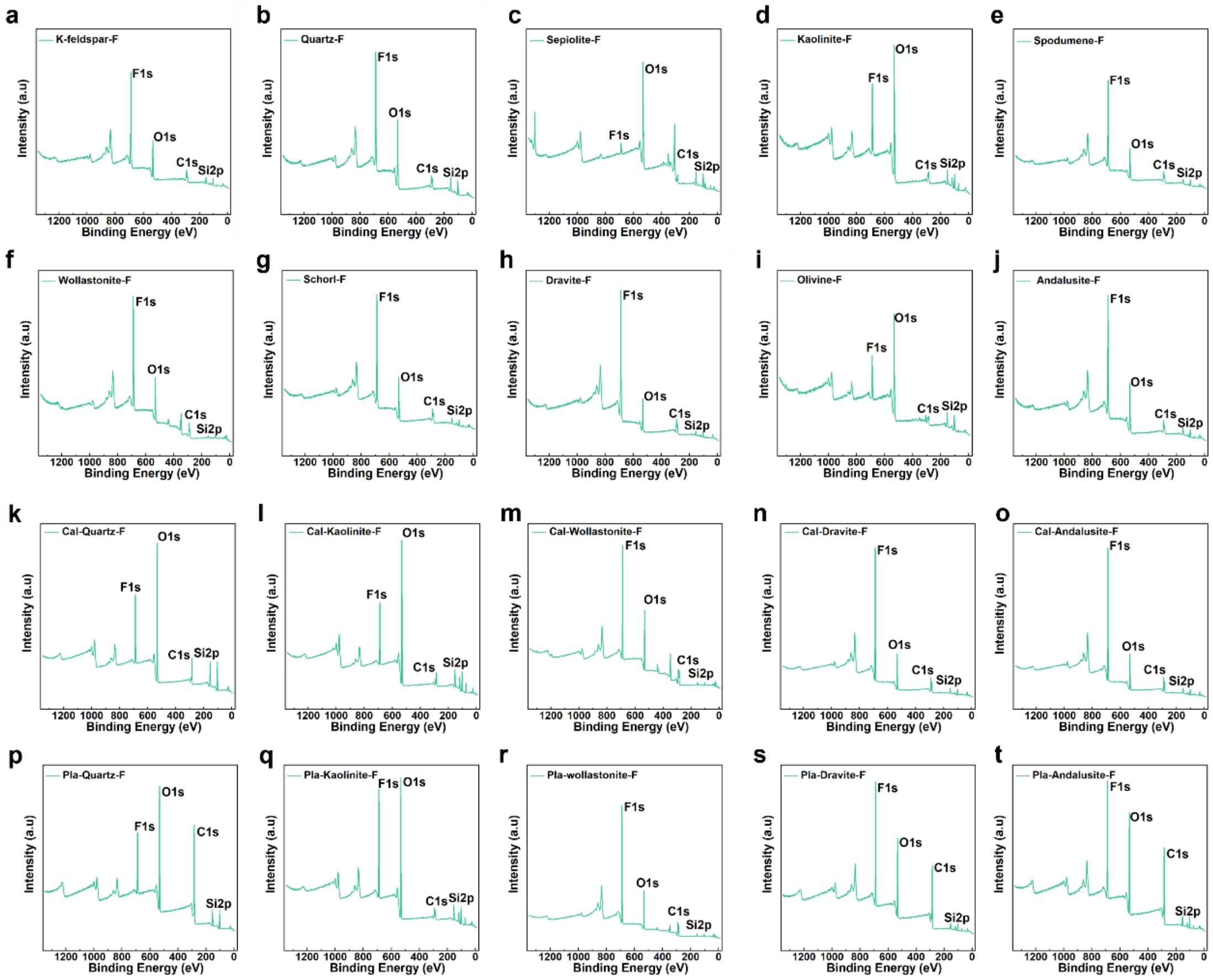

Fig. R8. The total elemental spectra of (a-j) M-F, (k-o) Cal-M-F, and (p-t) Pla-M-F by XPS.

Comment 3: The author's explanation for the different effects of H_2O_2 production via CEC after fluorination of different silicate minerals (Fig. 1d) is confusing. First, different degrees of -F modification result from different silica-to-oxygen ratios. How the degree of -F modification was determined; the authors did not determine the F content of the different fluorinated silicate minerals. Then, different amounts of reactive

oxygen atoms were exposed on different crystalline surfaces at the same silicon to oxygen ratio. How were the active oxygen exposure areas between spodumene and wollastonite calculated in Fig. 1e? Were the exposed crystal faces of spodumene and wollastonite in the inset determined by any assay? Furthermore, with a nearly 1-fold difference in silica-oxygen ratios, does the crystal surface exposure really result in such a large degree of -F modification, which brings about a difference in activity of several times? All of these are very important to whether the hydrophobic strategies highlighted in the title are universal. They should not be studied and recounted in such a generalized manner.

Reply: We sincerely thank the reviewer for these detailed and insightful comments. We fully agree that our original explanation for the different CEC performances of fluorinated silicate minerals was not sufficiently rigorous. In the previous version of the manuscript, we relied too heavily on indirect structural considerations, such as silicon-oxygen ratios, presumed exposed crystal faces, and inferred reactive oxygen atoms exposure, without providing direct evidence for the actual surface composition after fluorination. We recognize that this made the structure-performance relationship unclear and could easily lead to confusion.

In response, the interpretation has been revised to shift from indirect crystallographic inference to direct surface characterization. Rather than estimating fluorination degree from crystal structure or assumed exposed facets, additional XPS analyses were conducted to directly determine the surface elemental composition of the modified minerals. This method more accurately reflects the actual outermost surface state participating in mineral-water contact during CEC.

The newly incorporated XPS results provide three consistent lines of evidence. First, among the ten fluorinated minerals, higher relative surface oxygen content generally correlates with lower contact angles and higher CEC activity (Fig. R9). Second, for the five minerals subjected to calcination prior to fluorination and plasma treatment prior to fluorination, surface oxygen content increased, accompanied by reduced hydrophobicity and improved CEC performance (Fig. R10). These results indicate that the surface composition measured by XPS can effectively capture the actual

fluorination state and residual oxygen of the mineral surface and its resulting wettability.

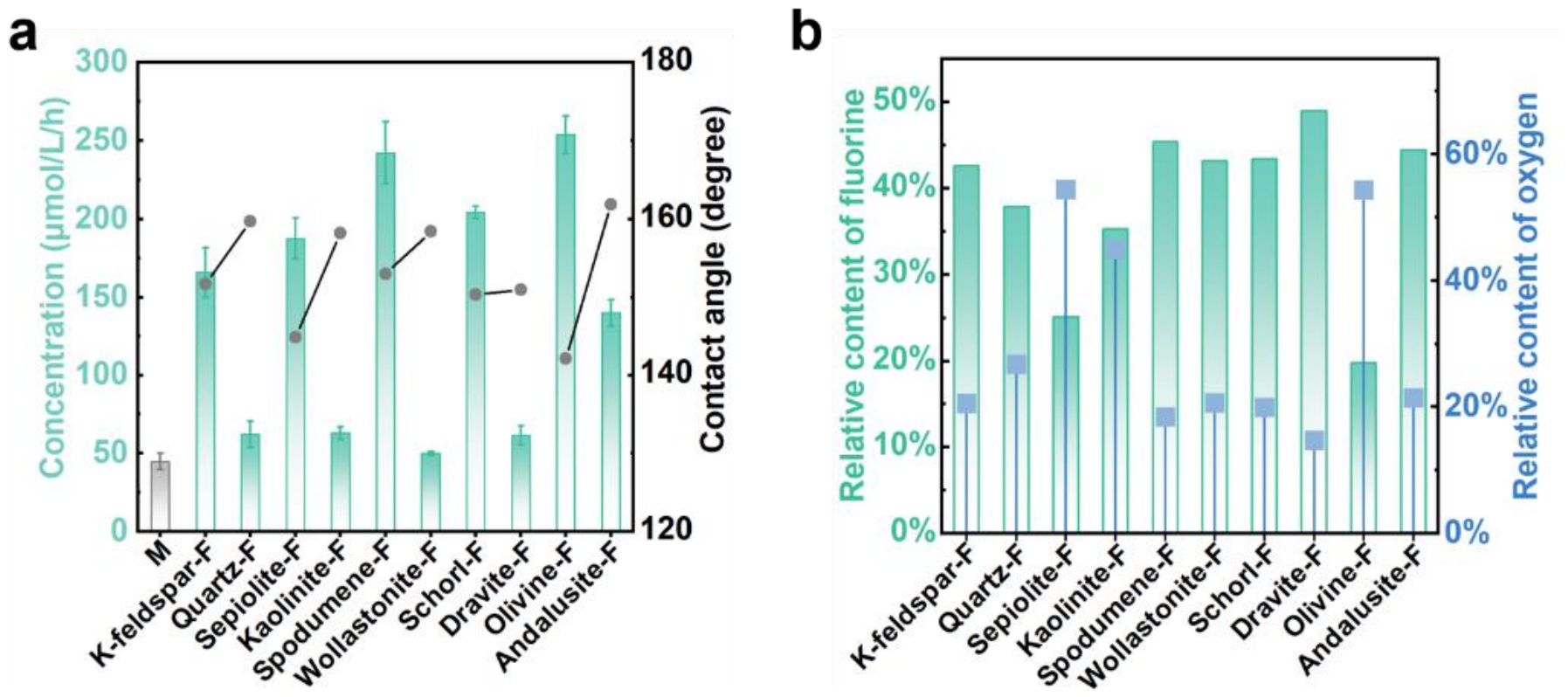

Fig. R9. Relationship between relative oxygen content of surface and contact angle and CEC performance of M-F. (a) H_2O_2 production (using 10 samples for error bar analysis) and contact angles of M-F. (b) Relative fluorine content and relative oxygen content of M-F surfaces measured by XPS. (Fig. 1d, Supplementary Fig.6)

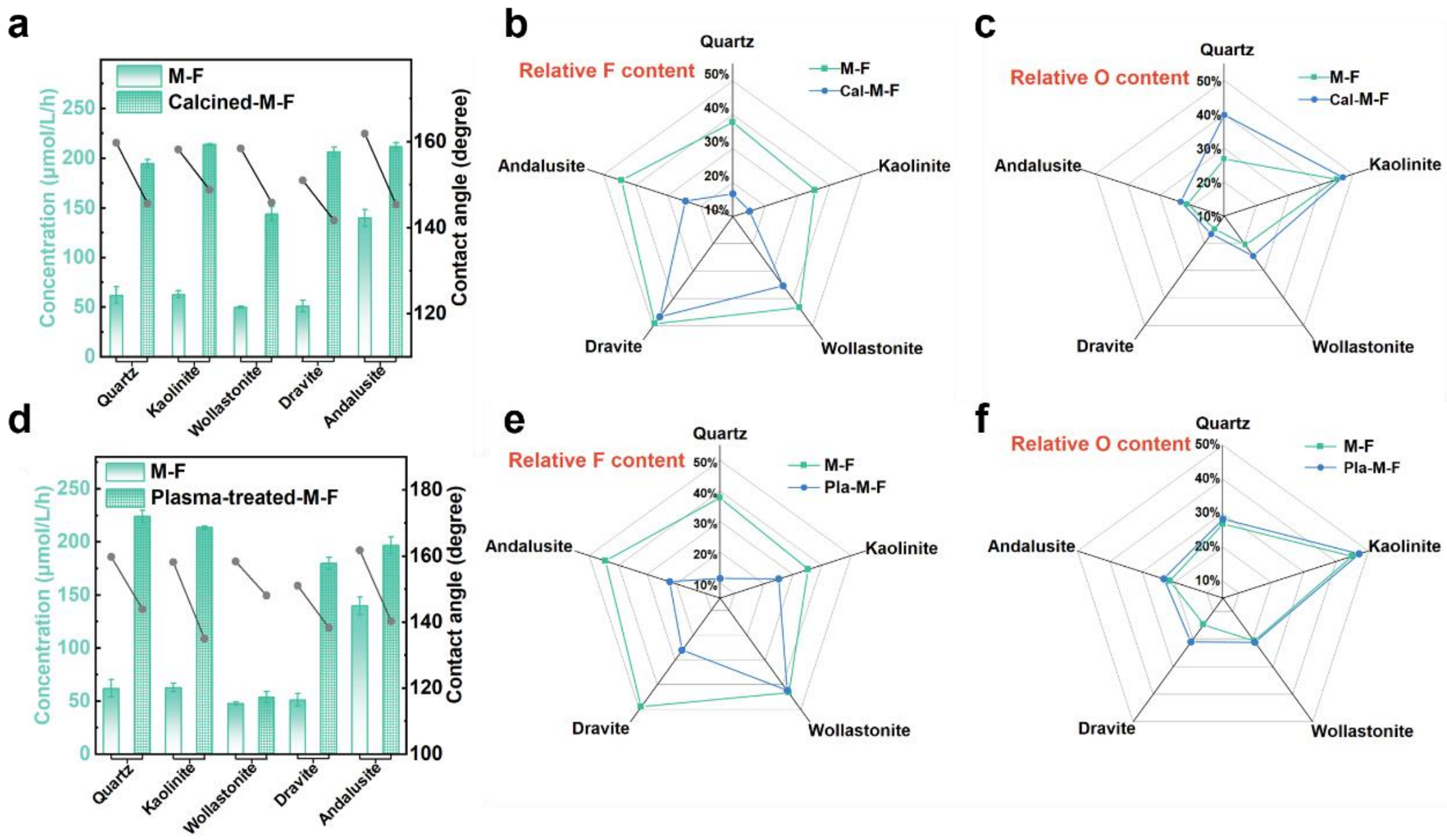

Fig. R10. (a) H_2O_2 production and contact angles of calcined then fluorinated silicate minerals. (b) Relative fluorine content and (c) relative oxygen content of M-F and calcined then fluorinated minerals measured by XPS. (d) H_2O_2 production and contact angles of plasma-treated then fluorinated silicate minerals. (e) Relative fluorine content and (f) relative oxygen content of M-F and plasma-treated then fluorinated minerals measured by XPS. (Fig.1e, Supplementary Fig. 5, 7)

Under the present modification conditions, the fluorination reagent was supplied in excess, and all M-F samples exhibited consistently high contact angles (Fig.1b, 1f),

suggesting near-saturation fluorination coverage. However, due to steric hindrance of the bulky POTS, carbon-fluorine chains, complete substitution of all surface oxygen-containing sites is unlikely. Therefore, differences among fluorinated minerals are more reasonably attributed to variations in residual polar surface sites rather than to large differences in fluorination extent. Minerals retaining more residual oxygen groups exhibit slightly reduced superhydrophobicity, which enhances effective mineral-water contact and benefits CEC performance (Fig. R9; Fig. R11). The calcination then fluorination and plasma-treatment then fluorination experiments respectively further support this interpretation: increasing surface oxygen sites shifts samples from excessively hydrophobic toward moderately hydrophobic states, with corresponding improvement in CEC performance (Fig. R10-11).

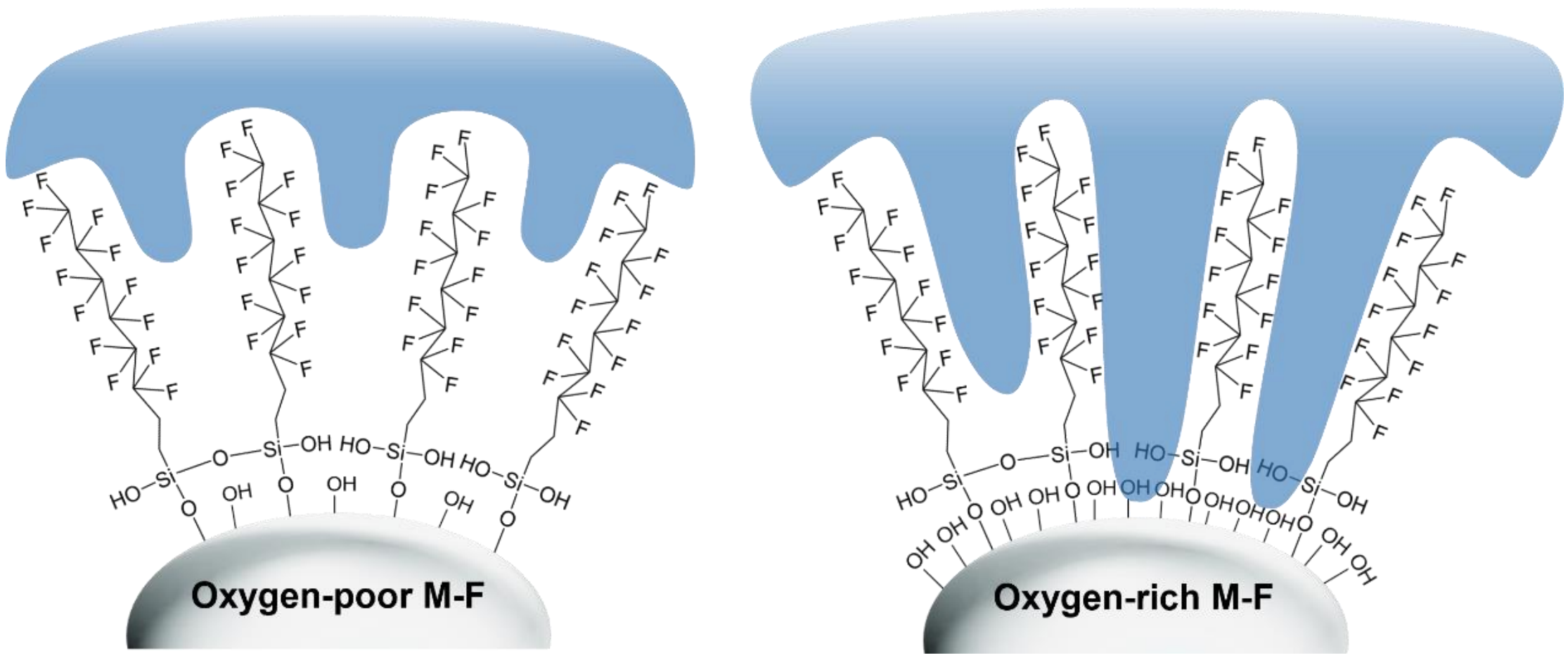

Fig. R11. Schematic diagram illustrating the contact state between water and the surfaces of oxygen-rich M-F and oxygen-poor M-F after M is modified with sufficient modifier. Oxygen groups are represented by hydroxyl groups. (Supplementary Fig. 8)

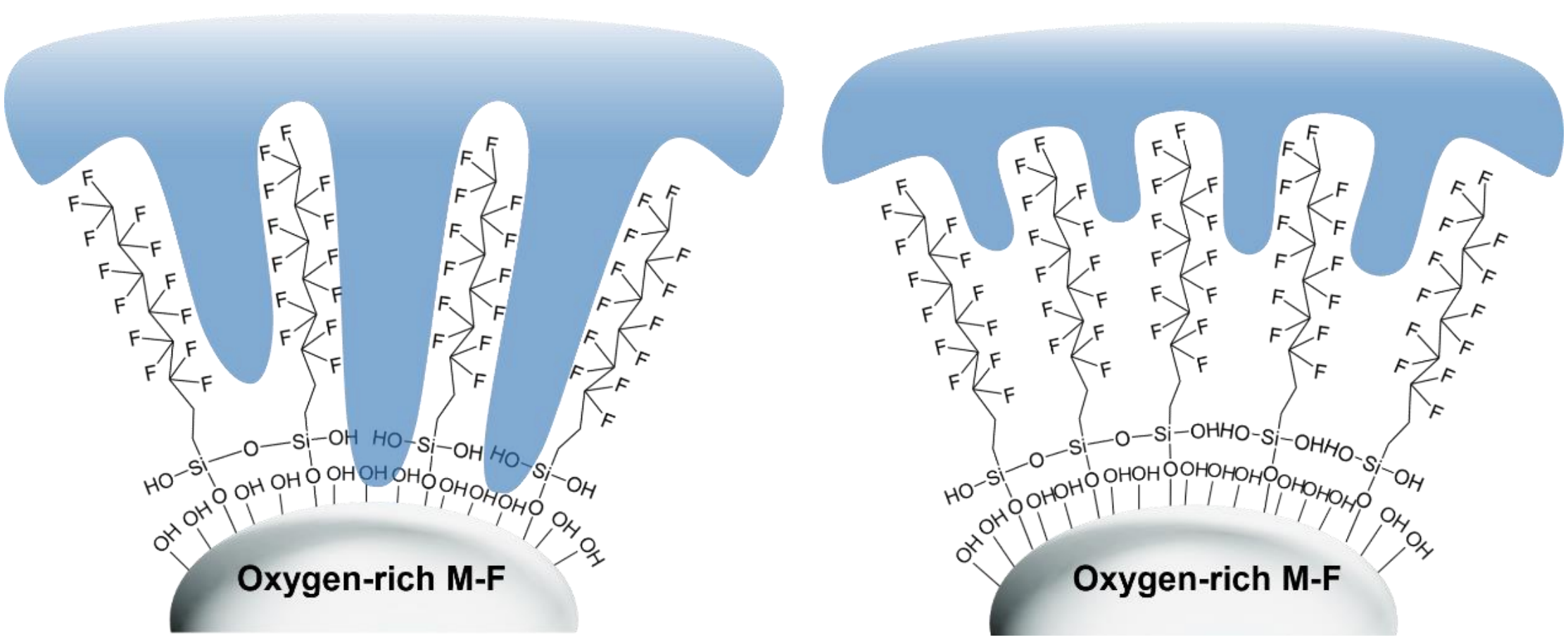

Fig. R12. Schematic diagram illustrating the contact state between the surfaces of oxygen-rich M-F and water after modification of M with sufficient modifier (left) and excessive modifier (right). Oxygen groups are represented by hydroxyl groups. (Supplementary Fig.9)

By tuning the dosage of the fluorinated modifier, we found that when the loading of -F group became excessive (4:1), the increase in hydrophobicity was nearly saturated, whereas the CEC performance dropped sharply (Fig. 1f). This finding indicates that simply increasing –F content does not enhance CEC; instead, overly strong hydrophobicity suppresses effective mineral-water contact and reduces catalytic efficiency (Fig. R12). Although detailed investigation of –F group dispersion is beyond the scope of the present study, the observed contact angle-performance relationship consistently supports the conclusion that moderate hydrophobicity is more favorable for CEC.

Accordingly, the discussion has been revised to emphasize XPS-based surface composition analysis as the primary evidence supporting the structure-performance relationship. This revision strengthens the mechanistic logic and more clearly supports the general applicability of the hydrophobicity-regulation strategy presented in this study.

We have revised the related discussion in the manuscript accordingly.

Pages 8-9, lines 155-174: "To better understand why the M-F samples still exhibited different wettability and CEC performances, we further analyzed their surface composition by XPS (Supplementary Fig. 6). The results revealed that the relative oxygen content of the surface was closely associated with the final wettability and CEC activity of the minerals: samples with a higher relative oxygen content on the surface generally exhibited lower contact angles and higher CEC performance, and the same trends were observed for the calcined then fluorinated minerals and the plasma-treated then fluorinated minerals (Supplementary Fig. 7). These results suggest that although fluorination reached a consistently high level under the present modification conditions, complete substitution of all surface oxygen-containing sites was still limited by the

steric hindrance of the carbon-fluorine chains of POTS. As a result, differences in the remaining polar surface sites led to subtle but important variations in wettability, which further affected the effective mineral-water contact during CEC (Supplementary Fig. 8). This interpretation is also supported by the modifier dosage experiment, in which excessive fluorination caused a marked decrease in catalytic activity (Fig. 1f), indicating that overly strong hydrophobicity suppresses effective mineral-water contact (Supplementary Fig. 9-10 and Supplementary Note 2). Therefore, for M-F, the final surface wettability is a more relevant descriptor of CEC performance than the fluorination treatment alone, and a moderately hydrophobic surface is more favorable for efficient CEC."

Comment 4: DFT calculations reveal that, at equivalent distances between the catalyst and H_2O molecules, the electron transfer amount at M-C (0.081 e at 1.8 Å) is markedly higher than that at M-F (0.019 e at 1.8 Å) (Fig. 1d). Does this contradict the stronger electronegativity of M-F (Fig. 2e)? Have computational models adequately accounted for the structure of water clusters at the actual interface, the dynamic conformations of modifying groups, and the influence of the double electric layer? Can this substantial discrepancy be fully explained by "contact propensity" alone?

Reply: We thank the reviewer for this valuable comment.

(1) According to the electron transfer results between M-C, M-F and water clusters and the discussion, the electron transfer amount at M-C (0.081 e at 1.8 Å) is significantly higher than that at M-F (0.019 e at 1.8 Å) (original Fig. 2d), which is not contradictory to the stronger electronegativity of M-F. The electron transfer between M-C and M-F and water involves two processes: electron cloud overlapping and electron transfer between the potential wells. Due to the same distance, M-C exhibits a relatively stronger hydrophilicity and interaction force with water than M-F. Therefore, M-C is in better contact with water than M-F, and the electron clouds overlap more strongly, which creates favorable conditions for subsequent electron transfer.

(2) We have further supplemented the DFT calculations regarding the water cluster (Fig. R13-14) and different conformations of modification groups (Fig. R15). It was

found that the water cluster and the different conformations of modified group did not affect the advantage of the moderate hydrophobicity of M-C in contacting more effectively with water.

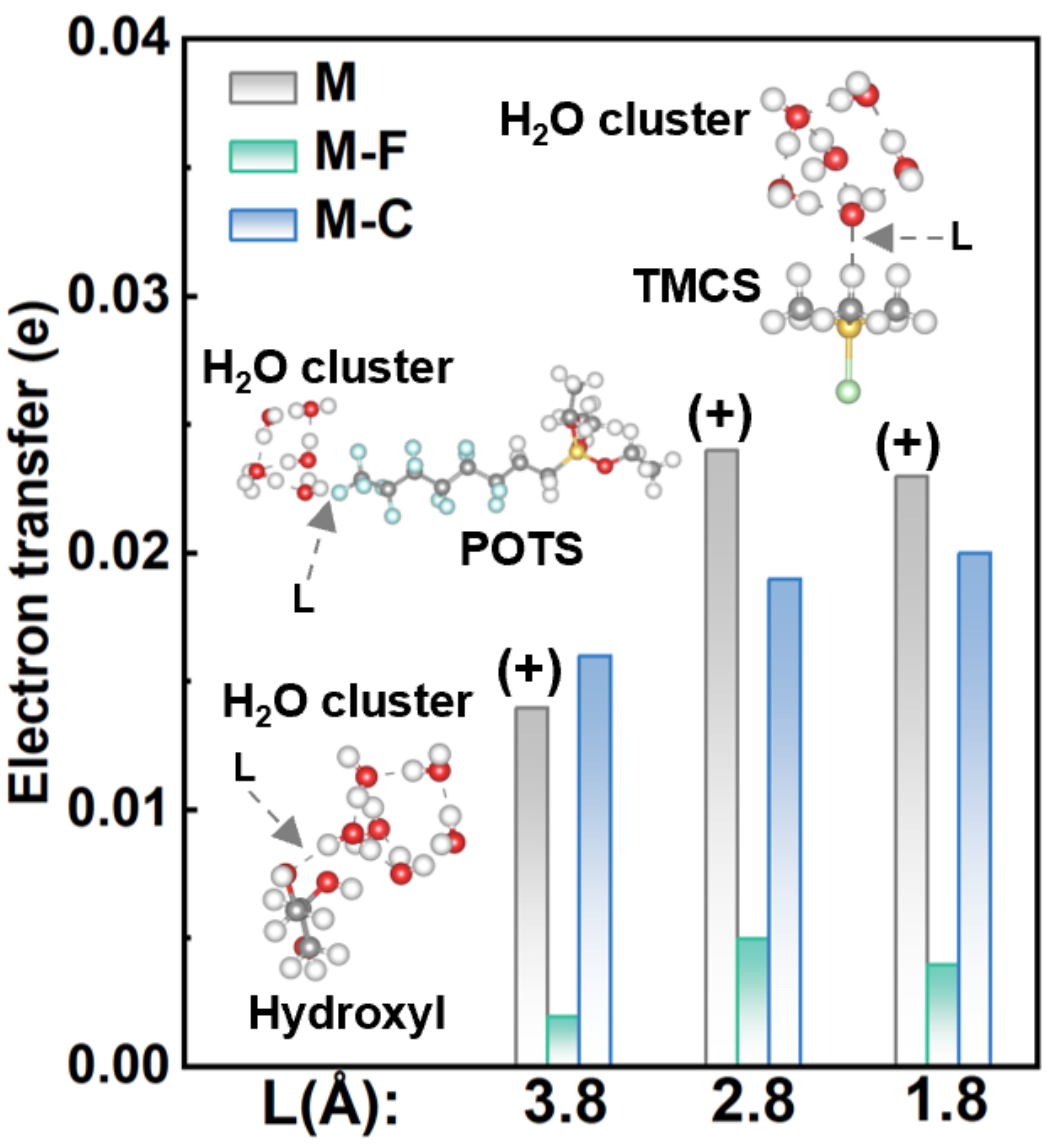

Fig. R13. Electron transfer between hydrophilic/hydrophobic molecules and a hexameric water cluster as a function of distance based on DFT simulations. (Fig. 2d)

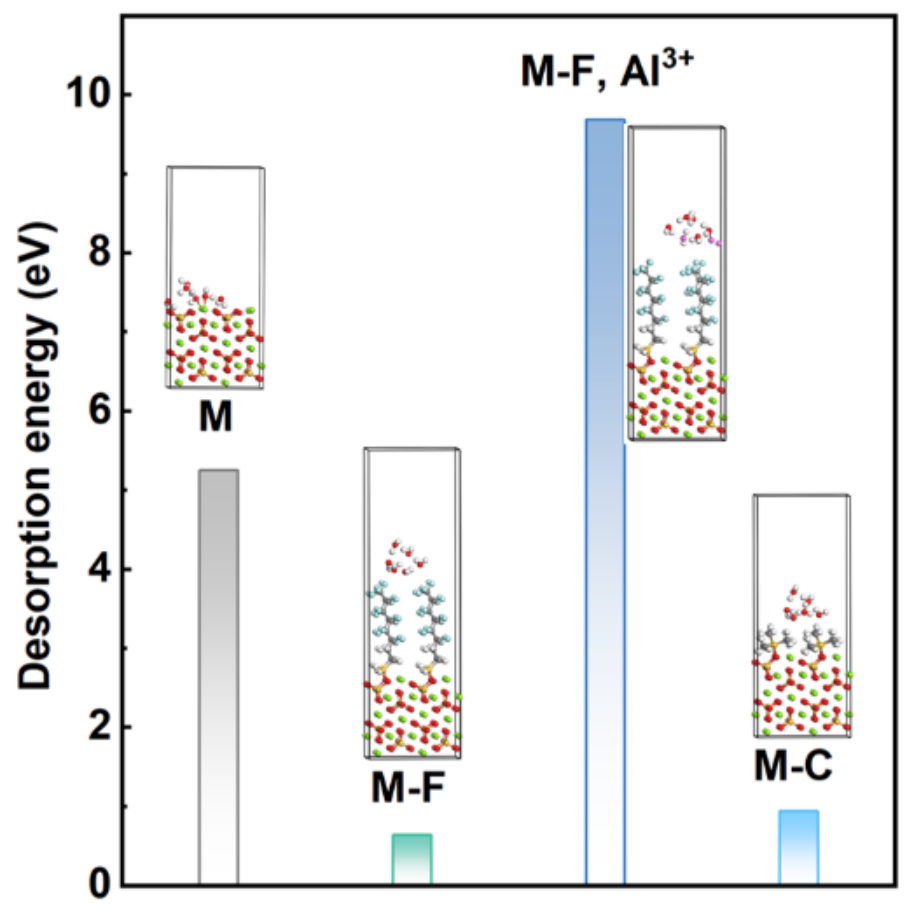

Fig. R14. Desorption energy of a hexameric water cluster on hydrophilic and hydrophobic mineral surfaces calculated by DFT simulations. (Fig. 2e)

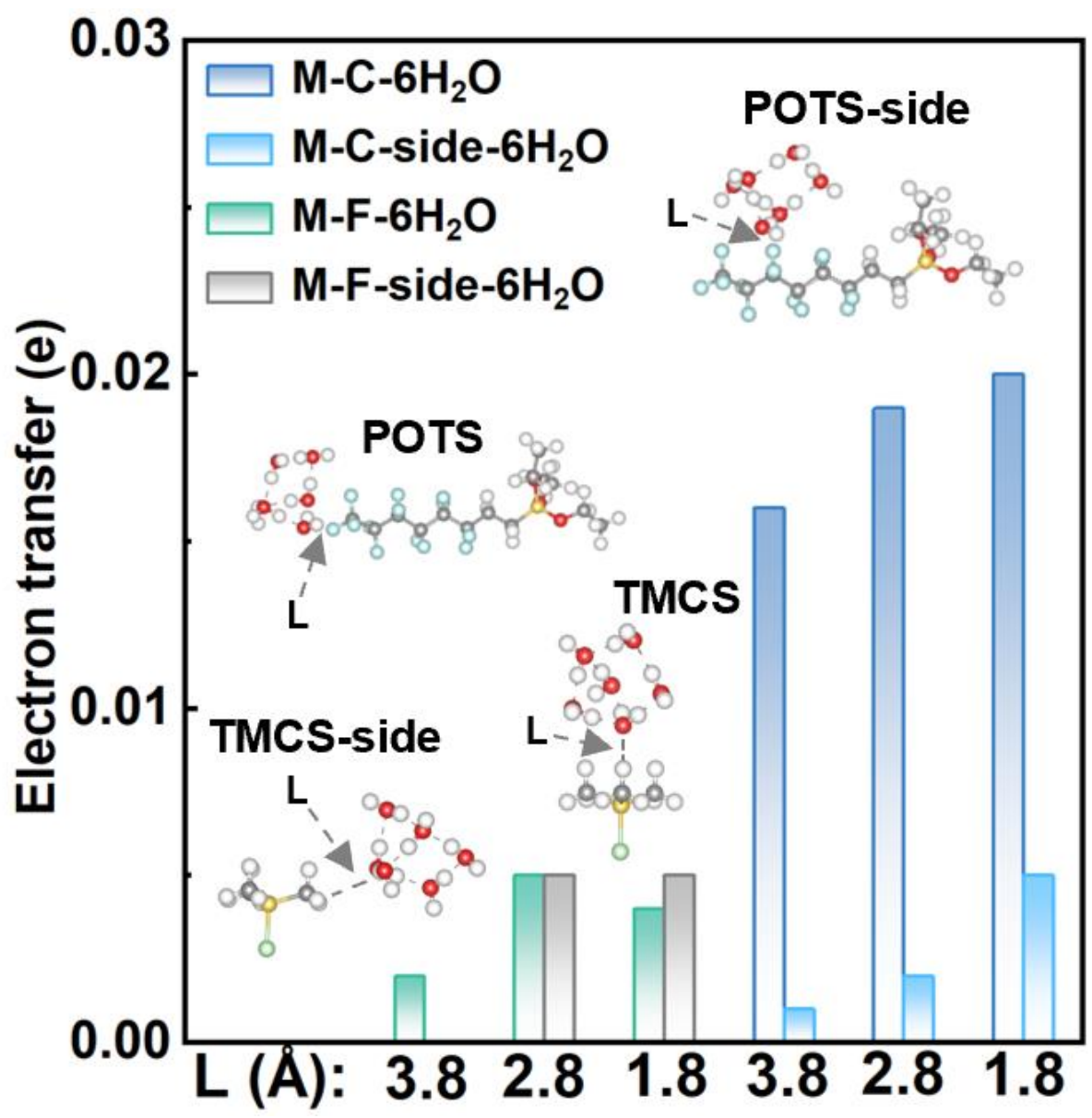

Fig. R15. Electron transfer between hydrophobic molecules and a hexameric water cluster as a function of configurations of hydrophobic molecules based on DFT simulations. (Supplementary Fig.14)

Pages 13-14, lines 234-250: “DFT calculations were performed to further clarify how interfacial contact affects CEC performance by analyzing electron transfer (Fig. 2d), desorption energy (Fig. 2e), and energy band structures (Fig. 2f). For all three samples, the amount of electron transfer increased as the contact distance with water decreased. M acted as an electron donor to water, in contrast to M-C and M-F, which were electron acceptors. Despite M having the greatest electron transfer, its high desorption energy (Fig. 2e, 5.25 eV) limited the completion of the CEC process, resulting in poor performance. In contrast, both M-C and M-F exhibited similarly low desorption energies, allowing easy detachment of water molecules to complete the CEC (Fig. 2e). Moreover, compared with M-F, M-C showed better electron transfer (Fig. 2d), which can be attributed to its favorable wettability, facilitating better contact with water (Fig. 2c) despite its weaker electron-withdrawing ability (Fig. 2f). This favorable balance between water approach and removal gave M-C a performance advantage over M-F. In addition, the contact position between the modifiers and the water cluster was also regulated (Supplementary Fig. 14), and the interaction between a single water molecule and the samples was further calculated (Supplementary Fig. 15-16), both of which

supported the positive role of moderately hydrophobic regulation in enhancing CEC performance."

(3) We agree that electric double layer (EDL) effects may influence interfacial interactions in aqueous media; however, in the present work, this aspect was only considered in a simplified manner through ion-involved desorption-energy calculations to explain the wettability-related changes under practical conditions, rather than as an independent focus for a detailed EDL study. We carefully discussed the relationship between ions and contact in the revised manuscript, and clarified the reason for the absence of detailed discussion on EDL. The related discussion in the manuscript has been revised accordingly.

Pages 14-15, lines 251-267: "Moreover, exchangeable cations in minerals and aqueous environments may influence the effective interfacial wettability of M-F under practical conditions. Because M-F has a more negative zeta potential (–6.9 to –35.2 mV) than M-C does (–3.0 to –23.7 mV), it is more susceptible to the adsorption of metal ions in water[36-38], including mineral-derived ions and environmentally relevant cations such as Al^{3+}, Ca^{2+} and Mg^{2+}**.** Such ion adsorption may shift the catalyst toward a more hydrophilic state in aqueous media, rather than the hydrophobic behavior suggested by air-based contact angle measurements. Consistently, the results of DFT calculations indicate that cation-associated conditions can increase the water cluster desorption energy (9.69 eV in Fig. 2e), implying that the aqueous environment may alter the apparent interfacial behavior of M-F and thereby partly suppress its CEC performance (Fig. 2g). In contrast, M-C, with its less negative surface potential, is expected to be less affected by such ion-induced perturbation. We therefore consider cation adsorption here as an auxiliary factor that may modify the effective hydrophobicity/hydrophilicity of the catalyst in water, which may further contribute to the better CEC performance of M-C relative to M-F under practical conditions. A systematic analysis of ion-specific adsorption or electric double-layer effects, however, is beyond the scope of the present study."

Comment 5: In experiments involving the degradation of MO in circulating water flow, S-C and S-F achieved significant degradation rates (77%, 69%) (Fig. 3c). The text mentions "negligible adsorption", yet Supplementary Fig. 24 explicitly states that removal of cationic dyes "is primarily attributed to adsorption". Why does the reaction mechanism differ for MO, which is also a cationic dye? And how can the contributions of CEC degradation and adsorption be quantitatively distinguished? Furthermore, Supplementary Fig. 23 and 24 only provide data for S-F, with data for S and S-C missing. This cannot demonstrate that S and S-C share the same degradation mechanism as S-F. Regarding the results in Fig. 3c, the authors claim that hydrophobicity exerts a more pronounced effect under weak mechanical stimulation, thereby conferring superior CEC performance to S-C. However, this finding is at odds with the observation that M-F exhibits greater hydrophobicity than M-C (Fig. 1b).

Reply: Thank you for your valuable observation and question. To provide a clearer response to this comment, each point is addressed individually below.

(1) Based on the zeta potential results, all types of minerals are negatively charged. Methyl orange (MO) dye belongs to anionic dyes [1–3], thus its adsorption by catalysts during CEC is negligible. Methylene blue (MB) [4], rhodamine B (RhB) [5], and crystal violet (CV) [6] dyes are cationic dyes, and thus are strongly adsorbed by the catalyst. We have added adsorption experiments of S, S-C and S-F on MO (Fig. R16) in the Supporting Information as follows.

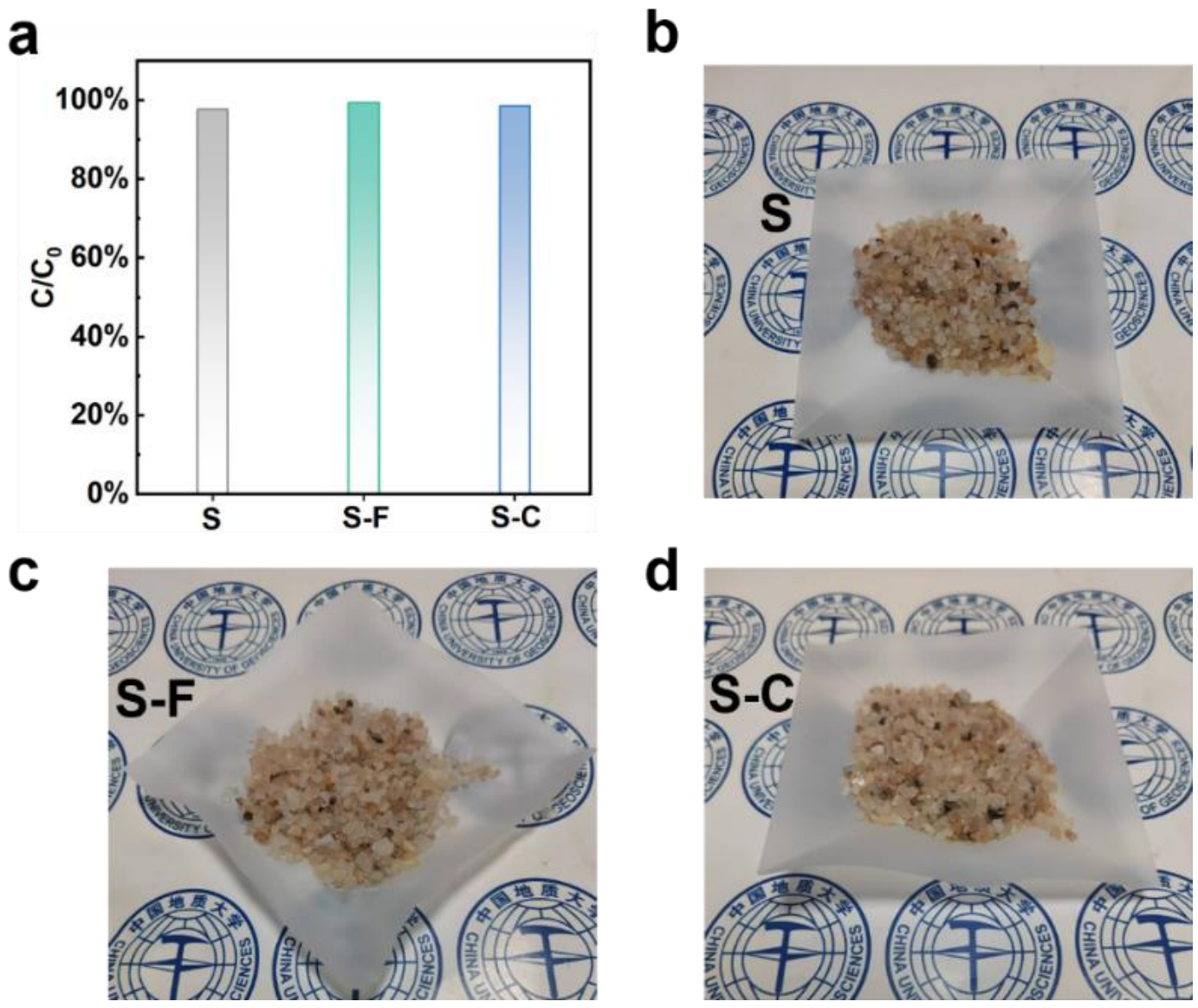

Fig. R16. Adsorption of MO by S, S-F, and S-C and the corresponding images after adsorption. (a) Comparison of MO absorbance before and after adsorption. (b) Image of S after adsorption of MO. (c) Image of S-F after adsorption of MO. (d) Image of S-C after adsorption of MO. (Supplementary Fig. 17)

Page 24, lines 357-359 in Supporting Information: “The adsorption capacity was quantified by immersing S, S-F, and S-C in MO solution for 48 h. As shown in Supplementary Fig 17, the adsorption of MO by the three types of stones was extremely weak.”

(2) The adsorption capacity was quantified by immersing S, S-C, and S-F in MB, RhB, and CV solutions for 48 h to achieve adsorption saturation. Adsorption of the three dyes by S-F was further supplemented in the Supporting Information as follows (Fig. R17). In addition, the degradation and adsorption of MB, RhB, and CV by S and S-C were also supplemented in the Supporting Information (Fig. R18).

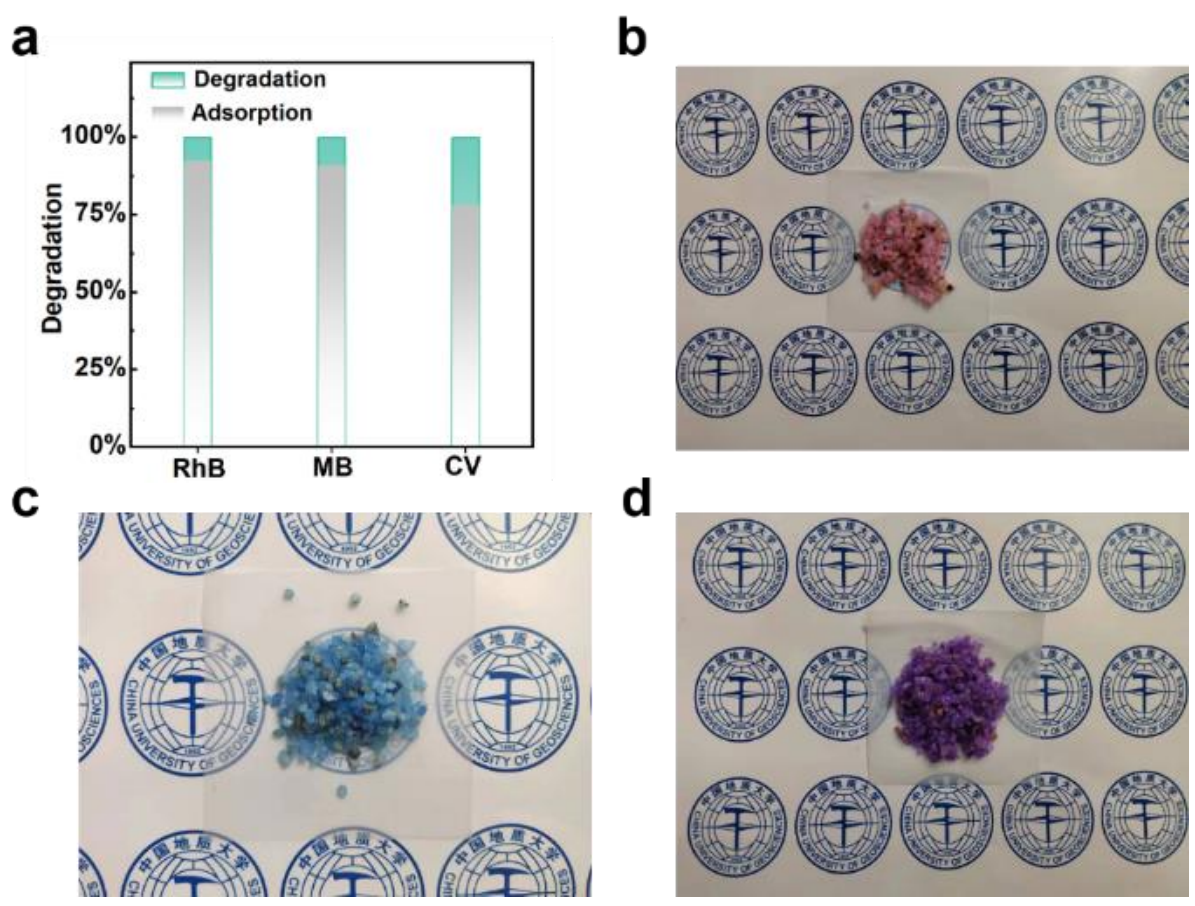

Fig. R17. Performance of S-F in degrading three cationic dyes. (a) Degradation and adsorption behavior of S-F toward the three dyes. (b) Image of S-F after treatment of rhodamine B (RhB). (c) Image of S-F after treatment of methylene blue (MB). (d) Image of S-F after treatment of crystal violet (CV). (Supplementary Fig. 18)

The **adsorption capacity of S** toward all three dyes was **higher than that of S-C (**Fig. R18-19**)**. The surface of natural minerals is enriched in hydroxyl groups, which can partially deprotonate in aqueous solution and generate negatively charged sites, thereby attracting cationic dyes via electrostatic interaction. After alkyl covalent modification, the surface negative charge density decreases, and adsorption of the three cationic dyes relies predominantly on van der Waals interactions, with significantly reduced Coulomb attraction, resulting in lower adsorption capacity. In contrast, the carbon-fluorine chains of POTS possess strong electron-withdrawing characteristics, and the adsorption capacity of M-F is only slightly influenced by the covalent modification.

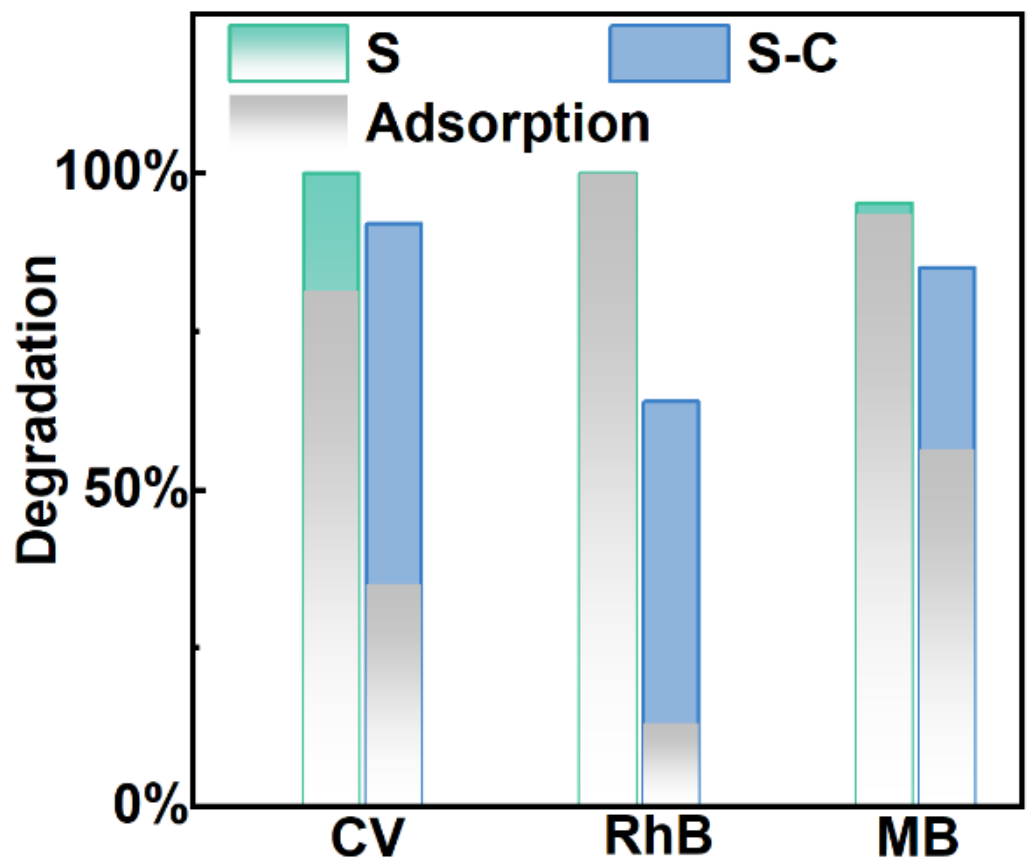

Fig. R18. Comparison of adsorption and degradation of MB, RhB, and CV by S and S-C.

(Supplementary Fig. 19)

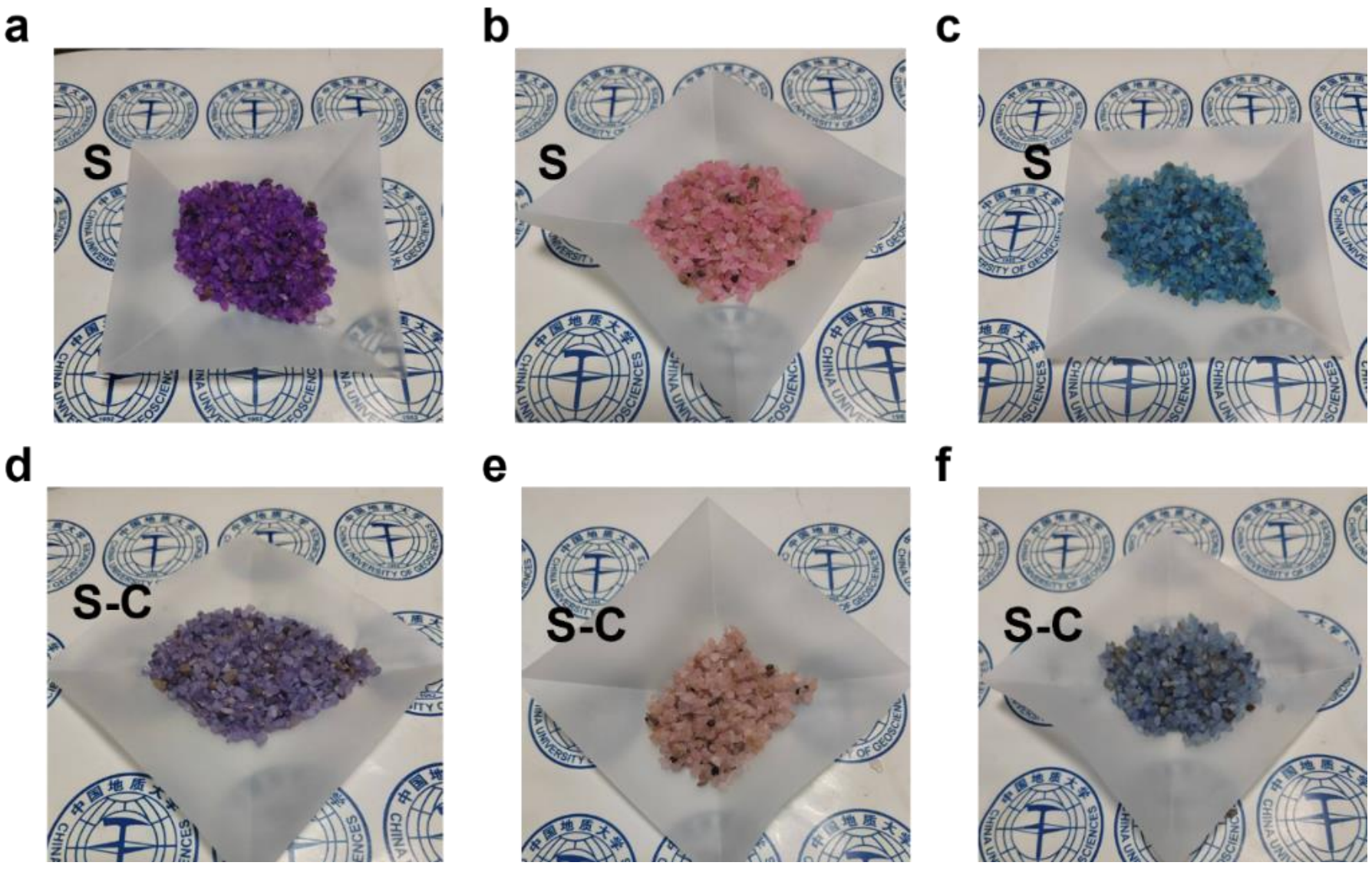

Fig. R19. Comparison of images after adsorption of the three dyes by S and S-C. (a) Image of S after treatment of crystal violet (CV). (b) Image of S after treatment of rhodamine B (RhB). (c) Image of S after treatment of methylene blue (MB). (d) Image of S-C after treatment of CV. (e) Image of S-C after treatment of RhB. (f) Image of S-C after treatment of MB. (Supplementary Fig. 20)

The original text in the Supporting Information has been revised as follows:

Page 25, lines 366-374 in Supporting Information: "The addition of 20 g S-F to 150 mL solutions of each dye (5 mg/L) resulted in >99.9% removal within 2 h. To distinguish adsorption from degradation, adsorption capacity was quantified by immersing S-F in MB, RhB, and CV solutions for 48 h to reach adsorption saturation. As shown in Supplementary Fig. 18a, the majority of dye removal was attributed to adsorption. This conclusion is further supported by Supplementary Fig. 18b-d, in which S-F exhibited chromatic changes corresponding to the respective dyes, confirming adsorption of cationic species onto the fluorinated surface during circulation. Consequently, these dyes were effectively removed from the aqueous system primarily through adsorption."

Page 26, lines 379-384 in Supporting Information: "The addition of 20 g S or S-C to 150 mL solutions of each dye (5 mg/L), respectively, and the corresponding removal

behaviors are presented in Supplementary Fig. 19. Adsorption capacity was quantified by immersing S and S-C in MB, RhB, and CV solutions for 48 h to achieve adsorption saturation. As shown in Supplementary Fig. 19-20, adsorption of all three dyes by S was greater than that by S-C (Supplementary Note 6)."

Page 11, lines 186-195 in Supporting Information: "As shown in Supplementary Fig. 19-20, removal of the three cationic dyes by S was almost entirely due to adsorption, which can be attributed to the abundant surface silicon hydroxyl groups (Si–OH) of the unmodified crystal sand (main component: silica). In near-neutral or mildly acidic aqueous solution, partial deprotonation of these hydroxyl groups generates a negatively charged surface[22], facilitating electrostatic adsorption of cationic dyes. Removal of the three dyes by S-C involved both CEC degradation and adsorption. Compared with S, the adsorption capacity of S-C toward the three cationic dyes decreased mainly due to the reduction in surface negative charge after grafting of alkyl groups, which weakened electrostatic attraction. Under these conditions, adsorption was dominated by van der Waals interactions."

(3) Thank you for pointing out this issue. The original wording was inaccurate and has been corrected to state that **moderate** hydrophobicity plays a more pronounced role under weak mechanical excitation.

The focus of the manuscript is that **moderated hydrophobicity** enhances CEC performance. In Fig. 3c, it was clarified that **under weak mechanical stimulation**, the **moderate hydrophobicity of M-C** exerts a more pronounced CEC than M-F. A parameter R was introduced to quantify this effect, defined as the fold enhancement in CEC performance under ultrasonic (U) and circulating water-flow (C) conditions. For S-C, R_U =7.2 and R_C = 12.8; for S-F, R_U = 5.4 and R_C =10.5. These values indicate that moderate hydrophobicity exerts a more pronounced beneficial effect under weak mechanical excitation. The stronger hydrophobicity of M-F further reduces effective catalyst-water interaction under weak-force conditions, thereby decreasing electron cloud overlap and suppressing CEC performance.

References:

[1] Abdel-Aziz AB, et al. Bio-inspired adsorption sheets from waste material for anionic methyl orange dye removal. *SN Appl. Sci* **5**, 371 (2023).

[2] Sadykov TT, Syakaev RS, Andriianova AN, Mustafin AG. Adsorption of anionic methyl orange dye by polyaniline, poly(o-methylaniline) and poly(o-methoxyaniline). *Int. J. Chem. Kinet* **56**, 81-95 (2023).

[3] El-Naggar NE-A, Hamouda RA, Saddiq AA, Alkinani MH. Simultaneous bioremediation of cationic copper ions and anionic methyl orange azo dye by brown marine alga Fucus vesiculosus. *Sci. Rep* **11**, 3555 (2021).

[4] Amri AE, et al. Adsorption of a cationic dye (Methylene bleu) by Typha Latifolia: Equilibrium, kinetic, thermodynamic and DFT calculations. *Chem. Data Collect* **38**, 100834 (2022).

[5] Alamier WM, D Y Oteef M, Bakry AM, Hasan N, Ismail KS, Awad FS. Green Synthesis of Silver Nanoparticles Using Acacia ehrenbergiana Plant Cortex Extract for Efficient Removal of Rhodamine B Cationic Dye from Wastewater and the Evaluation of Antimicrobial Activity. *ACS Omega* **8**, 18901-18914 (2023).

[6] Algarni TS, Al-Mohaimeed AM, Al-Odayni A-B, Abduh NAY. Activated Carbon/$ZnFe_2O_4$ Nanocomposite Adsorbent for Efficient Removal of Crystal Violet Cationic Dye from Aqueous Solutions. *Nanomaterials* **12**, 3224 (2022).

Comment 6: The author attributes the improvement in Olivine-F performance to the addition of more -F groups, and this needs to be supported by experimental evidence.

Reply: Thank you for this important comment. It is clarified that the superior performance of olivine-F is not attributed to a higher amount of –F groups, but rather to the presence of abundant residual oxygen-containing groups on its surface. XPS analysis of the ten M-F samples (Fig. R20) shows that olivine-F possesses the lowest relative fluorine content and the highest relative oxygen content among the tested minerals, yet exhibits the best CEC performance. This observation demonstrates that higher fluorine loading is not responsible for enhanced activity. Instead, the residual

oxygen-containing groups on the modified olivine surface facilitate water-catalyst interaction, thereby improving the effective contact state between grafted carbon-fluorine chains and water (Fig. R21). A more detailed discussion of this structure-performance relationship is provided in the **response to Comment 3**.

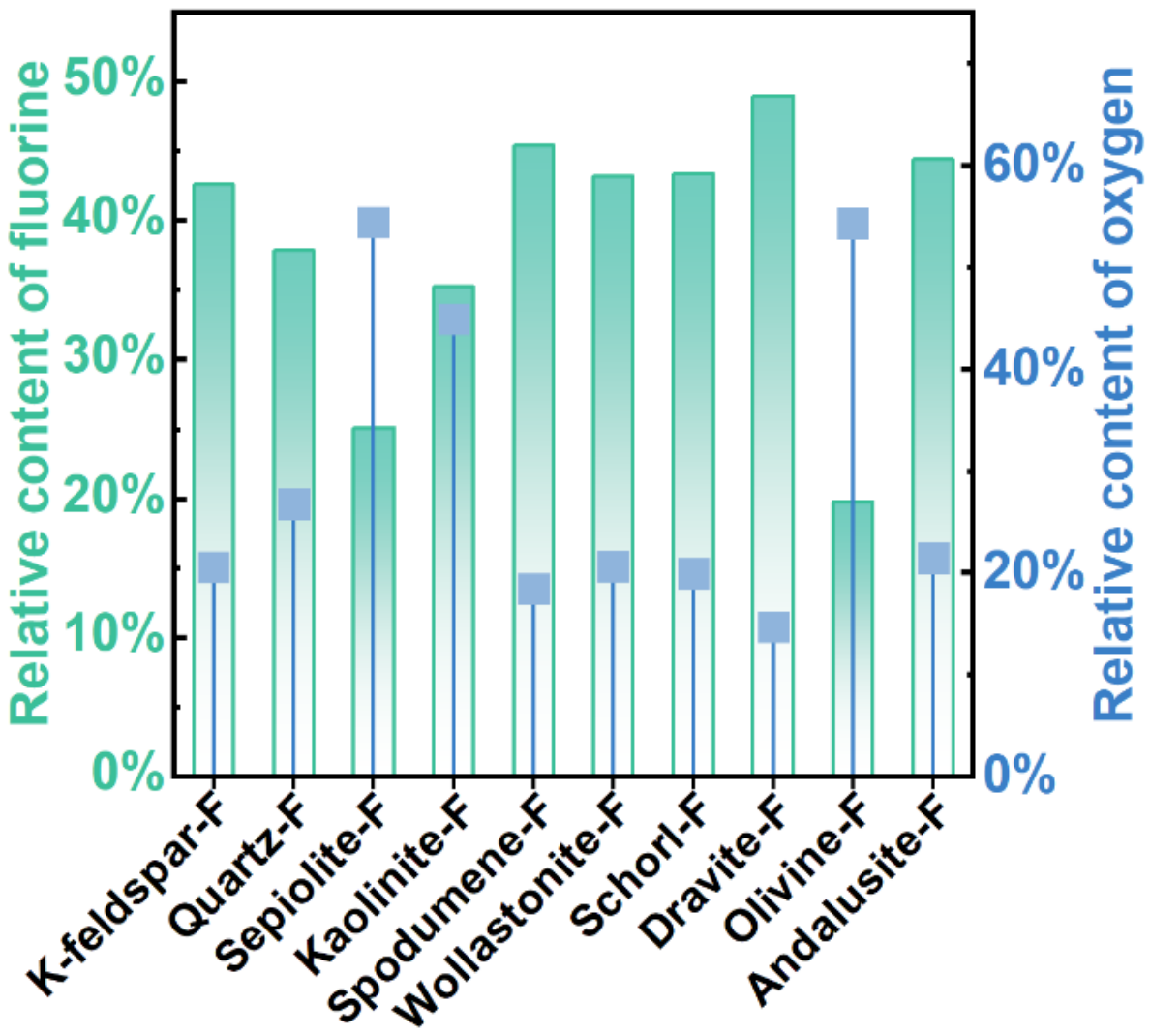

Fig. R20. Relative fluorine content and relative oxygen content of M-F surfaces measured by XPS. (Supplementary Fig. 6)

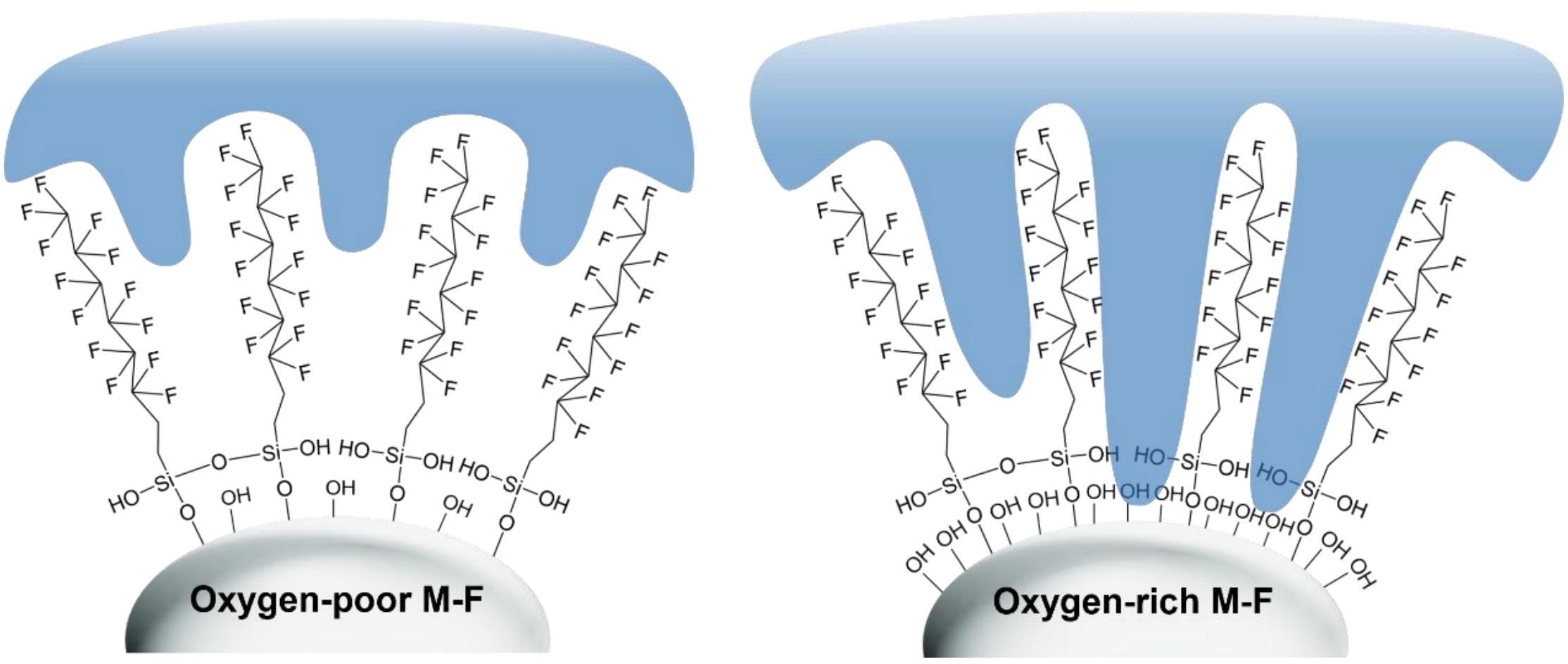

Fig. R21. Schematic diagram illustrating the contact state between water and the surfaces of oxygen-rich M-F and oxygen-poor M-F after M is modified with sufficient modifier. Oxygen groups are represented by hydroxyl groups. (Supplementary Fig. 8)

Comment 7: Figure 2d-h: The author conducted a detailed analysis of the relationship between the catalyst's acquisition of electrons and its hydrophilicity and

electronegativity during the WOR process, but failed to analyze the relationship between the catalyst's loss of electrons and these factors during the ORR process. There is a lack of evidence.

Reply: We thank the reviewer for this thoughtful and important comment. We fully agree that, in a complete CEC reaction process, both the water oxidation reaction (WOR, catalyst receiving electrons from H_2O) and the oxygen reduction reaction (ORR, catalyst transferring electrons to dissolved O_2) are generally involved. In the present study, however, our primary objective was to clarify how catalyst hydrophobicity regulates interfacial contact with water, because water is the dominant liquid phase, the direct medium receiving mechanical force, and the essential reactant governing catalyst-liquid contact behavior during CEC. By comparison, dissolved O_2 is present at a much lower concentration and participates within the water phase, so its contact with the catalyst is secondary to the catalyst-water interaction in the context of our central mechanistic question.

Accordingly, Fig. 2d–h was designed to focus on the relationship between catalyst wettability/electronegativity and the catalyst-H_2O interaction, which is considered the most fundamental and primary step to be clarified first. We agree that a parallel mechanistic analysis of the catalyst's electron loss during ORR would further enrich the mechanistic understanding. At the present stage, our experimental results (Fig. 1) already reflect the overall influence of all aqueous components, including dissolved oxygen, ions, and others, on catalyst contact behavior and CEC performance, but they do not allow exhaustive distinction of the mechanistic role of each individual species in this study.

Therefore, it is respectfully considered that the absence of a dedicated ORR-path analysis does not affect the main conclusion of this study, namely that regulating catalyst–water interfacial wettability is a key factor controlling CEC performance. To avoid overinterpretation, clarification has been added in the revised manuscript to explicitly state this limitation and to indicate that the detailed role of dissolved O_2 in the interfacial electron-transfer pathway warrants further investigation in future work.

Page 15, lines 268-270: "Moreover, although dissolved O_2 participate in the ORR, we focused only on catalyst-water interactions, as water is the main medium directly involved in mechanical contact and the most fundamental factor governing CEC performance.[39,40]"

Comment 8: The article provides new ideas for designing CEC catalysts that are non-fluorinated, green, and suitable for natural environments. However, the manuscript makes extensive use of M-F research data and lacks M-C research data, including but not limited to: Fig. 1d, 1f, and 1g, Supplementary Fig. 6-14, and Supplementary Fig. 20.

Reply: Thank you for your insightful observation. We have added the M-C data corresponding to original Fig. 1d and Fig.1g in revised manuscript in Fig. 1b–c (Fig. R22-23) and Fig.1g (Fig. R24) as follows.

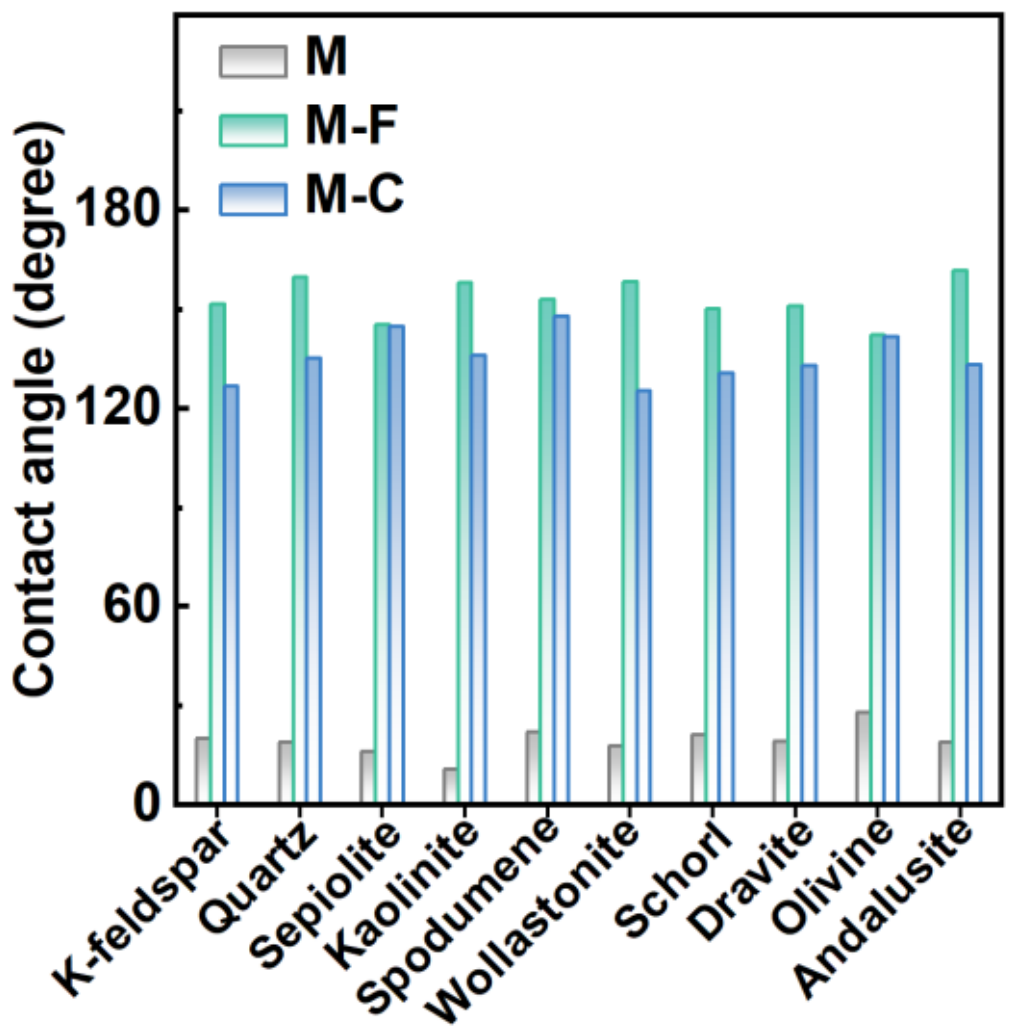

Fig. R22. Contact angles of three types of minerals with different hydrophilicity/hydrophobicity. (Fig. 1b)

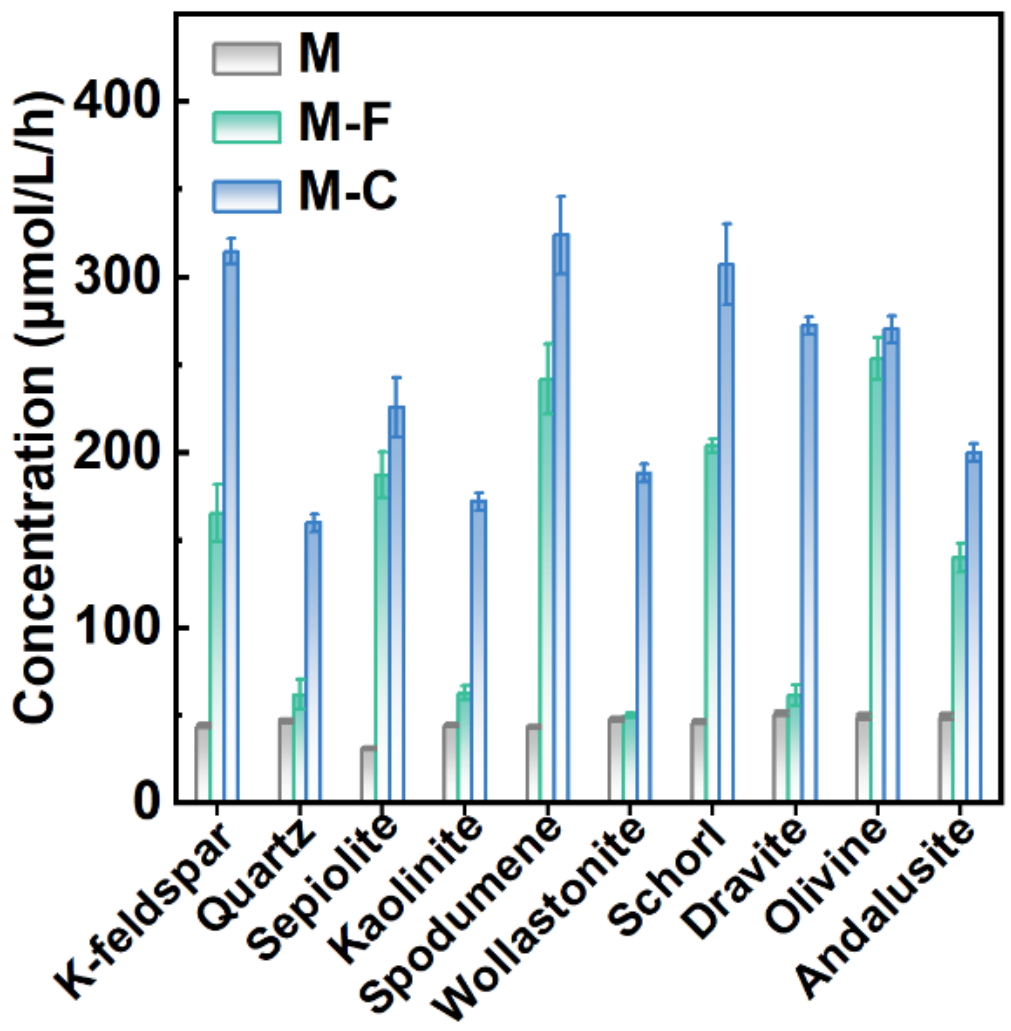

Fig. R23. H_2O_2 production of three types of minerals with different hydrophilicity/hydrophobicity through CEC. (Fig. 1c)

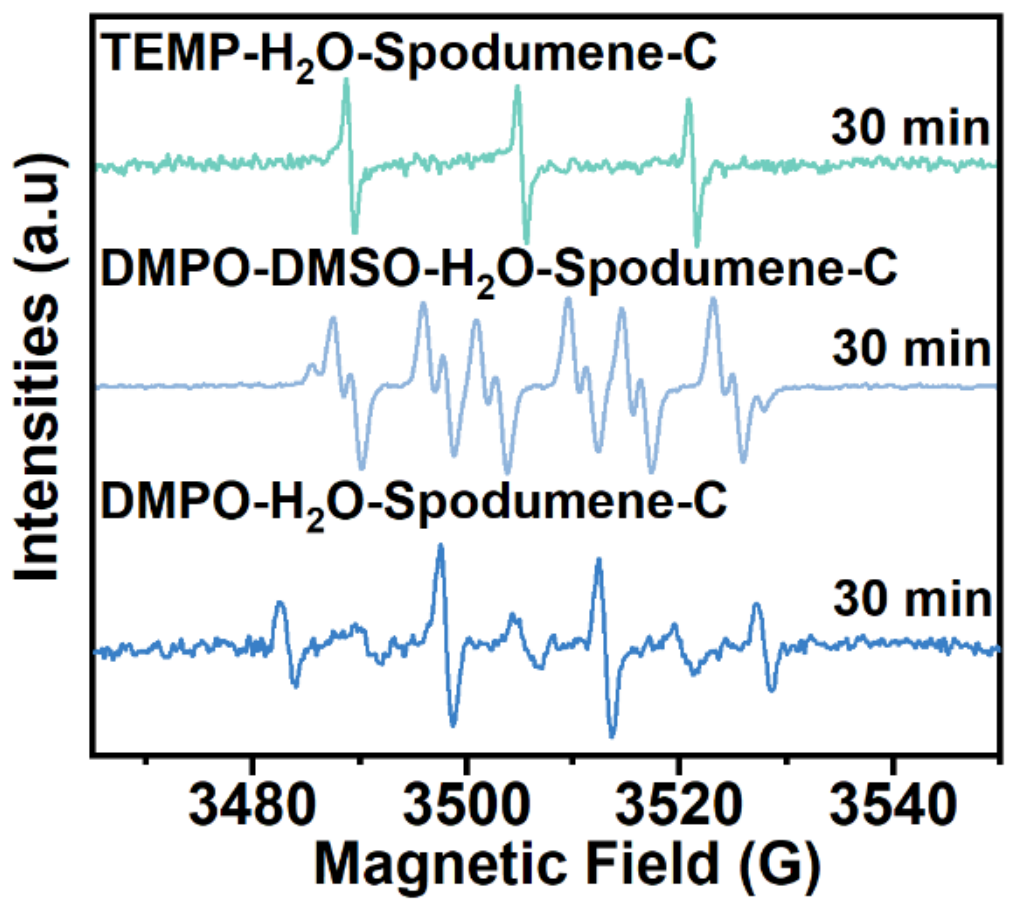

Fig. R24. Electron paramagnetic resonance (EPR) spectra of spodumene-C under ultrasonication for 30 min. (Fig. 1g)

In addition, part of the detailed characterization of M-F (original Supplementary Fig. 4-14, 16-17) has been streamlined to improve focus and coherence. The primary objective of this study is to establish the hydrophobicity-structure-CEC activity relationship, while the development of the green, non-fluorinated catalyst M-C represents a secondary innovation. Excessive M-F characterization not directly reinforcing these central conclusions was therefore reduced to enhance clarity and data

concentration.

Regarding Fig. 1d-f and Supplementary Fig. 20, these datasets were retained because they demonstrate that improving mineral-water contact, enhances the CEC performance, thereby reinforcing the conclusion that **moderate hydrophobicity benefits CEC**. The M-F platform was selected for gradient studies due to its **broader hydrophobicity range**, enabling clearer observation of contact-angle-performance relationships. Further clarification has been provided in the responses to Comments 2 and 3.

Comment 9: Please add XPS data for Fe2p before and after fluorination in Supplementary Fig. 7. In addition, for Supplementary Fig. 11a, what is the reason for the significant change in the ratio of $-CF_2$, $-CF_3$ signal peaks located near 292, 294 eV before and after the reaction?

Reply: Thank you for your valuable observation and question. We have supplemented the XPS spectra of Fe2p of olivine before and after fluorination as follows. As shown in Fig. R25, the binding energy of the Fe spectra in olivine did not change noticeably before and after fluorination.

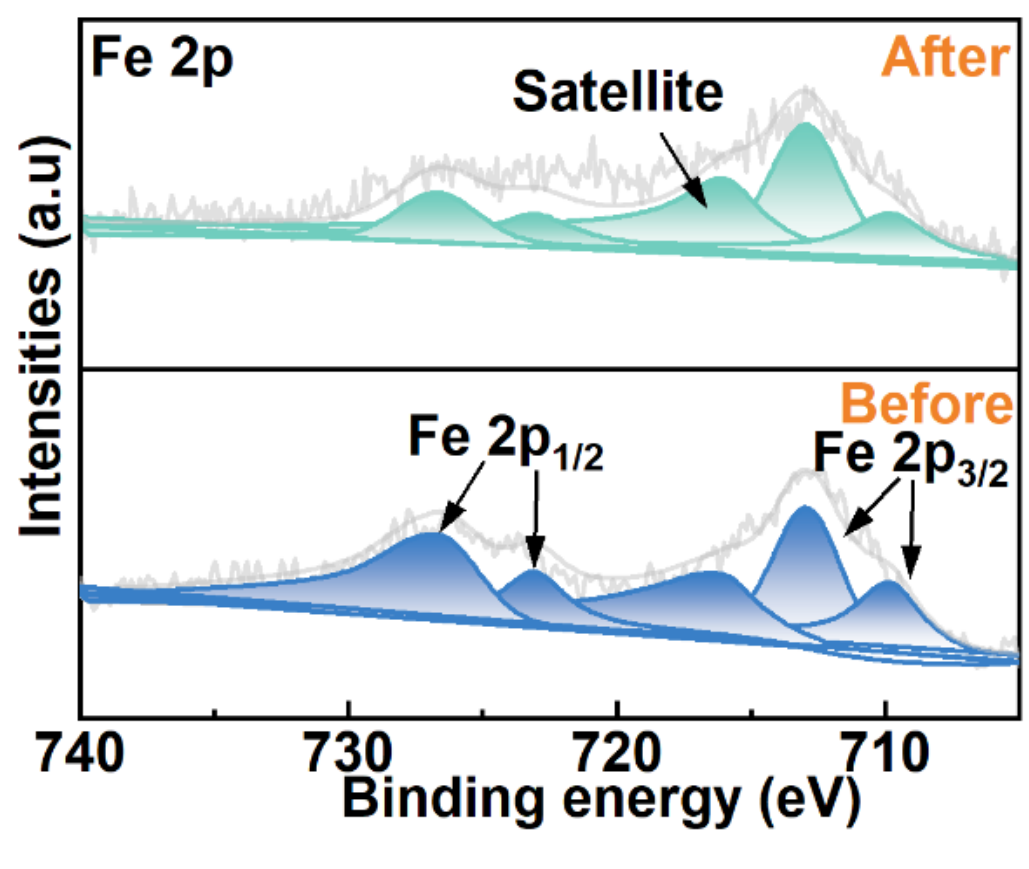

Fig. R25. X-ray photoelectron spectroscopy (XPS) spectra of the Fe 2p region for olivine and olivine-F.

Since no evident shift in the binding energies of $–CF_2$ and $–CF_3$ was observed before and after reaction, the covalent bonding state and functional groups remained unchanged. The variation in relative peak intensity is therefore attributed to changes in

the orientation and exposure angle of the grafted carbon-fluorine chains rather than to chemical transformation. Prior to the CEC reaction, terminal $–CF_3$ groups were preferentially oriented outward. After CEC, partial reorientation of the grafted chains likely occurred, resulting in greater exposure of $–CF_2$ groups [1]. For example, when the tilt angle of $–COO(CH_2)_2(CF_2)_8F$ silane changes from 45° to 5°, the measured XPS fluorine concentration increases from 53.1% to 62.4%, accompanied by evident surface segregation [2].

References:

[1] V. BINDU, K. SMITHA, T. PRADEEP. Tilted orientation of molecular chains at the surface of a series of perfluoropolyether liquids: an X-ray photoelectron spectroscopic investigation. *Mol. Phys* **93**, 85-94 (1998).

[2] Beamson G, Alexander MR. Angle-resolved XPS of fluorinated and semi-fluorinated side-chain polymers. *Surf Interface Anal* **36**, 323-333 (2004).

Comment 10: Fig. 1g and SI Fig. 17: How are •OH and $•O_2^-$ quantified? And how can the conclusion "•OH plays a more significant role than $•O_2^-$ in the formation of H_2O_2" be drawn based on the results of free radical capture?

Reply: Thank you for your valuable suggestion and question. The trends in radical production indicate that •OH and $•O_2^–$ represent two distinct pathways for H_2O_2 formation. Because the variation in •OH yield is consistent with that of H_2O_2, whereas the increase in $•O_2^–$ does not lead to higher H_2O_2 production, •OH is inferred to be the dominant pathway for H_2O generation. The quantitative methods for •OH and $•O_2^–$ are described as follows.

Hydroxyl radicals (•OH): 10 mg of the silicate mineral catalyst was added into 5 mL of DMPO solution (100 mM). The mixture was stirred and sonicated for 30 min, and aliquots were withdrawn for EPR analysis at 30 min. Superoxide radical ($•O_2^–$): 10 mg of the silicate mineral catalyst was added into 5 mL of DMPO/DMSO solution (containing 100 mM DMPO and 1 mM DMSO). The mixture was stirred and sonicated for 30 min, and aliquots were collected for EPR measurement at 30 min. Singlet oxygen

radical ($^1O_2$): 10 mg of the silicate mineral catalyst was added into 5 mL of TEMP solution (100 mM). The mixture was stirred and sonicated for 30 min, and aliquots were collected for EPR analysis at 30 min.

The •OH concentration was calculated by measuring the fluorescence intensity at an excitation wavelength of 315 nm using a sodium hydroxide solution of terephthalic acid in a fluorescence spectrophotometer (F97XP, China).

The yield of $•O_2^-$ was measured by the degradation of Nitrotetrazolium Blue chloride (NBT) solution in the UV absorption range of 200 nm–500 nm.

By comparing the M-F H_2O_2 production performance sequence (Fig. R26) and ROS quantification (Fig. R27) sequence of the three M-F samples (olivine-F, schorl-F, and spodumene-F) with the best CEC performance in Fig. 1d, it was found that the order of H_2O_2 generation was olivine-F > spodumene-F > schorl-F. In the quantitative experiments, the formation order of •OH was consistent with that of H_2O_2. However, the order of $•O_2^-$ generation was olivine-F > schorl-F > spodumene-F. Schorl-F exhibited a higher production of $•O_2^-$ but lower production of H_2O_2 than spodumene-F, indicating that •OH plays a more significant role in the synthesis of H_2O_2 than $•O_2^-$.

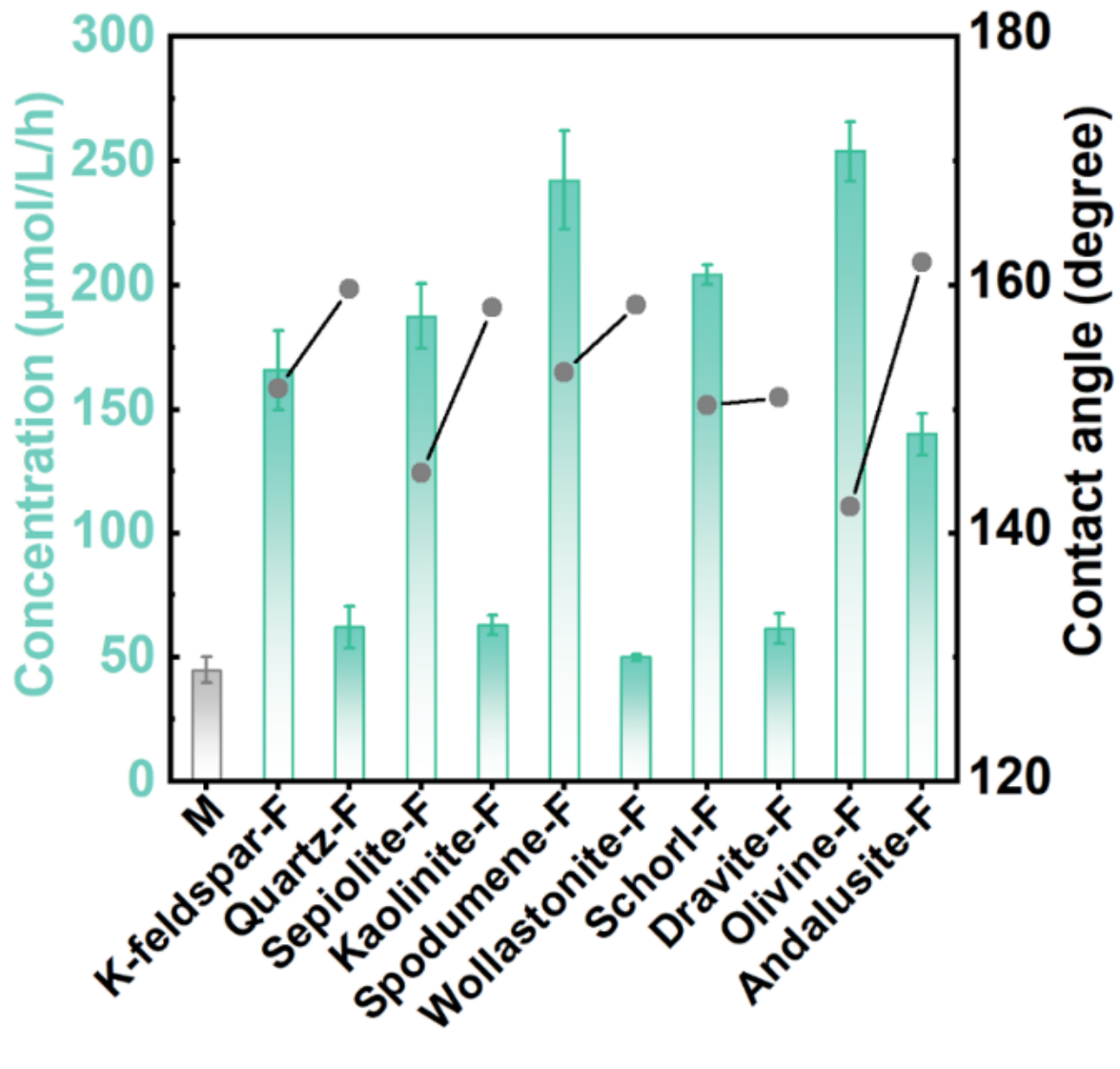

Fig. R26. H_2O_2 production (using 10 samples for error bar analysis) and contact angles of M-F. (Fig. 1d)

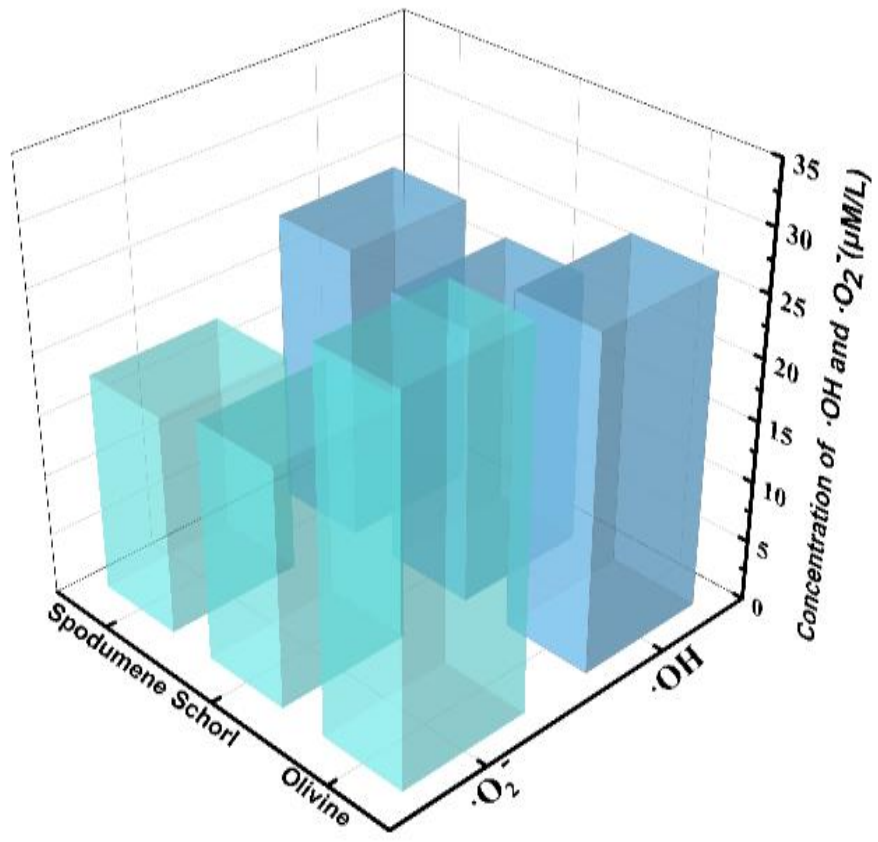

Fig. R27. Free-radical quantitative experiment.

Comment 11: SI Fig. 10: Why is the effect of the first cycle significantly worse than that of the subsequent cycles? Please explain.

Reply: Thank you for your insightful observation and question. The relevant discussion is described as follow.

CEC performance improved in subsequent cycles compared to the initial cycle (Fig. R28). This enhancement is attributed to the modified olivine achieving smaller particle size distribution after cycling as large particles fragmented into smaller ones (Fig. R29 c–f).

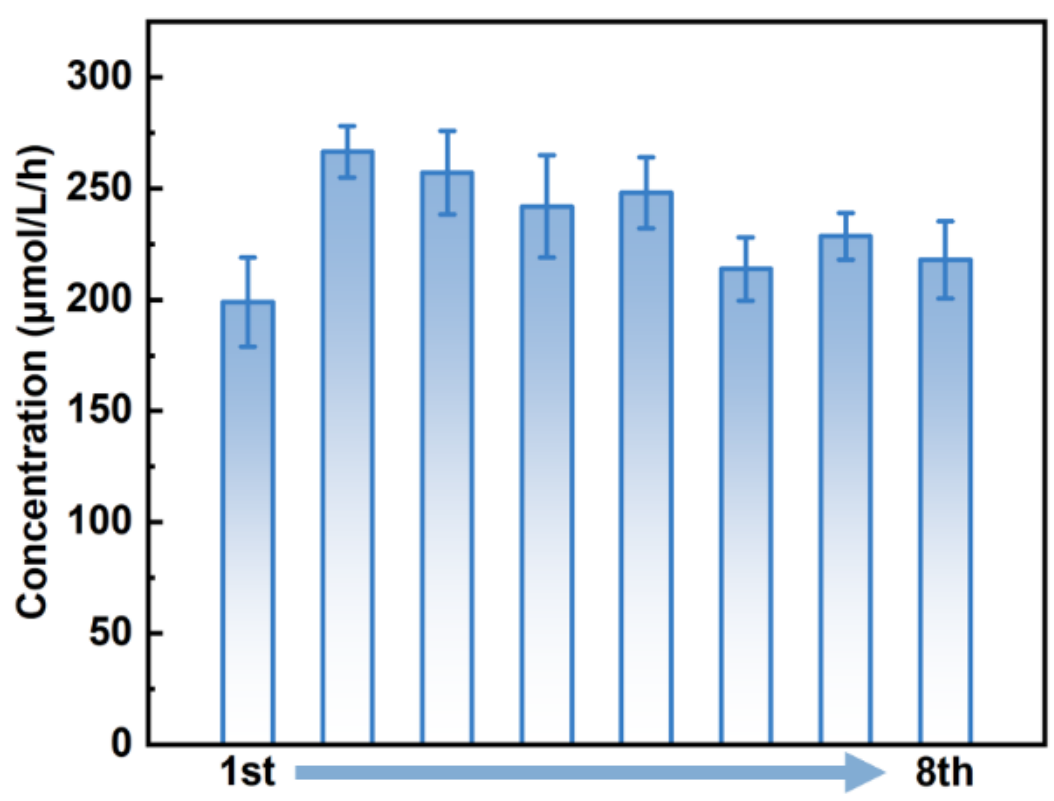

Fig. R28. Cycling performance of olivine-F.

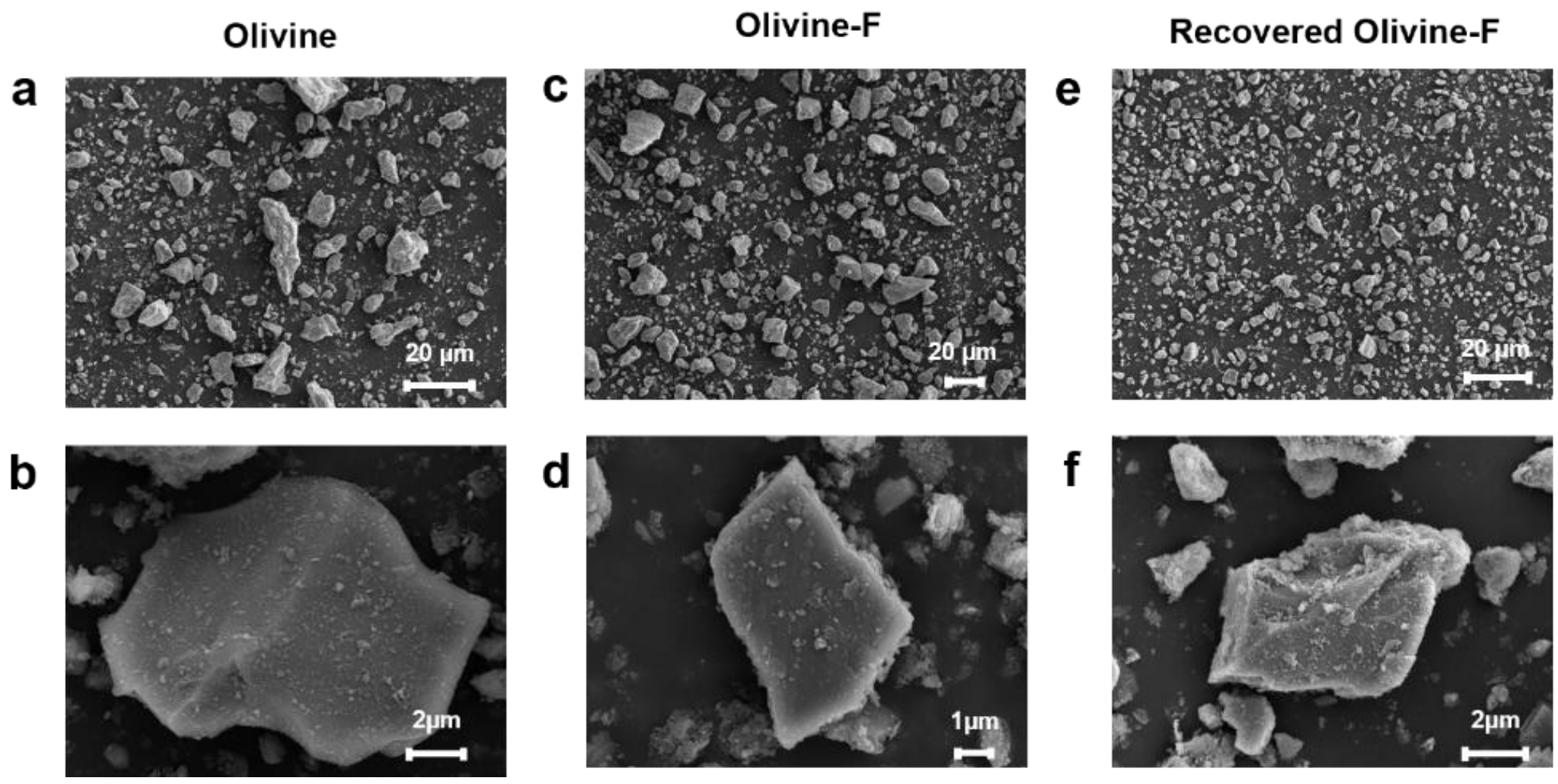

Fig. R29. SEM images of olivine before and after modification and cycling. (a and b) SEM images of olivine. (c and d) SEM images of olivine-F. (e and f) SEM images of recovered olivine-F.

Comment 12: Compared with the previously published works that produce H_2O_2 through CEC, the performance of H_2O_2 production in this study is not outstanding. Moreover, fluorine-containing materials such as PTFE and FEP have high stability and can be used for catalysis for a long time without causing excessive environmental pressure. The authors should more clearly elaborate on the uniqueness and innovation of the non-fluorine catalyst system in this work.

Reply: We thank the reviewer for this valuable comment. We agree that the innovation of the present work should be clarified beyond a simple comparison of absolute H_2O_2 production rates. Indeed, the main contribution of this study is not to claim the highest H_2O_2 yield among all CEC systems, but to demonstrate a new design strategy for CEC catalysts that does not rely on fluorinated materials.

Conventional CEC systems have predominantly employed fluorinated materials such as PTFE and FEP because of their strong electron-withdrawing ability and excellent durability. However, our results show that, on mineral surfaces, excessive hydrophobicity associated with fluorinated groups can reduce the effective contact probability between catalyst and water, which becomes particularly detrimental under weak-force conditions. In this context, the non-fluorine M-C catalyst is unique because

it achieves a more favorable balance between water approach and water removal, thereby enabling more efficient interfacial contact and stronger CEC activity than the fluorinated M-F counterpart.

Thus, the key novelty of this work is that it challenges the conventional assumption that stronger fluorination necessarily leads to improved CEC performance. Instead, we show that moderate hydrophobicity and optimized catalyst-water contact are more critical than simply maximizing electron-withdrawing ability, especially for mineral-based CEC systems. This provides a new design principle for future CEC catalysts.

In addition, the non-fluorine catalyst developed here is particularly relevant for weak mechanical excitation and natural environmental conditions. Unlike many previous studies that focused primarily on ultrasonic excitation, we further demonstrate that the mineral-based non-fluorine catalyst can operate under circulating water flow and even in a natural river environment. This extends CEC from a laboratory phenomenon toward a potentially self-powered environmental process. Therefore, the uniqueness of this work lies in the combination of (i) a contact-regulation mechanism (**main**), (ii) a non-fluorine catalyst system, and (iii) validation under realistic weak-force and natural hydrodynamic conditions.

We have revised the manuscript accordingly to better emphasize these aspects:

Page 25, lines 438-458: "This work establishes hydrophobicity regulation as a contact-optimization strategy for contact-electro-catalysis (CEC) at mineral-water interfaces. Rather than simply pursuing stronger electron-withdrawing ability, our results show that efficient mineral-based CEC depends on balancing interfacial wettability to coordinate water approach and removal during contact. As a material demonstration of this strategy, the moderately hydrophobic alkyl-modified mineral catalyst (M-C) outperformed both the hydrophilic raw mineral (M) and the more hydrophobic fluorinated counterpart (M-F), highlighting that moderate hydrophobicity is more favorable for CEC than either insufficient or excessive water affinity. Importantly, this advantage became even more pronounced under weak-force conditions, where excessive hydrophobicity more severely limits effective water-catalyst contact and

makes balanced interfacial transfer especially critical.

Beyond catalyst design, this strategy remained effective under environmentally relevant conditions. The operation of alkyl-modified stone under circulating water flow and in a natural river shows that non-fluorinated mineral catalysts can sustain self-powered CEC driven by ambient hydrodynamic energy, extending the CEC beyond that of conventional fluorinated laboratory systems. More broadly, these findings suggest that hydrophobicity-regulated mineral-water interfacial CEC may represent a previously underappreciated pathway linking reactive oxygen species generation with surface reactions, organic matter transformation and microbial niche differentiation in natural aquatic environments.”

Page 24, lines 425-428: “In addition, because the grafted functional groups are covalently bound to the mineral surface, leaching-related effects are expected to be limited under the tested conditions, but this aspect should be explicitly evaluated in future work.”

Reviewer #3: This manuscript reports a systematic investigation of how interfacial hydrophobicity governs contact-electro-catalysis (CEC) on natural silicate minerals. By introducing alkyl (–CH_3) surface groups, the authors demonstrate that moderate hydrophobicity maximizes hydrogen peroxide generation and ROS formation, outperforming both hydrophilic and fluorinated mineral surfaces. The work combines mineral screening, surface chemistry, theoretical analysis, density functional theory, weak-force flow experiments, and a rare field-scale deployment in a natural river, including genome-resolved metagenomic analysis of surface-associated microbial communities. The study addresses an important sustainability challenge in CEC by reducing reliance on fluorinated polymers and provides a conceptual framework for catalyst design under weak mechanical excitation. Below are some comments in detail:

Thank you for your insightful and constructive comments on our manuscript. We have carefully revised the manuscript to clarify the logical relationships among several datasets and to restructure parts of the text for greater accuracy and readability. Detailed point-by-point responses are provided below. It is hoped that these revisions adequately address the concerns raised.

Comment 1: While the field deployment and metagenomic results are compelling, claims related to “ecological restoration” and ecosystem-scale regulation are not fully supported. The data demonstrate microbial selection and organic matter attenuation, but not ecosystem recovery. The authors should temper these claims throughout the Abstract, Discussion, and Conclusion.

Reply: We agree with the reviewer that the present data demonstrate selective microbial enrichment and attenuation of organic matter, indicating that natural water flow can initiate CEC-driven surface reactions on mineral substrates, but do not directly establish ecosystem-scale restoration or long-term ecological recovery.

Accordingly, the language in the Abstract, Discussion, and Conclusion has been revised to moderate these claims. Our intention was to highlight the mechanistic relevance of CEC-driven interfacial ROS generation in influencing microbial communities and organic matter transformations under natural flow conditions. The

text has been revised to clearly distinguish between demonstrated effects (microbial selection and organic attenuation) and broader ecological implications that require further investigation.

Page 2, lines 35-39: "Together, these findings establish hydrophobicity regulation as an effective contact-optimization strategy for CEC, providing a basis for non-fluorinated catalyst design and for harnessing naturally available mechanical energy in environmentally relevant interfacial processes."

Page 25, lines 454-458: "More broadly, these findings suggest that hydrophobicity-regulated mineral-water interfacial CEC may represent a previously underappreciated pathway linking reactive oxygen species generation with surface reactions, organic matter transformation and microbial niche differentiation in natural aquatic environments."

Comment 2: Comparisons between ultrasonication and flow-driven CEC rely on relative performance metrics without rigorous normalization of mechanical energy input. A clearer discussion or estimation of energy densities and force regimes is required to substantiate claims regarding weak-force efficiency.

Reply: Thank you for your valuable suggestion. Circulating water flow has much lower energy density than ultrasonic conditions. The energy density and force regimes of ultrasonication and water flow impact have been estimated and compared in the Supporting Information as follows.

Page 17, lines 300-302: "The power density of the circulating water flow is 0.25 times that of ultrasonication (Supplementary Note 5)."

Page 10, lines 167-183 in Supporting Information: "For the ultrasonic cleaning machine, the energy density can be estimated using formula (1).

$$E\ (W/cm^2) = \frac{P\ (W)}{A\ (\mathrm{cm}^2)} \tag{1}$$

E represents the energy density, P represents the effective transmission power, and

A represents the emission area. By measuring the transmitted power (300W) and the emission area (480 cm^2) of the ultrasonic device, the energy density of the ultrasonic cleaning machine was calculated to be 6250 W/m^2.

For the peristaltic pump, the energy density can be estimated using formulas (2-3).

$$P_h\ (W) = \rho g Q H \quad (2)$$

$$I\ (w/cm^2) = \frac{P_h}{A} \quad (3)$$

P_h represents hydraulic power, ρ represents the liquid density, g is the gravitational acceleration, Q represents flow rate, H is head, I represents energy density, and A represents the scouring area. By measuring Q= 1000 ml/min, the inner diameter of the outlet d = 8 mm, and the scouring area A ≈ 10.6 cm^2, Ph was calculated to be 1.67 W, and I was calculated to be 1571 W/ m^2. The energy density of the peristaltic pump is much lower than that of the ultrasonic cleaning machine, indicating that the circulation flow impact force of the peristaltic pump is relatively weak."

Comment 3: The DFT calculations treat single water molecules interacting with mineral surfaces, whereas real CEC interfaces involve hydrated clusters and dynamic water networks. These limitations should be explicitly acknowledged in the Discussion, or need some further work to strengthen this part.

Reply: We thank the reviewer for this valuable comment. We have further supplemented the DFT calculations regarding the water cluster and different conformations of modification groups. It was found that the water cluster (Fig. R1–2) and the different conformations of modified group (Fig. R3) did not affect the advantage of the moderate hydrophobicity of M-C in contacting more effectively with water.

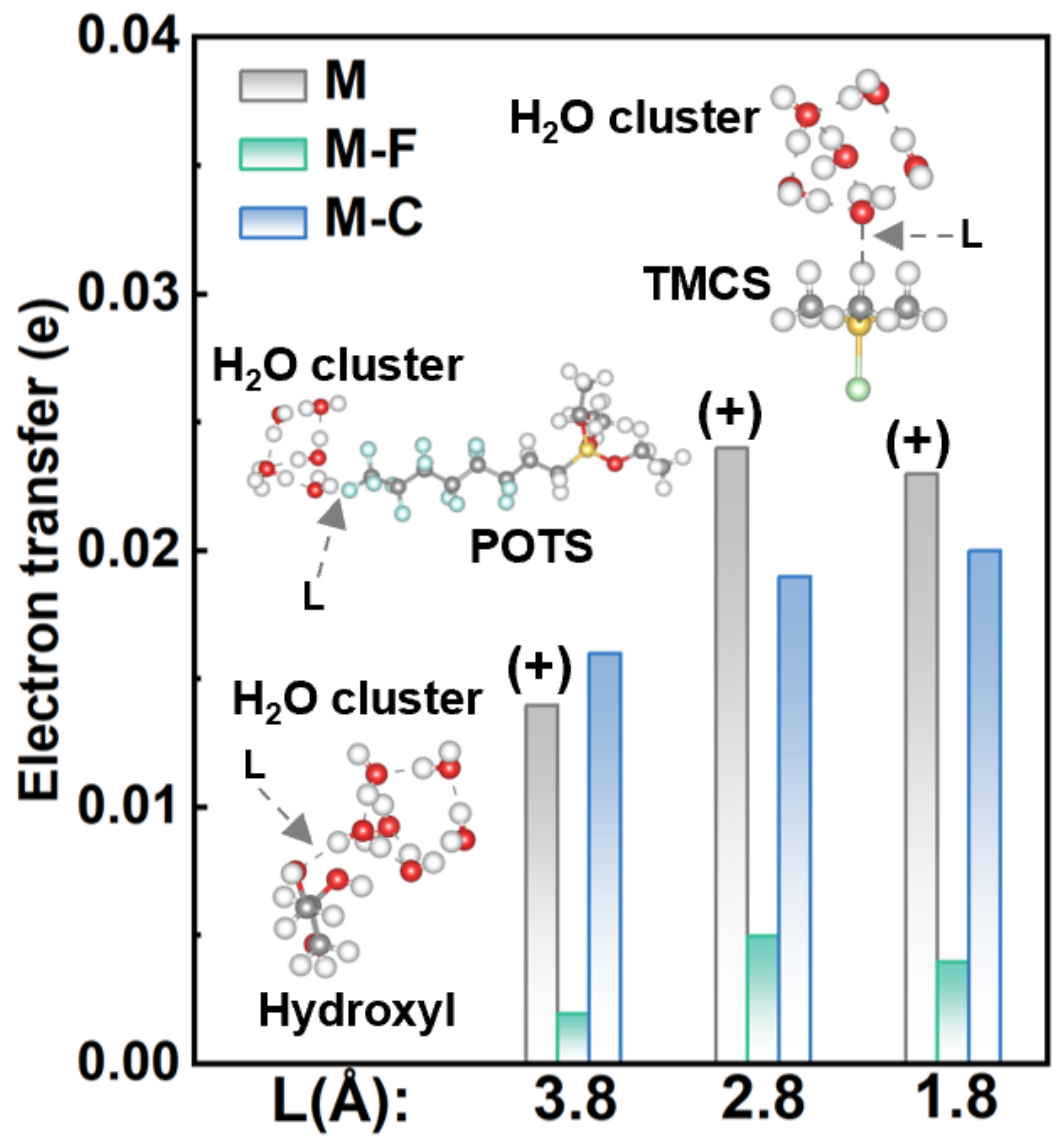

Fig. R1. Electron transfer between hydrophilic/hydrophobic molecules and a hexameric water cluster as a function of distance based on DFT simulations. (Fig. 2d)

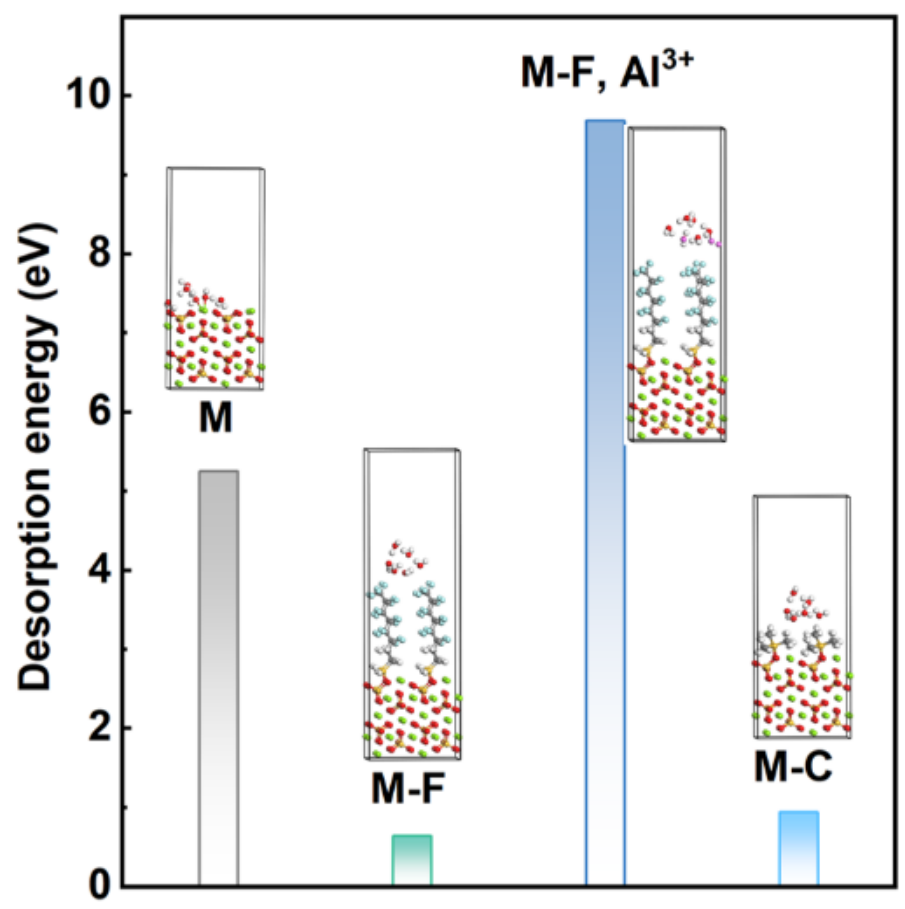

Fig. R2. Desorption energy of a hexameric water cluster on hydrophilic and hydrophobic mineral surfaces calculated by DFT simulations. (Fig. 2e)

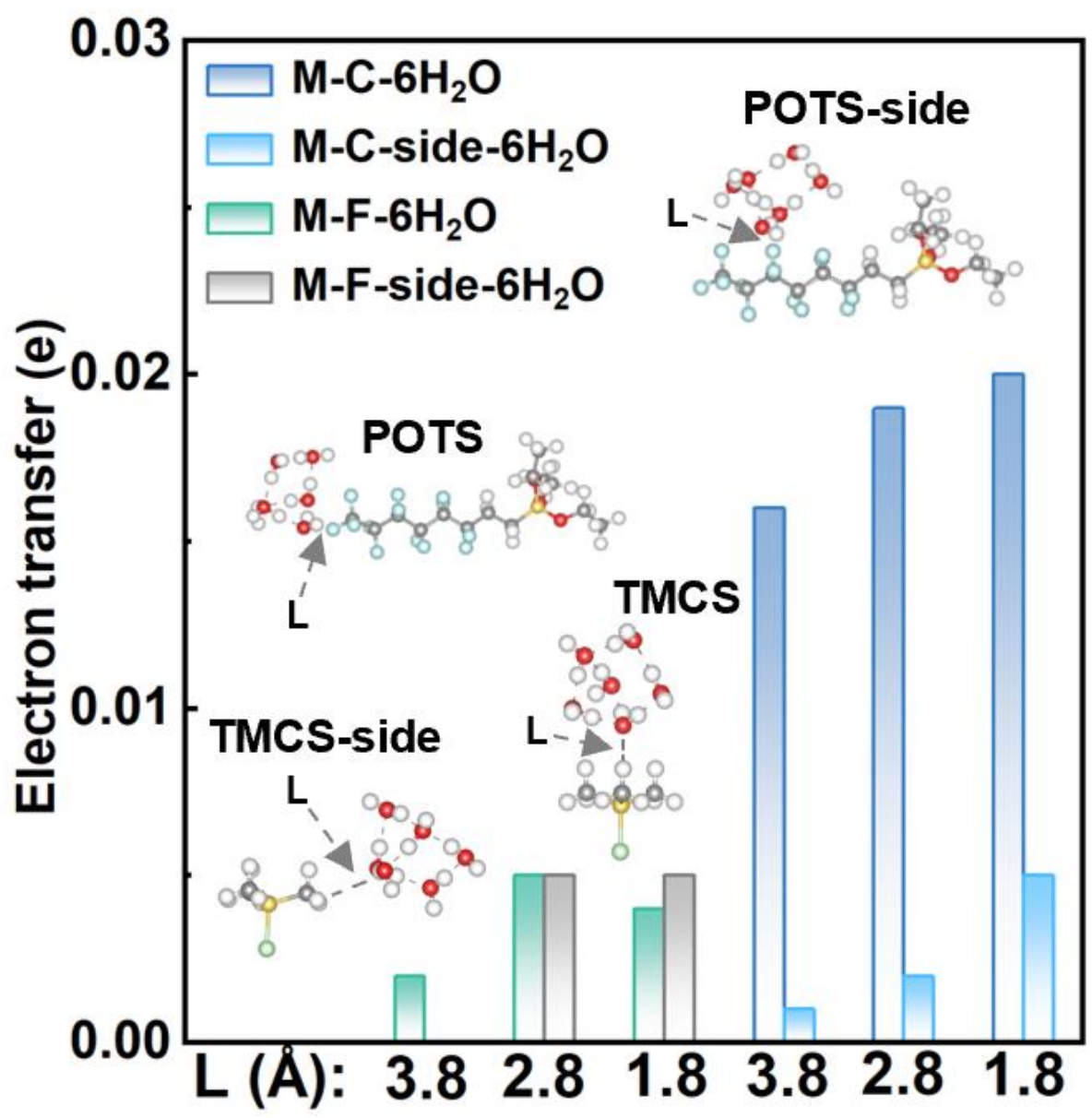

Fig. R3. Electron transfer between hydrophobic molecules and a hexameric water cluster as a function of configurations of hydrophobic molecules based on DFT simulations. (Supplementary Fig. 14)

Pages 13-14, lines 234-250: "DFT calculations were performed to further clarify how interfacial contact affects CEC performance by analyzing electron transfer (Fig. 2d), desorption energy (Fig. 2e), and energy band structures (Fig. 2f). For all three samples, the amount of electron transfer increased as the contact distance with water decreased. M acted as an electron donor to water, in contrast to M-C and M-F, which were electron acceptors. Despite M having the greatest electron transfer, its high desorption energy (Fig. 2e, 5.25 eV) limited the completion of the CEC process, resulting in poor performance. In contrast, both M-C and M-F exhibited similarly low desorption energies, allowing easy detachment of water molecules to complete the CEC (Fig. 2e). Moreover, compared with M-F, M-C showed better electron transfer (Fig. 2d), which can be attributed to its favorable wettability, facilitating better contact with water (Fig. 2c) despite its weaker electron-withdrawing ability (Fig. 2f). This favorable balance

between water approach and removal gave M-C a performance advantage over M-F. In addition, the contact position between the modifiers and the water cluster was also regulated (Supplementary Fig. 14), and the interaction between a single water molecule and the samples was further calculated (Supplementary Fig. 15-16), both of which supported the positive role of moderately hydrophobic regulation in enhancing CEC performance."

Comment 4: Statistical treatment (error bars, replicate numbers) should be clarified for key figures, particularly Figs. 1d and 3c.

Reply: Thank you for your valuable observation. The relevant information (Fig. R4) has been added in the manuscript as follows.

Page 6, lines 100-101: "H_2O_2 production (using 10 samples for error bar analysis) and contact angles of M-F."

Page 16, lines 284-285: "Comparison of CEC performance under different mechanical forces, including ultrasonication and circulating water flow (per sample with 10 parallel samples)."

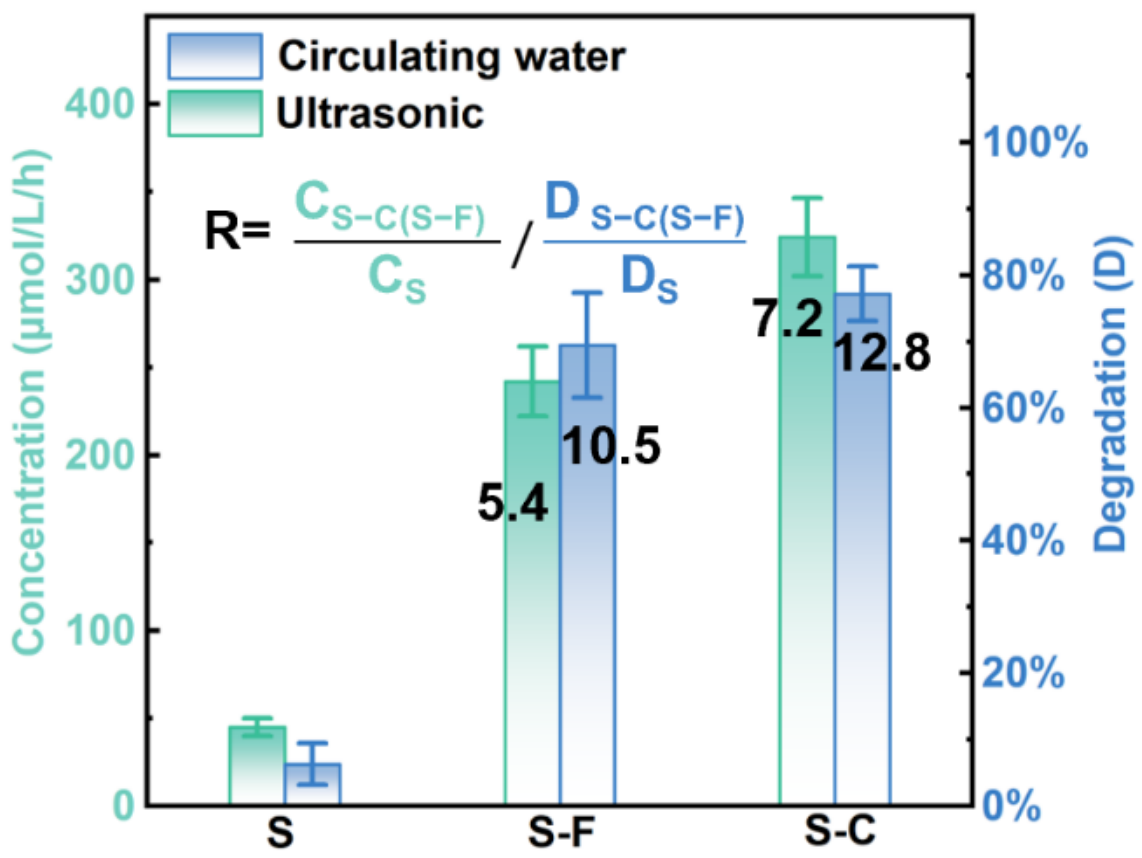

Fig. R4. Comparison of CEC performance under different mechanical forces, including ultrasonication and circulating water flow (per sample with 10 parallel samples). (Fig. 3c)

Comment 5: The influence of ionic strength and cation adsorption on zeta potential and hydrophobicity could be discussed more clearly.

Reply: We thank the reviewer for raising this important issue. We agree that zeta potential may affect cation adsorption and the effective hydrophobicity of catalyst surfaces in aqueous media. In the revised manuscript, we have added clarification that these factors were considered here only to provide a more realistic representation of how the water environment may alter surface wettability relative to the air-phase contact angle measurement. Our zeta-potential results and cation-related DFT analysis were therefore intended as supporting evidence for environmental effects on interfacial contact behavior.

However, we would also like to clarify that a systematic investigation of ion-specific adsorption, electrical double layer formation, or capacitance effects on CEC is beyond the scope of the present study. The central focus of this study is the regulation of catalyst-water contact through hydrophobicity tuning, and our conclusions are based on that contact-regulation framework rather than on the electric double layer-capacitance mechanism. We have revised the manuscript to make this scope more explicit and to avoid implying a deeper mechanistic interpretation of ionic-strength effects than is currently supported by the data.

We have revised the manuscript as follows.

Pages 14-15, lines 251-267: "Moreover, exchangeable cations in minerals and aqueous environments may influence the effective interfacial wettability of M-F under practical conditions. Because M-F has a more negative zeta potential (–6.9 to –35.2 mV) than M-C does (–3.0 to –23.7 mV), it is more susceptible to the adsorption of metal ions in water[36-38], including mineral-derived ions and environmentally relevant cations such as Al^{3+}, Ca^{2+} and Mg^{2+}**.** Such ion adsorption may shift the catalyst toward a more hydrophilic state in aqueous media, rather than the hydrophobic behavior suggested by air-based contact angle measurements. Consistently, the results of DFT calculations indicate that cation-associated conditions can increase the water cluster desorption

energy (9.69 eV in Fig. 2e), implying that the aqueous environment may alter the apparent interfacial behavior of M-F and thereby partly suppress its CEC performance (Fig. 2g). In contrast, M-C, with its less negative surface potential, is expected to be less affected by such ion-induced perturbation. We therefore consider cation adsorption here as an auxiliary factor that may modify the effective hydrophobicity/hydrophilicity of the catalyst in water, which may further contribute to the better CEC performance of M-C relative to M-F under practical conditions. A systematic analysis of ion-specific adsorption or electric double-layer effects, however, is beyond the scope of the present study."

Comment 6: The Introduction is somewhat lengthy and repetitive; streamlining would improve readability.

Reply: Thank you for your valuable suggestion and insightful observation. We have revised the introduction section of the manuscript as follows.

Pages 2-5, lines 42-91: "Contact-electro-catalysis (CEC) is an emerging catalytic process in which mechanical energy drives interfacial electron transfer between solids and water, leading to the generation of reactive oxygen species (ROS) [1, 2]. Because ROS can mediate pollutant degradation[3], metal recovery[4] and H_2O_2 production[5], CEC has attracted increasing attention as a potentially strategy for environmental and chemical transformations. To date, ultrasonic excitation has been the dominant approach for activating CEC because it provides sufficient energy to induce electron-cloud overlap between H_2O and catalyst surfaces[6]. However, ultrasonic systems are energy intensive, instrument dependent and often accompanied by noise pollution, which limits their broader practical deployment[7,8]. In contrast, hydrodynamic forces such as flowing water represent a more sustainable and widely available energy source, but CEC under such weak-force conditions remains underexplored.

Current CEC systems operating under weak mechanical excitation still rely largely on perfluorinated materials, such as fluorinated ethylene propylene (FEP) and polytetrafluoroethylene (PTFE), owing to their strong electron-withdrawing ability[9-11].

However, the CEC is not determined solely by the electron-transfer ability; it also depends on the effectiveness of the catalyst-water interfacial contact. The strong hydrophobicity associated with fluorinated surfaces may reduce the probability of water approaching the catalyst, thereby limiting productive interfacial electron transfer. Although this limitation can be partly masked under intense ultrasonic excitation, it is expected to become much more important under weak-force conditions, where interfacial contact is inherently less intense. Therefore, maximizing fluorination may not be the optimal design strategy for CEC under realistic hydrodynamic environments. Instead, regulating catalyst wettability to balance the water approach and water removal may offer a more effective route to improve CEC performance.

Natural minerals provide an attractive platform for testing this idea because they are abundant, environmentally compatible and widely involved in surface-mediated redox reactions[12,13], ROS generation[14-17], contaminant transformation and biogeochemical cycling in natural systems[18-23]. Silicate minerals are particularly suitable because they constitute more than 90% of the Earth's crust and possess abundant surface hydroxyl groups that can be chemically tuned to regulate hydrophilicity and hydrophobicity. Moreover, natural hydrodynamic settings such as rivers, waterfalls, rainfall, and tidal flows provide continuous low-grade mechanical energy that may drive mineral–water interfacial CEC over large spatial and temporal scales[24-26], which may drive mineral-water interfacial CEC over large spatial and temporal scales. However, whether mineral-based CEC can operate effectively under natural weak-force conditions and how interfacial wettability governs this process remain largely unknown.

Here, we develop a hydrophobicity-regulation strategy for CEC on natural silicate minerals to optimize interfacial water transfer. As a proof of this strategy, alkyl-functionalized minerals are used to construct a non-fluorinated catalyst (M-C) with moderate hydrophobicity, which outperforms both the hydrophilic raw mineral (M) and the more hydrophobic fluorinated mineral (M-F). Combining experiments under ultrasonication, circulating water flow, and natural river conditions with a theoretical analysis based on extended Lennard-Jones (L-J) interactions and density functional theory (DFT), we show that moderate hydrophobicity enhances the CEC by balancing

the water approach and water removal at the interface. We further demonstrate that this strategy is effective under weak-force and natural hydrodynamic conditions, where it reshapes interfacial chemistry and surface-associated microbial communities. These findings establish hydrophobicity regulation as a contact-optimization principle for CEC and broaden its relevance from fluorinated laboratory systems to environmentally relevant mineral-water interfaces."

Comment 7: Several grammatical and stylistic issues should be corrected during revision.

Reply: Thank you for your valuable suggestion and insightful comment. We have revised the grammar of the entire manuscript and performed professional editing by native English researchers.

Reviewer #3 (Remarks to the Author):

The manuscript has been substantially improved and many textual/presentation comments were addressed. However, several mechanistic and control-related reviewer concerns remain only partially resolved, especially Fenton/metal-background chemistry, flow-driven ROS evidence, and light/adsorption controls for MO degradation. Below are some comments in detail:

We thank the reviewer for this important and insightful comment. Regarding the questions on flow-driven ROS evidence and the light/adsorption controls for MO degradation, each point is addressed in detail below under "Comments in Detail." Here, we first provide the response concerning Fenton/metal-background chemistry.

Question: Fenton/metal-background chemistry

Response: We agree that, because CEC produces H_2O_2 and some natural minerals contain transition metals, a Fenton-like process is theoretically possible and should be considered carefully.

In our system, however, metal-catalyzed background reactions are expected to contribute only a limited ROS generation compared with the dominant CEC process. As noted by the reviewer, Fenton or Fenton-like reactions generally require accessible redox-active metal species. In our case, XPS analysis shows that the surface Fe content of the representative fluorinated olivine sample is about 1% (Fig. R1). Similarly, the crystal sand used in the circulating water-flow experiments contains only about 1% surface Fe (Fig. R2). Furthermore, the Fe2p spectra of the other nine silicate minerals also do not exhibit any obvious characteristic peaks (Fig. R3). At such low surface concentrations, and with these metals predominantly incorporated within the silicate lattice rather than existing as freely dissolved ions, their capacity to drive substantial Fenton-like reactions is considered limited.

Although CEC-coupled Fenton systems have been reported in previous studies, those systems typically involved the deliberate addition of relatively high concentrations of Fe ions (1 mM) to enhance the conversion of H_2O_2 to •OH [1,2]. In contrast, no external Fe ions were introduced in the present experiments. Therefore, the experimental

conditions differ fundamentally from those intentionally designed to promote strong Fenton chemistry.

Moreover, the primary performance metric evaluated in this study is H_2O_2 production during CEC. A Fenton-like reaction would consume H_2O_2 to generate •OH. Consequently, even if a minor Fenton-like contribution occurred, it would not artificially inflate the measured H_2O_2 production. Instead, such a side reaction would tend to decrease the detectable H_2O_2 level.

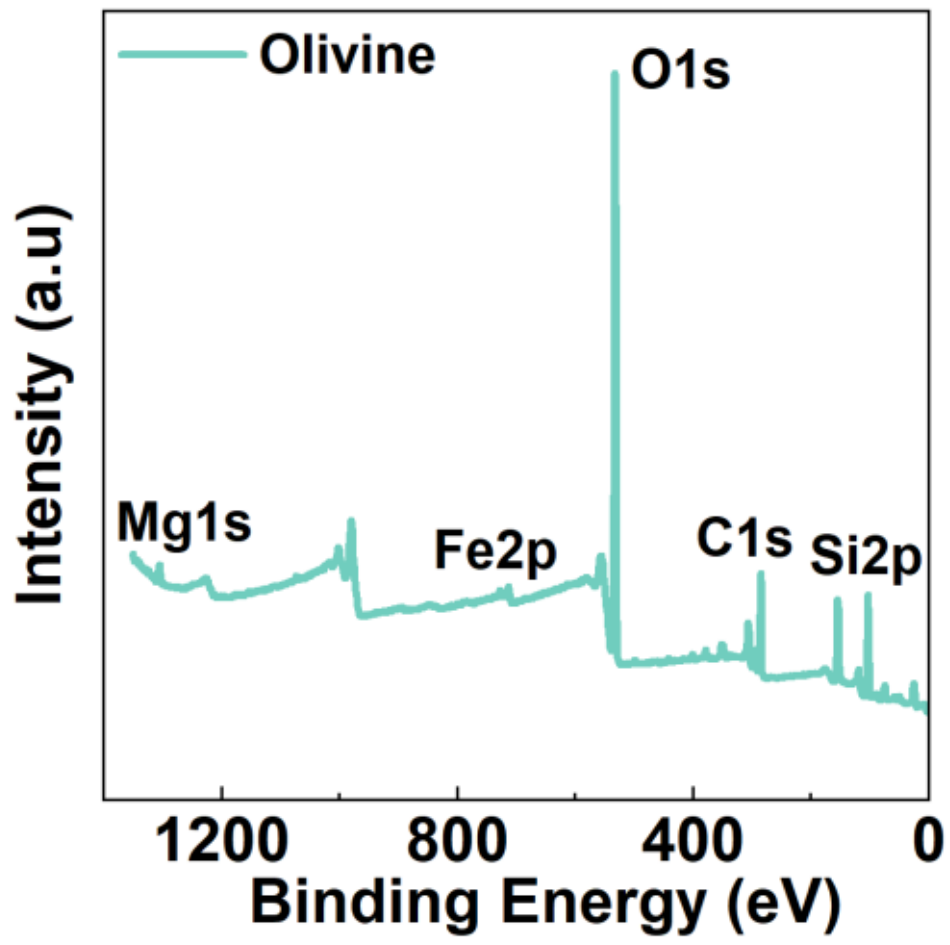

Fig. R1. The total element XPS spectrum of olivine mineral.

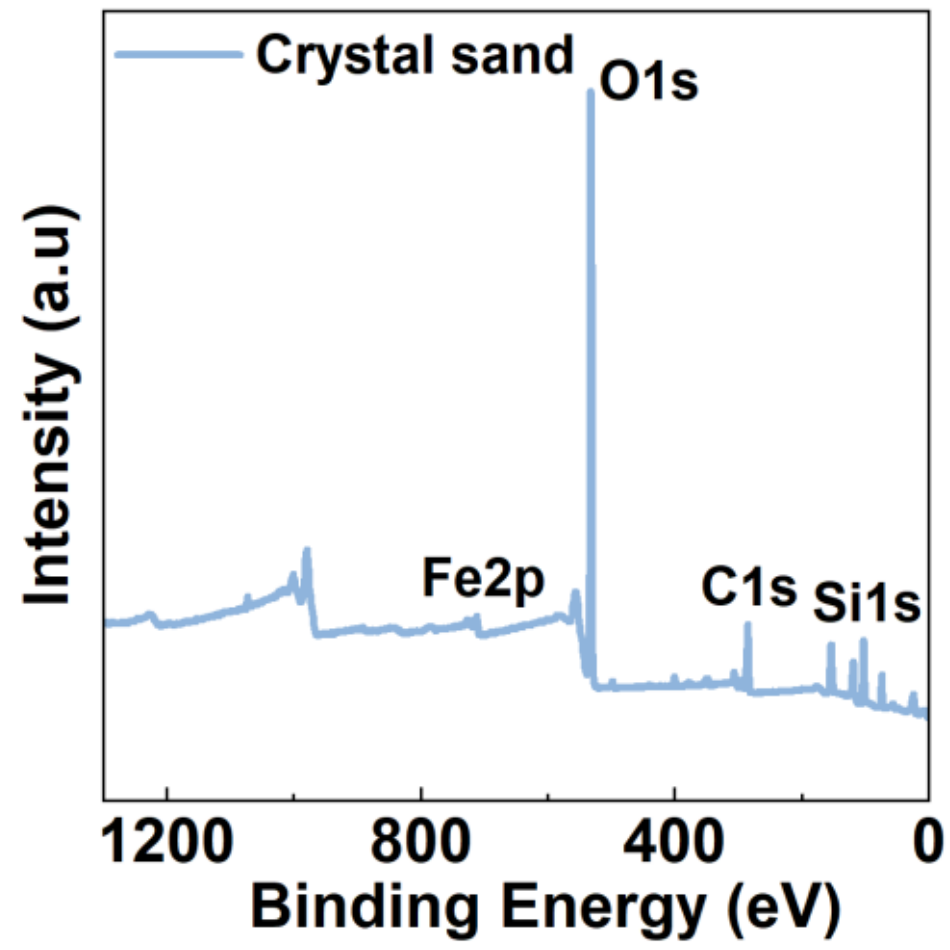

Fig. R2. The total element XPS spectrum of crystal sand.

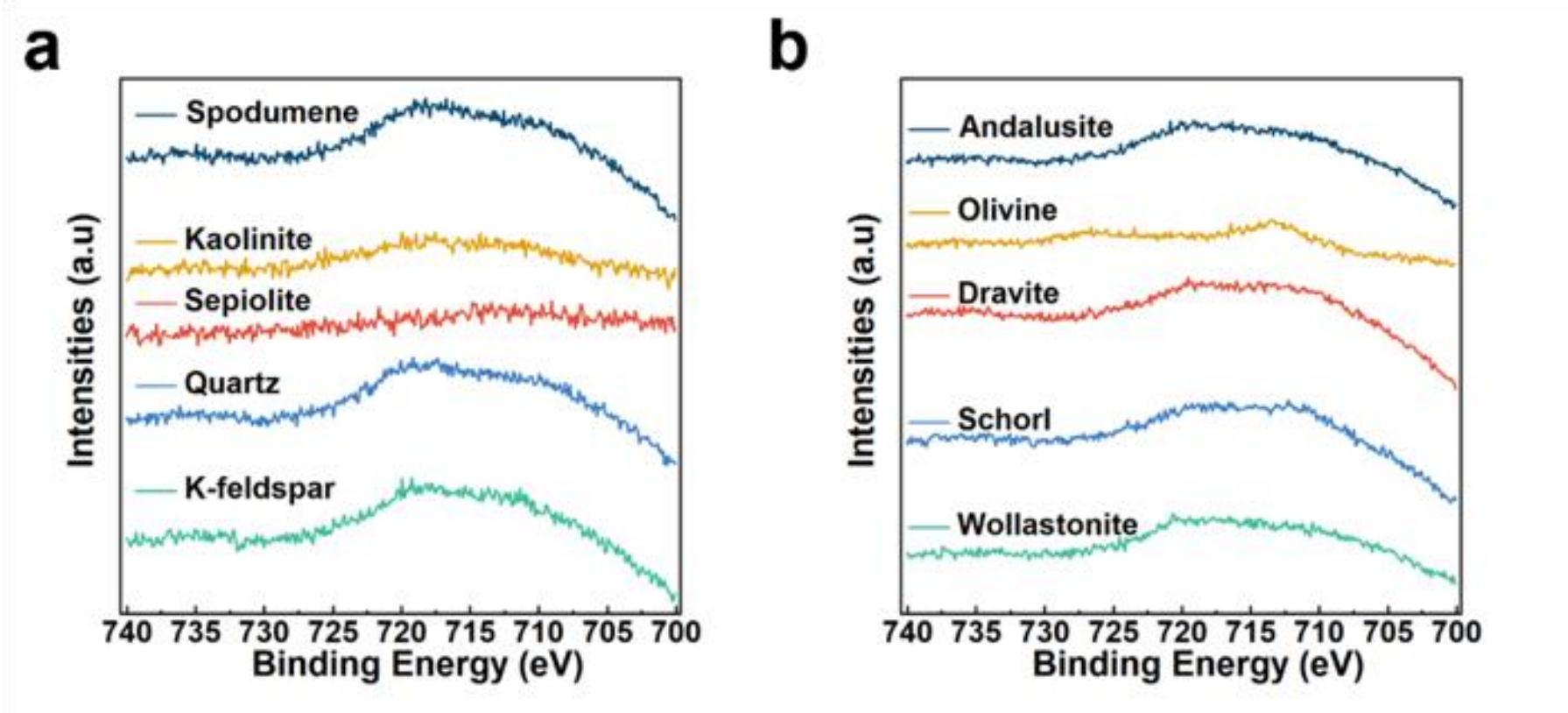

Fig. R3. The Fe 2p XPS spectra of original silicate minerals.

References:

[1] Ma D, et al. Fe III-driven self-cycled Fenton via contact-electro-catalysis for water purification. *npj Clean Water* **8**, 42 (2025).

[2] Li W, et al. Boosting Reactive Oxygen Species Generation via Contact-Electro-Catalysis with Fe III-Initiated Self-cycled Fenton System. *Angew. Chem. Int. Ed* **64**, e202413246 (2024).

The corresponding content has been added to the Manuscript and Supporting Information.

Page 10, lines 185-189 in Manuscript: "In addition, XPS analysis shows only negligible Fe signals in the tested minerals, suggesting that Fe-mediated Fenton-like reactions are unlikely to substantially contribute to ROS generation or affect the evaluation of CEC performance (Supplementary Fig. 13)"

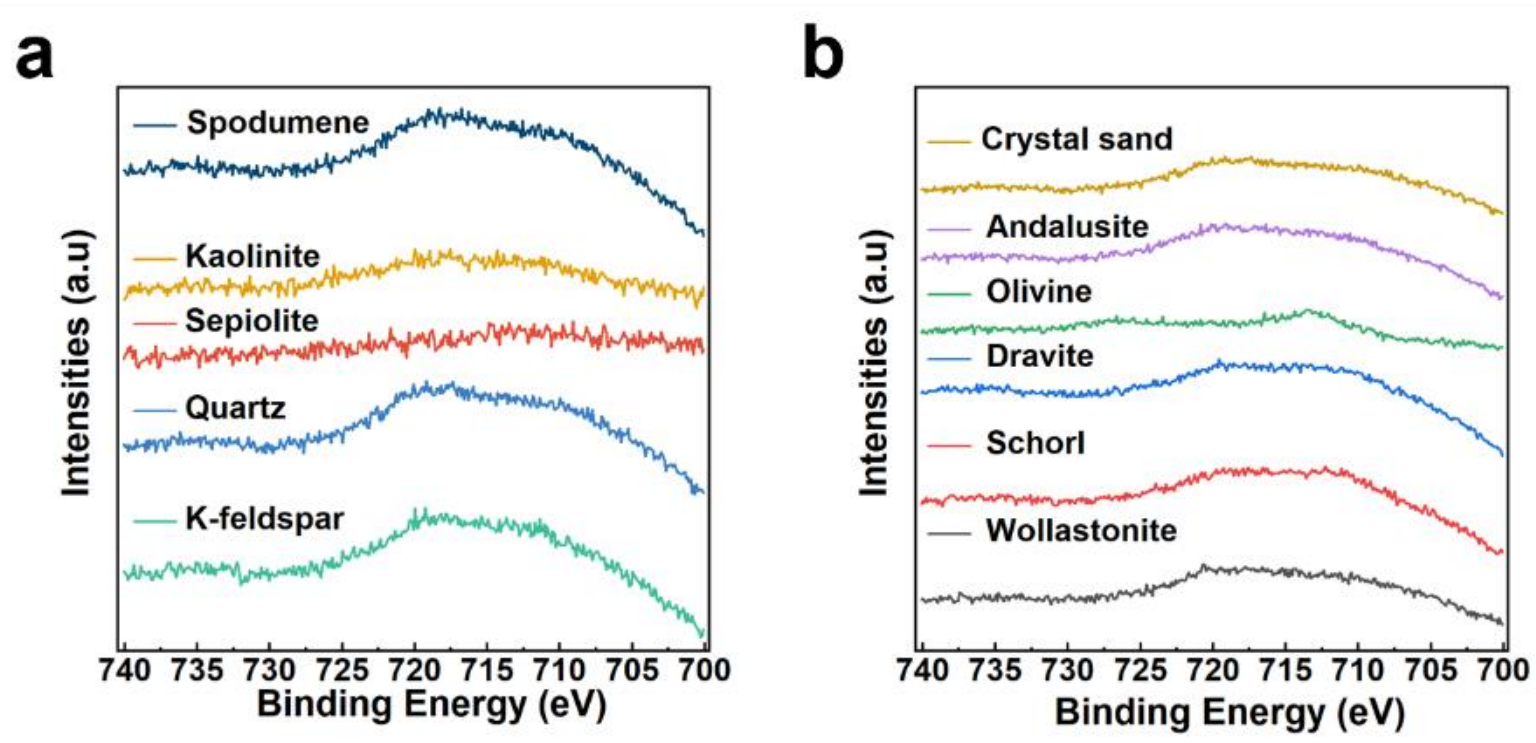

Fig. R4. The Fe 2p XPS spectra of original silicate minerals and crystal sand. (Supplementary Fig. 13)

Pages 22-23, lines 348-360 in Supporting Information: "Metal-catalyzed background reactions are expected to contribute only a limited ROS generation compared with the dominant CEC process. Fe2p spectra of the ten silicate minerals do not exhibit any obvious characteristic peaks (Supplementary Fig. 13). At such low surface concentrations, and with these metals predominantly incorporated within the silicate lattice rather than existing as freely dissolved ions, their capacity to drive substantial Fenton-like reactions is considered limited. Similarly, the crystal sand later used in the circulating water-flow experiments contains little surface Fe.

Moreover, the primary performance metric evaluated in this study is H_2O_2 production during CEC. A Fenton-like reaction would consume H_2O_2 to generate •OH. Consequently, even if a minor Fenton-like contribution occurred, it would not artificially inflate the measured H_2O_2 production. Instead, such a side reaction would tend to decrease the detectable H_2O_2 level."

Question 1: The flow-driven MO degradation experiment needs special attention. The Methods state that MO degradation was conducted "under xenon lamp irradiation." If that is correct, then the authors must include no-catalyst light controls, dark flow controls, catalyst-without-flow controls, and possibly radical scavenger controls under the same illumination. If the xenon lamp statement is a leftover error, it should be removed immediately.

Response: We thank the reviewer for pointing out this important issue. The xenon lamp irradiation used in the flow-driven MO degradation experiment was intentional rather than a leftover error. Xenon lamp was introduced based on previously reported weak-force CEC studies [1], in which light was suggested to facilitate electron consumption and help restore the catalyst state, thereby reducing catalyst poisoning. However, we agree that additional experiments are necessary to assess the effects of xenon lamp irradiation as well as other factors influencing the reaction.

Accordingly, we have added the corresponding control experiments in the revised Supporting Information, including no-catalyst controls under xenon lamp and dark conditions, catalyst-containing flow experiments under xenon lamp and dark conditions,

and quantitative experiments of free radicals under xenon lamp and dark conditions (Fig. R5a). The results show that xenon lamp irradiation alone caused approximately 8% direct MO degradation. However, after accounting for this xenon lamp background, illumination did not produce an observable enhancement of the CEC performance. These results indicate that the xenon lamp introduced a measurable but limited photolysis background and did not affect the comparative evaluation of the flow-driven CEC performance.

The newly added results of quantitative experiments of free radicals further support this interpretation. Under illumination, the $\bullet O_2^-$ signal increased markedly, whereas it was nearly absent under dark conditions. In contrast, the •OH signal remained relatively stable with or without xenon lamp (Fig. R5b). This indicates that light mainly promotes the oxygen reduction-related pathway, likely by facilitating electron consumption and thereby reducing catalyst poisoning, whereas the dominant CEC pathway remains associated with water oxidation and •OH generation [1-3]. This conclusion is consistent with the stable MO degradation result with or without xenon lamp irradiation and the ^{18}O isotope labeling results showing that •OH originates from water during CEC. We have revised the Supporting Information to clarify the role of xenon lamp irradiation and to state explicitly that the flow-driven CEC performance comparison is not governed by light-induced MO degradation.

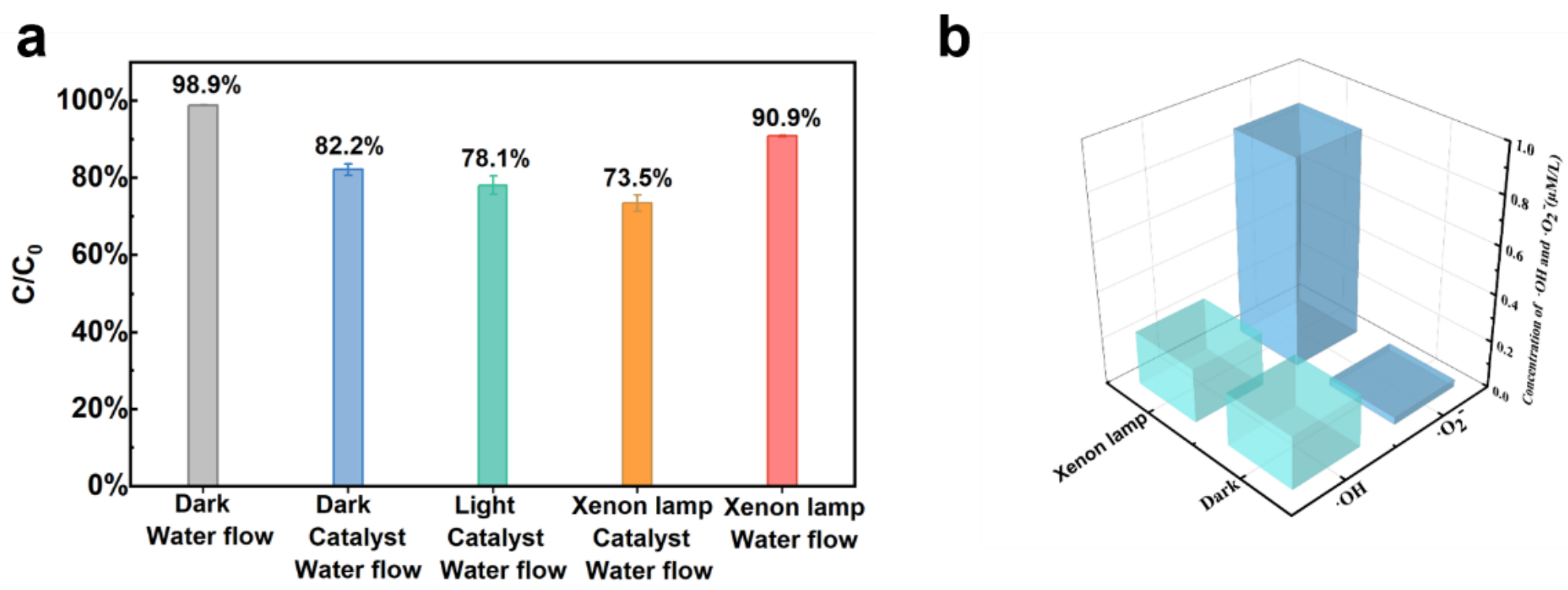

Fig.R5 The control experiment with xenon lamp irradiation. a. MO degradation in no-catalyst controls under xenon lamp and dark conditions; MO degradation by catalyst-containing systems under flow conditions with and without xenon lamp irradiation (dark, natural light, and xenon lamp light). b. Quantitative experiments of free radicals with or without xenon lamp irradiation.

(Supplementary Fig. 24).

References:

[1] Song WZ, et al. Insulator polymers achieve efficient catalysis under visible light due to contact electrification. *Water Res* **226**, 119242 (2022).

[2] Zhang M, et al. Rotation-mode liquid-solid triboelectric nanogenerator for efficient contact-electro-catalysis and adsorption. *Nano Energy* **110**, 108329 (2023).

[3] Dong F, et al. Advanced Triboelectric Materials for Contact Electrocatalytic Degradation of Pollutants. Small **21**, 2500369 (2025).

Page 28, lines 521-524 in Manuscript: The xenon lamp was introduced to reduce catalyst poisoning, but did not affect the comparative evaluation of the flow-driven CEC performance (Supplementary Fig. 24 and Supplementary Note. 7)

Pages 12-13, lines 200-240 in Supporting Information:

Supplementary Note 7. Effect of xenon lamp irradiation on flow-driven MO degradation

The flow-driven methyl orange (MO) degradation experiments were conducted under xenon lamp irradiation. This illumination condition was intentionally introduced based on previous weak-force CEC studies, in which light was suggested to facilitate electron consumption and help restore the catalyst state, thereby reducing catalyst poisoning[23-25]. Nevertheless, because xenon lamp irradiation may introduce direct photolysis or alter radical-generation pathways, additional control experiments were conducted to clarify its role in the flow-driven MO degradation system.

To distinguish flow-driven CEC from light-induced background degradation, control experiments were performed under xenon lamp and dark conditions. These experiments included no-catalyst controls under xenon lamp and dark conditions, catalyst-containing flow experiments under xenon lamp and dark conditions, and quantitative experiments of free radicals under xenon lamp and dark conditions. As shown in Supplementary Fig. 24a, xenon lamp irradiation alone caused approximately 8% MO degradation in the absence of catalyst, whereas MO removal under the dark no-catalyst

condition was negligible. This result confirms that xenon lamp irradiation introduced a measurable but limited photolysis background.

Catalyst-containing flow experiments further showed that illumination did not produce an observable enhancement of the flow-driven CEC performance after accounting for the xenon lamp background. The degradation efficiencies obtained under xenon lamp and dark conditions were comparable, and the relative catalytic trend remained unchanged. Therefore, the xenon lamp background did not affect the comparative evaluation of flow-driven CEC performance among the tested catalyst systems.

Quantitative experiments of free radicals were further conducted to clarify the effect of xenon lamp irradiation on radical-generation pathways. Under xenon lamp irradiation, the $•O_2^-$ signal increased markedly, whereas it was nearly absent under dark conditions. In contrast, the •OH signal remained relatively stable with or without xenon lamp irradiation (Supplementary Fig. 24b). These results indicate that light mainly promotes the oxygen reduction-related pathway, likely by facilitating electron consumption and thereby reducing catalyst poisoning, whereas the dominant CEC pathway remains associated with water oxidation and •OH generation[23-25]. This interpretation is consistent with the stable MO degradation result with or without xenon lamp irradiation and the ^{18}O isotope labeling results showing that •OH originates from water during CEC.

Overall, xenon lamp irradiation introduced a limited photolysis background and enhanced the auxiliary $•O_2^-$ pathway, but it did not govern MO degradation or alter the comparative evaluation of flow-driven CEC performance. The flow-driven degradation behavior is therefore primarily attributed to CEC associated with water oxidation-derived •OH generation.

Question 2: The microbial interpretation is now more cautious, but still not fully resolved. The authors should not claim that ROS alone drives microbial restructuring. The current data support a combined effect of hydrophobic surface selection, altered organic matter adhesion, and possible ROS-mediated oxidative pressure.

Response: Thank you for this important clarification. We agree that microbial restructuring likely results from a combination of interfacial factors, including hydrophobic surface-driven selection, differential organic matter adhesion, and potential ROS-related oxidative stress. We have further revised the relevant sections in the Results and Discussion to reflect on this multifactorial interpretation. The updated text now reads:

(1) Pages 22-23, lines 394-400: "Taken together, these results support a multifactorial restructuring mechanism in which hydrophobic surface selection, altered organic matter adhesion, and potential oxidative pressure from ROS collectively select for microorganisms that are both capable of metabolizing the newly generated organic substrates and tolerant to oxidative stress. It should also be noted that the present metagenomic data cannot disentangle the relative contributions of hydrophobic surface selection, organic matter adhesion, and ROS-mediated oxidative pressure."

(2) Page 22, lines 373-374: "… indicating selective enrichment **on the modified mineral surface**" (Replaced "in the CEC-active mineral environment")

Question 3: The hydrophobicity universality claim should be moderated. The data support "broadly observed among the tested silicate minerals under these conditions," not a universal rule across natural minerals or all hydrodynamic regimes.

Response: We appreciate this suggestion and agree that our conclusions regarding the role of hydrophobicity in natural minerals should be more carefully framed. We have adjusted the relevant statements throughout the manuscript to avoid overgeneralization. Specifically, we now describe the effect as "broadly observed among the tested silicate minerals under the studied conditions," rather than presenting it as a universal phenomenon. This change helps clarify the scope of our findings while maintaining the observed novelties.

The updated text now reads:

(1) Page 2, lines 34-35: "Together, these findings **identify** hydrophobicity regulation…." (Replaced "establish")

(2) Page 4, lines 84-88: "These findings support hydrophobicity regulation as a contact-

optimization strategy for CEC in the tested silicate mineral systems and suggest that balanced wettability may be relevant to environmentally occurring mineral-water interfaces under weak hydrodynamic excitation. ”

(3) Page 6, lines 113-114: “To examine whether the hydrophobic modification strategy is broadly applicable across the tested silicate minerals,…”

(4) Page 7, lines 126-127: “These results indicate that -CH_3 and -F surface modifications can broadly impart hydrophobicity to the tested silicate minerals under the present modification conditions. ”

(5) Page 23, lines 411-414: “Furthermore, such processes may be relevant to a broader range of low-energy aquatic systems, including groundwater, wetland, estuaries, and coastal zones, although this possibility requires validation under additional mineral compositions, water chemistries, biological settings, and hydrodynamic regimes. ”

(6) Page 25, lines 442-444: “This work demonstrates that hydrophobicity regulation can serve as a contact-optimization strategy for contact-electro-catalysis (CEC) at the tested silicate mineral-water interfaces. ”

(7) Pages 25-26, lines 458-463: “More broadly, these findings suggest that hydrophobicity-regulated mineral-water interfacial CEC may represent a previously underappreciated pathway under certain mineral-water and weak-flow conditions linking reactive oxygen species generation with surface reactions, organic matter transformation and microbial niche differentiation in natural aquatic environments. ”

Question 4: The abstract also appears to be about 190 words, while the Nature Communications formatting instructions and checklist specify an abstract limit of 150 words or fewer.

Response: Abstract with 150 words has been revised in Manuscript.

Pages 1-2, lines 21-35: “Contact-electro-catalysis (CEC) at catalyst-water interfaces shows promise for wastewater purification, metal recovery, and H_2O_2 production, but catalyst design has mainly emphasized electron-withdrawing ability while overlooking

interfacial water transfer. Here, we develop a hydrophobicity engineering strategy for natural silicate minerals to optimize catalyst-water contact by balancing the water approach and removal. As a representative non-fluorinated system, alkyl-modified minerals with moderate hydrophobicity (water contact angle of 125–148°) achieve a H_2O_2 yield of 350.0 $\mu mol \cdot L^{-1} \cdot h^{-1}$ under ultrasonication, outperforming fluorinated minerals. Mechanistic analysis indicates that this enhancement arises from improved interfacial contact efficiency rather than stronger electron-withdrawing ability alone. This strategy also remains effective under weak-force conditions, enabling self-powered CEC in a natural river. The modified minerals selectively enriched microorganisms associated with oxidative stress resistance and oxidized organic carbon utilization, including *Novosphingobium* sp. Together, these findings identify hydrophobicity regulation as an effective contact-optimization strategy for CEC."
